# QM calculations predict the energetics and infrared spectra of transient glutamine isomers in LOV photoreceptors†

**DOI:** 10.1039/d1cp00447f

**Published:** 2021-06-18

**Authors:** Prokopis C. Andrikopoulos, Aditya S. Chaudhari, Yingliang Liu, Patrick E. Konold, John T. M. Kennis, Bohdan Schneider, Gustavo Fuertes

**Affiliations:** Institute of Biotechnology of the Czech Academy of Sciences, BIOCEV Průmyslová 595 CZ-252 50 Vestec Czechia prokopios.andrikopoulos@ibt.cas.cz gustavo.fuertes@ibt.cas.cz; Department of Physics and Astronomy, Faculty of Sciences, Vrije Universiteit 1081 De Boelelaan 1081HV Amsterdam The Netherlands

## Abstract

Photosensory receptors containing the flavin-binding light-oxygen-voltage (LOV) domain are modular proteins that fulfil a variety of biological functions ranging from gene expression to phototropism. The LOV photocycle is initiated by blue-light and involves a cascade of intermediate species, including an electronically excited triplet state, that leads to covalent bond formation between the flavin mononucleotide (FMN) chromophore and a nearby cysteine residue. Subsequent conformational changes in the polypeptide chain arise due to the remodelling of the hydrogen bond network in the cofactor binding pocket, whereby a conserved glutamine residue plays a key role in coupling FMN photochemistry with LOV photobiology. Although the dark-to-light transition of LOV photosensors has been previously addressed by spectroscopy and computational approaches, the mechanistic basis of the underlying reactions is still not well understood. Here we present a detailed computational study of three distinct LOV domains: EL222 from *Erythrobacter litoralis*, AsLOV2 from the second LOV domain of *Avena sativa* phototropin 1, and RsLOV from *Rhodobacter sphaeroides* LOV protein. Extended protein-chromophore models containing all known crucial residues involved in the initial steps (femtosecond-to-microsecond) of the photocycle were employed. Energies and rotational barriers were calculated for possible rotamers and tautomers of the critical glutamine side chain, which allowed us to postulate the most energetically favoured glutamine orientation for each LOV domain along the assumed reaction path. In turn, for each evolving species, infrared difference spectra were constructed and compared to experimental EL222 and AsLOV2 transient infrared spectra, the former from original work presented here and the latter from the literature. The good agreement between theory and experiment permitted the assignment of the majority of observed bands, notably the ∼1635 cm^−1^ transient of the adduct state to the carbonyl of the glutamine side chain after rotation. Moreover, both the energetic and spectroscopic approaches converge in suggesting a facile glutamine flip at the adduct intermediate for EL222 and more so for AsLOV2, while for RsLOV the glutamine keeps its initial configuration. Additionally, the computed infrared shifts of the glutamine and interacting residues could guide experimental research addressing early events of signal transduction in LOV proteins.

## Abstract 2

The isomerisation of a conserved glutamine residue along the early photocycle of 3 LOV domains is explored by QM calculations, revealing IR spectral shifts upon rotation in agreement with time-resolved experiments.

## Graphical abstract

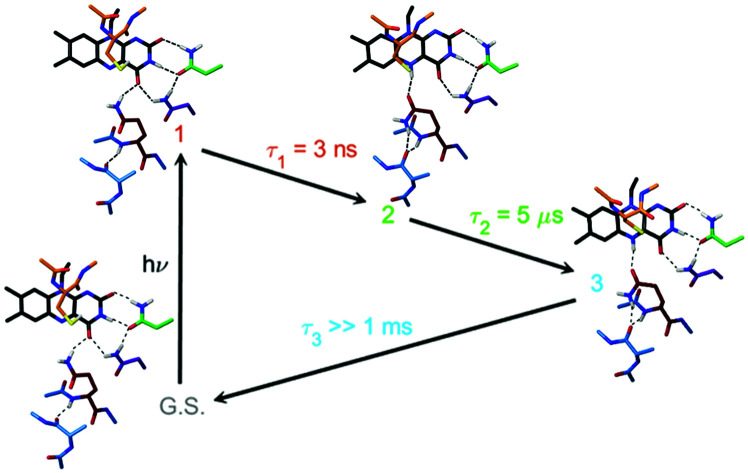

## 1. Introduction

**Fig. 1:**
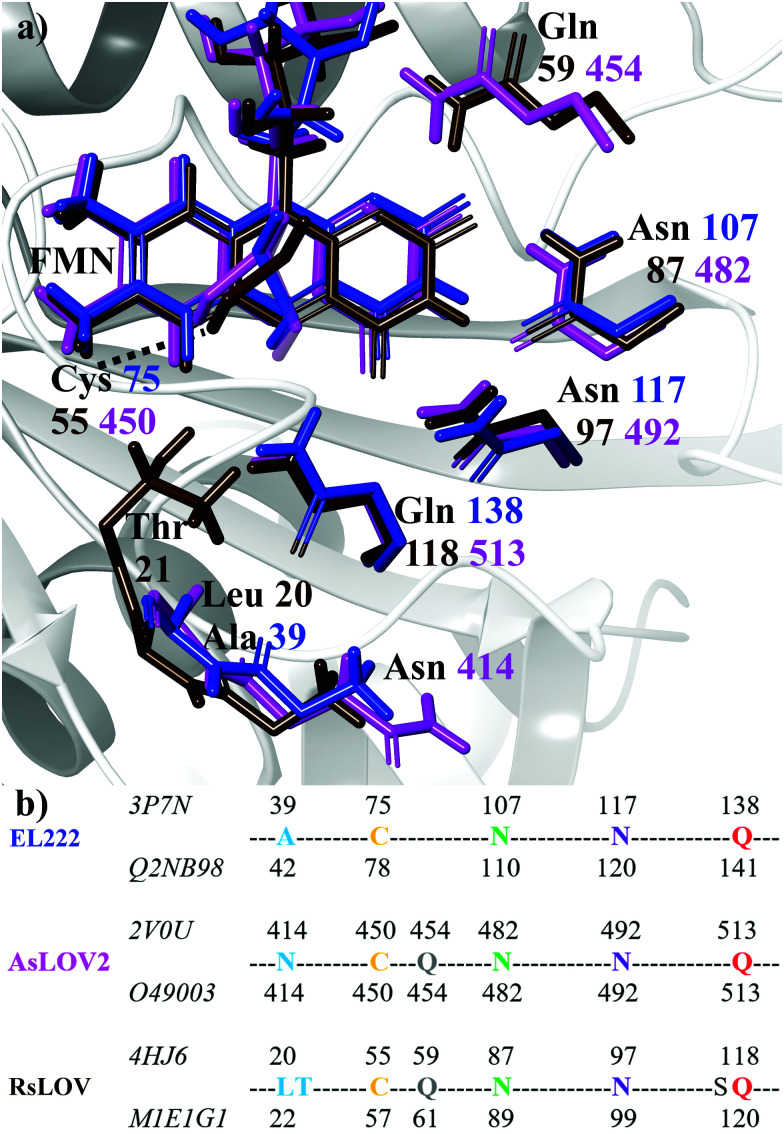
(a) Superposition of key residues of the crystal structures of dark state EL222 (blue), AsLOV2 (magenta) and RsLOV (brown). Labels show the residue number colour-coded to each crystal structure. The structure of the EL222 protein is shown in the background with grey ribbons. (b) Sequence alignment of the three LOV proteins used in this study. For each protein, the residue numbers according to the Protein Data Bank and UniprotKB databases are shown above and below their sequences, respectively (the corresponding IDs are displayed on the left).

Flavin-binding photosensory proteins containing the light-oxygen-voltage (LOV) domains, a subset of the sensory Per-Arnt-Sim (PAS) domain, serve many biological functions (*e.g.* phototropism) and are also invaluable tools in optogenetics.^1–3^ Upon photoexcitation of the embedded chromophore, typically flavin mononucleotide (FMN), the signal is allosterically transmitted to adjacent domains, eventually resulting in intramolecular conformational changes, oligomerisation/dissociation events, or binding to other biomolecules.^4^ Apart from sharing a common isoalloxazine ring, a number of residues are conserved in the ligand binding pocket (Fig. 1a). These include two asparagines that envelop FMN within the active site through non-covalent interactions, the adduct-forming cysteine, and a glutamine residue. The latter is postulated to play a cardinal role in signal transduction by acting at the interface between the chromophore and the polypeptide chain. Specifically, the side chain of the conserved glutamine may trigger – through a 180° rotation after the H-transfer step – a process of structural changes by altering the hydrogen bond map of the protein.^5–12^

**Fig. 2:**
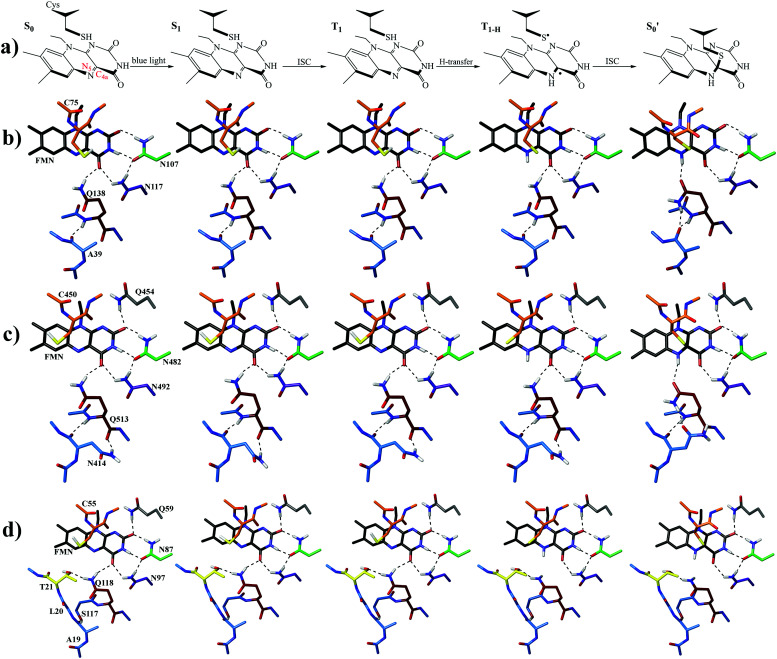
(a) Scheme showing the five key intermediates in the LOV domain photocycle. Optimised structures of (b) EL222 (c) AsLOV2 and (d) RsLOV along the reaction coordinate. The hydrogen bonding network is shown with black dashed lines. ISC = intersystem crossing.

The photocycle of LOV domains commences typically *via* a blue light trigger that excites the ground state flavin (S_0_) rapidly to the singlet state (S_1_) within femtoseconds (Fig. 2a). Then *via* intersystem crossing (ISC) the triplet state is reached (T_1_), with typical lifetimes of 2–3 ns. At this stage, the chromophore is primed for the H-transfer from the sulfhydryl (–SH) side chain of a conserved cysteine to the N_5_ isoalloxazine atom to form the reactive triplet biradical (T_1-H_). The mechanism of H-transfer (simultaneous/concerted) will not be investigated here, only the resultant neutral biradical species. Subsequently, a covalent bond is formed between the cysteine sulfur and flavin C_4a_ atoms with a concomitant crossing to the electronic ground state at typical time scales of 0.2–20 μs,^13–17^ which is indicated by an absorption signal at 390 nm.^18^ Once the biradical (T_1-H_) or the adduct 
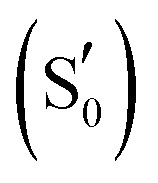
 intermediates have been formed in the LOV domain, large scale effects are initiated,^19,20^ which ultimately enable the parental full-length protein to perform its assigned function.

In particular, EL222 of *Erythrobacter litoralis* has its effector helix-turn-helix (HTH) domain bound to the photoactive LOV domain in the dark.^21^ Illumination exposes the HTH domain and allows the protein to dimerise, bind to DNA and activate gene transcription.^22^ Conversely, RsLOV the LOV protein of *Rhodobacter sphaeroides*, has still an unclear physiological function, but it is known to self-associate as dimer in the dark.^23^ RsLOV forms the FMN–cysteine adduct in adequate yields, and dissociates to a monomeric state under illumination, as evidenced by fluorescence experiments.^24^ Finally, AsLOV2 is the most photosensitive of the two LOV domains present in phototropin 1 of *Avena sativa*, and is reported to assist the autophosphorylation of the kinase domain, ultimately promoting plant growth.^25^ It is postulated that a vital role in the AsLOV2 ability to propagate signals is played by the unfolding of its Jα helix.^26^ The metastable cysteine–FMN covalent bond is cleaved in the dark on timescales ranging from seconds to hours depending on the particular combination of LOV domain and the associated effector modules.^27^

To monitor the above light-triggered changes involved in LOV signal transduction, time-resolved vibrational spectroscopy, and in particular transient infrared spectroscopy, has proven to be an invaluable instrument in the researchers’ toolbox.^7,28–34^ A challenge is posed by the need to separate spectral contributions originating from the chromophore and the protein environment, a task that can be approached by a combination of (i) analysis of the spectra of the isolated chromophore,^35,36^ (ii) isotopic labelling of key residues such as the glutamine (achieved currently only for a BLUF domain),^37^ and the chromophore,^33,38^ (iii) mutation of key residues^32,39,40^ or (iv) by inserting non-canonical amino acid probes in the protein sequence.^41^ More recently, femtosecond-stimulated Raman experiments indicate that FMN modes can be selectively enhanced under appropriate resonance conditions.^35,42,43^

Computational efforts have been instrumental in the elucidation of large scale changes of LOV domains *via* molecular dynamics simulations.^6,7,44–49^ However, from a theoretical spectroscopy point of view, few vibrational studies have tried to tackle protein-chromophore interactions,^50–53^ with most limiting their scope to the chromophore.^43,54,55^ In forming a consistent strategy in the vibrational analysis of light-sensitive domains, theoretical spectroscopy can aid in the interpretation of the experimental transient peaks coupled to electronic and structural changes that the protein undergoes through its photocycle.^56^ It can also help streamline the experimental effort by guiding the choice of isotope labelling and identifying possible marker bands in the spectrum.

**Scheme 1:**
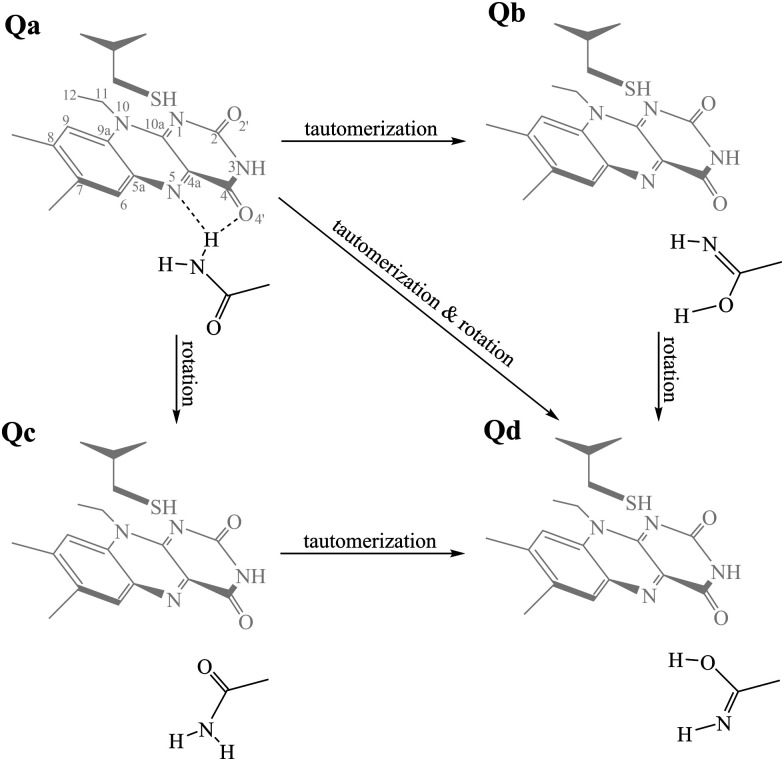
The side chain of the critical glutamine residue (**Qa**) of LOV domains can undergo tautomerisation (**Qb**), rotamerisation (**Qc**), or a combination of the above (**Qd**). As a reference, the FMN–cysteine moiety is shown in the dark state configuration (S_0_) greyed out.

The aim of this work is to address a missing element in the literature by providing a combined theoretical/experimental assignment of LOV domain time-resolved IR spectra.^7,32,33^ Cluster calculations were employed for this task, modelling a portion of the chromophore and surrounding protein residues known to be important mediators in signal propagation. Three different LOV proteins were chosen for the study, namely EL222, AsLOV2 and RsLOV, due to their variation in the vicinity of the flavin (Fig. 1a and b) and mechanism of action. The key intermediates along the photocycle were modelled and analysed vibrationally, producing difference spectra, which were then correlated with the experimental infrared spectra of the EL222 and AsLOV2 proteins from this work and the literature.^7,32,57^ Since both the photocycle and the spectroscopic analysis are presented here through the prism of the key glutamine residue, the possibility of imidic tautomerisation of its side chain was also considered for the LOV domains (Scheme 1). This was highlighted before in BLUF,^37,53,58–61^ a class of blue-light absorbing photoreceptors sharing the isoalloxazine ring. While with this computational setup the conformational changes of the protein evolution cannot be tracked, the scope is to probe the electronic, vibrational and structural changes from femtoseconds-to-microseconds, describing the flavoprotein photocycle from excitation right up to adduct formation *i.e.*, before signals emanating from large scale protein conformational changes start to dominate the observed spectral changes. Recent evidence suggests that the critical step initiating large scale structural effects is the flavin photoreduction to the neutral semiquinone radical and not the adduct state.^19,20^ However, spectroscopic evidence of the radical intermediate remains essentially elusive from an experimental point of view.^62–64^ Pertaining to the above, glutamine side chain rotational barriers were computed for both the triplet biradical and adduct steps of the LOV domains studied.

## 2. Methods

### 2.1 Cluster setup

The computational models were designed on the basis of the crystal structures of the LOV proteins EL222 (PDB entry 3P7N, chain B, UniprotKB Q2NB98),^21^ RsLOV (PDB entry 4HJ6, UniprotKB M1E1G1),^23^ and AsLOV2 (PDB entry 2V0U, UniprotKB O49003).^25^ For RsLOV and AsLOV2 the light state structures were also utilised (PDB entries 4HNB and 2V0W, respectively). Fig. 1a and b illustrate the limited variety present in the active site with regards to the relative position of the key conserved residues. AsLOV2 and RsLOV were chosen over other LOV domains due to the different residues present in the flavin binding pocket. The cluster models were constructed as follows:

#### EL222

The cluster considered (Fig. S1, left, ESI†) has a total of 135 atoms, containing the FMN isoalloxazine ring truncated at the second ribityl carbon C_12_. It includes two asparagine residues (Asn107 and Asn117) truncated at Cα and the full Cys75 and Gln138 residues capped with CH_3_CO– and –NHCH_3_. It also includes the Ala39 residue of the proximal β-sheet that stabilises the glutamine *via* hydrogen bonding also capped by CH_3_CO– and –NHCH_3_. The crystallographic water present in all the LOV domains in the proximity of the cysteine backbone was included. No crystal structure of the light state of EL222 exists, so light state structures were based on the dark state as outlined in Section 2.2.

#### AsLOV2

A cluster of 154 atoms was constructed, containing Asn414, Cys450 and Gln513 (capped) and Gln454, Asn482 and Asn492 (Cα-truncated). For the light state models, the equivalent crystal structure was utilised. FMN, truncated at C_12_, and the conserved crystallographic water were also included as per the EL222 cluster (see Fig. S1, middle, ESI†).

#### RsLOV

A cluster of 175 atoms was constructed, comprised of the residues Cys55 and Gln118-Ser117 (capped), Asn87, Asn97, Gln59 (Cα-truncated) and the Thr21-Leu20-Ala19 sequence capped at the two ends. For the light state models, the same atoms were included but fixed at the equivalent crystal structure positions. FMN was truncated at C_12_ and the conserved crystallographic water was included (see Fig. S1, right, ESI†).

### 2.2 Computational details

All calculations were performed with the Gaussian program (G16 Rev. B.01).^65^ The hybrid B3LYP^66,67^ and the cc-pvdz^68–70^ combination of DFT functional and basis set was used. For all calculations dispersion corrections were included,^71^ and the –CH_3_ method recommended by Siegbahn *et al.* was utilised to fix the Cα-carbons of residues to the crystallographic positions.^72^ A dielectric constant was applied (*ε* = 4) *via* the polarisable continuum model (PCM) for ground-state optimisations^73–75^ and through its implementation for excited states.^76^ Altering the dielectric in the 2–16 range did not alter the obtained spectra substantially – affecting only the intensities of backbone NH vibrations around ∼1550 cm^−1^. For the RsLOV calculations the solvent accessible surface (SAS) was employed,^77^ since the default tesserae creation resulted in a fragmented solvent surface during a few of the optimisations. For all IR curves a half-width at half-maximum value of 8 cm^−1^ (HWHM) was used to match the experimental curve shapes. All calculated IR spectra, including those on the S_1_ surface, were scaled according to the theory level by a factor of 0.97,^78^ since the aim was to produce difference spectra by combining ground and excited state signals (see Section 3.6). Excited state optimisations on the S_1_ state manifold were obtained *via* TDDFT, with the same combination of method and basis set as the ground state calculations. When degenerate double excitations were observed, they were both optimised (*e.g.***Qd** models of AsLOV2 and RsLOV). The triplet states of the LOV clusters were obtained *via* the unrestricted B3LYP method, and triplet biradicals were constructed by displacing the Cys75 hydrogen from –SH to the N_5_ atom of isoalloxazine. Adduct states were constructed by either using the existing lit state crystal structure as initial geometries (RsLOV and AsLOV2), or in the absence of this, by manually forming the S–C_4a_ bond based on the dark state structure (EL222). To obtain the glutamine rotational barriers of the **Qa** → **Qc** reaction, relaxed scans were performed on the T_1-H_ and 
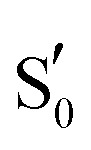
 manifolds of the three LOV domain clusters by altering the CCCN dihedral angle of the glutamine side chain with a step size of 10°. Stationary points on the EL222 and AsLOV2 clusters were confirmed by subsequent transition state optimisations and frequency analysis, which yielded a single imaginary frequency corresponding to the out-of-plane vibration of the dihedral angle. The RsLOV cluster proved too large to obtain a transition state, so the reported approximate barriers refer to the highest point in the relaxed scan optimisation.

Since deuteration is standard practice in IR spectroscopic experiments with proteins, the effect of immersion in a deuterated water medium was also taken into account by replacing exchangeable protons in the clusters by deuterium. For example, in EL222 these included the two protons of the crystallographic water and the C75 backbone proton H-bonded to it, the side chain C75 –SH proton and the two protons of the NH_2_ group of the Q138 side chain.

### 2.3 Experimental details

The EL222 protein was purified as previously described,^21^ buffer exchanged to MES 50 mM NaCl 100 mM imidazole 0.5 mM pD = 6.8 (D_2_O-based buffer) and concentrated to an optical density of ∼1 measured at 1650 cm^−1^ and 25 μm path-length (*i.e.* ∼3 mM protein concentration).

Time-resolved infrared spectra of EL222 (17-225) were obtained at time delays ranging from femtoseconds to sub-milliseconds and were performed on a setup described before.^28,79–81^ The pump wavelength was tuned to 475 nm while the probe beam covered the mid-IR range from 1511 to 1759 cm^−1^ with 4 cm^−1^ spectral resolution. 108 time points were acquired from −100 ns to 702 μs with background spectra measured at negative time delays. Transient absorption data were calculated as the difference between the probe spectrum with pump light and probe spectrum without pump light. To ensure EL222 recovery to the dark ground state, the sample was refreshed by scanning in a Lissajous pattern with a return time of 2 minutes.

Transient IR spectra were analysed using global kinetic modelling^82^ as implemented in the Glotaran software.^83,84^ A sequential decay scheme was applied to extract the evolution-associated difference spectra (EADS) and characteristic times from the raw data. The number of components was determined by singular valued decomposition analysis of the time traces. Because mono-exponential decays were fitted to the data, the derived time constants correspond to the lifetime of each component *i.e.* the time at which the population is reduced by 1/*e* times its initial value.

## 3. Results and discussion

To study the LOV domain photocycle, apart from the initial ground state S_0_, three distinct electronic states were considered (Fig. 2a). The excited singlet state S_1_, the equivalent triplet T_1_ and the formed adduct 
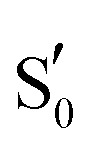
. Hot singlet states were disregarded at this point since the focus is on the time scales between ns-to-μs rather than fs-to-ps. Moreover, two intermediates can be placed on the triplet manifold, before (T_1_) and after H-transfer to N_5_ (T_1-H_), while in the adduct, the ground state 
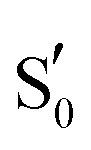
 is the most valid electronic state since formation on that surface proceeds barrierless.^13^ With respect to the conserved glutamine (Scheme 1), at each one of the intermediates described above, it can have the side chain amide group either towards the FMN or flipped, and can also be in amidic or imidic tautomeric form as was suggested for the BLUF proteins.^37^

By combining Scheme 1 and Fig. 2a, **Qa–Qd** models were created for each of the intermediates totalling 20 structures for each LOV domain. For example, the EL222 S_1_**Qd** model involves a structure with the conserved glutamine in the imidic tautomeric form, rotated (flipped) by ∼180° with respect to **Qb** (CCCN dihedral value, Table S1, ESI†), and lying on the excited singlet potential energy surface. Thus, information about possible glutamine tautomerism and rotation is provided at each step of the photocycle. The ground state or S_0_ will be also referred throughout the manuscript as the dark state. The lit states include all subsequent states in the photocycle, the excited singlet (S_1_), the triplet before (T_1_), and after H-transfer (T_1-H_), and the adduct 
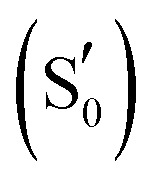
. When referring to the experimental evolution associated difference spectra (EADS) in Sections 3.2–3.6, the experimental nomenclature is retained, namely 1FMN*, 3FMN* and A390 which are assigned to the excited singlet, triplet and adduct states, respectively.

### 3.1 Energetics along the LOV Photocycle

In the ground state, the **Qa** model is predicted more stable by ∼4–11 kcal mol^−1^, depending on the LOV domain, from the second most stable **Qc** model. Additionally, the calculations reveal that the imidic tautomers are higher in energy by more than 10 kcal mol^−1^ over the amidic across all LOV intermediates (see Fig. S8–S10, ESI†). As is known from calculations on the chromophore itself,^35^ and YtvA,^13^ T_1_ tends to be more stable by ∼10 kcal mol^−1^ than the equivalent S_1_ state. In the protein cluster environment this also holds true, except for RsLOV where the stability of the triplets falls to 6 kcal mol^−1^. Validating the above, the computed energy of the AsLOV2 **Qa** T_1_ model relative to S_0_ (44.7 kcal mol^−1^, Fig. S9, ESI†) is found close to the experimentally determined value of 47.5 kcal mol^−1^.^85^ One exception is found involving the **Qc** intermediate of the EL222 cluster, where the equilibrated S_1_ state is predicted more stable than the triplet (for this issue see the discussion at the end of this section). The subsequent H-transfer in the triplet manifold incurs further stabilisation by over 17 kcal mol^−1^. Finally, the adduct computed energies with respect to the ground state span 7–19 and 27–37 kcal mol^−1^ for the amide and imidic glutamine tautomers, respectively. The optimised geometries at each state are depicted in Fig. 2b–d and Fig. S2–S7 (ESI†), their relative energies along the reaction path in Fig. S8–S10 (ESI†), and key geometric distances and dihedral angles are collected in Tables S1–S3 (ESI†).

More analytically, for AsLOV2 the relative energies of the computed intermediates are included in Fig. S9 (ESI†) and geometries along a minimum energy path dictated by their relative stabilisation are shown in Fig. 2c. The rotation of the Q513 side chain is predicted exothermic at the last step of the cycle where the **Qc** model is more stable than **Qa** by 3.6 kcal mol^−1^. Other values along the photocycle range from 11.6 kcal mol^−1^ at the S_1_ state to as close as 1.2 kcal mol^−1^ at the T_1-H_ state in favour of the initial glutamine configuration. Apart from the H-bond formed between the Q513 side chain carbonyl and the N_5_–H of FMN, additional stabilisation in the adduct is provided by the flexible N414 which H-bonds its side chain carbonyl group to NH_2_ of the rotated Q513 residue (Fig. 2c, far right). That configuration has been reported previously by analysis of the trajectories of MD simulations.^7,48^ The rest of the geometries of the amidic variants are included in the ESI† in Fig. S4 and S5 displaying no interaction of the N414 side chain except with the backbone of Q513. With respect to the Q513 imidic isomeric state, energies range from 22 kcal mol^−1^ in S_1_ to 16 kcal mol^−1^ in the adduct, relative to the amide counterparts. The **Qd** model of AsLOV2 possesses two degenerate ππ* excitations that were both optimised and yielded different S_1_ state geometries. Their relative energies are shown in green and light green in Fig. S9 (ESI†).

For EL222, the relative energies of the computed intermediates are included in Fig. S8 (ESI†), and the optimised geometries with glutamine in amidic form are shown in Fig. 2b and Fig. S2, S3 (ESI†). With respect to the glutamine, a possible rotation is indicated at the S_1_ state – see discussion at the end of this section, but this is not maintained in the subsequent step. The imidic isomers are less favoured energetically by over 14 kcal mol^−1^ along the whole the reaction coordinate. At the H-transfer and adduct steps, the alanine (A39) of the proximal β-sheet stabilises the glutamine Q138 side chain rotation similarly to the asparagine of AsLOV2 (Fig. 2b). In that case though, the A39 backbone carbonyl site is shared between the side chain and backbone of Q138 since no flexible side chain with H-bonding capability is present in the vicinity of the glutamine. Accordingly, the stabilisation that the glutamine rotation affords to both the H-transfer and adduct states is computed at ∼4.3 kcal mol^−1^.

For the RsLOV cluster, the glutamine flip is not predicted endothermic at any step, yet the energetic gap between **Qa** and **Qc** becomes progressively smaller along the reaction coordinate, from 14.6 kcal mol^−1^ in the excited singlet state to as low as 2.5 kcal mol^−1^ in the adduct (Fig. S10, ESI†). This can be rationalised by inspecting their geometries along the reaction path shown in Fig. 2d and Fig. S6, S7 (ESI†). In the **Qa** models, the primary amide of Q118 in the initial configuration H-bonds with the side chain hydroxy group of T21 (Fig. 2d), whereas in rotated position (**Qc**), it H-bonds to the backbone carbonyl of L20 (Fig. S7, ESI†). Notable also is the absence of a N_5_–H⋯O=C H-bond after glutamine rotation; in the **Qc** adduct the glutamine side chain carbonyl prefers to interact with the hydroxy of T21. In analogous fashion, in **Qa** the H-bond of the Q118 primary amide with O_4′_ is exchanged after H-transfer with the T21 hydroxy oxygen. The imidic **Qd** models are over 17 kcal mol^−1^ higher in energy at each step, and the **Qb** isomers are found the least stable (see green and blue lines in Fig. S10, ESI†). The lack of significant changes in the glutamine configuration of RsLOV raises intriguing questions about the signal transduction mechanism in this protein.

To refine the above issues, reaction free energies and rotational barriers were computed for the last two steps of the studied photocycle and are collected in Table 1 along with their respective dihedral angles. Rotational barriers have been reported previously for the LOV protein Vivid. The authors reported a 1.7 kcal mol^−1^ free energy barrier for glutamine rotation and a stabilisation of the adduct by 6 kcal mol^−1^ at the light state.^6^ AsLOV2 exhibits a very facile rotation at the adduct step with a free energy barrier as low as 0.4 kcal mol^−1^ while the barrier is predicted at 4.8 kcal mol^−1^ in the biradical intermediate. Moreover, the stabilisation of the flipped conformation in the biradical is predicted small which makes the reverse reaction possible with a barrier of +1.7 kcal mol^−1^. EL222 barriers are computed at 6.5 and 4.6 kcal mol^−1^ for the T_1-H_ and 
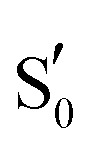
 states respectively. The **Qa** → **Qc** reaction is predicted exergonic for both LOV domains at the adduct step with a stabilisation of more than 4 kcal mol^−1^ favouring the flipped glutamine conformation (**Qc**). Finally, in RsLOV, barriers are predicted over 9 kcal mol^−1^ for both steps (approximate Δ*E*_a_ values, see Section 2.2). The reaction is predicted endergonic at both steps by 6.4 and 0.7 kcal mol^−1^ for T_1-H_ and 
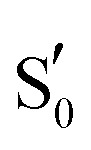
, respectively. To place these values in perspective, the H-transfer barrier has been estimated at 5.2 kcal mol^−1^ (Δ*E*_a_) for the YtvA LOV domain while the bond formation is barrierless,^13^ so computed values >5 kcal mol^−1^ could place the glutamine rotation as the rate limiting step of the photocycle – if we assume that glutamine rotation does not affect the ISC between states.

**Table 1:** Relative free energies Δ*G*, rotation barriers Δ*G*^‡^ and dihedral angles of the reactants, transition states and products of the **Qa** → **Qc** glutamine flip reaction for the T_1-H_ and 
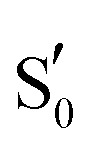
 states of the three LOV domains

	EL222		AsLOV2		RsLOV	
	Δ*G*/Δ*G*^‡^ (kcal mol^−1^)	CCCN dihedral (°)	Δ*G*/Δ*G*^‡^ (kcal mol^−1^)	CCCN dihedral (°)	Δ*G*/Δ*E*_a_ (kcal mol^−1^)	CCCN dihedral (°)
	**Triplet biradical (T_1-H_)**					
**Qa**	0.0	178.4	0.0	178.7	0.0	−136.7
**TS**	+6.5	102.3	+4.8	99.4	+10.3^a^	94.5
**Qc**	−1.2	16.4	+3.1	63.8	+6.4	70.4
	**Adduct** 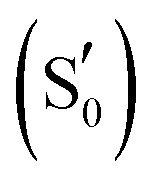					
**Qa**	0.0	165.3	0.0	148.0	0.0	−133.8
**TS**	+4.6	113.6	+0.4	55.4	+9.3^a^	89.6
**Qc**	−4.1	23.3	−4.9	23.1	+0.7	59.8

a Approximate Δ*E*_a_ barrier.

To summarise the results between the LOV domains, owed to the different topology and β-sheet residues, the calculations show a divergence in the photocycles of each domain. The rotation of the glutamine in AsLOV2 and EL222 occurs easier at the adduct state, being much more facile for the former, while the rotation is disfavoured in RsLOV. For the former two, the flipped configuration is predicted much more stable in the adduct rather than the biradical.

**Fig. 3:**
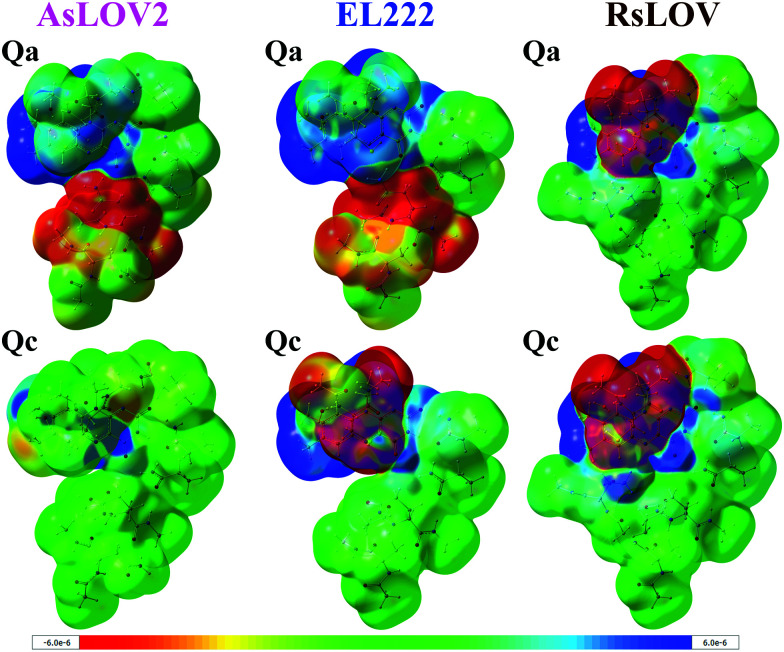
Electron density difference plots of the S_1_ state of AsLOV2 **Qa** (top left) and **Qc** (bottom left), EL222 **Qa** (top middle) and **Qc** (bottom middle) and RsLOV **Qa** (top right) and **Qc** (bottom right). Blue areas indicate positive values *i.e.* where excited state density is larger than the ground state density, and red indicate larger ground state density. Surfaces were plotted with a density isosurface value of 0.0001 and the colour range for the plots is given in the bottom of the figure.

As noted above, the **Qc** model of EL222 in the S_1_ state exhibits lower energy than the triplet. By inspecting their optimised geometry, it can be seen that the former possesses a transition state (TS)-like geometry with respect to the cysteine –SH moiety and FMN, therefore they are not equivalent structures. This is illustrated by the N_5_⋯H–S distance which in S_1_**Qc** is as short as 1.674 Å. For comparison with the other S_1_ models, typical N_5_⋯H–S distances span over 2.8 Å in EL222 (Table S1, ESI†), 5.3 Å in AsLOV2 (Table S2, ESI†) and 4.4 Å in RsLOV (Table S3, ESI†). Hence, the EL222 **Qc** S_1_ structure represents an intermediate further along the excited singlet potential energy surface (PES) than their counterparts (or on a different PES), and closer to the H-transfer step. This explains for the discrepancy in the energy relative to T_1_ and the other S_1_ states. A single point energy calculation on the T_1_ PES employing the EL222 **Qc** S_1_ geometry reveals a lower energy of the triplet by 22.8 kcal mol^−1^. Thus, for a given geometry the triplet will be more stable than the excited singlet, as was demonstrated by QM(MS-CASPT2)/MM calculations in the YtvA LOV domain.^13^ Further information is provided by the density difference plots shown in Fig. 3 for the **Qa** and **Qc** models of RsLOV, AsLOV2 and EL222 (the latter are repeated from Fig. S11, left, ESI†). Density difference surfaces are constructed by subtracting the ground state density from the first excited state density and mapping them on the total density surface of the molecule. Blue regions indicate positive values, where density is larger in the excited state than the ground state, while red regions indicate the opposite. AsLOV2 **Qc** (Fig. 3, bottom left) showcases the typical pattern of a ππ* local excitation similar to free FMN. The electron density is flowing from one of the methyl groups and C_2_=O_2′_ (red area) to the N_5_ and C_4a_ atoms of FMN (blue area). For the **Qa** models of EL222 and AsLOV2 in particular, glutamine is included in the area of decreasing density. Finally, another pattern is shown in the TS-like structure obtained for EL222 **Qc** (Fig. 3, bottom middle) and the **Qa** and **Qc** plots of RsLOV. These exhibit a decrease in electron density in the cysteine residue with commensurate increase in the isoalloxazine ring. The density plots presented here (see also the differential plots in Fig. S12, ESI†) exhibit a similarity with previously reported S_1_ charge-transfer (CT) states in BLUF.^60^ However, inspection of the charge distribution in the isoalloxazine moiety of the S_1_ states obtained here, reveals only one instance of a true CT state – the **Qc** state of EL222 (−0.7 charge) – while in the rest no charge transfer takes place between the aforementioned residues and the isoalloxazine moiety (Table S4, ESI†). Further investigation is required to clarify if such states take part in the photocycle, as in BLUF,^60^ or are an artifact of the functional.

The spin difference plots (spinA–spinB) for the T_1_ and T_1-H_ states are displayed in Fig. S11 (ESI†) (middle and right sections, respectively). The unpaired electron in the T_1_ state is located solely in the isoalloxazine ring (Fig. S11, middle, ESI†). Once the H-transfer is complete, excess spin encompasses also the cysteine residue (Fig. S11, right, side views, ESI†), typical of the biradical configuration reported elsewhere for YtvA,^13,14^ and phototropin-LOV1,^86,87^ which is rapidly followed by the formation of the C–S bond. Inspection of the isoalloxazine charge distribution between the T_1_ and T_1-H_ states confirms the neutral biradical character of the structures described here (Table S4, ESI†).

The spectroscopic part of the study follows in the subsequent Sections 3.2–3.6. Since the cluster models described limit the area of study to the vicinity of the chromophore, no large-scale conformational changes can be modelled with the setup detailed above. Additionally, since FMN was truncated at C_12_, ribityl-phosphate vibrations are missing from the computed spectra. It was reported by our group, *via* Raman calculations in free FMN, that vibrations of the ribityl-phosphate moiety can be expected up to 1450 cm^−1^, usually coupled to isoalloxazine modes.^35^ However, the ribityl chain retains an unfolded conformation in the protein environment, unlike in solution. A possible interaction that might register in the spectra is between ribityl hydroxy groups and the side chain of Q454 and Q59 in AsLOV2 and RsLOV, respectively which is missing from the calculations.

### 3.2 Experimental EL222 time-resolved IR spectra

**Fig. 4:**
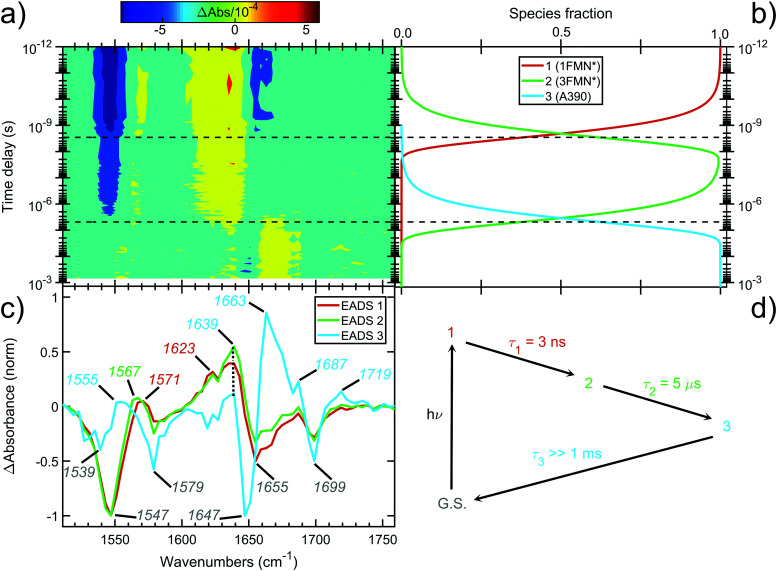
Time-resolved picosecond-to-sub-millisecond infrared spectra of EL222 in deuterated buffer. (a) Contour plot of the mid-IR difference spectra at different time delays (in log-scale) after 475 nm photoexcitation. (b) Amplitudes (species fraction) derived from global kinetic analysis. (c) Normalised evolution-associated difference spectra (EADS) derived from global kinetic analysis. The principal bands discussed in the text are highlighted. (d) Three-component sequential decay scheme employed for the global kinetic analysis. Components 1, 2 and 3 are assumed to correspond to 1FMN*, 3FMN* and A390 species, respectively. An additional component was used to fit the coherent artefact in the sub-picosecond time scale (not plotted for the sake of clarity). The fitted lifetimes (*τ*) are indicated. G.S. denotes the ground state.

The contour plot of the transient IR difference spectra obtained at different time delays (ps-to-sub-ms) after photoexcitation is presented in Fig. 4a. Four sequential components (0th, 1st, 2nd, 3rd) were fitted to the obtained data for EL222. The 0th component (sub-ps time scale) was used to fit the coherent artifact and will not be further discussed. The 3rd component does not decay within the experimental time window so *τ*_3_ was fixed to 26 seconds, which reflects the recovery time to the dark state.^21^ The amplitudes and spectra (EADS) derived from the global analysis are shown in Fig. 4b and c respectively. According to the proposed model, the 1st EADS evolves into the 2nd EADS with a time constant *τ*_1_ = 3 ns, which in turn evolves into the 3rd EADS with time constant *τ*_2_ = 5 μs (Fig. 4d). The 1st, 2nd and 3rd components were assumed to correspond to the singlet (1FMN*), triplet (3FMN*) and adduct (A390) states, respectively. The three EADS will be discussed in the subsequent sections involving the dark state computed IR spectra, and in Section 3.6 where the experimental difference spectra of EL222 are presented together with the computed EL222 analogues. In comparison, AsLOV2 displayed time constants of *τ*_1_ = 2.4 ns and *τ*_2_ = 9.5 μs for the first two states while adduct decay exceeded the experimental window of hundreds of μs.^32^ To our knowledge no time-resolved spectroscopic experiment exists in the literature for RsLOV.

### 3.3 EL222 dark and lit state IR spectra

The computed S_0_ ground state spectra will be subtracted from the spectra of all other species to simulate the experimental curves (see Section 3.6), therefore it is crucial to establish a reasonable agreement with the experimental ground state spectra. In principle, negative peaks (bleach) in the light-minus-dark difference spectrum are associated with the (diminishing) ground state population.

For EL222, the experimental difference spectrum exhibits a strong bleach signal at 1547 cm^−1^ (Fig. 4c). Other ground state features include the peaks at 1579, 1655 and 1699 cm^−1^. The wavenumbers remain constant for the 1FMN* and 3FMN* EADS and are in reasonable agreement with time-resolved infrared spectra reported by Meech, Tonge and co-workers.^32^ In the A390 EADS, pronounced changes are recorded in the negative portion of the spectra. The 1547 cm^−1^ peak shifts to 1539 cm^−1^, the peak at 1655 cm^−1^ is obscured with a simultaneous appearance of a strong bleach at 1647 cm^−1^, while peaks at 1579 and 1699 cm^−1^ remain unaffected (Fig. 4c). These changes signify possible new states due to altered secondary structure and cannot be described by any of the computational models analysed here – therefore no correlation is attempted for the below baseline peaks of the A390 EADS.

The correlation of the 1FMN* negative peaks with the EL222 **Qa** cluster is presented in the top left rows of Table 3 together with mean absolute deviation (MAD) values for the correlation. The wavenumbers reported are taken from the deuterated spectra. As mentioned in Section 3.1, the **Qa** model will be prevalent in the ground state (S_0_); nonetheless, for the sake of completeness, the rest of the models **Qb–Qd** are also included in the top rows of the correlation Tables S9 and S10 (ESI†). The non-deuterated ground state spectra of **Qa** are included in the ESI,† plotted together with the models **Qb–Qd** in Fig. S13 (ESI†). For **Qa**, decent agreement is achieved with experiment, which is manifest by the MAD value of 11.8 cm^−1^.

All calculated excited state spectra of the EL222 cluster are included in Fig. S13–S16 (ESI†) (non-deuterated). The usual pattern for all LOV clusters in the fingerprint region is observed: The various intense C=O stretch peaks are concentrated at ∼1600–1700 cm^−1^. The asparagine and glutamine side chain C=O stretches are usually coupled to the corresponding NH_2_ modes. Side chain NH_2_ modes either uncoupled or coupled to FMN breathing modes, appear lower in the spectrum in the range 1550–1400 cm^−1^. Finally, methyl and methylene peaks are located at ∼1300 cm^−1^. The evolution of selected modes from the ground state up to the adduct is tracked in Table 2 for **Qa–Qc** and Table S7 (ESI†) for **Qb–Qd**, to make for a more facile analysis of the numerous vibrations.

**Table 2:** Evolution of selected **Qa**, **Qc** EL222 and AsLOV2 IR peaks along the reaction coordinate. Isotopic shifts from the non-deuterated spectra are given with red numbers. Values in cm^−1^

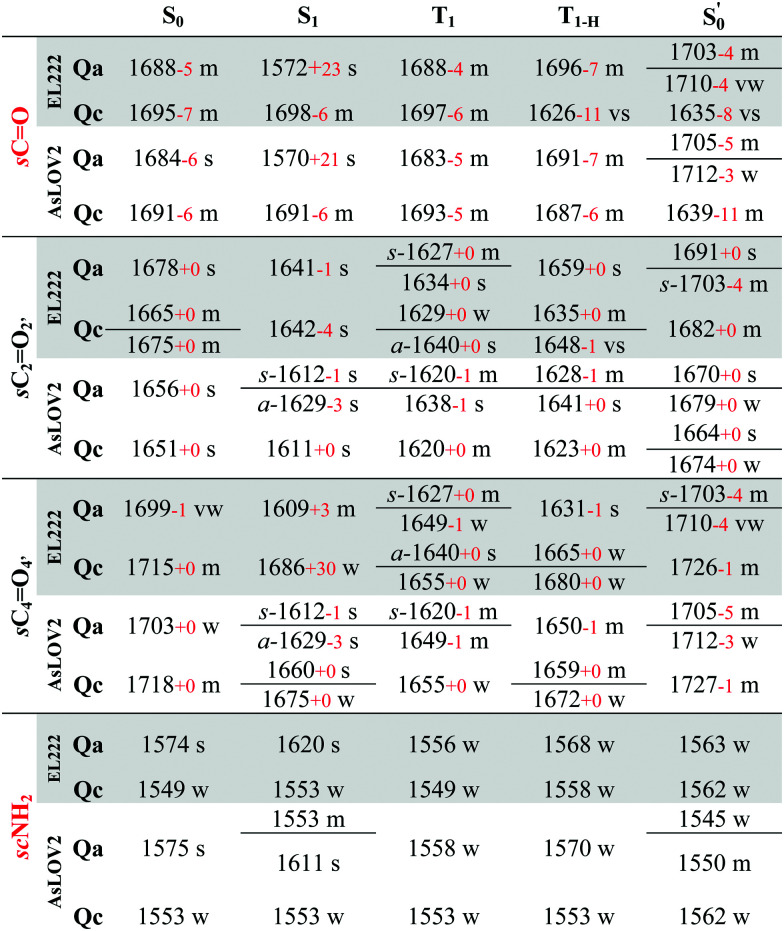

**Table 3:** EL222 and AsLOV2 IR spectra assignments for the **Qa** and **Qc** models. Normal modes are coloured according to residue; FMN black, Q138/Q513 red, C75/C450 orange, N107/N482 green, N117/N492 violet, Q454 grey, A39/N414 blue and other backbone modes, light blue. Isotopic shifts from the non-deuterated spectra are given with red numbers. Mean absolute deviations are included for each theoretical/experimental correlation, for both the deuterated (black) and non-deuterated spectra (red). When multiple normal modes are reported for a vibration, assignments are presented in order of decreasing displacement. All values in cm^−1^

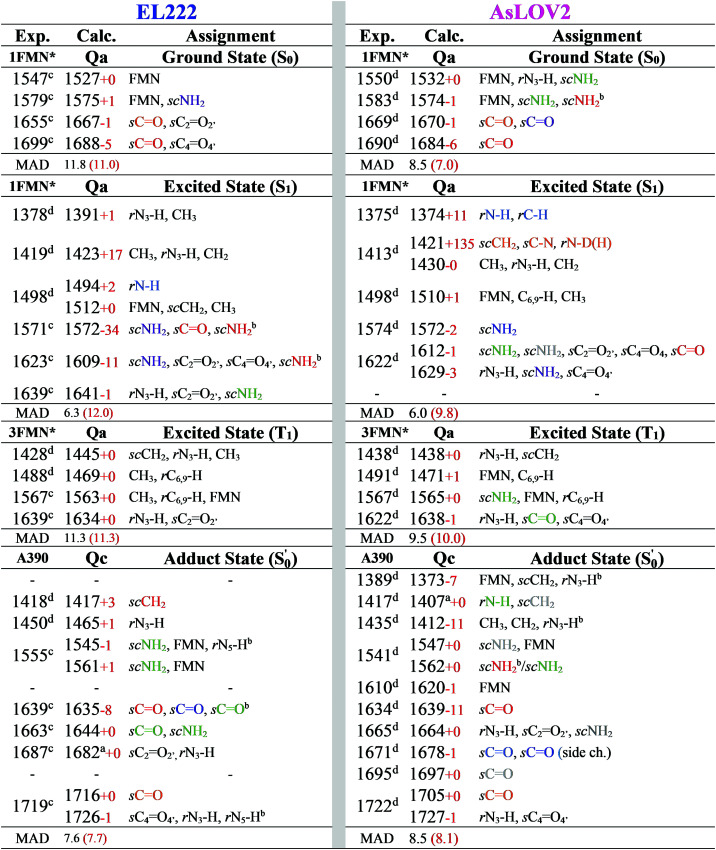

a Relatively intense ES peak in proximity to the experimental, offset by the GS signal.

b Normal mode only in non-deuterated spectrum.

c This study.

d Taken from ref. 32.

Theoretically, two modes can report directly on the glutamine side chain conformation. The first is the carbonyl stretch, usually coupled to NH_2_ scissoring, and the second is the NH_2_ scissoring mode itself, although this mode will be suppressed from the fingerprint region under deuteration. The glutamine carbonyl stretch is predicted at a rather low wavenumber for **Qa** S_1_ at 1572 cm^−1^ and is coupled to NH_2_ scissoring in the non-deuterated spectrum (1549 cm^−1^, Fig. S14, ESI†). The amide mode itself is predicted in **Qa** at 1620 cm^−1^ coupled to FMN carbonyl stretches – with no equivalent in the 1350–1750 cm^−1^ region in the deuterated spectrum. Proceeding to the triplet manifold, the T_1_ and the biradical T_1-H_ spectra are plotted together in Fig. S15 (ESI†). The glutamine carbonyl stretches of **Qa** and **Qc** are identical to the ground state, with the corresponding amide modes predicted with weak intensity in the non-deuterated spectra. Finally, in the adduct state of **Qa**, the glutamine carbonyl stretch is found at the highest wavenumber observed, with two vibrations of medium and weak intensity (1703, 1710 cm^−1^, respectively). In **Qc** it is coupled to the backbone C=O stretch of the adjacent β-sheet alanine (A39), with the strongest intensity in the spectrum, making it a possible marker for the glutamine flip during adduct formation.

With regards to the FMN carbonyl stretches (C_2_=O_2′_/C_4_=O_4′_), their intensity depends on their coupling with modes of other functional groups, usually the N_3_–H bend and the NH_2_ scissoring and C=O stretching modes of the proximal asparagines. The two carbonyl stretches can also be coupled together in an asymmetric or symmetric fashion (see Table 2, tagged with s- for symmetric and a- for asymmetric). In principle, asymmetric stretches display increased IR activity due to the change in net dipole and inversely, the symmetric stretches are IR-inactive. In the EL222 cluster, the asymmetric vibrations have strong-to-very-strong computed intensities such as in the T_1_ state of **Qc**, while the symmetric ones tend to be weaker (*e.g.***Qa** in T_1_ and 
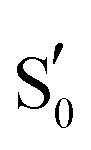
, medium intensity). In the sequence from T_1_ → T_1-H_ → 
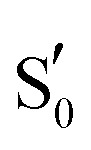
, a progressive blue shift is evident in the FMN carbonyl stretches. The blueshift can be connected to the increasing perturbation of the isoalloxazine ring due to the H-transfer in T_1-H_ and the subsequent covalent bond formation in the adduct. By inspecting the bond lengths, a lengthening-shortening pattern is observed for both C_2_=O_2′_ and C_4_=O_4′_ (Table S1, ESI†), so this effect cannot be rationalised solely with respect to the C=O bond length but in combination with the other normal modes coupled to the vibration. For example, the N_3_–H⋯O=C_N107_ hydrogen bond progressively weakens from the triplet state to the adduct (**Qa**: 1.823 Å → 1.830 Å → 1.856 Å, Table S1, ESI†) which affects the N_3_–H bending coupled to the carbonyl modes. When comparing S_1_ with T_1_, the computed peaks give a more mixed picture, *e.g.* the C_4_=O_4′_ stretch is predicted to move to the red from S_1_ → T_1_, with the exception of **Qa**.

**Fig. 5:**
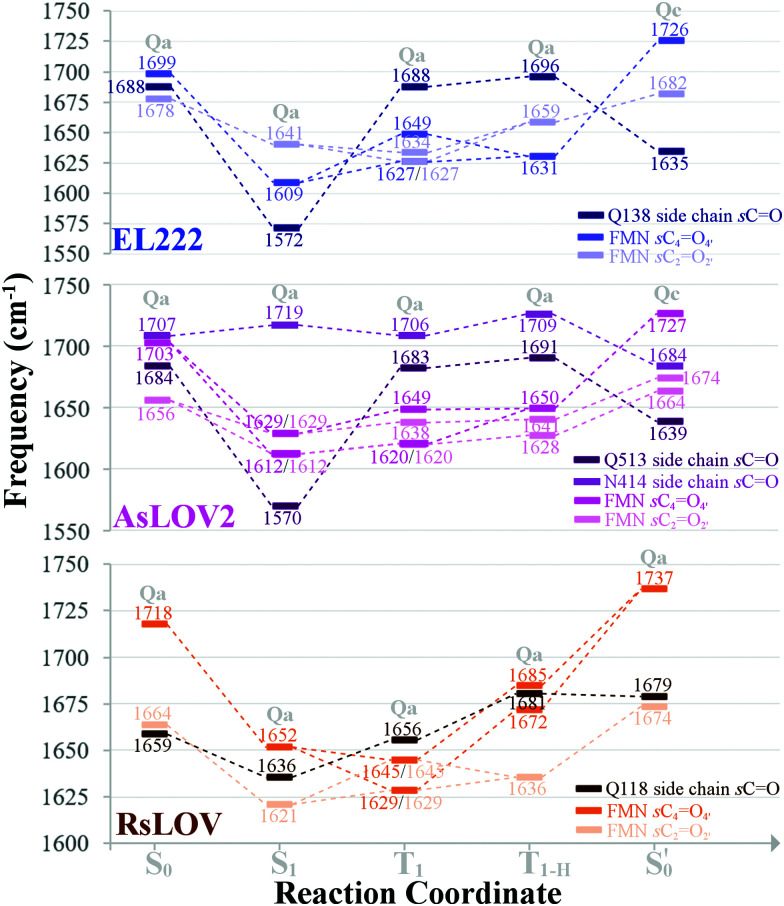
Evolution of selected carbonyl stretches of the EL222, AsLOV2 and RsLOV clusters along the reaction coordinate.

The aforementioned vibrations are tracked along a tentative minimum energy path, shown for EL222 in the top portion of Fig. 5. For EL222 this involves **Qa** from S_0_ up to the T_1-H_ state and the flipped **Qc** model in the adduct – although this scheme is not based on an extensive description of the PESs of the different states. With the exception of the S_1_ state, little variation is shown in the glutamine carbonyl peaks progressing along the photocycle from the ground state up to T_1-H_. Interestingly, in the spectra of the S_1_ state, a large redshift is recorded (−116 cm^−1^) for the glutamine sidechain carbonyl stretch of EL222. This can be explained by the predicted weakening of the C=O bond in the excited singlet of EL222 – with respect to S_0_ – by 0.03 Å. In T_1_ the bond is predicted equally strong to S_0_ and the vibration follows suit. In the adduct formation step, due to the flip of the glutamine, EL222 displays a characteristic red shift by 53 cm^−1^ of the glutamine C=O vibration with respect to T_1_. On the other hand, the FMN C_2_=O_2′_ and C_4_=O_4′_ stretches exhibit large blue shifts, with respect to T_1_, upon glutamine rotation by 48 cm^−1^ and 77 cm^−1^, respectively.

A unique feature in the non-deuterated S_1_ spectrum of **Qc**, due to the “TS-like” structure obtained (S–H⋯N_5_ distance = 1.674 Å), is the Cys75 S–H stretch predicted at a rather low wavenumber with strong intensity (1797 cm^−1^, not shown in the figures). It should be stressed here that S_1_**Qc** is a CT state, which might affect the computed spectrum (see end of Section 3.1). For comparison, in T_1_ where the contact length is much longer (2.897 Å) the same vibration is computed at 2603 cm^−1^ with weak intensity which is a typical value for the S–H stretch.^39^ For **Qa** the equivalent S_1_ and T_1_ values are 2600 cm^−1^ and 2601 cm^−1^ with medium and weak intensities, respectively. All S–H modes will be redshifted by ∼800 cm^−1^ upon deuteration.

### 3.4 AsLOV2: dark and lit state IR spectra

The correlation of the 1FMN* negative peaks with the AsLOV2 **Qa** S_0_ spectrum is presented in the top right portion of Table 3 together with mean absolute deviation (MAD) values. The rest of the models **Qb–Qd** are included in the top rows of the correlation Tables S9 and S10 (ESI†). The non-deuterated ground state spectra of **Qa** are included in the ESI,† plotted together with the models **Qb–Qd** in Fig. S17 (ESI†).

The strongest experimental ground state signal in AsLOV2 is found at 1550 cm^−1^. Other features include peaks at 1583, 1669 and 1690 cm^−1^ for both the 1FMN* and 3FMN* EADS.^28,32,54,88^ The experimental 1550 and 1583 cm^−1^ peaks of AsLOV2 have been assigned before to FMN breathing modes,^33^ based on calculations on the chromophore,^55^ which agree partially with the assignment in Table 3. Meech and co-workers assigned the bleach at 1690 cm^−1^ to Q513 in AsLOV2,^33^ which conforms with the computational assignment here. This vibration is coupled to C_4_=O_4′_ in EL222 while it is only due to the glutamine in AsLOV2. Moreover, the calculations predict the slight shift to the red of the 1547 cm^−1^ (1527 cm^−1^) bleach in EL222, compared to the 1550 cm^−1^ (1532 cm^−1^) bleach in AsLOV2 – computed peaks in parentheses. In the AsLOV2 adduct, loss of intensity and a small blueshift is evidenced at 1553 cm^−1^. A new intense bleach signal appears at 1625 cm^−1^, the 1669 cm^−1^ signal disappears, and the one at 1690 cm^−1^ remains constant.^32^ As mentioned in Section 3.3, no correlation will be attempted with the negative portion of the A390 EADS. Overall, the MAD values for the deuterated **Qa** models of EL222 (11.8 cm^−1^), and more so for AsLOV2 (8.5 cm^−1^), indicate quite a good agreement with the experimental ground state peaks. This shows that the MAD values can be relied upon for the more challenging to assign excited state spectra that will be analysed in Section 3.6.

All calculated excited spectra of the AsLOV2 cluster are collected in Fig. S17–S19 (ESI†) (non-deuterated spectra), and the evolution of selected bands along the photocycle is recorded in Table 2 and Table S7 (ESI†) for **Qa–Qc** and **Qb–Qd**, respectively. In the S_1_ state of **Qa**, as with EL222, the glutamine side chain C=O stretch is predicted at 1570 cm^−1^ due to the lengthening of the C=O bond by 0.03 Å. In the T_1_ and T_1-H_**Qa** states, the same modes, uncoupled from NH_2_, are positioned at 1683 and 1691 cm^−1^, respectively. For **Qc**, the corresponding frequencies are 1693 and 1687 cm^−1^ with peaks of medium intensity, showing little variation from the **Qc** singlet ground state spectrum. Finally, in the **Qc**
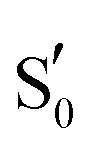
 model, the glutamine carbonyl stretch displays a similar pattern as in EL222 with a downshifted peak at 1639 cm^−1^. With regards to the FMN carbonyl stretches (C_2_=O_2′_/C_4_=O_4′_) a symmetric stretch is present in the S_1_**Qa** spectrum (s-1612 cm^−1^) which is maintained in the T_1_ state. The blueshift of the peaks from triplet to adduct state complies with the EL222 findings; when comparing the shift of the C_2_=O_2′_/C_4_=O_4′_ peaks going from the excited singlet to the triplet, most of the vibrations shift to the blue.

In Fig. 5 (middle part), the selected peaks of AsLOV2 are followed along the minimum energy path – which is identical to EL222. Apart from the aforementioned bands, the N414 side chain carbonyl stretch is also tracked. Upon glutamine rotation in the adduct step, both the Q513 and N414 C=O stretches redshift, with respect to T_1_, by 44 cm^−1^ and 22 cm^−1^, respectively. Similarly, the FMN carbonyl stretches blueshift by 78 cm^−1^ (C_4_=O_4′_) and 36 cm^−1^ (C_2_=O_2′_) on adduct formation coupled to the glutamine rotation.

### 3.5 RsLOV: lit state IR spectra

This is the larger cluster in the study, including 14 side chain and backbone carbonyl bonds, which makes for a more congested spectrum in the 1600–1750 cm^−1^ region. The non-deuterated spectra are included in Fig. S20–S22 (ESI†) and the evolution of selected peaks is tracked in Table S8 in the ESI.† Upon glutamine rotation – which does not result in stabilisation at any intermediate (Fig. S10, ESI†) – a blue shift of the Q118 side chain C=O peaks is expected, except in the adduct state. Overall, the trend of FMN carbonyl modes shifting to the blue as the photocycle progresses is maintained, as with the other LOV domains above. In Fig. 5 (bottom part), the minimum energy path entails no glutamine flip, so the vibrations of the **Qa** model are followed along the whole reaction coordinate. The glutamine carbonyl stretch displays some sensitivity to the Q118–T21 side chain interactions with shifts of ±22 cm^−1^ along the reaction coordinate. For the FMN C=O stretches, a large blue shift is evidenced on adduct formation, which for C_4_=O_4′_ reaches 92 cm^−1^.

### 3.6 IR difference spectra

In order to translate the above observations to a better understanding of the experimental findings, difference spectra were devised combining the excited and ground state computed spectra. For the correlation with the published time-resolved infrared spectra,^32^ and those obtained in this study, the following considerations should be taken into account. First, the experimental difference spectra have a positive (transient) portion due to the new population at each time delay, and a negative (bleach) signal due to the depopulation of the initial state. In principle, one could safely assume that the initial state corresponds to a unique LOV dark state with FMN in the ground state. In addition, cleavage of a bond in the excited state, would appear as a negative peak with corresponding frequency (*e.g.* the cysteine S–H(D) mode in the H-transfer/adduct steps). By subtracting the two signals (intermediate minus initial), it is assured that only normal modes that pertain to changes in the system will be visible, since the rest cancel each other *via* the subtraction. Second, the experimental spectra were analysed in terms of the evolution-associated difference spectra (EADS) which, strictly speaking, do not correspond to actual species. These are directly compared to calculated spectra emanating from pure, equilibrated electronic states. Third, EADS were derived for the ^1^FMN*, ^3^FMN*, and A390 states (see Section 3.2) which are assumed to correspond to the computed S_1_, T_1_, and 
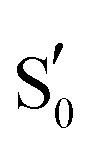
 states, respectively. No transient infrared spectrum is available for the T_1-H_ state (one possible exception will be discussed below) so together with the T_1_, it is compared to the available ^3^FMN* EADS.

To mimic the experimental spectra that contain information both on the initial ground state and the transient lit populations, the subtraction procedure detailed above was followed with the computational spectra – a methodology that has been employed previously in lumiflavin,^64^ riboflavin^89^ and BLUF cluster calculations.^37^ The unaltered spectra were discussed in Sections 3.3–3.5 and are also included in the ESI† (non-deuterated, Fig. S13–S22). For each of these, an experiment-like difference spectrum can be devised by subtracting the ground S_0_ state spectrum (negative values) from each of the spectra of the excited species: S_1_, T_1_, T_1-H_ and 
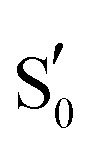
 (positive values). At each of the four distinct intermediate species, the state of the glutamine residue can be probed according to the four glutamine configurations **Qa–Qd**. Since experimental samples are routinely immersed in D_2_O, the deuterated spectra were employed to construct the computational difference spectra and positive and negative intensities were normalised before subtraction. The global ground state **Qa** S_0_ spectrum was employed as the reference state for subtraction, which assumes glutamine Q138/Q513 in its initial position without tautomerisation as the dark state. For example, the difference spectrum of the **Qb** in the triplet state is indicated as [**Qb**]T_1_–S_0_[**Qa**], or for simplicity **Qb** T_1_.

**Fig. 6:**
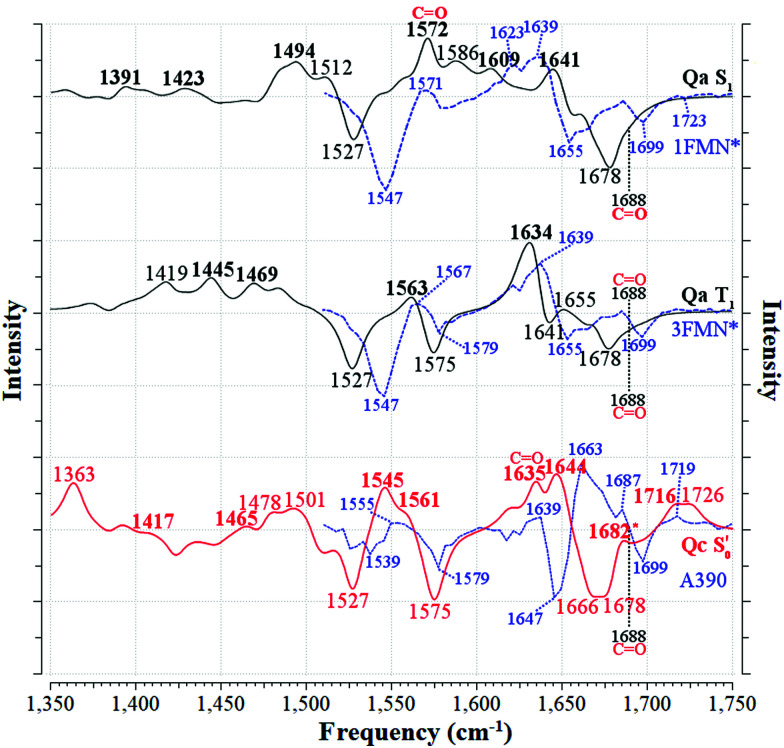
Correlation between the computed infrared difference spectra of EL222 and the experimental evolution associated difference spectra (EADS) of EL222 along the reaction coordinate. The [**Qa**]S_1_–S_0_[**Qa**] and [**Qa**]T_1_–S_0_[**Qa**] spectra are plotted with solid black lines (—) and the 
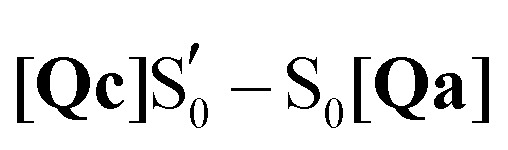
 with a solid red line (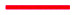). The Q138 side chain carbonyl peaks are labelled for both the excited and GS computed spectra. The corresponding EADS, plotted with dashed blue lines (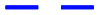), are overlapped with each computed spectrum. Peaks assigned to the experimental EADS peaks are labelled in bold. The calculated spectra were normalised before subtraction, scaled by 0.97 and a half-width at half-height maximum (HWHM) of 8 cm^−1^ was applied.

For EL222 all the produced difference spectra are displayed in Fig. S23–S25 (ESI†). Following the minimum energy path employed in Fig. 5, the spectrum of the most stable isomer at each intermediate is plotted against the experimental EADS produced in this work (Fig. 6). The range from 1750–1510 cm^−1^ is covered by that EADS, and the rest of the fingerprint region down to 1350 cm^−1^ is correlated with the very similar EADS of the referenced work.^32^ For S_1_ and T_1_, the corresponding **Qa** spectrum is employed, while the A390 EADS is compared to the most stable adduct model (**Qc**). As can be seen in Fig. 6, the difference spectra obtained are of adequate similarity to attempt a correlation, which is given to the left of Table 3 for the EL222 minimum energy path. The full assignments for the **Qa–Qc** for **Qb–Qd** are included in the ESI† in Tables S9 and S10 respectively, including the T_1-H_ state correlation with the 3FMN* EADS. Due to the offset of the intensities of the numerous peaks owed to the subtracted ground state spectrum, experimental intensities can be assigned to single vibrations. For example, the 1639 cm^−1^ peak of the EL222 1FMN* EADS is assigned to the 1641 cm^−1^ peak of **Qa** which contains N_3_–H, FMN C=O, and N107 NH_2_ modes in order of decreasing displacement. In the case when no calculated peak is apparent after subtraction in the vicinity of an experimental peak, the strongest vibration in the range is selected. To reduce a portion of the ambiguity in the assignments, mean absolute deviations (MAD) were determined for each correlation and are included in Table 3 and Tables S9, S10 (ESI†). When two calculated peaks are assigned to the same experimental one, the closest of the two is used in the MAD determination irrespective of their relative intensity. The MAD values of the non-deuterated spectra are given in red in parentheses. On the whole, deuteration improves the agreement with experiment, which gives credence to the attempted correlation.

The best agreement with the EL222 1FMN* EADS is provided by **Qa** (MAD: 6.3 cm^−1^) while both **Qa** and **Qc** T_1_ state spectra correlate with mean errors of ∼11 cm^−1^ with the 3FMN* EADS which is similar to the **Qb**, **Qd** models. Overall, the amide **Qa**, **Qc** models fare better than their imidic tautomers specially for the S_1_ and adduct states. The T_1-H_ spectra correlate much worse than the pure T_1_ state with the experimental triplet curve with MAD values of >12.8 cm^−1^ for all models. This is expected, since the short-lived intermediate is challenging to detect in the time resolved experiment and the equilibrated T_1_ state dominates the 3FMN* EADS. The best agreement with the A390 EADS is provided by **Qc** (MAD: 7.6 cm^−1^) which is the expected product of the photocycle (see Section 3.1). However, the A390 EADS curve resembles the steady-state spectrum of EL222 obtained under continuous illumination^32^ and therefore contains changes in protein secondary, tertiary and possibly quaternary structure (oligomerisation). More specifically, unfolding of the A′α-helix,^57,90^ unfolding of Jα-helix,^28,88,91^ and rearrangement of beta-sheets^90^ have been postulated in the spectral region between 1620 and 1670 cm^−1^ of many LOV domains. While the aforementioned large-scale effects are not incorporated in the current computational setup, the good agreement of the computed **Qc** pertains mostly to features above baseline, hinting that quite a few of those features can be assignable to small-scale effects. The changes evident in the negative portion of the EADS (detailed in the analysis of the ground state spectra in Sections 3.3–3.4) reflect the protein dynamics of which the computed subtracted spectra cannot reproduce. This affects the overall line shape and particularly the area around 1647 cm^−1^ where the A390 EADS exhibits a strong bleach.

**Fig. 7:**
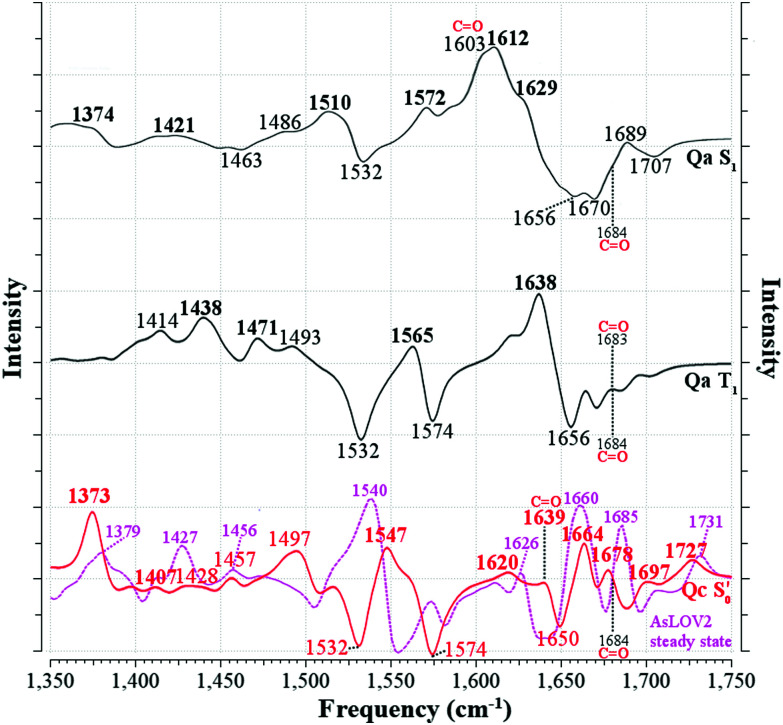
Computed infrared difference spectra of AsLOV2 along the reaction coordinate. The [**Qa**]S_1_–S_0_[**Qa**] and [**Qa**]T_1_–S_0_[**Qa**] spectra are plotted with solid black lines (—) and the 
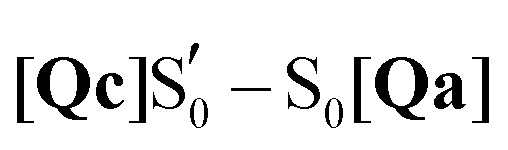
 with a solid red line (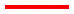). Peaks assigned to the experimental EADS peaks are labelled in bold. The calculated spectra were normalised before subtraction, scaled by 0.97 and a half-width at half-height maximum (HWHM) of 8 cm^−1^ was applied. The Q513 side chain carbonyl peaks are labelled for both the excited and GS computed spectra. The steady-state spectrum of a truncated AsLOV2 protein is plotted with dashed magenta line (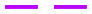), overlapped to the computed 
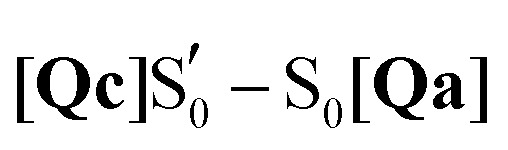
 spectrum, and is reprinted from the referenced work.^57^

For AsLOV2, all generated spectra are displayed in Fig. S26–S28 (ESI†). Following the reaction coordinate employed in Fig. 5, the difference spectrum of the most stable model at each intermediate step is plotted in Fig. 7. The experimental-theoretical assignments for that reaction coordinate are given in the right portion of Table 3, which are only based on the referenced work,^32^ together with their respective MAD values. The full assignments of models **Qa–Qd**, including T_1-H_, are given in the right part of Tables S9 and S10 (ESI†). The agreement between the **Qa** S_1_ and T_1_ spectra and the respective EADS is satisfactory, with MAD values of 6.0 and 9.5 cm^−1^, respectively while **Qc** fits slightly better with the experimental curve in the latter (8.3 cm^−1^). As is the case with EL222, none of the T_1-H_ models’ spectra correlates well with the 3FMN* EADS (MAD > 16 cm^−1^), whereas in the adduct, **Qc** exhibits the best overall agreement (8.5 cm^−1^), on a par with EL222, demonstrating that positive features of the A390 EADS can be attributed to changes around the chromophore.

Comparing the excited 1FMN* and 3FMN* EADS of AsLOV2, the peaks at 1375 and 1413 cm^−1^ disappear in the former, giving rise to two peaks at 1438 cm^−1^ and 1491 cm^−1^ in the latter.^32^ The first three are assigned mostly to methylene scissoring and C–H/N–H rocking modes (**Qa**), and 1491 cm^−1^ involves FMN breathing. On the other hand, in the T_1_ of EL222 the downshift of the lower transient (1428 cm^−1^) is not predicted by the calculations (assigned to 1445 cm^−1^). In the higher frequency region of AsLOV2, the strongest peak is located at 1622 cm^−1^ and remains unaltered between S_1_ and T_1_. It is assigned to a doublet at 1612 cm^−1^ and 1629 cm^−1^ (S_1_, **Qa**) involving side chain and FMN carbonyl stretches and NH_2_ scissoring. In contrast, in the EL222 1FMN* EADS the peak is resolved to a doublet experimentally, with the more intense at 1639 cm^−1^ and the weaker peak at 1623 cm^−1^, assigned to the computed **Qa** peaks at 1641 cm^−1^ and 1609 cm^−1^, respectively, which are constituted by a mix of FMN carbonyl and NH_2_ scissoring modes. Progressing to the adduct, the AsLOV2 spectrum includes more features, where ten transient peaks are resolved compared to seven in EL222. It is also clear for the AsLOV2 adduct that the computed combined spectra cannot reproduce the bleach signals at 1553 and 1625 cm^−1^ of the A390 EADS. The AsLOV2 triplet population decays within 9.5 μs [5 μs] (EL222 lifetime in brackets), *i.e.* the adduct rises with the same lifetime, so most likely the A390 EADS includes extensive structural changes in the protein.^28,32^ Indeed, MD simulations predict pronounced changes in AsLOV2, including dissociation of the N482/N492 residues from FMN within a couple of μs and rapid Jα-helix unfolding.^7,48^ The bleach at 1625 cm^−1^ and transient at 1634 cm^−1^ have been attributed to secondary structure changes,^28,32^ however the latter peak is assigned here to the glutamine carbonyl stretch in **Qc** (see also the discussion in the following paragraph). In EL222 the 1555(+)/1539(−) cm^−1^ pair was assigned to 1545(+)/1527(−) cm^−1^ involving N107 and FMN in the excited, but only FMN in the ground state. The equivalent pair in AsLOV2 is reversed (1553(−)/1541(+) cm^−1^) which is not reproduced by the calculations 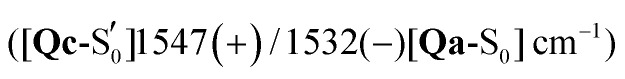 both assigned to FMN breathing and N482/Q454 NH_2_ scissoring. Nevertheless, the calculations capture the downshift of the aforementioned positive transient from the 3FMN* to the A390 EADS. That downshift is more evident in the EL222 EADS (Fig. 4c), which is similarly captured in the referenced work,^32^ rather than AsLOV2. For EL222 the triplet 1567 cm^−1^ (1563 cm^−1^) peak downshifts to 1555 cm^−1^ (1545 cm^−1^) in the adduct, and for AsLOV2 from 1567 cm^−1^ (1565 cm^−1^) to 1541 cm^−1^ (1547 cm^−1^), with calculated peaks in parentheses. All these vibrations involve FMN ring modes (Table 3) which could act as a marker band for adduct formation. Finally, the highest observed adduct transients of AsLOV2 and EL222 (1722 cm^−1^ and 1719 cm^−1^, respectively) are assigned to the 1727/1726 cm^−1^ computed peaks due to C_4_=O_4′_ stretching coupled to N_3_–H bending (**Qc**). This assignment is in agreement with previous studies on other LOV domains,^92,93^ and acts as another marker for adduct formation and possibly, glutamine rotation (**Qa** C_4_=O_4′_ stretches are predicted at ∼1700 cm^−1^, Table 2).

Recently, an additional intermediate between the triplet and the adduct state of AsLOV2 was reported,^7^ with an EADS time constant of 8.8 μs. The spectrum of this species resembles more the A390 rather than the 3FMN* EADS, unlike the intermediate described here (T_1-H_). The most prominent feature of the 8.8 μs EADS is the appearance of the aforementioned 1625(−)/1634(+) cm^−1^ pair present in the A390 EADS (1636(+) cm^−1^ in the reference). With the aid of an N414Q mutation the authors postulate that the peak reports either directly to the Q513 side chain carbonyl or the N414–Q513 interaction, which is the trigger for Jα-helix unfolding. As detailed in Table 2, with the exception of S_1_, the Q513 C=O stretching mode is not expected to be found below 1683 cm^−1^ up to the adduct. In the adduct upon rotation of the Q513 side chain, a shift to 1639 cm^−1^ is registered, which is coupled to the N414 side chain carbonyl stretch shift from 1709 cm^−1^ to 1684 cm^−1^ (Fig. 5). Therefore, the calculations support the assignment of the A390 1634 cm^−1^ peak to the Q513 side chain C=O stretch,^7^ but cannot rule out Jα unfolding.^28,32^ The equivalent A390 peak in EL222 (1639 cm^−1^), is assigned to 1635 cm^−1^ (**Qc**) which is also due to the glutamine C=O stretch but here it is coupled to one of the A39 backbone carbonyl modes. Interestingly, the steady-state difference FTIR spectra of an AsLOV2 variant lacking the A′α and Jα extensions^57^ produced a very good agreement with the computed AsLOV2 
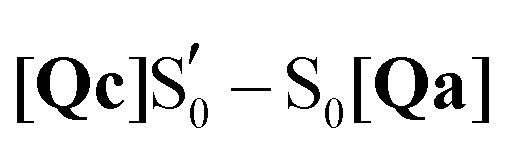
 spectrum (MAD = 5.7 cm^−1^ – compared to 7.6 cm^−1^ with the AsLOV2 A390 EADS). The two spectra are overlaid in the bottom of Fig. 7, and as can be seen, apart from a good MAD value, there is also a good match in relative intensities. In this case, the Q513 side chain carbonyl of **Qc** is assigned to the 1626 cm^−1^ transient which is redshifted by 8 cm^−1^ compared to the full protein EADS spectrum. This assignment does not necessarily exclude the possibility of changes in β-sheet folds.^90,94^

The produced difference spectra for RsLOV are included in Fig. S29–S31 (ESI†); however, since to the best of our knowledge, no experimental time resolved IR spectrum has been published to date, a theoretical/experimental correlation was not feasible. Nevertheless, the computed infrared spectra presented here could be used to facilitate the interpretation of future experimental efforts addressing RsLOV.

A summary of the assessment of the glutamine isomers by energetics and spectroscopic terms is provided in Table 4. RsLOV is not evaluated by spectroscopy due to the absence of experimental spectra. When evaluating the spectroscopic correlation solely by MAD values, in some instances it proved difficult to distinguish between the spectra of the various glutamine isomers, such as in the S_0_ and T_1_ states of EL222. This exception aside, both approaches concur to a large extend to the proposed glutamine isomer for each state. Specifically, both spectroscopy and energetics point that **Qc** is the most likely end product of the studied photocycle of EL222 and AsLOV2, while other intermediates are suggested with a smaller degree of confidence. The stable flipped glutamine in the adduct can propagate the large-scale changes in the protein, while in the H-transfer step, comparatively higher barriers have to be overcome and smaller resultant stabilisation is expected.

**Table 4:** Summary of the most plausible glutamine configurations along the reaction coordinate according to either computed Δ*E*s/Δ*G*s, or similarity between computed and experimental infrared EADS spectra *via* their MAD values

		S_0_	S_1_	T_1_	T_1-H_	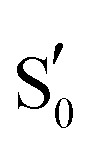
EL222	Energetics	**Qa**	**Qa** a	**Qa**	**Qc**	**Qc**
	Spectroscopy	**Qc**	**Qa**	**Qd**	N/A	**Qc**
AsLOV2	Energetics	**Qa**	**Qa**	**Qa**	**Qc** b	**Qc**
	Spectroscopy	**Qa**	**Qa**	**Qc**	N/A	**Qc**
RsLOV	Energetics	**Qa**	**Qa**	**Qa**	**Qa**	**Qa**
	Spectroscopy	N/A	N/A	N/A	N/A	N/A

a The energy of **Qc** is disregarded, see discussion in Section 3.1.

b Conversion back to **Qa** is possible due to the small reverse barrier.

The approach detailed here could benefit from QM clusters extracted from MD trajectories,^6,7^ as an alternative of relying on the dark and light crystal structures. AsLOV2 in particular features large alterations in the vicinity of the chromophore as early in the photocycle as 1.2 μs.^7^ This could improve the assignment in the adduct, where the largest changes are observed in the experimental spectra and the produced difference plots do not follow the +/− patterns of the experimental curves. Moreover, a thorough exploration of the excited potential energy surfaces of the photocycle could establish a minimum energy path with higher confidence.

## 4. Conclusions

The computational study presented here describes the essential excited singlet, triplet and adduct intermediates of the photocycle of three different LOV domains, chosen due to their distinct geometries around the chromophore. The crucial glutamine side chain rotation was explored together with the possibility of imidic tautomerisation, which had been reported before for the BLUF domain. The rotation of the glutamine was found to be sensitive to the LOV domain topology and, in particular, to the stabilisation provided by residues of the adjacent β-sheet. In EL222 and AsLOV2 the glutamine flip is energetically favoured at the adduct formation which for the latter proceeds barrierless, while in RsLOV glutamine rotation is not attainable at any step. A reason for the different behaviour is that in RsLOV the glutamine side chain is afforded additional stabilisation by the threonine hydroxy group which can H-bond to both the amide and carbonyl groups, in a similar role that a conserved tyrosine plays in the BLUF photocycle. Changes in the glutamine side chain before the adduct state cannot be completely excluded but are predicted less favourable. At no stage of the LOV domain photocycles were the imidic variants favoured energetically, while possible tautomerisation marker bands such as the C=N stretch mode appear with weak intensity. Infrared spectra of four possible isomers were collected along a tentative reaction coordinate up to the adduct, and key marker bands were followed, such as the carbonyl stretching vibrations of FMN, the side chain glutamine, and the asparagine residues. It was found that Q138 in EL222 and Q513/N414 in AsLOV2 experience large IR spectral shifts upon glutamine rotation. Finally, difference spectra were devised and directly compared to the solid body of experimental spectra published in the literature,^7,28,32,33^ and our own experiments on EL222. A full assignment of excited and ground state peaks in the 1350–1750 cm^−1^ fingerprint region was completed for both EL222 and AsLOV2. Reasonable agreement was reached between the experimental and theoretical difference spectra with a mean absolute deviation well within 11 cm^−1^ for the assigned peaks of most intermediates. An exception was the triplet biradical intermediate which did not correlate well with the available 3FMN* EADS (MAD: >15 cm^−1^ for most models). The adduct EADS spectra are marred by large scale protein dynamics in the amide I region which could not be reproduced with the current setup. On the other hand, positive transients gave a ∼8 cm^−1^ agreement with calculations for the inverted glutamine models of EL222 and AsLOV2. Moreover, the calculations assigned the A390 transient appearing at 1634 and 1639 cm^−1^ for EL222 and AsLOV2, respectively to the carbonyl stretch of the glutamine side chain after flipping from its original configuration, agreeing with recent experimental suggestions. Direct experimental evidence of the glutamine side chain orientation could be obtained *via* isotopic labelling of the glutamine itself^95,96^ or other interacting residues. Future combined computational/experimental studies can benefit from the experimental study of shorter LOV domains, as was demonstrated here by the superior correlation reached when employing a truncated AsLOV2 protein (MAD = 5.7 cm^−1^).

## Conflicts of interest

There are no conflicts of interest to declare.

## Supplementary Material

CP-023-D1CP00447F-s001
