# Supplementary material for: QM calculations predict the energetics and infrared spectra of transient glutamine isomers in LOV photoreceptors
Source: Phys Chem Chem Phys. 2021 Jun 18;23(25):13934–50. doi: 10.1039/d1cp00447f (PMC8246142; doi:10.1039/d1cp00447f)
Supplement: CP-023-D1CP00447F-s001 [file CP-023-D1CP00447F-s001.pdf]

Electronic Supplementary Material (ESI) for Physical Chemistry Chemical Physics.
This journal is © the Owner Societies 2021

SUPPLEMENTARY INFORMATION

QM calculations predict the energetics and infrared spectra of transient glutamine isomers in LOV photoreceptors

Prokopis C. Andrikopoulos,*[a] Aditya S. Chaudhari,[a] Yingliang Liu,[a] Patrick E. Konold,[b] John T. M. Kennis,[b] Bohdan Schneider,[a] and Gustavo Fuertes,*[a]

[a] Institute of Biotechnology of the Czech Academy of Sciences, BIOCEV, Průmyslová 595, CZ-252 50 Vestec, Czechia

[b] Department of Physics and Astronomy, Faculty of Sciences, Vrije Universiteit, 1081 De Boelelaan, 1081HV Amsterdam, The Netherlands

Correspondence to: Prokopis C. Andrikopoulos; e-mail: prokopios.andrikopoulos@ibt.cas.cz; and Gustavo Fuertes e-mail: gustavo.fuertes@ibt.cas.cz

LIST OF CONTENTS

Page 3 **1. Electronic and Geometry Data**

Page 3 Figure S1 Optimised structures of the S_0 **Qa** models of EL222, AsLOV2 and RsLOV highlighting fixed atoms to the X-Ray positions.

Page 3 Figure S2-S3 Optimised structures of EL222 along the reaction coordinate.

Page 4 Figure S4-S5 Optimised structures of AsLOV2 along the reaction coordinate.

Page 4 Figure S6-S7 Optimised structures of RsLOV along the reaction coordinate.

Page 5 Figure S8 Relative energies along the reaction path of the EL222 cluster.

Page 5 Figure S9 Relative energies along the reaction path of the AsLOV2 cluster.

Page 6 Figure S10 Relative energies along the reaction path of the RsLOV cluster.

Page 7 Figure S11 Electron density difference surface plots between the S_0 and S_1 state and spin density plots of the $T_1/T_{1\text{-}H}$ states of the EL222 cluster **Qa-Qd** models.

Page 8 Figure S12 Differential electron density plots of the S_1 state of the **Qa** and **Qc** models of EL222, AsLOV2 and RsLOV.

Page 9 Table S1 Key distances and glutamine dihedral angle of the EL222 cluster along the reaction coordinate.

Page 10 Table S2 Key distances and glutamine dihedral angle of the AsLOV2 cluster along the reaction coordinate.

Page 11 Table S3 Key distances and glutamine dihedral angle of the RsLOV cluster along the reaction coordinate.

Page 11 Table S4 Mulliken charges of the isoalloxazine moiety of the EL222, AsLOV2 and RsLOV cluster models **Qa-Qd** along the reaction coordinate.

Page 12 **2a. Vibrational Data**

Page 12 Brief description of computed spectra not analysed in the main text.

Page 13 Figure S13 Calculated Infrared Spectra of the S_0 of the EL222 cluster.

Page 14 Figure S14 Calculated Infrared Spectra of the S_1 state of the EL222 cluster.

Page 15 Figure S15 Calculated Infrared Spectra of the T_1 and $T_{1\text{-}H}$ states of the EL222 cluster.

Page 16 Figure S16 Calculated Infrared Spectra of the S_0' state of the EL222 cluster.

Page 17 Figure S17 Calculated Infrared Spectra of the S_0 and S_1 states of the AsLOV2 cluster.

Page 18 Figure S18 Calculated Infrared Spectra of the T_1 and $T_{1\text{-}H}$ states of the AsLOV2 cluster.

Page 19 Figure S19 Calculated Infrared Spectra of the S_0' state of the AsLOV2 cluster.

Page 20 Figure S20 Calculated Infrared Spectra of the S_0 and S_1 states of the RsLOV cluster.

Page 21	Figure S21 Calculated Infrared Spectra of the T_1 and $T_{1\text{-}H}$ states of the RsLOV cluster.
Page 22	Figure S22 Calculated Infrared Spectra of the S_0' state of the RsLOV cluster.
Page 23	Table S5 Assignment Tables for major vibrations of the EL222 cluster.
Page 24-26	Table S6 Assignment Tables for major vibrations of the AsLOV2 and RsLOV clusters.
Page 27	Table S7 Evolution of selected **Qb** and **Qd** EL222 and AsLOV2 IR peaks along the reaction coordinate.
Page 28	Table S8 Evolution of selected RsLOV IR peaks along the reaction coordinate.
Page 29	**2b. Difference Spectra**
Page 29	Figure S23 Difference Spectra between the S_0 and S_1 states of the EL222 cluster.
Page 30	Figure S24 Difference Spectra between the S_0 and the T_1 and $T_{1\text{-}H}$ states of the EL222 cluster.
Page 31	Figure S25 Difference Spectra between the S_0 and the S_0' states of the EL222 cluster.
Page 32	Figure S26 Difference Spectra between the S_0 and S_1 states of the AsLOV2 cluster.
Page 33	Figure S27 Difference Spectra between the S_0 and the T_1 and $T_{1\text{-}H}$ states of the AsLOV2 cluster.
Page 34	Figure S28 Difference Spectra between the S_0 and S_0' states of the AsLOV2 cluster.
Page 35	Figure S29 Difference Spectra between the S_0 and S_1 states of the RsLOV cluster.
Page 36	Figure S30 Difference Spectra between the S_0 and the T_1 and $T_{1\text{-}H}$ states of the RsLOV cluster.
Page 37	Figure S31 Difference Spectra between the S_0 and S_0' states of the RsLOV cluster.
Page 38	Table S9 Assignments of the experimental EADS spectra based on the computed **Qa** and **Qc** difference spectra of EL222 and AsLOV2 along the reaction coordinate.
Page 39	Table S10 Assignments of the experimental EADS spectra based on the computed **Qb** and **Qd** difference spectra of EL222 and AsLOV2 along the reaction coordinate.
Page 40-66	**3. Cartesian Coordinates**

1. Electronic and Geometry Data

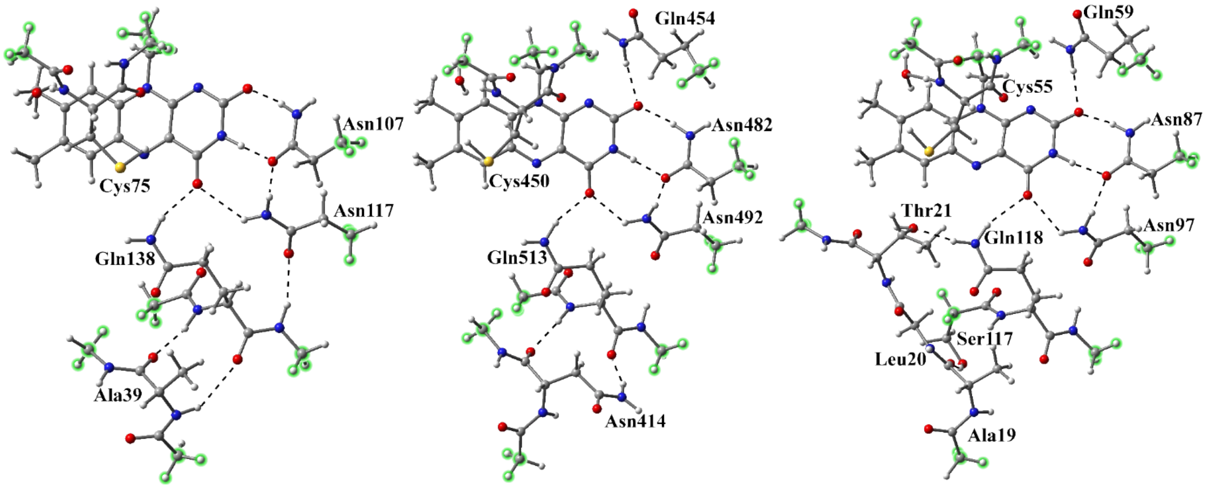

Figure S1 Optimised structures of the ground state **Qa** models of EL222 (left), AsLOV2 (middle) and RsLOV (right). Fixed atoms to the X-ray positions are highlighted in green and the hydrogen bonding network is displayed with dashed lines.

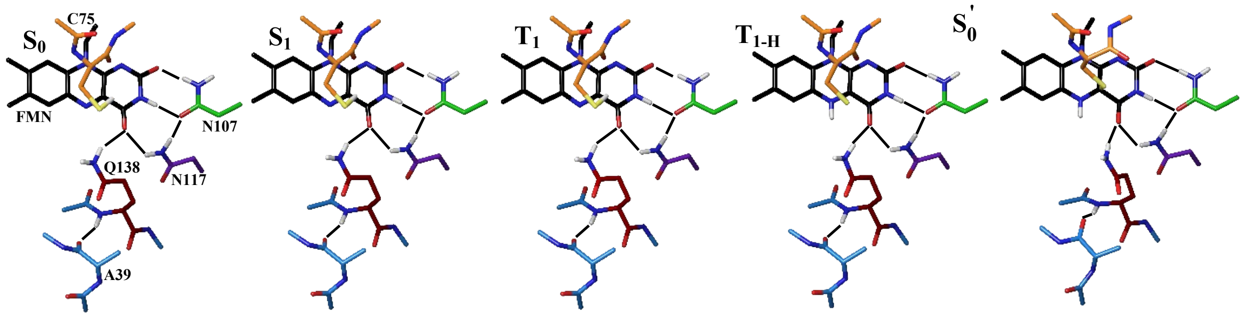

Figure S2 Optimised **Qa** structures of the EL222 cluster along the reaction coordinate. The hydrogen bonding network is shown with black dashed lines.

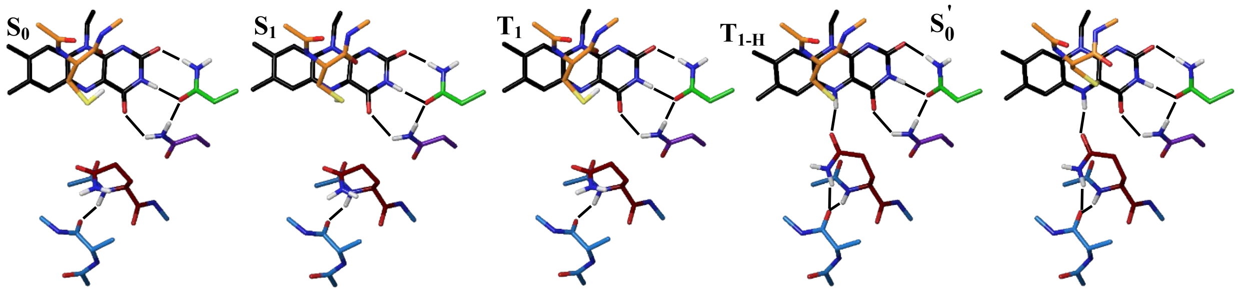

Figure S3 Optimised **Qc** structures of the EL222 cluster along the reaction coordinate. The hydrogen bonding network is shown with black dashed lines.

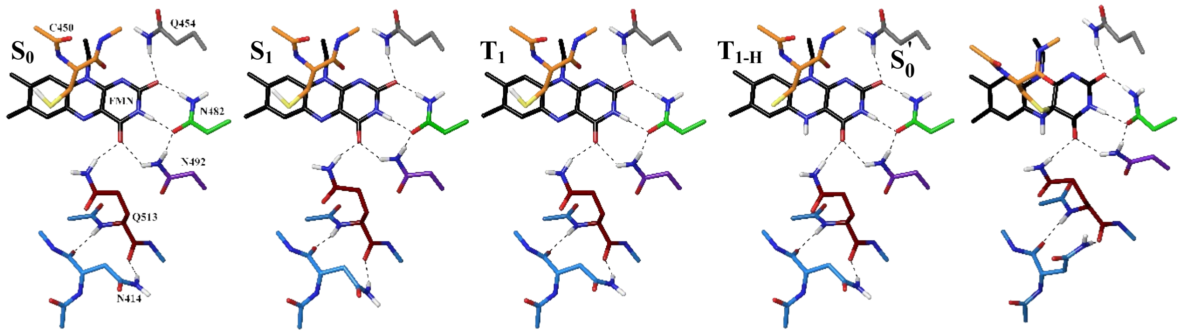

Figure S4 Optimised **Qa** structures of AsLOV2 along the reaction coordinate. The hydrogen bonding network is shown with black dashed lines.

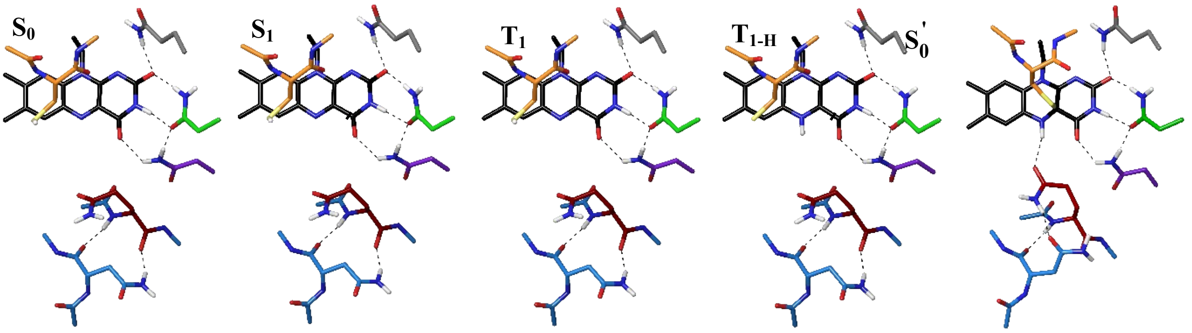

Figure S5 Optimised **Qc** structures of AsLOV2 along the reaction coordinate. The hydrogen bonding network is shown with black dashed lines.

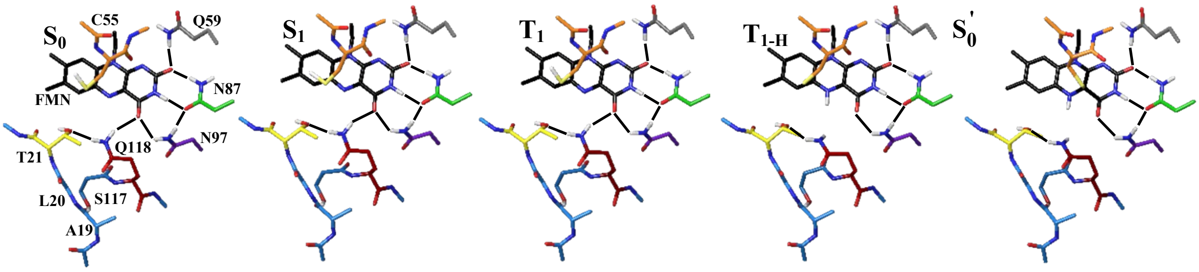

Figure S6 Optimised **Qa** structures of RsLOV along the reaction coordinate. The hydrogen bonding network is shown with black dashed lines.

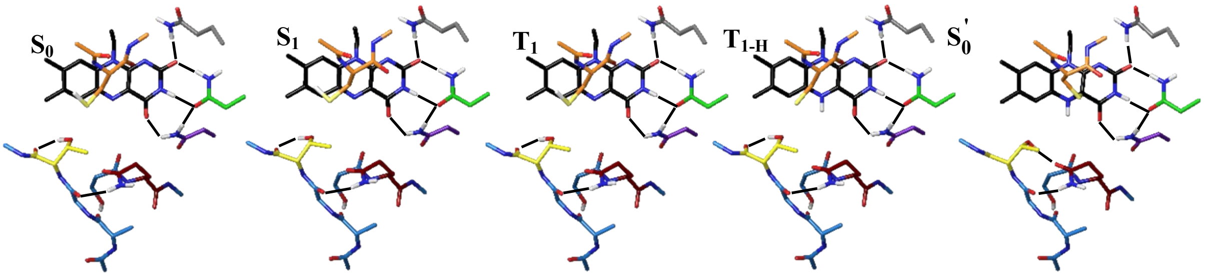

Figure S7 Optimised **Qc** structures of RsLOV along the reaction coordinate. The hydrogen bonding network is shown with black dashed lines.

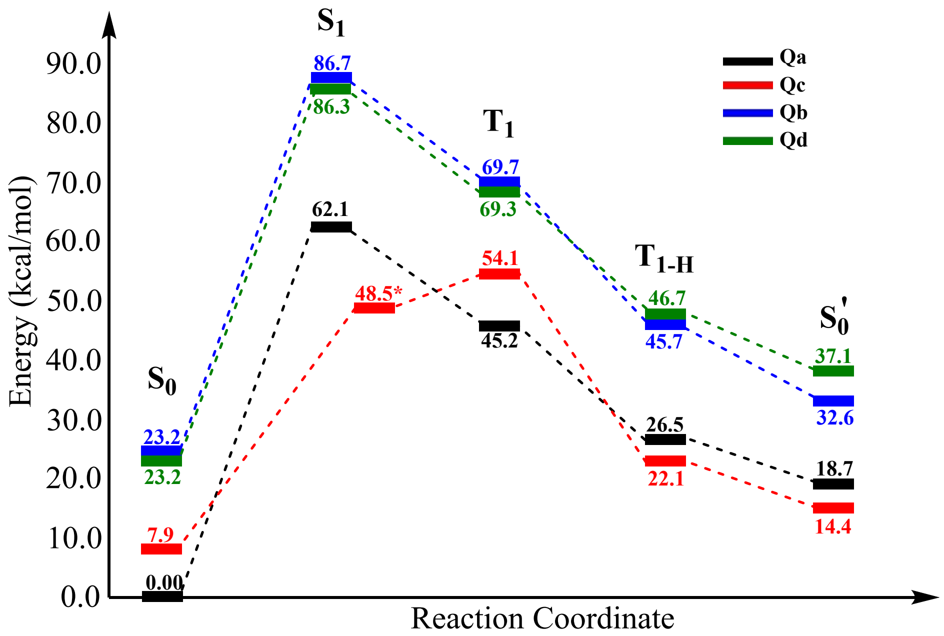

Figure S8 Relative energies along the reaction path of the EL222 cluster. The imidic Q138 isomers **Qb** and **Qd** are displayed with blue and green, and the amidic **Qa** and **Qc** in black and red respectively. *: The **Qc** S_1 structure is placed further along the reaction coordinate relative to the rest of the S_1 and T_1 structures (See discussion in Section 3.1 of the main text).

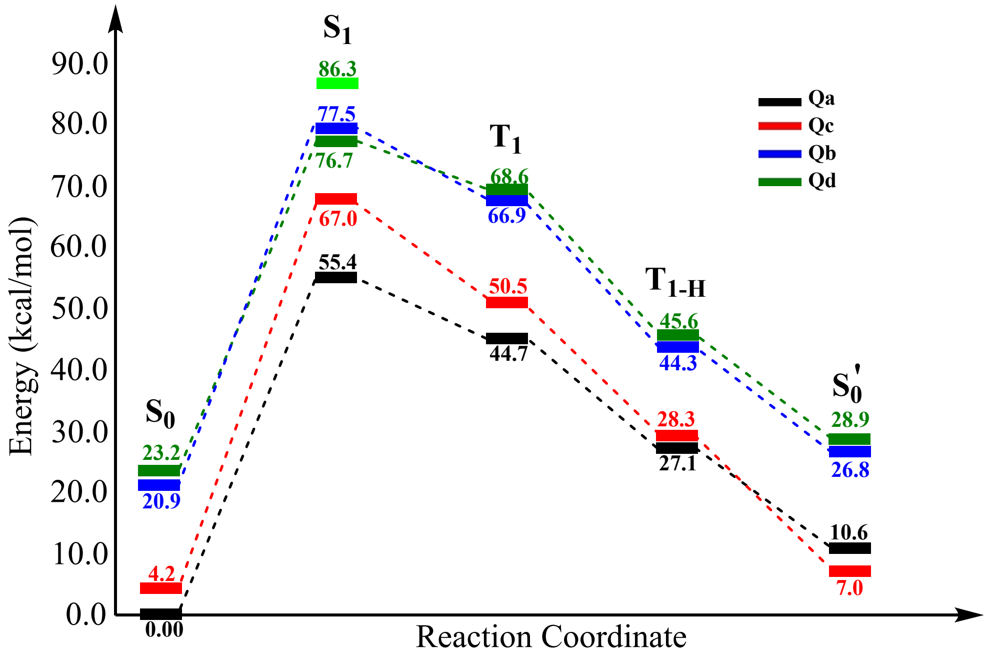

Figure S9 Relative energies along the reaction path of the AsLOV2 cluster. The imidic Q513 isomers **Qb** and **Qd** are displayed with blue and green, and the amidic **Qa** and **Qc** in black and red respectively. The relative energy of a second $\pi\pi^*$ degenerate **Qd** excitation is shown in light green.

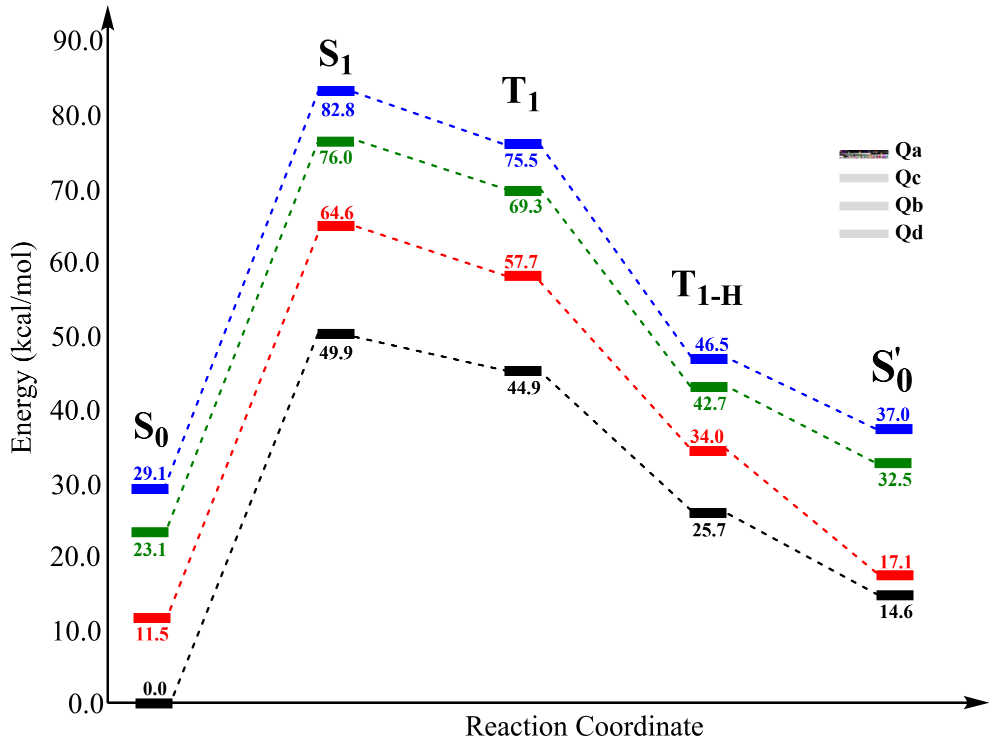

Figure S10 Relative energies along the reaction path of the RsLOV cluster. The imidic Q118 isomers **Qb** and **Qd** are displayed with blue and green, and the amidic **Qa** and **Qc** in black and red respectively.

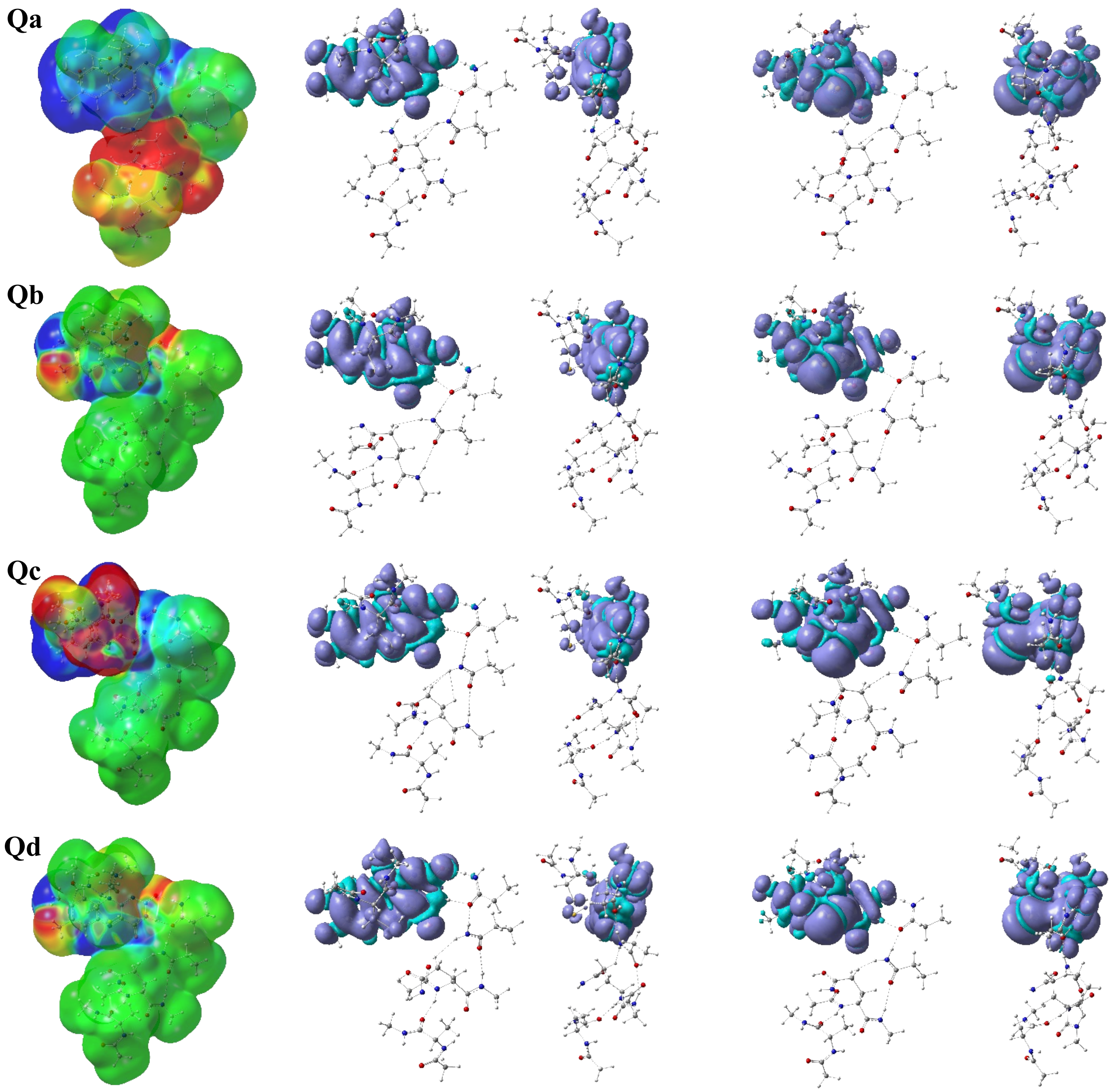

Figure S11 Left: Electron density difference plots of the S_1 state of the EL222 clusters **Qa-Qd** mapped on the total density (density isosurface = 0.0001). Blue areas indicate positive values *i.e.* where excited state density is larger than the ground state density, and red indicates larger ground state density. Middle: Front and side views of spinA-spinB density of the T_1 state of the EL222 cluster models **Qa-Qd**. Right: Front and side views of spinA-spinB density of T_1-H of the EL222 cluster models **Qa-Qd**.

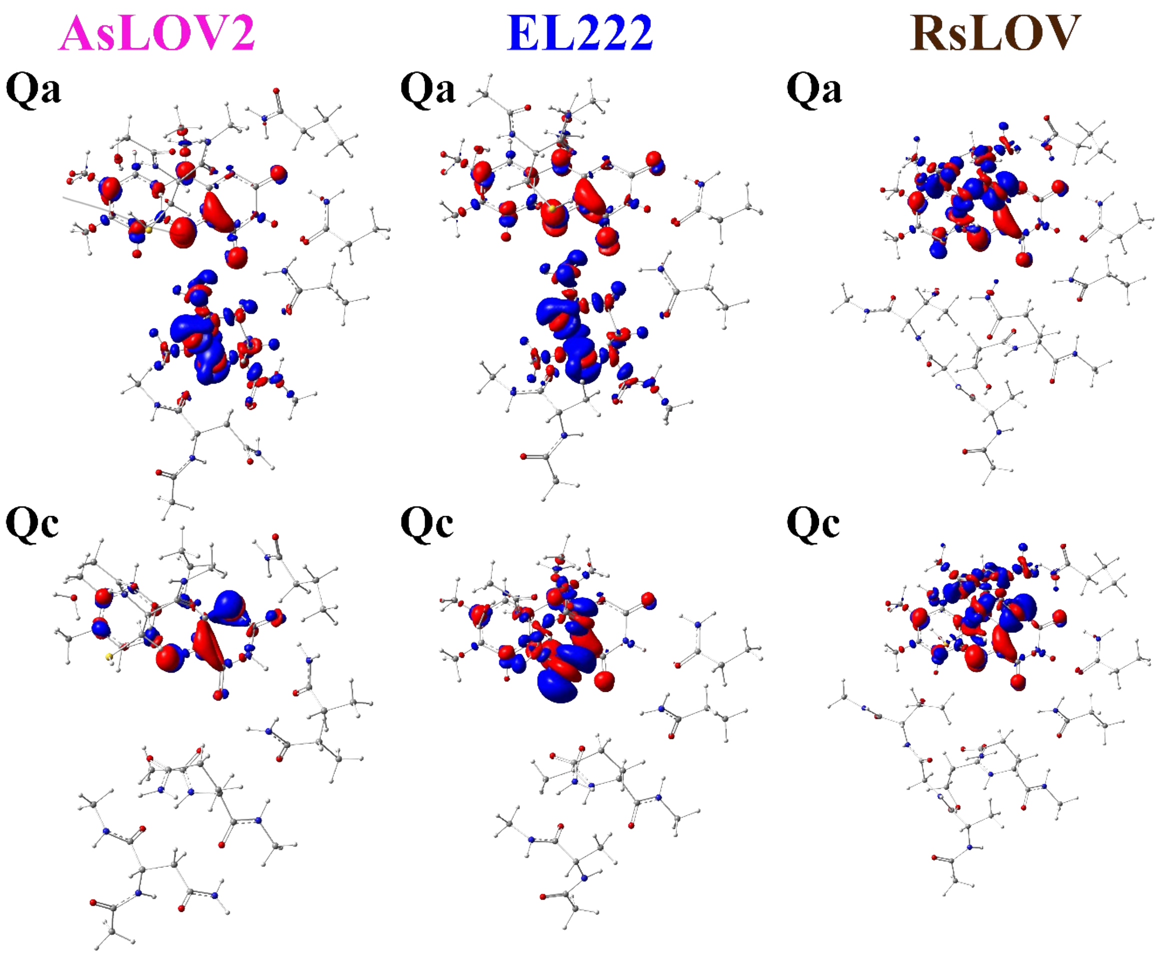

Figure S12 Differential electron density plots of the S_1 state of the **Qa** and **Qc** models of EL222, AsLOV2 and RsLOV.

Table S1 Key distances (in Å) and Q138 sidechain dihedral angle of the EL222 cluster along the reaction coordinate.

		N107 $O\cdots HN_3$	N107 $NH\cdots O_{2'}$	C75 $S\cdots C_{4a}$	C75 $SH\cdots N_5$	N117 $NH\cdots O_{4'}$	Q138 $NH\cdots O_{4'}$	Q138 CCCN	FMN $C_2{=}O_{2'}$	FMN $C_4{=}O_{4'}$
S_0	**Qa**	1.818	1.894	3.865	2.953	2.126	2.524	-167.5	1.233	1.229
	**Qb**	1.836	1.919	5.270	5.675	2.443	5.257	-131.3	1.235	1.224
	**Qc**	1.815	1.935	5.277	5.480	2.463	5.280	64.6	1.235	1.224
	**Qd**	1.897	1.830	3.924	2.916	2.446	5.977	53.2	1.234	1.224
S_1	**Qa**	1.838	1.910	3.802	2.862	2.449	2.008	-167.9	1.237	1.241
	**Qb**	1.836	1.916	3.860	2.853	2.407	5.454	-132.8	1.238	1.231
	**Qc**	1.891	1.863	3.453	1.674	2.956	5.447	59.5	1.246	1.240
	**Qd**	1.846	1.915	3.843	2.867	2.408	5.945	53.4	1.237	1.231
T_1	**Qa**	1.823	1.920	3.819	2.976	2.501	2.114	-166.8	1.236	1.235
	**Qb**	1.819	1.924	3.917	2.867	2.435	5.458	-132.8	1.237	1.230
	**Qc**	1.821	1.921	3.894	2.897	2.452	5.332	58.9	1.237	1.230
	**Qd**	1.829	1.919	3.887	2.914	2.442	5.939	53.5	1.237	1.230
$T_{1\text{-}H}$	**Qa**	1.830	1.856	3.804	1.018	2.575	1.993	-178.4	1.239	1.246
	**Qb**	1.824	1.851	3.658	1.022	2.397	4.836	-141.4	1.241	1.239
	**Qc**	1.803	1.882	3.760	1.038	2.158	5.551	16.4	1.243	1.237
	**Qd**	1.835	1.857	3.658	1.019	2.429	5.856	47.9	1.240	1.240
	**TS**	1.845	1.850	3.625	1.019	2.555	4.206	94.0	1.240	1.240
S_0'	**Qa**	1.856	1.851	1.943	1.017	2.618	2.048	-165.3	1.228	1.226
	**Qb**	1.797	1.836	1.948	1.020	2.541	4.398	-127.9	1.230	1.220
	**Qc**	1.753	1.841	1.968	1.030	2.508	5.319	23.3	1.232	1.221
	**Qd**	1.856	1.841	1.945	1.018	2.663	5.602	48.2	1.229	1.221
	**TS**	1.792	1.852	1.943	1.018	2.596	2.438	113.6	1.229	1.223

Table S2 Key distances (in Å) and Q513 sidechain dihedral angle of the AsLOV2 cluster along the reaction coordinate.

		N482 O···HN_3	N482 NH···$O_{2'}$	Q454 NH···$O_{2'}$	C450 S···C_{4a}	C450 SH···N_5	N492 NH···$O_{4'}$	Q513 NH···$O_{4'}$	Q513 CCCN	FMN C_2=$O_{2'}$	FMN C_4=$O_{4'}$
S_0	**Qa**	1.776	1.900	2.033	5.093	5.304	2.469	2.075	-171.5	1.242	1.229
	**Qb**	1.836	1.919	2.040	5.270	5.675	2.443	5.257	-131.3	1.242	1.222
	**Qc**	1.815	1.936	1.992	5.277	5.480	2.463	5.280	64.6	1.245	1.224
	**Qd**	1.811	1.928	1.997	5.235	4.971	2.473	5.953	50.9	1.245	1.223
S_1	**Qa**	1.893	1.819	1.926	5.099	5.313	2.397	1.858	176.8	1.261	1.252
	**Qb**	2.023	1.846	2.006	5.457	5.870	1.743	5.302	-135.2	1.261	1.254
	**Qc**	1.859	1.938	2.016	5.186	5.380	2.390	5.248	64.1	1.245	1.232
	**Qd**	1.875	1.904	2.019	5.065	4.739	2.448	5.857	51.6	1.246	1.231
	**Qd2**	1.956	1.866	1.905	5.290	5.790	2.395	5.918	51.7	1.261	1.237
T_1	**Qa**	1.771	1.927	2.040	5.043	5.290	2.450	2.067	-172.7	1.243	1.236
	**Qb**	1.829	1.938	2.039	5.177	5.565	2.442	5.243	-130.7	1.243	1.229
	**Qc**	1.810	1.952	2.016	5.204	5.414	2.456	5.216	64.2	1.246	1.230
	**Qd**	1.798	1.938	2.026	5.144	4.956	2.499	5.893	51.3	1.244	1.229
$T_{1\text{-H}}$	**Qa**	1.793	1.866	2.006	5.034	1.018	2.581	2.010	-178.7	1.248	1.245
	**Qb**	1.846	1.896	2.022	5.244	1.021	2.384	5.023	-138.3	1.248	1.238
	**Qc**	1.824	1.912	1.967	5.107	1.019	2.437	5.344	63.8	1.250	1.239
	**Qd**	1.858	1.911	2.014	5.141	1.020	2.349	5.929	43.0	1.248	1.239
	**TS**	1.788	1.876	1.978	5.067	1.019	2.624	3.251	99.4	1.250	1.240
S'_0	**Qa**	1.762	1.868	2.020	1.953	1.021	2.373	2.207	148.0	1.237	1.226
	**Qb**	1.765	1.872	2.007	1.966	1.029	2.271	4.338	-164.1	1.238	1.221
	**Qc**	1.755	1.877	2.001	1.966	1.026	2.284	5.321	23.1	1.239	1.221
	**Qd**	1.753	1.874	2.012	1.950	1.021	2.314	5.427	5.7	1.238	1.221
	**TS**	1.784	1.848	2.003	1.949	1.017	2.358	5.311	55.4	1.238	1.221

Table S3 Key distances (in Å) and Q118 sidechain dihedral angle of the RsLOV cluster along the reaction coordinate.

		N87 O···HN_3	N87 NH···$O_{2'}$	Q59 NH···$O_{2'}$	C55 S···C_{4a}	C55 SH···N_5	N97 NH···$O_{4'}$	Q118 NH···$O_{4'}$	Q118 CCCN	FMN C_2=$O_{2'}$	FMN C_4=$O_{4'}$
S_0	**Qa**	1.822	1.823	1.923	5.093	5.317	2.363	2.340	-156.8	1.242	1.225
	**Qb**	1.796	1.831	1.904	5.106	5.340	2.429	5.379	-119.9	1.245	1.219
	**Qc**	1.826	1.822	1.907	5.080	5.325	2.418	5.173	73.3	1.244	1.220
	**Qd**	1.781	1.837	1.907	5.090	5.295	2.376	5.761	19.0	1.244	1.219
S_1	**Qa**	1.930	1.781	1.822	5.224	5.086	2.214	2.110	-163.8	1.260	1.241
	**Qb**	1.883	1.757	1.808	4.802	4.484	2.358	5.351	-116.9	1.263	1.230
	**Qc**	1.925	1.749	1.812	4.780	4.461	2.346	4.963	77.5	1.263	1.232
	**Qd**	1.871	1.760	1.812	4.796	4.368	2.324	5.593	24.8	1.263	1.231
T_1	**Qa**	1.818	1.843	1.936	5.040	5.304	2.325	2.330	-157.8	1.244	1.231
	**Qb**	1.791	1.850	1.919	5.049	5.313	2.409	5.373	-118.3	1.246	1.226
	**Qc**	1.821	1.840	1.923	5.019	5.300	2.401	5.101	75.64	1.246	1.227
	**Qd**	1.784	1.855	1.922	5.035	5.253	2.348	5.703	19.6	1.246	1.227
$T_{1\text{-}H}$	**Qa**	1.823	1.793	1.892	5.096	1.022	2.346	3.653	-136.7	1.249	1.237
	**Qb**	1.756	1.813	1.874	5.229	1.047	2.141	4.195	-157.6	1.252	1.235
	**Qc**	1.848	1.792	1.892	5.083	1.020	2.381	5.193	70.4	1.249	1.237
	**Qd**	1.775	1.808	1.888	5.126	1.026	2.245	5.613	11.0	1.250	1.236
$S_0^{'}$	**Qa**	1.851	1.785	1.936	1.938	1.020	2.381	3.572	-133.8	1.237	1.219
	**Qb**	1.786	1.781	1.928	1.953	1.025	2.313	4.440	-138.7	1.239	1.218
	**Qc**	1.824	1.790	1.934	1.943	1.018	2.355	5.177	59.8	1.238	1.221
	**Qd**	1.808	1.789	1.932	1.941	1.022	2.320	5.540	19.8	1.238	1.218

Table S4 Mulliken charges of the isoalloxazine moiety of the EL222, AsLOV2 and RsLOV cluster models **Qa-Qd** along the reaction coordinate.

		S_0	S_1	T_1	$T_{1\text{-}H}$	$S_0^{'}$
EL222	**Qa**	0.031	-0.050	0.032	0.037	-0.010
	**Qb**	-0.019	-0.017	-0.021	-0.008	-0.050
	**Qc**	-0.016	-0.700	-0.019	-0.081	-0.086
	**Qd**	-0.018	-0.017	-0.021	0.000	-0.045
AsLOV2	**Qa**	0.081	0.142	0.055	0.080	-0.036
	**Qb**	0.049	0.133	0.029	0.072	-0.051
	**Qc**	0.002	0.006	-0.012	0.088	-0.053
	**Qd**	0.020	0.057	0.013	0.081	-0.045
RsLOV	**Qa**	-0.014	0.046	-0.042	-0.051	-0.121
	**Qb**	-0.044	0.042	-0.065	-0.076	-0.086
	**Qc**	-0.053	0.035	-0.074	-0.039	-0.086
	**Qd**	-0.038	0.038	-0.066	-0.049	-0.113

2a. Vibrational Data (Non-Deuterated Spectra)
A brief description of the computed spectra not discussed in the main text is given here:

EL222: The **Qc** S_1 of EL222 was established as a CT state so it is not discussed in the main text. Due to the flip of the Q138 side chain, the carbonyl stretch is blueshifted to 1698 cm^{-1}, while the amide peak, uncoupled to other modes, is found at 1553 cm^{-1} (non-deuterated value). When glutamine tautomerises, as in **Qb**, a unique weak-to-medium shoulder appears at 1664 cm^{-1} due to the C=N stretch, and another weak peak at 1259 cm^{-1} due to the H-rocking of the HOCNH (DOCND) imidic moiety which is downshifted due to the deuteration (Table S7). Similarly, in **Qd**, the former appears at 1658 cm^{-1} with weaker intensity, displaying also sensitivity to the side chain rotation, and the latter at 1242 cm^{-1} with matching intensity. A unique feature of the non-deuterated $T_{1\text{-}H}$ spectra (and the subsequent adduct S_0') is the N_5-H bending mode, which appears throughout the fingerprint region, from 1600 cm^{-1} down to 1390 cm^{-1} coupled to other modes. This mode downshifts below 1350 cm^{-1} in deuterium. Adduct formation (and H-transfer) can be traced by the appearance of a negative peak in the transparent window, due to the disappearance of the S-H(D) mode of cysteine (~2600 cm^{-1}). It was shown that, leading to the H-transfer TS, the peak can shift to as low a wavenumber as 1800 cm^{-1}. (S_1 **Qc**). These peaks are expected to downshift by 800 cm^{-1} in deuterium.

AsLOV2: In the AsLOV2 S1 **Qc** spectrum, the side chain glutamine carbonyl stretch is predicted at 1691 cm^{-1} with medium intensity decoupled from the ND_2 mode, which downshifts to 1115 cm^{-1} with weak intensity. With regards to the imidic isomers, **Qb** displays a weak C=N stretch at 1666 cm^{-1} and **Qd** at 1661 cm^{-1} with medium intensity (Table S7). The HNCOH bending modes span 1360-1380 cm^{-1} with weak intensity for all species up to and including the adduct and redshift substantially upon deuteration. In the adduct and $T_{1\text{-}H}$ spectra, the bending mode of the newly formed N_5-H is registered in many different vibrations, as in EL222, spanning the whole 1350-1750 cm^{-1} region. As mentioned above, upon deuteration, these uncouple from fingerprint vibrations and shift to the red of the studied region.

RsLOV: Compared to EL222 and AsLOV2, unique weak-to-medium peaks arise from the hydroxy group of the additional threonine residue at 1650 cm^{-1} coupled to the adjacent backbone C=O stretch, and at 1390 cm^{-1} of weak relative intensity. These are predicted to downshift below 1350 cm^{-1} in D_2O.

Overall, imidic tautomerisation peaks include C=N stretching and HOCNH bending modes. Both are of weak intensity and are located around 1680 and 1360 cm^{-1}, respectively with the latter expected to disappear from the fingerprint region in D_2O. Thus, possible tautomerisation could be better tracked indirectly by the disappearance of the glutamine carbonyl and amide modes. Amide labelling and double difference spectra are required to reveal imidic vibrations in the experimental spectra, as was shown in BLUF.

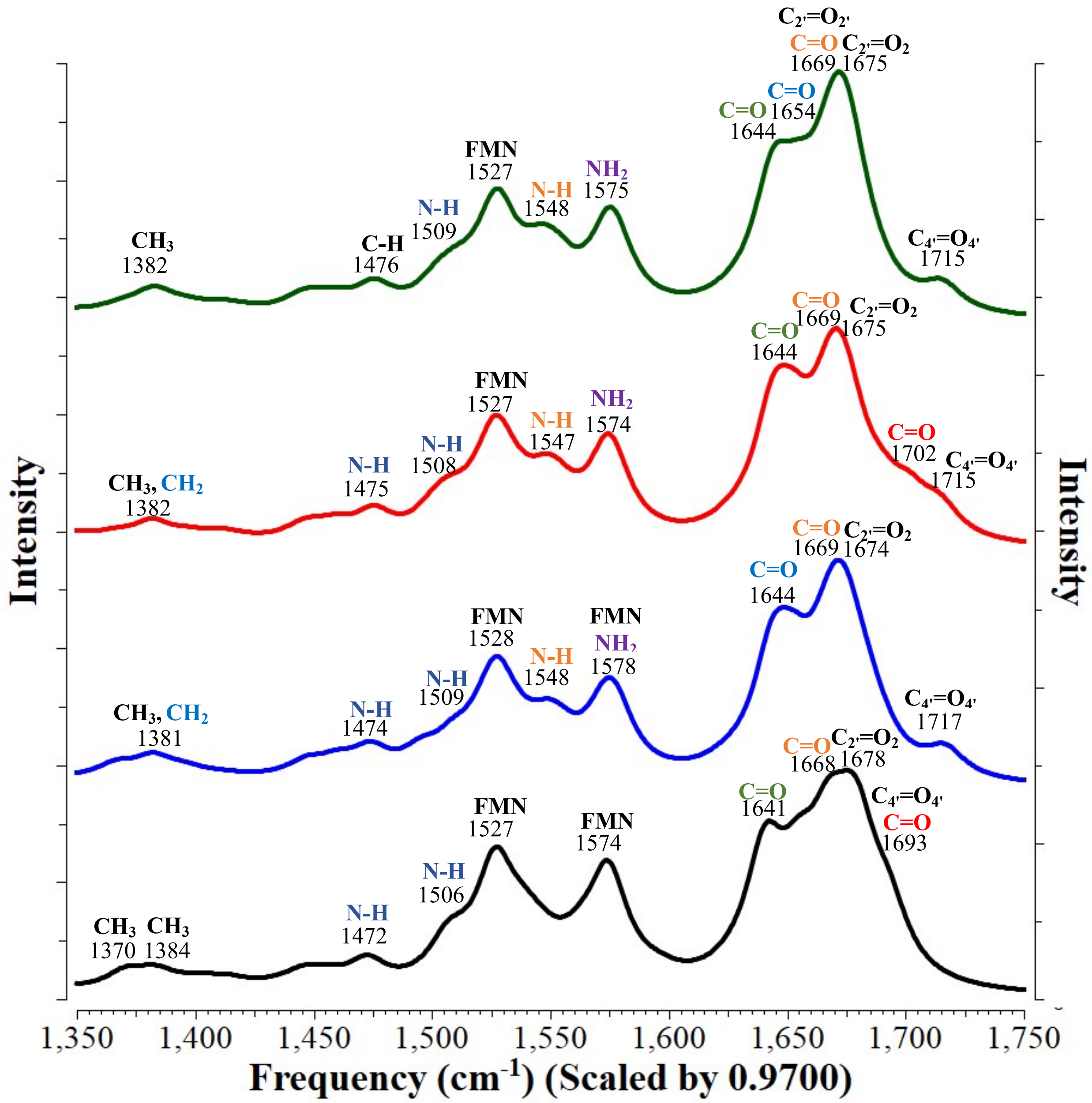

Figure S13 Calculated non-deuterated infrared spectra of the ground state S_0 of the EL222 cluster. The spectra of the amidic Q138 models **Qa** and **Qc** are shown with black and red lines, and of the imidic isomers **Qb** and **Qd** with blue and green, respectively. Vibrations assigned to FMN normal modes are labelled black, to Q138 red, to C75 orange, to N107 green, to N117 violet and to other backbone modes, blue. All spectra were drawn with a half-width at half-height maximum (HWHM) of 8 cm^{-1}.

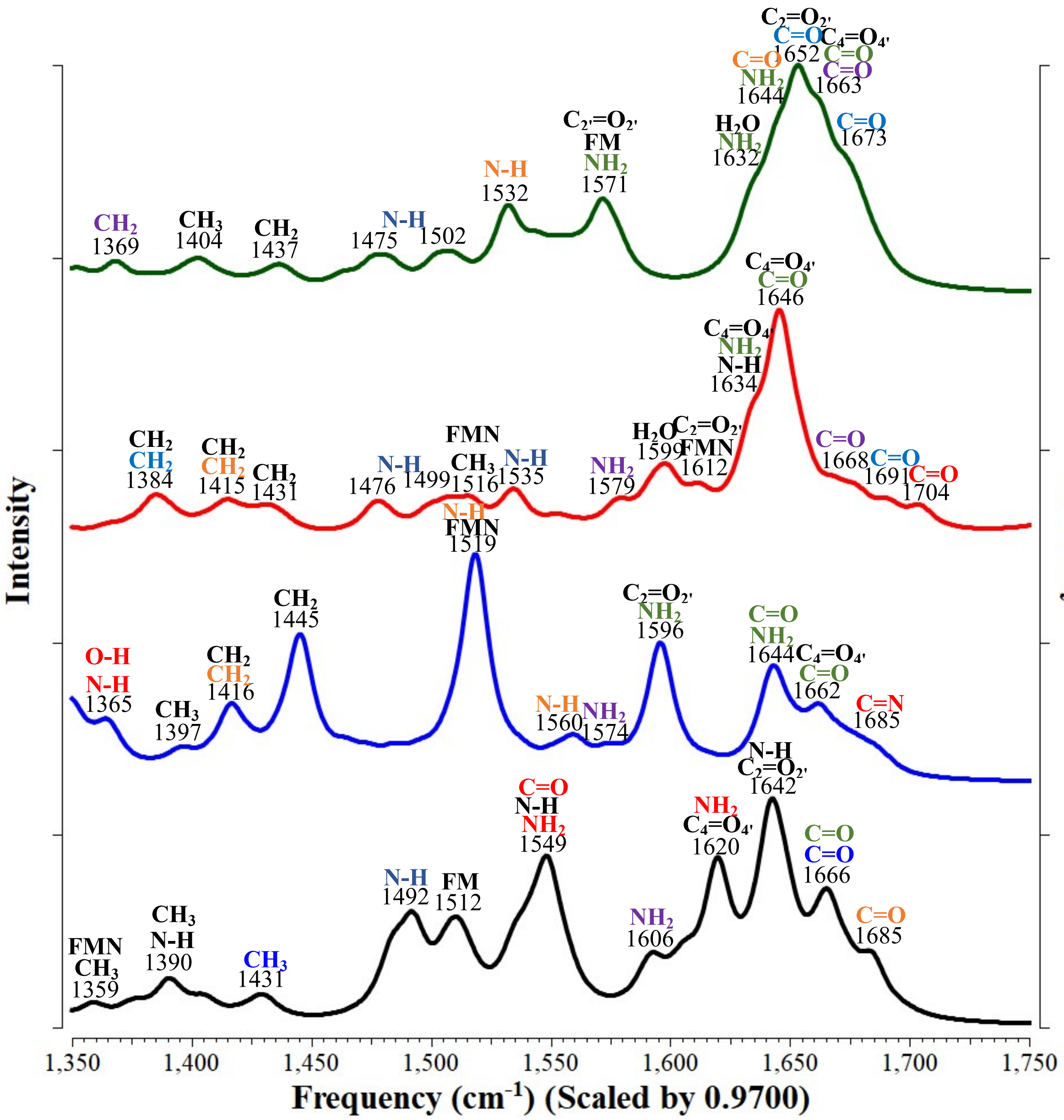

Figure S14 Calculated non-deuterated infrared spectra of the S_1 state of the EL222 cluster. The spectra of amidic Q138 models **Qa** and **Qc** are shown with black and red lines, and of the imidic isomers **Qb** and **Qd** with blue and green, respectively. Vibrations assigned to FMN normal modes are labelled black, to Q138 red, to C75 orange, to N107 green, to N117 violet and to other backbone modes, blue. All spectra were drawn with a half-width at half-height maximum (HWHM) of 8 cm^{-1}.

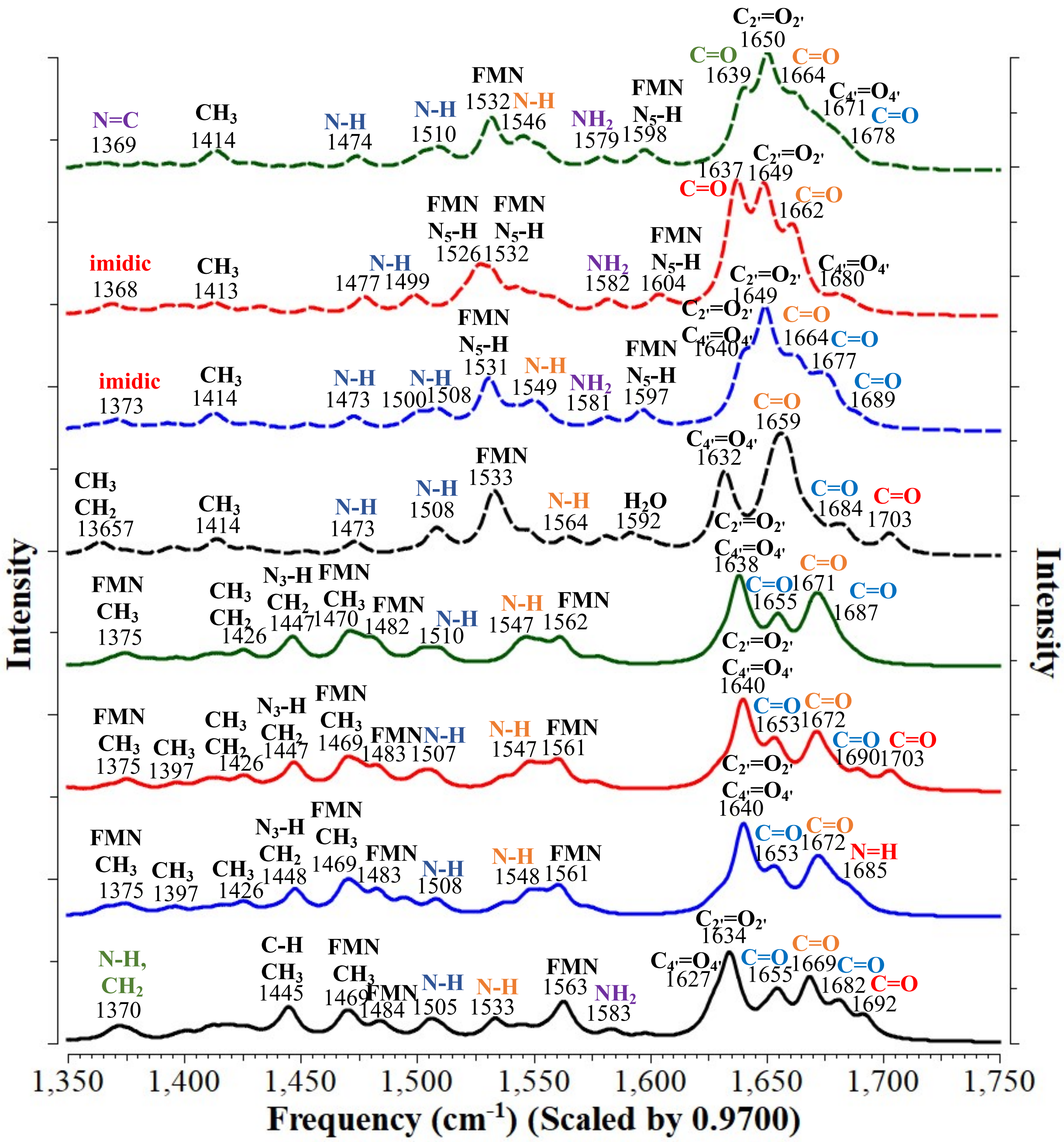

Figure S15 Calculated non-deuterated infrared spectra of the T_1 (solid lines) and $T_{1\text{-}H}$ states (dashed lines) of the EL222 cluster. The spectra of the amidic Q138 models **Qa** and **Qc** are shown with black and red lines, and of the imidic isomers **Qb** and **Qd** with blue and green, respectively. Vibrations assigned to FMN normal modes are labelled black, to Q138 red, to C75 orange, to N107 green, to N117 violet and to other backbone modes, blue. All spectra were drawn with a half-width at half-height maximum (HWHM) of 8 cm^{-1}.

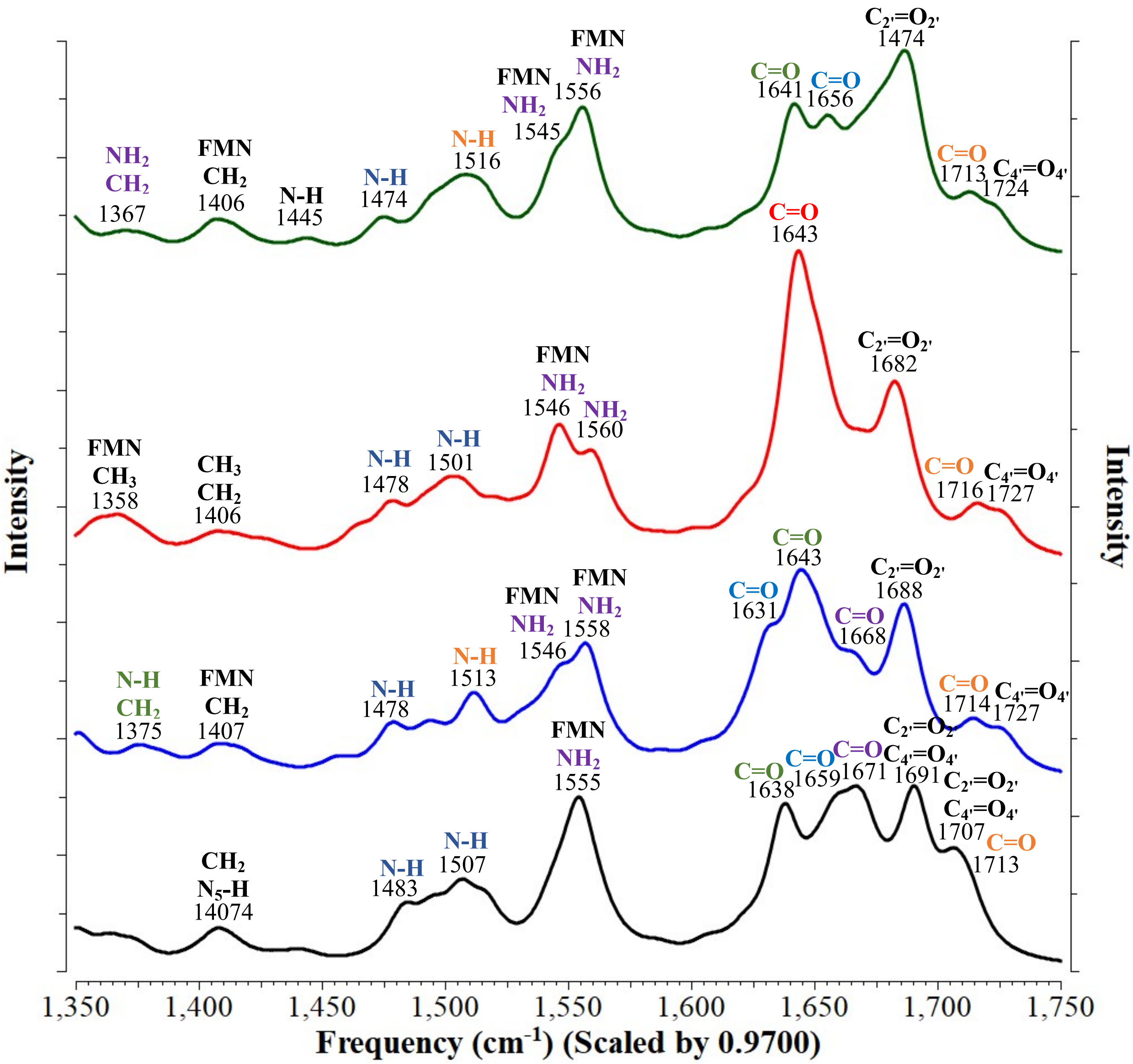

Figure S16 Calculated non-deuterated infrared spectra of the adduct state $S_0^{'}$ of the EL222 cluster. The spectra of amidic Q138 models **Qa** and **Qc** are shown with black and red lines, and of the imidic isomers **Qb** and **Qd** with blue and green, respectively. Vibrations assigned to FMN normal modes are labelled black, to Q138 red, to C75 orange, to N107 green, to N117 violet and to other backbone modes, blue. All spectra were drawn with a half-width at half-height maximum (HWHM) of 8 cm^{-1}.

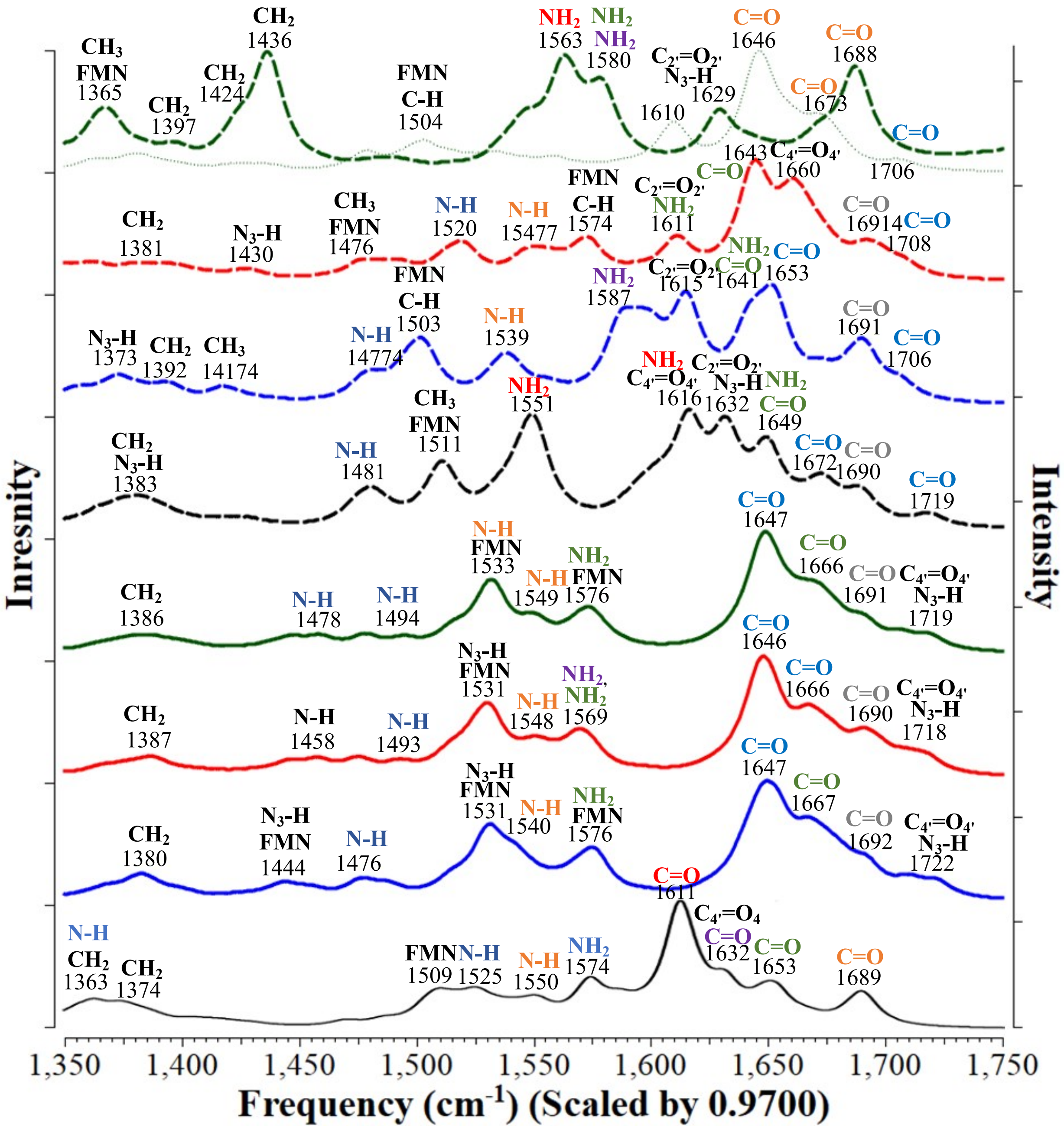

Figure S17 Calculated non-deuterated infrared spectra of the S_0 (solid lines) and S_1 states (dashed lines) of the AsLOV2 cluster. The spectra of the amidic Q513 models **Qa** and **Qc** are shown with black and red lines, and of the imidic isomers **Qb** and **Qd** with blue and green, respectively. The infrared spectrum of the second ππ* excitation of **Qd** is also included with a green dotted line. Vibrations assigned to FMN normal modes are labelled black, to Q513 red, to C450 orange, to N482 green, to N492 violet, to Q454 grey and to other backbone modes, blue. All spectra were drawn with a half-width at half-height maximum (HWHM) of 8 cm^{-1}.

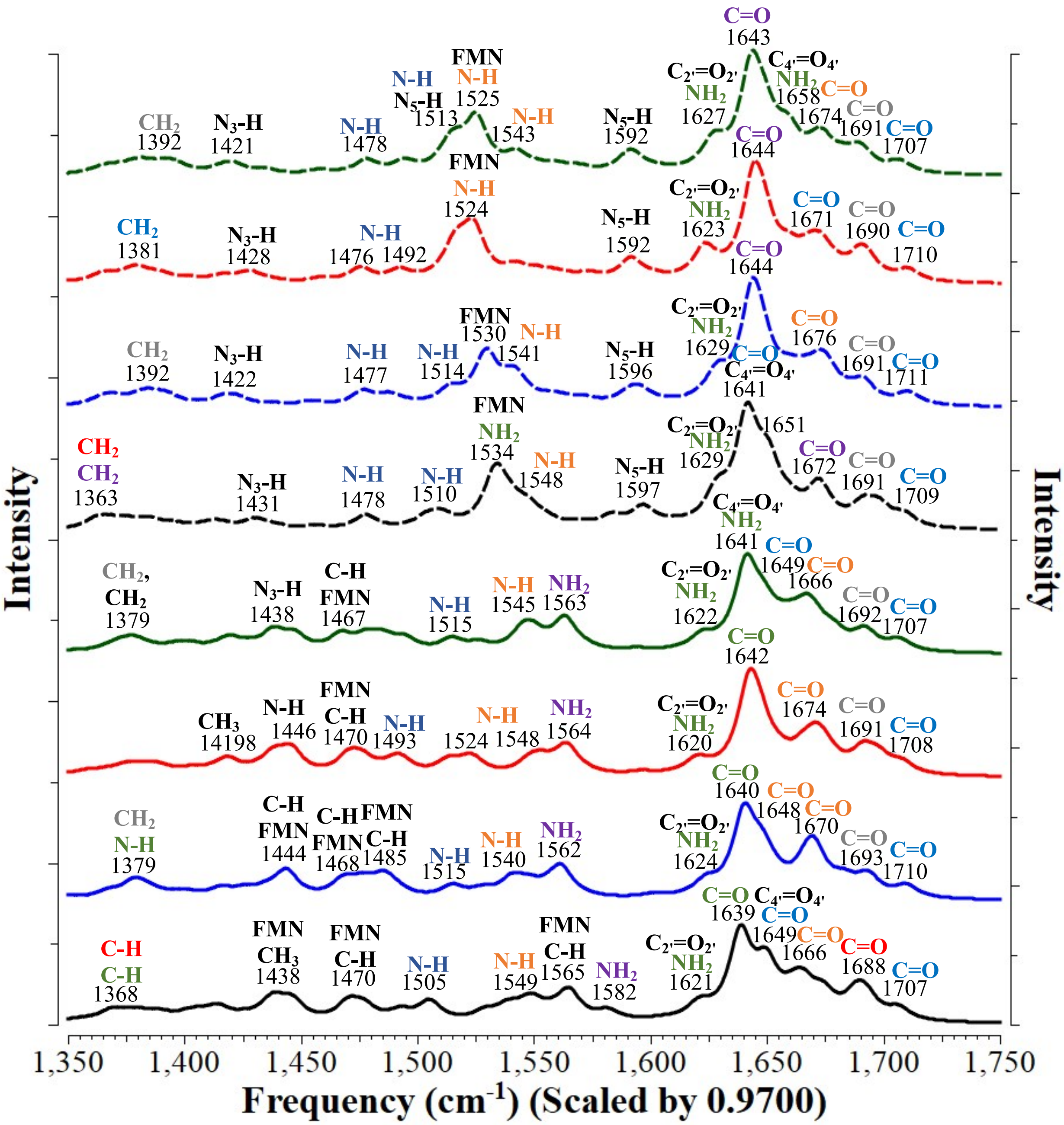

Figure S18 Calculated non-deuterated infrared spectra of the T_1 (solid lines) and $T_{1\text{-}H}$ states (dashed lines) of the AsLOV2 cluster. The spectra of the amidic Q513 models **Qa** and **Qc** are shown with black and red lines, and of the imidic isomers **Qb** and **Qd** with blue and green, respectively. Vibrations assigned to FMN normal modes are labelled black, to Q513 red, to C450 orange, to N482 green, to N492 violet, to Q454 grey and to other backbone modes, blue. All spectra were drawn with a half-width at half-height maximum (HWHM) of 8 cm^{-1}.

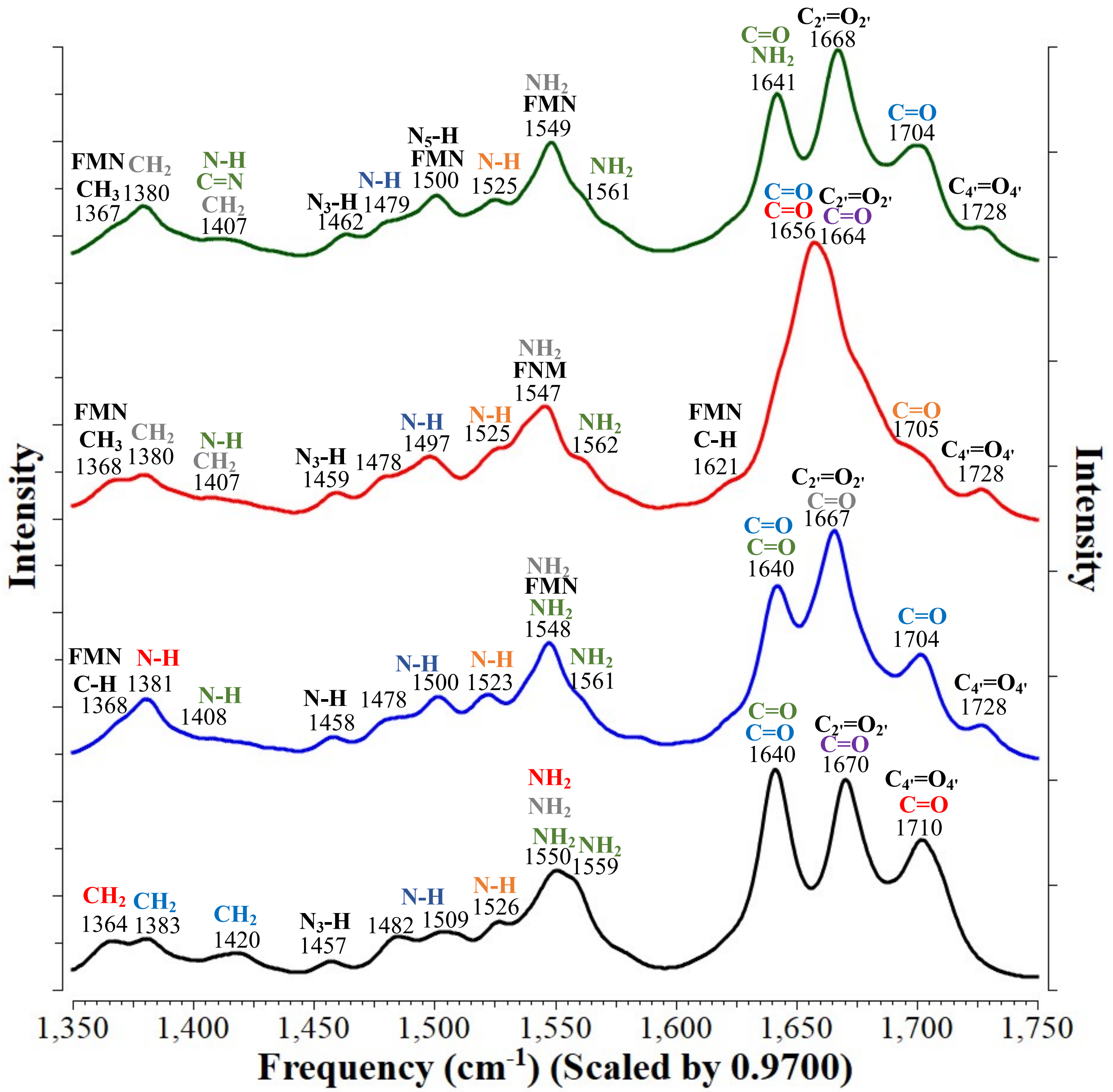

Figure S19 Calculated non-deuterated infrared spectra of the adduct state (S'_0) of the AsLOV2 cluster. The spectra of the amidic Q513 models **Qa** and **Qc** are shown with black and red lines, and of the imidic isomers **Qb** and **Qd** with blue and green, respectively. Vibrations assigned to FMN normal modes are labelled black, to Q513 red, to C450 orange, to N482 green, to N492 violet, to Q454 grey and to other backbone modes, blue. All spectra were drawn with a half-width at half-height maximum (HWHM) of 8 cm^{-1}.

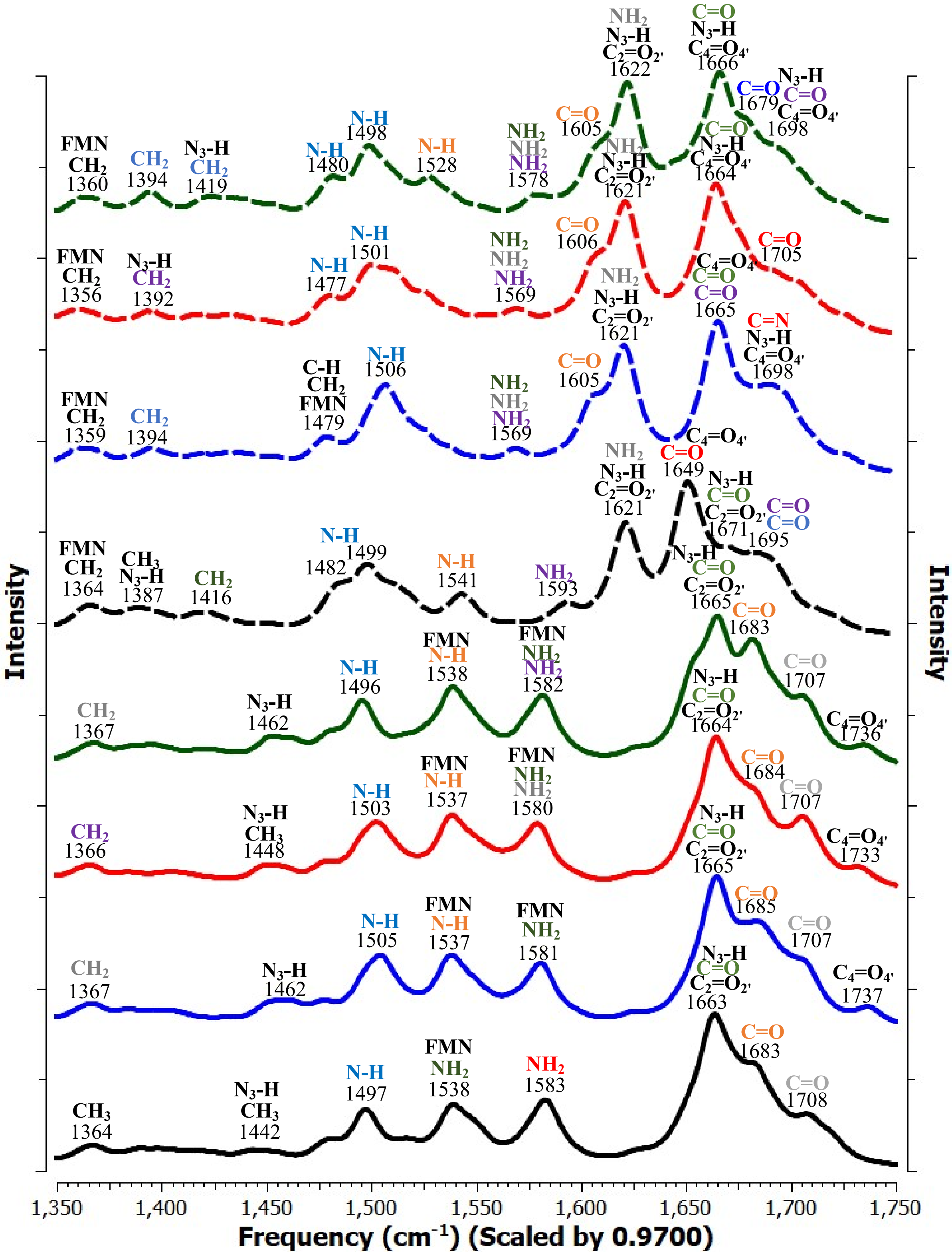

Figure S20 Calculated non-deuterated infrared spectra of the S_0 (solid) and S_1 states (dashed) lines of the RsLOV cluster. The spectra of the amidic Q118 models **Qa** and **Qc** are shown with black and red, and of the imidic isomers **Qb** and **Qd** with blue and green, respectively. Vibrations assigned to FMN normal modes are labelled black, to Q118 red, to C55 orange, to N87 green, to N97 violet, to Q59 grey, to T21 yellow, and to other backbone modes, blue. All spectra were drawn with a half-width at half-height maximum (HWHM) of 8 cm^{-1}.

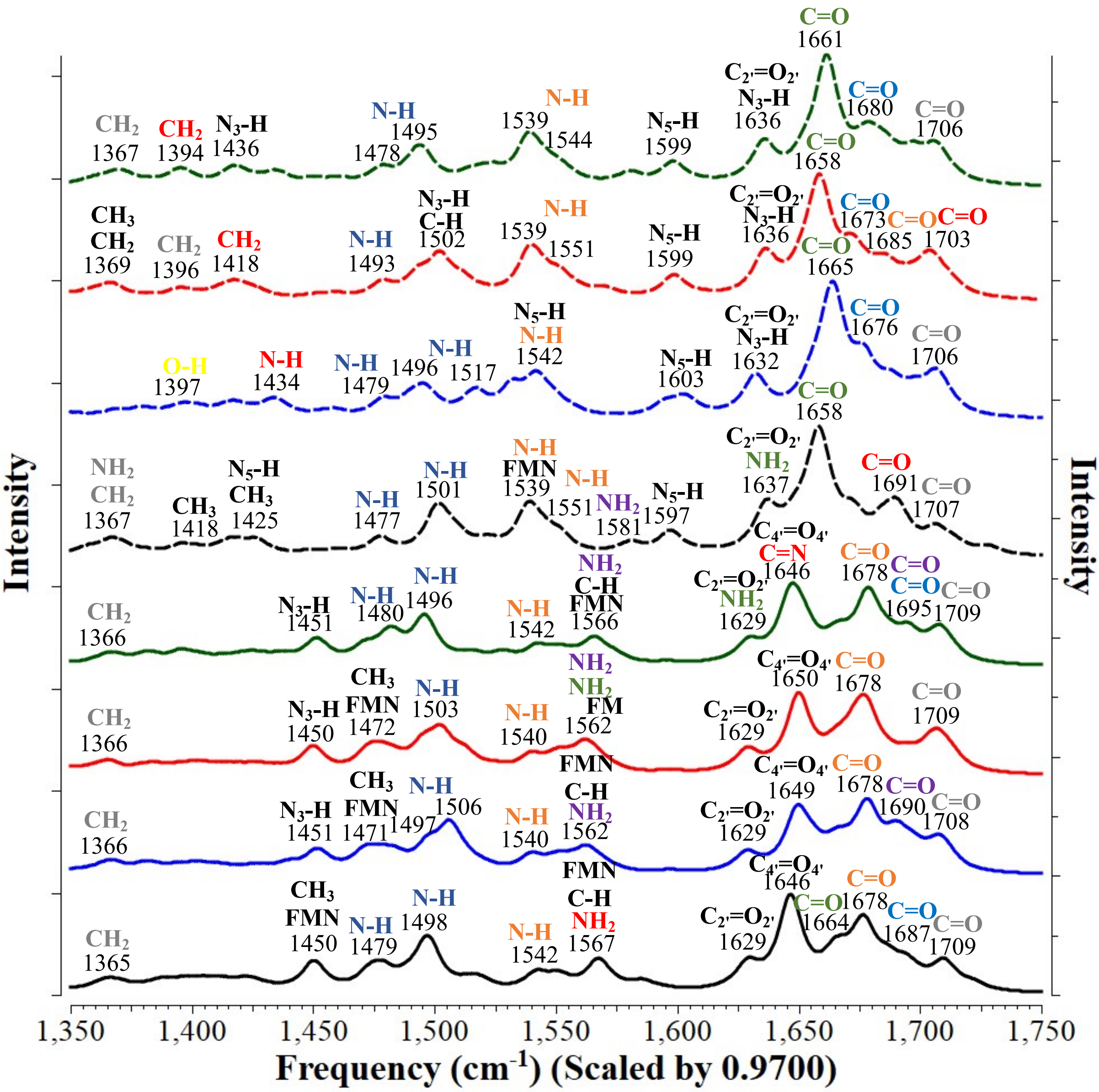

Figure S21 Calculated non-deuterated infrared spectra of the T_1 (solid lines) and T_1-H states (dashed lines) of the RsLOV cluster. The spectra of the amidic Q118 models **Qa** and **Qc** are shown with black and red lines, and of the imidic isomers **Qb** and **Qd** with blue and green, respectively. Vibrations assigned to FMN normal modes are labelled black, to Q118 red, to C55 orange, to N87 green, to N97 violet, to Q59 grey, to T21 yellow, and to other backbone modes, blue. All spectra were drawn with a half-width at half-height maximum (HWHM) of 8 cm^{-1}.

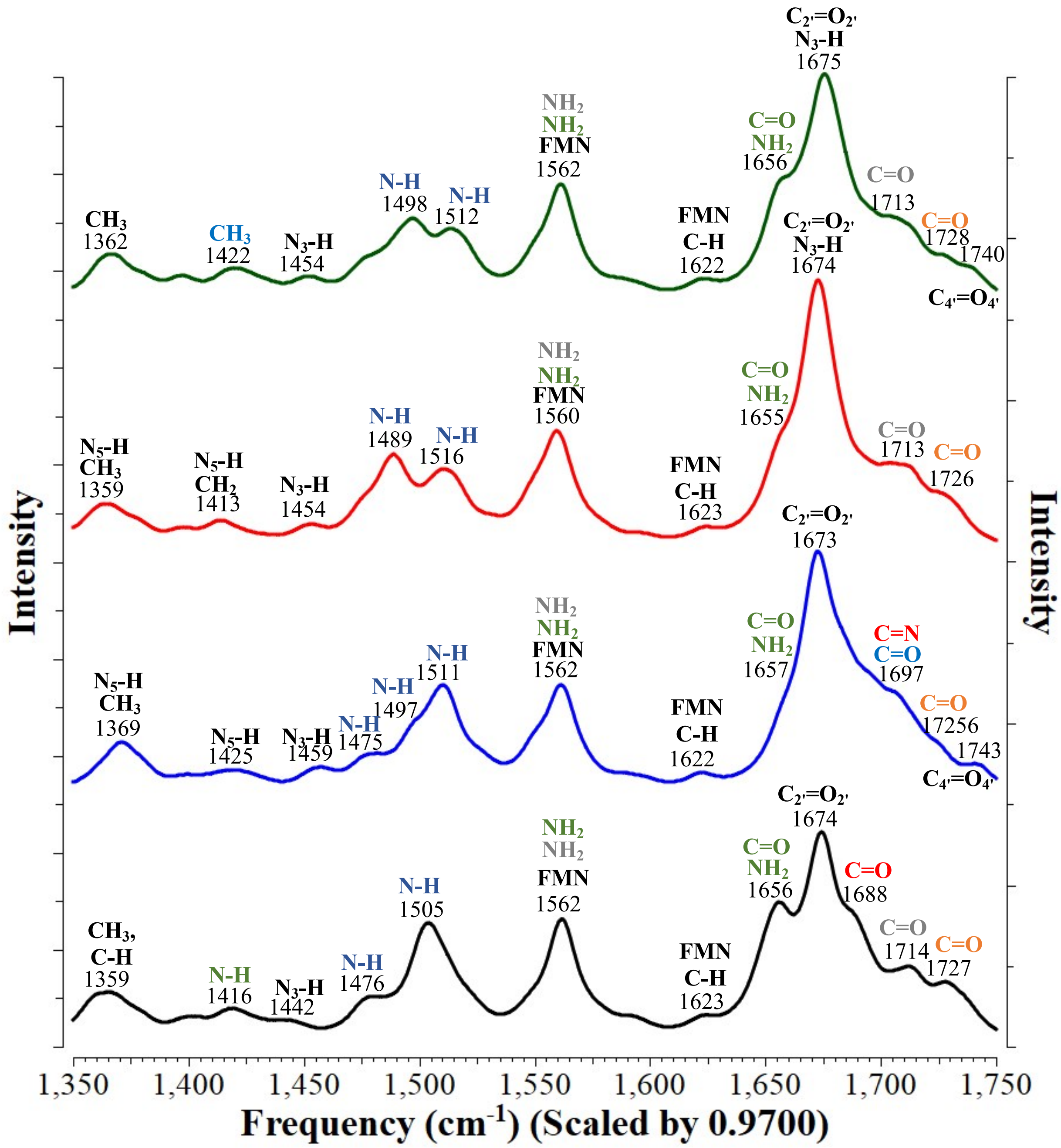

Figure S22 Calculated non-deuterated infrared spectra of the adduct state (S'_0) of the RsLOV cluster. The spectra of the amidic Q118 models **Qa** and **Qc** are shown with black and red lines, and of the imidic isomers **Qb** and **Qd** with blue and green, respectively. Vibrations assigned to FMN normal modes are labelled black, to Q118 red, to C55 orange, to N87 green, to N97 violet, to Q59 grey, to T21 yellow, and to other backbone modes, blue. All spectra were drawn with a half-width at half-height maximum (HWHM) of 8 cm^{-1}.

Table S5 Assignment Table for major vibrations of the EL222 cluster. Values for the non-deuterated spectra in cm^{-1}.

		C=O	C=O	C=N	CHNH	C=O		C=O	$C_{4'}=O_{4'}$	$C_{2'}=O_{2'}$	C=O	NH_2	NH_2	NH_2	N_5-H	N_3-H	N-H		N-H	CH_3	CH_2	C-H	FMN	H_2O
**EL222**	S_0 **Qa**	1641	1693	-	-	1683	1655	1666	1693	1678	1668<br>1639	1641<br>1550	1584	1574<br>1560	-	1678<br>1457	1506	1472	1563<br>1538	1447<br>1384	1370	1474	1574<br>1527	1596
	S_1 **Qa**	1662<br>1648<br>1554	1549	-	-	1674<br>1666<br>1659	1698	1662	1620<br>1588	1642	1684<br>1635	1648<br>1554	1606<br>1592<br>1588	1620<br>1606	-	1642<br>1406<br>1390	1516<br>1507<br>1492	1483	1557<br>1542<br>1535	1431<br>1406<br>1390	1431<br>1406	1438	1512<br>1484	1601
	T_1 **Qa**	1650	1692	-	-	1682	1655	1673<br>1668	1650<br>1627	1634<br>1627	1669<br>1638	1627<br>1550	1583	1556	-	1634<br>1484	1545	1505	1563<br>1533	1427<br>1420	1445<br>1370	1563<br>1469	1563<br>1484	1598
	T_{1-H} **Qa**	1650<br>1632	1703	-	-	1684	1655	1668	1650<br>1632	1659<br>1654	1659<br>1637	1632<br>1533	1581<br>1550	1568	1600<br>1514	1654<br>1452	1547	1508	1564<br>1537	1426<br>1414	1473<br>1365	1495<br>1452	1533<br>1452	1600<br>1592
	S'_0 **Qa**	1638	1711<br>1707	-	-	1671	1659	1671	1714<br>1707	1707<br>1691	1713<br>1689	1638<br>1555	1587<br>1543	1563	1621<br>1573	1691<br>1443	1507	1483	1517<br>1495	1374<br>1364	1413<br>1407	1621<br>1496	1621<br>1555	1607
	S_0 **Qb**	1644	-	1684	1366	1689<br>1679	1676<br>1653	1665	1717	1674<br>1665	1669<br>1644	1644<br>1578	1578<br>1573	-	-	1717<br>1674<br>1665<br>1460	1537<br>1509	1495	1644<br>1553<br>1548<br>1474	1447	1381	1475	1578<br>1527<br>1521	1620
	S_1 **Qb**	1644<br>1653	-	1685	1366	1690	1679	1644	1674<br>1662	1596	1669<br>1641	1644<br>1596	1574<br>1556	-	-	1662<br>1464	1537	1495	1560<br>1519	1425<br>1365	1445<br>1416	1485	1485<br>1464	1616
	T_1 **Qb**	1640<br>1656	-	1685	1365	1653		1670	1640<br>1656	1629	1644<br>1672<br>1676<br>1679<br>1690	1629<br>1656	1574	-	-	1629<br>1640<br>1483<br>1448	1536<br>1508	1495<br>1474	1552<br>1548	1469<br>1425<br>1397	1425<br>1375	1561<br>1469	1561<br>1494<br>1483<br>1469	1627
	T_{1-H} **Qb**	1640<br>1655	-	1673<br>1677	1373	1655<br>1678	1689	1671	1660<br>1671	1649	1644<br>1664	1640	1581	-	1597<br>1512	1649	1541<br>1508	1500<br>1473	1644<br>1553<br>1549	1414<br>1391	1412<br>1370	1607<br>1453	1607<br>1531	1618
	S'_0 **Qb**	1643	-	1663	1386	1630<br>1651	1655	1668	1727	1686<br>1688	1714<br>1643	1643<br>1558<br>1546	1588	-	1485	1727<br>1688	1478	1456	1513<br>1510<br>1493	1407<br>1351	1407<br>1375	1621<br>1497	1621<br>1497<br>1351	1605
	S_0 **Qc**	1644	1702	-	-	1653<br>1673	1679<br>1690	1666	1715	1666<br>1673<br>1675	1644<br>1669	1644	1578<br>1574	1549	-	1715<br>1675<br>1459	1536<br>1508	1501	1669<br>1553<br>1547<br>1475	1447	1382<br>1367	1624<br>1476	1527<br>1520	1627
	S_1 **Qc**	1646	1704	-	-	1653<br>1675	1679<br>1691	1668	1656	1634	1612<br>1643	1634<br>1646<br>1656<br>1552	1579	1553	-	1634<br>1646<br>1656	1533<br>1507	1499<br>1476	1643<br>1577<br>1535	1516<br>1437<br>1391	1431<br>1414<br>1384	1480<br>1437	1516<br>1480	1599<br>**SH:**<br>**1612**
	T_1 **Qc**	1640	1703	-	-	1653<br>1675	1679<br>1690	1670	1640<br>1656	1629<br>1640	1644<br>1672	1629<br>1656	1577	1549	-	1629<br>1640<br>1483<br>1447	1536<br>1507	1501<br>1475	1644<br>1672<br>1553<br>1547	1469<br>1426	1447<br>1426<br>1408<br>1375	1561	1561<br>1483<br>1469<br>1375	1625
	T_{1-H} **Qc**	1649<br>1652	1637	-	-	1686<br>1659	1686	1680<br>1665	1680<br>1665	1635<br>1649	1642<br>1662	1635<br>1652	1582	1637<br>1558	1604<br>1532<br>1526	1680<br>1635<br>1455<br>1433	1527<br>1519	1499<br>1477	1551<br>1543	1413<br>1391<br>1371	1400<br>1368	1497<br>1455	1526<br>1455	1644
	S'_0 **Qc**	1644	1643<br>1654<br>1661	-	-	1652<br>1654	1661<br>1687	1669	1727	1682	1684<br>1716	1643<br>1644<br>1560<br>1546	1586	1654<br>1661<br>1562	1571<br>1507	1682<br>1727<br>1464	1530<br>1520	1501<br>1478	1509<br>1507<br>1491	1358<br>1378<br>1406	1408<br>1369	1621<br>1496	1621<br>1571<br>1546	1601
	S_0 **Qd**	1644	-	1681	1387	1655<br>1674	1678<br>1687	1665	1716	1675	1645<br>1669	1644<br>1555	1575<br>1578	-	-	1716<br>1458<br>1447	1543<br>1510	1502	1553<br>1548	1447<br>1382	1409<br>1382	1625<br>1476	1625<br>1528<br>1521	1638
	S_1 **Qd**	1652<br>1655	-	1679<br>1681	1386	1655<br>1663<br>1673	1678<br>1687	1652<br>1663	1663<br>1681	1652	1644<br>1652<br>1669	1571<br>1655<br>1554	1578	-	-	1663<br>1679<br>1681<br>1463	1687<br>1544<br>1510	1502<br>1475	1561<br>1554<br>1551<br>1532	1463<br>1404<br>1400<br>1352	1437<br>1369<br>1352	1483	1483<br>1463	1632<br>1636
	T_1 **Qd**	1655	-	1680	1386	1655<br>1674	1678	1669	1638	1630	1636<br>1644<br>1671	1562<br>1630<br>1638<br>1655	1578	-	-	1630<br>1638<br>1655<br>1482	1674<br>1543<br>1510	1502<br>1475	1644<br>1553<br>1547<br>1375	1470<br>1426<br>1397<br>1375	1447<br>1426<br>1408	1562<br>1470	1562<br>1482<br>1470	1636
	T_{1-H} **Qd**	1639<br>1655	-	1683<br>1688	1381	1655<br>1675	1678<br>1688	1671	1659<br>1671	1650	1644<br>1664	1639<br>1655<br>1659	1579	-	1598<br>1513	1650<br>1659<br>1671	1545<br>1510	1503<br>1474	1553<br>1546	1453<br>1414<br>1359<br>1393	1414<br>1381	1496<br>1453	1532<br>1453	1704
	S'_0 **Qd**	1641	-	1684<br>1687	1381	1656<br>1674	1679	1668	1724	1687<br>1689	1687<br>1713	1641<br>1556<br>1544	1586	-	1571<br>1481	1689<br>1445	1545<br>1509	1503<br>1474	1516<br>1494	1374<br>1406	1441<br>1406<br>1367	1497	1621<br>1571<br>1556	1606

Table S6 Assignment Tables for major vibrations of the AsLOV2 and RsLOV clusters. Values for the non-deuterated spectra in cm^{-1}.

		C=O	C=O	C=N	CHNH	C=O	C=O	$C_{4'}=O_{4'}$	$C_{2'}=O_{2'}$	C=O	C=O	NH_2	NH_2	NH_2	NH_2	N_5-H	N_3-H	N-H	N-H	CH_3	CH_2	C-H	O-H	FMN	H_2O
AsLOV2	S_0 **Qa**	1639	1690	-	-	1650<br>1661<br>1673<br>1674<br>1676<br>1707	1671<br>1676	1703	1656	1653<br>1671<br>1673	1692	1545	1575<br>1639	1584	1575<br>1563	-	1656<br>1703<br>1463<br>1448	1529<br>1506<br>1477	1550<br>1539	1448<br>1403<br>1386	1434<br>1403<br>1389<br>1386<br>1368	1626	-	1575<br>1626<br>1532	1602
	S_1 **Qa**	1653<br>1667	1611	-	-	1619<br>1647<br>1655<br>1691	1667	1631	1613	1648<br>1689	1689	1537<br>1689	1653	1667	1611	-	1414<br>1543<br>1613<br>1632	1363<br>1470<br>1504<br>1517<br>1525	1532	1430<br>1486<br>1509	1363<br>1382<br>1404	1441<br>1486<br>1509	-	1486<br>1509<br>1537<br>1594	1598
	T_1 **Qa**	1650	1688	-	-	1649<br>1661<br>1674<br>1707	1674<br>1676	1650	1621	1653<br>1666	1693	1545	1621<br>1639<br>1649<br>1650	1582	1558	-	1621<br>1639<br>1650<br>1493<br>1446<br>1438	1529<br>1505<br>1477	1549<br>1539	1493<br>1438<br>1415	1438<br>1415<br>1403<br>1389<br>1368	1565<br>1470	-	1565<br>1493<br>1470	1605
	$T_{1\text{-}H}$ **Qa**	1641	1698	-	-	1660<br>1672<br>1674<br>1677<br>1709	1672	1651<br>1652	1629<br>1641	1647	1691	1546<br>1558	1558<br>1629<br>1641<br>1651<br>1652	1583	1570	1597	1641<br>1651<br>1652<br>1431	1531<br>1510<br>1504<br>1478	1540<br>1548<br>1647	1415<br>1395	1409<br>1386<br>1382<br>1363	1609	-	1534<br>1597<br>1609	1598
	S_0' **Qa**	1640<br>1642	1710<br>1715	-	-	1672<br>1674<br>1702	1679	1710<br>1715	1670<br>1679	1689<br>1703	1698	1537<br>1550	1559<br>1640<br>1642	1579<br>1581	1545<br>1550	1581<br>1499	1670<br>1457	1646<br>1509<br>1501<br>1482	1526<br>1487	1435<br>1424<br>1381	1425<br>1409<br>1383<br>1364	1621<br>1494	-	1581<br>1621	1610
	S_0 **Qb**	1640	-	1682<br>1683	1366	1647<br>1666<br>1671<br>1682<br>1710	1667	1722	1654	1648<br>1676	1692	1531<br>1544	1562<br>1569<br>1576<br>1640	1562<br>1569<br>1576	-	-	1531<br>1654<br>1455<br>1444	1528<br>1515<br>1488<br>1476	1540<br>1548	1476<br>1444<br>1425<br>1419<br>1356	1438<br>1385<br>1383<br>1380	1625<br>1476	-	1531<br>1576<br>1625<br>1521<br>1476	1598
	S_1 **Qb**	1641	-	1687	1368	1706<br>1672<br>1653	1599	1649	1615	1691<br>1644	1691	1534	1641<br>1615<br>1557	1649<br>1599<br>1593<br>1587	-	-	1649<br>1615<br>1373	1504<br>1499<br>1495<br>1477	1543<br>1539	1356	1417<br>1395<br>1392<br>1381	1593<br>1503<br>1483	-	1593<br>1534<br>1503<br>1483<br>1356	1604
	T_1 **Qb**	1640<br>1654	-	1684	1366	1648<br>1666<br>1671<br>1682<br>1710	1669	1640<br>1654	1624	1648<br>1670	1693	1547	1624<br>1640<br>1654	1562	-	-	1624<br>1640<br>1654<br>1444<br>1435	1515<br>1528<br>1488<br>1476	1540<br>1548	1493<br>1468<br>1426<br>1416	1444<br>1426<br>1379	1562<br>1468	-	1562<br>1493<br>1485	1601
	$T_{1\text{-}H}$ **Qb**	1644	-	1675	1358<br>1367	1649<br>1666<br>1671<br>1683<br>1711	1659<br>1673	1659<br>1673	1629	1646<br>1676	1691	1540	1562<br>1572<br>1629<br>1644<br>1649	1562<br>1572	-	1596<br>1512	1629<br>1644<br>1659<br>1673<br>1454<br>1422	1528<br>1514<br>1488<br>1477	1541<br>1546	1454<br>1416<br>1363	1392<br>1385<br>1383<br>1363	1607<br>1454	-	1530<br>1596<br>1607<br>1454	1591
	S_0' **Qb**	1643	-	1657	1381<br>1382	1640<br>1664<br>1673<br>1679	1677<br>1679<br>1704	1728	1667	1687<br>1703	1698	1538<br>1548	1561<br>1643	1585	-	1505<br>1436	1667<br>1458	1518<br>1504<br>1500	1523<br>1487<br>1478	1436<br>1381<br>1368	1436<br>1408<br>1375	1622	-	1548<br>1622	1603
	S_0 **Qc**	1651	1697	-	-	1646<br>1666<br>1671<br>1678<br>1709	1667<br>1690	1718	1638<br>1651	1647<br>1677	1690	1552<br>1562	1569<br>1574<br>1638<br>1651	1562<br>1569<br>1574	1553	-	1638<br>1651<br>1458<br>1445	1514<br>1522<br>1493<br>1475	1526<br>1548	1445<br>1390	1390<br>1387<br>1386<br>1379<br>1332	1627	-	1531<br>1569<br>1627<br>1514	1596
	S_1 **Qc**	1643	1697	-	-	1647<br>1665<br>1671<br>1678<br>1708	1660<br>1664<br>1675	1660<br>1675	1611	1646<br>1660<br>1664	1691	1547<br>1569	1569<br>1611<br>1643	1558	1553	-	1611<br>1660<br>1675	1512<br>1520<br>1493<br>1476	1520<br>1547	1399<br>1361<br>1348	1381<br>1377<br>1361	1574<br>1486	-	1574<br>1486<br>1399<br>1361	1594
	T_1 **Qc**	1647<br>1655	1698	-	-	1647<br>1665<br>1671<br>1678<br>1708	1670	1642<br>1655	1620	1647<br>1674	1691	1551<br>1561	1561<br>1571<br>1620<br>1642<br>1647<br>1655	1561<br>1564<br>1571	1553	-	1620<br>1642<br>1446<br>1438	1476<br>1493<br>1513<br>1521	1524<br>1548	1470<br>1490<br>1419	1419<br>1389<br>1382<br>1375	1470<br>1564	-	1470<br>1490<br>1564<br>1357	1596
	$T_{1\text{-}H}$ **Qc**	1644<br>1649<br>1659	1693	-	-	1649<br>1667<br>1671<br>1679<br>1711	1672	1659<br>1672	1623	1649<br>1674	1690	1551<br>1563	1563<br>1573<br>1623<br>1644<br>1649<br>1659	1563<br>1573	1553	1514<br>1592<br>1459<br>1366	1623<br>1644<br>1659<br>1672<br>1459	1476<br>1492<br>1515<br>1524	1518<br>1524<br>1542	1415<br>1366	1387<br>1376<br>1366	1459	-	1524<br>1592<br>1459	1590

	S'_0 **Qc**	1642	1650	-	-	1656<br>1663<br>1675<br>1679<br>1685	1674	1728	1664	1685<br>1705	1697	1538<br>1547	1562<br>1642	1579	1562	1503	1459<br>1664	1478<br>1497<br>1502<br>1518	1488<br>1525	1368	1407<br>1391<br>1363	1621	-	1547<br>1621	1602
	S_0 **Qd**	1639<br>1652	-	1679<br>1682	1380	1647<br>1660<br>1671<br>1679<br>1682<br>1707	1666	1719	1639<br>1652	1647<br>1673	1691	1551<br>1562	1562<br>1569<br>1576<br>1639<br>1652	1562<br>1569<br>1576	-	-	1652<br>1459<br>1447	1494<br>1515<br>1526<br>1478	1531<br>1533<br>1549<br>1363	1447<br>1386<br>1370	1438<br>1396<br>1386	1629	-	1517<br>1533<br>1629<br>1363	1587
	S_1 **Qd**	1642<br>1651	-	1679	1382	1651<br>1670<br>1672<br>1707	1660<br>1674	1660<br>1670	1629	1647<br>1688	1682	1559	1559<br>1580<br>1642	1563<br>1572<br>1580	-	-	1629<br>1422	1537<br>1549<br>1563<br>1495<br>1478	1546<br>1563	1489<br>1370<br>1363	1438<br>1436<br>1424<br>1397<br>1383	1482<br>1365	-	1489<br>1482<br>1365	1613
	S_1 **Qd2**	1644<br>1659	-	1677	1380	1650<br>1661<br>1671<br>1682<br>1706	1671	1644<br>1659<br>1671	1610	1646<br>1673	1679	1534<br>1543<br>1560	1543<br>1572<br>1610<br>1644<br>1659	1560<br>1572	-	-	1543<br>1610<br>1644<br>1671<br>1362	1495<br>1515<br>1525	1501<br>1532<br>1534<br>1646	1504<br>1534<br>1362<br>1429	1378<br>1381<br>1389<br>1398<br>1437	1478<br>1504<br>1587<br>1362<br>1378	-	1478<br>1504<br>1534<br>1587<br>1362	1601
	T_1 **Qd**	1655	-	1678<br>1681	1380	1649<br>1660<br>1671<br>1678<br>1681<br>1707	1669	1655	1622	1647<br>1666	1692	1549	1622<br>1641<br>1655	1563	-	-	1622<br>1641<br>1655<br>1438<br>1447	1478<br>1495<br>1515<br>1526	1545<br>1549	1467<br>1402<br>1419<br>1427	1369<br>1373<br>1379<br>1396<br>1402<br>1438	1467<br>1485<br>1563	-	1467<br>1485<br>1563	1594
	T_{1-H} **Qd**	1643<br>1658	-	1685	1380	1649<br>1660<br>1671<br>1680<br>1707	1673	1658<br>1673	1627	1649<br>1674	1691	1541<br>1560	1560<br>1570<br>1627<br>1643<br>1658	1560<br>1570	-	1513<br>1592<br>1366	1627<br>1643<br>1658<br>1673<br>1421	1513<br>1526<br>1478<br>1495	1517<br>1525<br>1543	1366<br>1392<br>1415	1366<br>1379<br>1384<br>1392	1458	-	1525<br>1592<br>1458	1589
	S'_0 **Qd**	1641<br>1643	-	1696	1383	1663<br>1672<br>1674<br>1704	1679	1728	1668	1687<br>1705	1697	1539<br>1549	1561<br>1641<br>1643	1576	-	1500<br>1379<br>1462	1668	1500<br>1503<br>1516<br>1479	1525<br>1489	1367<br>1379	1375<br>1379<br>1380<br>1406	1622	-	1500<br>1549<br>1622	1609
	S_0 **Qa**	1656	1670<br>1674	-	-	1649<br>1687<br>1693<br>1700<br>1711<br>1721	1693	1719	1663	1669<br>1683	1708	1573	1538<br>1552<br>1656	1586	1577<br>1583	-	1663<br>1719<br>1442<br>1456	1497<br>1498<br>1515<br>1518<br>1478	1544<br>1550	1367<br>1388<br>1456<br>1456	1366<br>1370	1626<br>1388	1399<br>1423<br>1649	1538<br>1626	1593
	S_1 **Qa**	1671	1649<br>1656	-	-	1680<br>1688<br>1695<br>1702<br>1713<br>1722	1688<br>1695	1649<br>1653	1621	1663<br>1690	1687	1621	1549<br>1593<br>1621<br>1671	1584	1569<br>1649<br>1656	-	1621<br>1649<br>1653<br>1387	1496<br>1499<br>1517<br>1520<br>1482<br>1485	1541<br>1545	1510	1364<br>1371<br>1416	1510<br>1592<br>1487	1420<br>1656	1510<br>1545<br>1592<br>1364<br>1487	1617
	T_1 **Qa**	1664<br>1667	1666	-	-	1648<br>1675<br>1700<br>1712<br>1722	1687<br>1694	1646	1629	1667<br>1678<br>1687	1709	1573	1629<br>1664	1586	1575<br>1666	-	1629<br>1646<br>1489	1479<br>1496<br>1498<br>1515<br>1519	1542<br>1551	1387<br>1405<br>1421<br>1450<br>1473	1365<br>1371<br>1387	1567<br>1450<br>1473<br>1489	1648<br>1399<br>1424	1567<br>1387<br>1450<br>1473<br>1489	1594
	T_{1-H} **Qa**	1658<br>1672<br>1673	1691	-	-	1647<br>1652<br>1714<br>1728	1694	1672<br>1673<br>1685	1637	1663<br>1685	1707	1553	1553<br>1637<br>1658<br>1672<br>1673	1581<br>1637	1581	1512<br>1597	1637<br>1658<br>1673<br>1425	1477<br>1501<br>1508<br>1522<br>1477	1539<br>1551	1418	1367<br>1396	1512<br>1611	1403<br>1428<br>1647	1512<br>1539<br>1597<br>1611	1596
	S'_0 **Qa**	1656	1688	-	-	1649<br>1654<br>1668<br>1686<br>1694<br>1713<br>1730	1694<br>1686	1737	1674	1706<br>1727	1714	1569<br>1562	1547<br>1562<br>1656	1591	1578	1579<br>1359	1674<br>1442	1501<br>1505<br>1523<br>1476	1512<br>1483	1359	1368<br>1369<br>1380<br>1416	1623	1401<br>1649	1562<br>1579<br>1623	1596
	S_0 **Qb**	1650<br>1665	-	1691<br>1698	1362	1658<br>1676<br>1679<br>1691<br>1705<br>1711	1689	1737	1650<br>1665	1668<br>1685<br>1689	1707	1575	1555<br>1567<br>1581<br>1650<br>1665	1555<br>1567	-	-	1650<br>1737<br>1449<br>1462	1497<br>1505<br>1511<br>1477	1537<br>1542<br>1548	1385<br>1403<br>1449	1367<br>1454	1624<br>1385	1658<br>1393	1537<br>1581<br>1624<br>1385	1596
	S_1 **Qb**	1665<br>1678<br>1684	-	1691<br>1697<br>1698	1364	1659<br>1676<br>1684<br>1691<br>1705<br>1711	1698<br>1697<br>1684<br>1665	1665<br>1684<br>1697<br>1698	1621	1605<br>1727	1689	1551<br>1569<br>1584<br>1621	1551<br>1569<br>1584<br>1621<br>1665<br>1678	1551<br>1569	-	-	1551<br>1621<br>1697<br>1698	1499<br>1506<br>1512<br>1518<br>1454<br>1476	1526<br>1437	1433	1368<br>1370<br>1382<br>1394	1508<br>1588<br>1359<br>1444<br>1479	1659<br>1394	1508<br>1538<br>1588<br>1359<br>1444<br>1479	1605<br>1613
	T_1 **Qb**	1665<br>1668	-	1675<br>1691<br>1697	1351<br>1363	1656<br>1675<br>1678<br>1680<br>1690<br>1691<br>1705<br>1712	1690	1649	1629	1665<br>1678<br>1680	1708	1568	1555<br>1568<br>1629<br>1665<br>1668	1555<br>1562<br>1568	-	-	1451<br>1483<br>1629<br>1649<br>1440	1497<br>1454<br>1477<br>1497<br>1506<br>1511	1540<br>1550	1471<br>1384<br>1402	1366<br>1380	1451<br>1471<br>1562	1394<br>1656	1451<br>1471<br>1483<br>1562<br>1384	1597

	T_{1-H} **Qb**	1665<br>1678	-	1661	1434	1655<br>1676<br>1690<br>1706<br>1708<br>1712	1698	1678<br>1690<br>1698	1632	1662<br>1687	1706	1557	1557<br>1632<br>1665<br>1678	1595	-	1532<br>1542<br>1603	1632<br>1698<br>1414<br>1434<br>1459	1479<br>1490<br>1496<br>1516<br>1517	1541<br>1542<br>1549	1414<br>1418<br>1434<br>1459	1368<br>1378<br>1396<br>1403	1459	1397<br>1655	1532<br>1603	1596
	$S_0^{'}$ **Qb**	1657	-	1668<br>1675	1372	1668<br>1683<br>1686<br>1697<br>1707<br>1708	1693	1743	1673	1705<br>1725	1715	1550<br>1562	1550<br>1562<br>1657	1589	-	1622<br>1369<br>1380	1673<br>1459	1475<br>1482<br>1497<br>1511<br>1513<br>1528<br>1452	1507	1362<br>1369	1370<br>1380	1622	1668<br>1398	1562<br>1622	1598
	S_0 **Qc**	1664<br>1651	1705	-	-	1652<br>1673<br>1678<br>1695<br>1713	1687	1733	1651<br>1664	1668<br>1684	1707	1574<br>1580	1553<br>1580<br>1651<br>1664	1553<br>1566	1559	-	1651	1477<br>1495<br>1503<br>1509<br>1514	1537<br>1542<br>1548	1385<br>1404	1366<br>1367	1624	1652<br>1393<br>1413	1537<br>1580<br>1624<br>1385	1596
	S_1 **Qc**	1664<br>1677	1705	-	-	1654<br>1674<br>1681<br>1690<br>1696<br>1713	1664<br>1694	1681<br>1694	1621	1606<br>1727	1689<br>1690	1569<br>1621	1569<br>1621<br>1664<br>1677	1569	1558	-	1621<br>1681<br>1694	1477<br>1497<br>1501<br>1510<br>1514<br>1527	1526<br>1438	1432<br>1443	1358<br>1369<br>1392<br>1415	1481<br>1509<br>1358	1654<br>1397<br>1413	1481<br>1509<br>1538<br>1358	1606<br>1613
	T_1 **Qc**	1665<br>1667	1705	-	-	1650<br>1673<br>1678<br>1687<br>1695<br>1713	1687	1650	1629	1665<br>1667<br>1678<br>1679	1709	1562<br>1568	1553<br>1562<br>1568<br>1629<br>1665<br>1667	1553<br>1562<br>1568	1559	-	1450<br>1483<br>1629<br>1650	1478<br>1495<br>1503<br>1511<br>1513	1540<br>1550	1450<br>1472<br>1385<br>1401	1450<br>1366<br>1379<br>1385	1450<br>1472<br>1483	1650<br>1414	1472<br>1483<br>1385	1597
	T_{1-H} **Qc**	1658<br>1671	1703	-	-	1652<br>1673<br>1679<br>1695<br>1713	1688	1671<br>1673	1636	1663<br>1685	1706	1636	1552<br>1636<br>1658<br>1671	1552<br>1570	1559	1599<br>1369	1636<br>1658<br>1688<br>1425<br>1459	1478<br>1493<br>1502<br>1511	1539<br>1544<br>1551	1369<br>1418	1367<br>1369<br>1396<br>1418	1459	1652<br>1414	1539<br>1599<br>1459	1597
	$S_0^{'}$ **Qc**	1655	1660<br>1670	-	-	1682<br>1694<br>1703<br>1712	1690<br>1694	1733	1674	1705<br>1726	1714	1549<br>1560	1549<br>1560<br>1655	1579	1567	1359<br>1378<br>1413	1674<br>1454	1475<br>1488<br>1492<br>1506<br>1516<br>1531	1483<br>1510	1359<br>1378	1366<br>1369<br>1413	1623	1425<br>1398	1560<br>1623	1597
	S_0 **Qd**	1665	-	1650<br>1654	1434	1654<br>1680<br>1687<br>1694<br>1703<br>1707<br>1713	1687<br>1694	1736	1651<br>1665	1669<br>1683<br>1687	1707	1575	1556<br>1582<br>1651<br>1665	1573	-	-	1451<br>1462<br>1651<br>1665<br>1736	1453<br>1480<br>1496<br>1518<br>1526	1538<br>1544<br>1549	1451<br>1386	1453<br>1367<br>1396	1626<br>1386	1654<br>1396	1538<br>1582<br>1626	1594
	S_1 **Qd**	1666	-	1644	1440	1655<br>1679<br>1691<br>1698<br>1704<br>1707<br>1713	1680<br>1698	1666<br>1689<br>1698	1552<br>1622	1610<br>1725	1689<br>1691	1583<br>1588<br>1622	1552<br>1582	1552<br>1578	-	-	1443<br>1552<br>1622<br>1666<br>1680<br>1622<br>1698	1453<br>1480<br>1498	1437<br>1528	1408<br>1426<br>1434	1394<br>1419	1508<br>1588	1395<br>1424<br>1655	1481<br>1508<br>1538<br>1588	1605
	T_1 **Qd**	1665<br>1668	-	1646	1436	1653<br>1679<br>1687<br>1695<br>1708<br>1713	1687<br>1695	1646<br>1650<br>1668	1629	1665<br>1678	1709	1575	1556<br>1629<br>1665<br>1668	1566	-	-	1451<br>1483<br>1629<br>1646<br>1650<br>1438	1453<br>1480<br>1496<br>1497<br>1517<br>1528	1542<br>1550	1451<br>1471<br>1384<br>1404	1366<br>1380<br>1384<br>1395<br>1428	1451<br>1471<br>1483<br>1566	1395<br>1423<br>1653	1451<br>1471<br>1483<br>1566	1596
	T_{1-H} **Qd**	1661<br>1676	-	1666<br>1661	1430	1652<br>1680<br>1690<br>1697<br>1703<br>1707<br>1713	1690<br>1697	1676<br>1690<br>1697	1636	1663<br>1685	1706	1555	1555<br>1636<br>1661<br>1676	1581	-	1599	1636<br>1676<br>1690<br>1415<br>1436<br>1461	1478<br>1491<br>1495<br>1514<br>1520<br>1525	1539<br>1544<br>1551	1418<br>1461	1367<br>1372<br>1394<br>1415	1461	1398<br>1423<br>1652	1539<br>1599<br>1461	1595
	$S_0^{'}$ **Qd**	1656	-	1666	1420	1654<br>1680<br>1685<br>1700<br>1706<br>1715	1695	1740	1675	1705<br>1728	1713	1562<br>1569	1550<br>1562<br>1656	1562<br>1587	-	1362	1675<br>1454	1475<br>1491<br>1498<br>1516<br>1520	1484<br>1512	1362<br>1369	1369<br>1380<br>1449	1622	1397<br>1426<br>1654	1562<br>1622	1596

Table S7 Evolution of selected **Qb**, **Qd** EL222 and AsLOV2 IR peaks along the reaction coordinate. Isotopic shifts from the non-deuterated spectra are given with red numbers. Values in cm^{-1}.

			S_0	S_1	T_1	T_{1-H}	S_0'
*s*C=N	EL222	**Qb**	1663-21 m	1664-21 m	1664-21 m	1653-20 m	1654-9 w
		**Qd**	1660-21 w	1658-20 w	1660-20 w	1664-19 m	1664-20 w
	AsLOV2	**Qb**	1662-20 m	1666-21 w	1663-21 w	1655-20 w	1639-18 m
		**Qd**	1661-18 m	1661-18 m	1661-17 m	1664-21 m	1684-11 m
sC_2=O_2'	EL222	**Qb**	1665+0 m, 1674+0 m	1593-3 s	1629+0 w	1649+0 vs	1687-1 m
		**Qd**	1665+0 w, 1675+0 m	1652+0 s	1630+0 w, *a*-1638+0 vs	1650+0 vs	1688-1 s
	AsLOV2	**Qb**	1654+0 s	1615+0 s	*a*-1640+0 vs, 1624+0 m	1628-1 m	1666-1 m, 1677+0 w
		**Qd**	1639+0 w, 1652+0 s	1629+0 s	1622+0 m, *a*-1641+0 s	1627+0 m, *a*-1643+0 s	1667-1 s, 1679+0 m
sC_4=O_4'	EL222	**Qb**	1717+0 m	1662+0 m, 1674+0 w	1656+0 w	1672+1 m	1726-1 m
		**Qd**	1715+0 w	1680-1 w	*a*-1638+0 vs, 1655+0 m	1658-1 m	1724+0 w
	AsLOV2	**Qb**	1722+0 m	1587+0 s, 1649+0 w	*a*-1640+0 vs, 1654+0 m	1659+0 m, 1673+0 w	1727-1 m
		**Qd**	1719+0 m	1671+1 s, 1674+0 m	*s*-1641+0 s, 1655+0 w	*a*-1643+0 s, 1673+0 w, 1658+0 m	1727-1 m
*r*HNCOH	EL222	**Qb**	1259-107 w	1259-107 w	1259-106 w	1266-107 w	1257-129 w
		**Qd**	1241-146 w	1242-144 w	1242-144 w	1230-151 w	1233-148 w
	AsLOV2	**Qb**	1258-100 vw, 1264-102 w	1224-134 w, 1260-108 w	1256-102 vw, 1263-103 w	1260-98 vw, 1265-102 w	1277-105 vw
		**Qd**	1238-142 w	1239-142 w	1239-141 w	1227-153 w	1206-177 w

Intensities vs: very strong, s: strong, m: medium, w: weak, vw: very weak

Assignments: *s*: stretch, *a*-: antisymmetric, *s*-: symmetric, *r*: rocking, *sc*: scissor

Table S8 Evolution of selected RsLOV IR peaks along the reaction coordinate. Underlining denotes that scNH$_2$/rHNCOH, or rOH are major modes in that vibration. Isotopic shifts from the non-deuterated spectra are given with red numbers – with the exception of the scNH$_2$/rHNCOH, and rOH modes that shift below 1350 cm^{-1} in D$_2$O. All values in cm^{-1}.

		S_0	S_1	T_1	$T_{1\text{-}H}$	S_0'
sC=O/sC=N	Qa	1659-11 w, 1664-10 m	1636-13 s, 1652-4 w	1656+10 s, 1666 w	1681-10 m, 1685-9 w	1679-9 m
	Qb	1668-8 w, 1682-16 m	1668-29 w, 1682-16 m	1667-8 w, 1682-9 w, 1697 w	1641-20 m	1658-9 m, 1669-6 w 1684-2 m
	Qc	1700-5 m	1700-5 m	1700-5 m	1699-3 m	1646-14 w, 1668-2 s
	Qd	1635-19 m	1628-16 w	1633-13 s, 1650 m	1643-18 vs, 1649-17 w	1645-21 m
sC_2=O_2'	Qa	1664-1 vs	1621+0 s	*s*-1629+0 m *a*-1645-1 vs	1636-1 s	1674+0 vs
	Qb	1650+0 w, 1665-1 vs	1621+0 vs	*s*-1629+0 m, *a*-1649+0 s	1631-1 s	1673+0 vs, 1693+0 w
	Qc	1651+0 w, 1664+0 vs	1621+0 vs	*s*-1629+0 m, *a*-1650+0 s	*s*-1636+0 m, *a*-1658+0 vs	1674+0 vs
	Qd	1651+0 w, 1665+0 vs	1622+0 vs	*s*-1629+0 m, *a*-1648+2 s	*s*-1635-1 m, *a*-1662+1 vs	1676+1 vs
sC_4=O_4'	Qa	1718-1 w	1652-1 m	1665-2, *a*-1645-1 s, *s*-1629+0 m	1672+0 m, 1685+0 m	1737+0 w
	Qb	1737+0 w	1684+0 w, 1698+0 m	*s*-1629+0 m, *a*-1649+0 s, 1667-1 w	1677+0 m, 1690+0 w, 1698+0 w	1742-1 w
	Qc	1733+0 w	1664+0 vs, 1693-1 w	*s*-1629+0 m, *a*-1650+0 s, 1666-1 w	*s*-1636+0 m, *a*-1658+0 vs, 1671+0 w, 1678-1 w	1733+0 w
	Qd	1736+0 w	1666+0 vs, 1680+0 w, 1697-1 m	*s*-1629+0 m, *a*-1648+2 s, 1649-1 m, 1667+0 w	*s*-1635-1 m, *a*-1662+1 vs, 1676+0 m, 1689-1 w	1739+4 w
scNH$_2$/rHNCOH	Qa	1577 w, 1583 s	1569 vw	1567 m, 1575 vw	1579 vw, 1581 w	1578 w, 1579 vw
	Qb	1362 w	1364 vw	1363 w	1403 w	1372 w
	Qc	1559 w	1558 w	1559 w	1559 w	1567 w
	Qd	1434 vw	1440 vw	1436 vw	1430 vw	1420 vw
rO-H	Qa	1399 w, 1649 m	1420 vw, 1656 w	1399 w, 1424 vw, 1648 m	1402 w, 1428 w, 1647 m	1401 w, 1426 vw, 1649 m
	Qb	1658 w	1394 w, 1421 vw, 1659 w	1394 w, 1421 vw, 1656 w	1399 vw, 1425 vw, 1655 m	1372 w, 1398 vw, 1668 w
	Qc	1413 w, 1652 w	1413 w, 1654 w	1414 vw, 1650 w	1414 w, 1652 w	1398 vw, 1427 vw
	Qd	1424 vw, 1654 m	1395 vw, 1655 m	1395 w, 1653 w	1398 w, 1652 w	1397 w, 1654 w

Intensities vs: very strong, s: strong, m: medium, w: weak, vw: very weak
Assignments: *s*: stretch, *a*-: antisymmetric, *s*-: symmetric, *r*: rocking, *sc*: scissor

2b. Difference Spectra

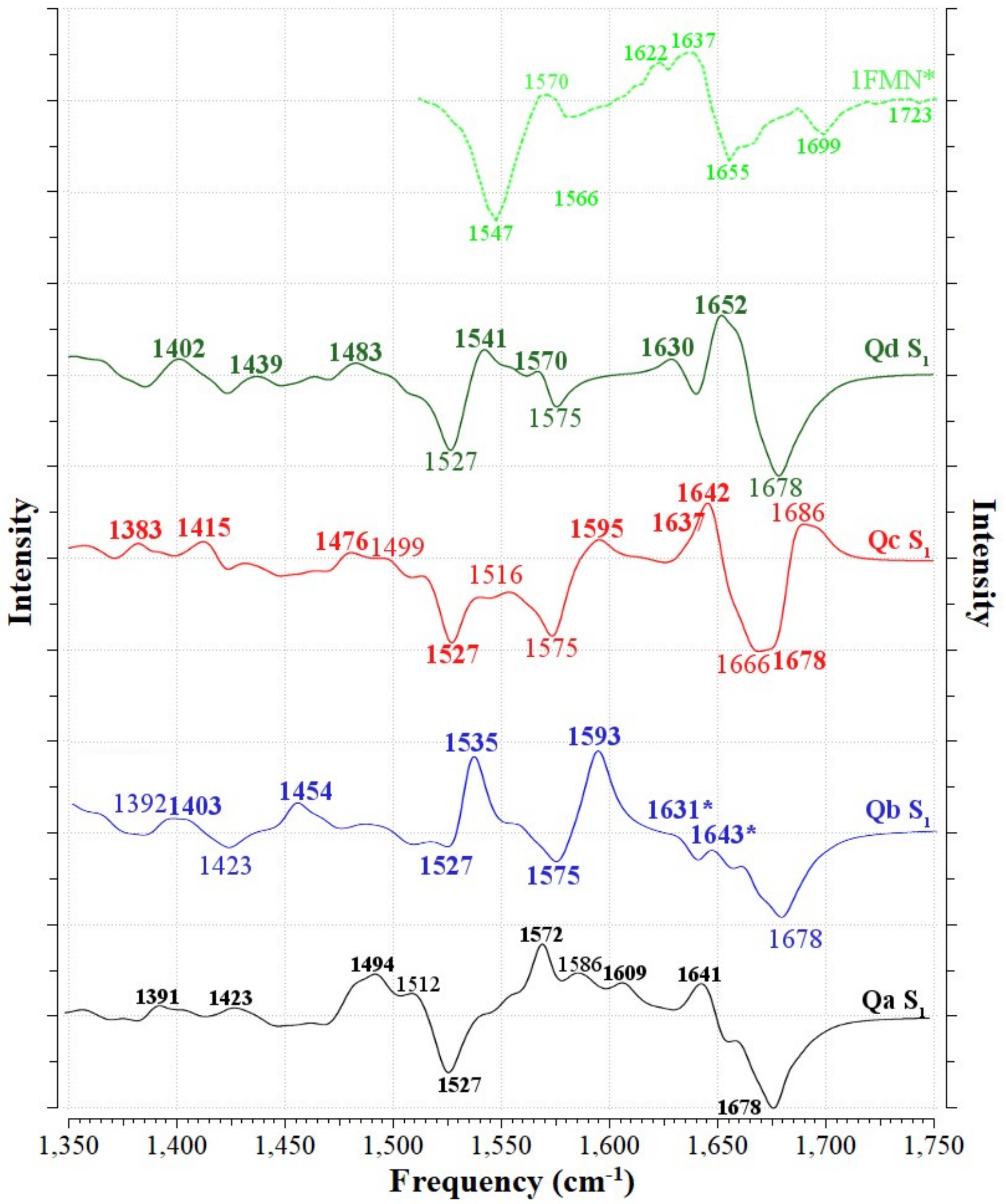

Figure S23 Calculated deuterated infrared difference spectra between the ground state S_0 of **Qa** and the S_1 states of **Qa**-**Qd** of the EL222 cluster. The difference spectra of **Qa** (—), **Qb** (—), **Qc** (—), and **Qd** (—) are included with the indicated line colours. The experimental 1FMN* EADS of EL222 is included in light green (—). The calculated spectra were normalised before subtraction, scaled by 0.97 and a half-width at half-maximum peak (HWHM) of 8 cm^{-1} was applied to resemble the experimental curve. The apparent strongest peaks after subtraction are labelled with the respective colour.

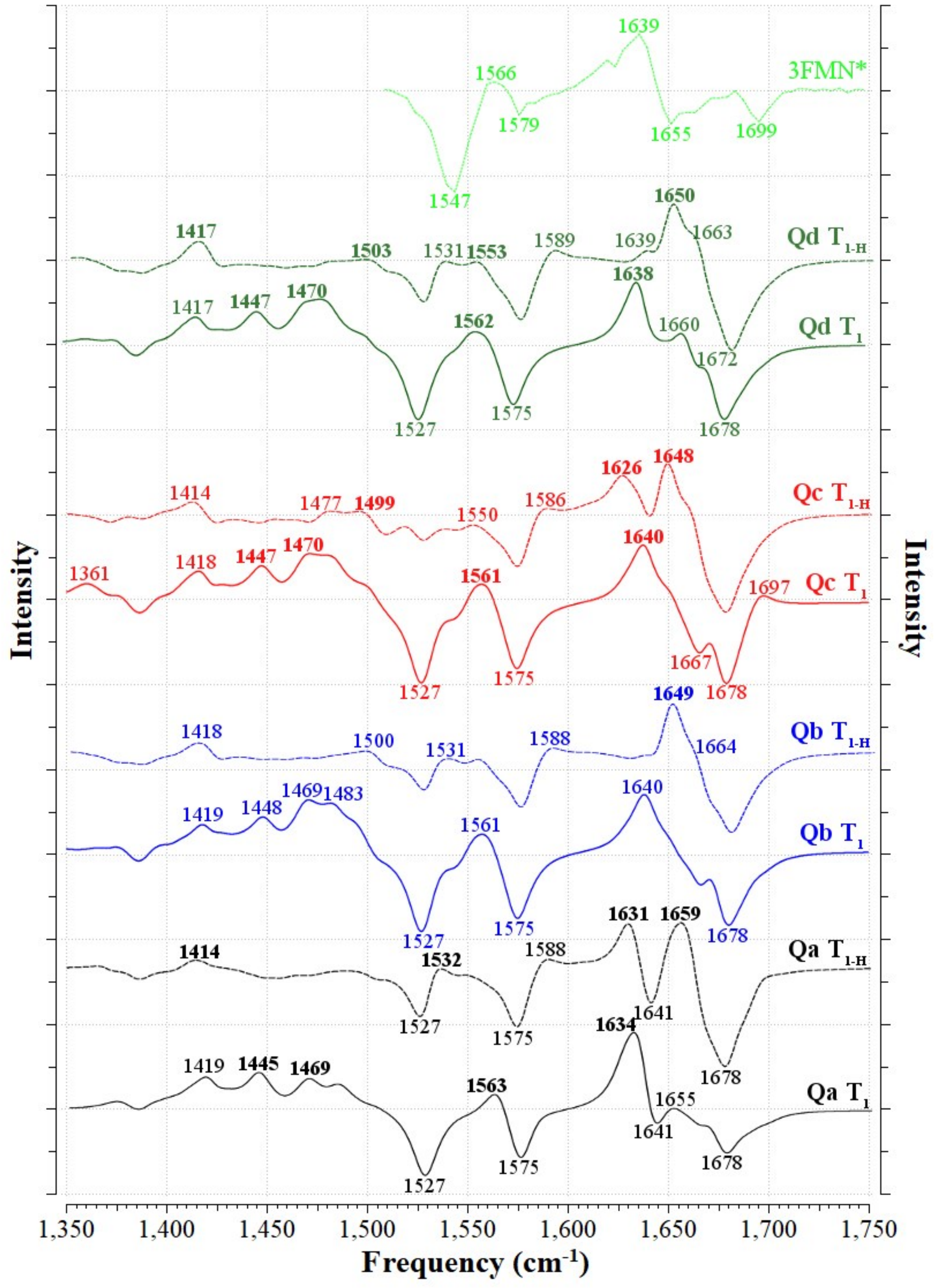

Figure S24 Calculated deuterated infrared difference spectra between the ground state S_0 of **Qa** and the T_1 and $T_{1\text{-}H}$ states of **Qa-Qd** of the EL222 cluster. The T_1 difference spectra of **Qa** (—), **Qb** (—), **Qc** (—), **Qd** (—) and the T_1-H difference spectra of **Qa** (––), **Qb** (––), **Qc** (––), and **Qd** (––) are plotted with the indicated line colours. The experimental 3FMN* EAS spectrum of EL222 is included in light green (—). The calculated spectra were normalised before subtraction, scaled by 0.97 and a half-width at half-maximum peak (HWHM) of 8 cm^{-1} was applied to resemble the experimental curve. The apparent strongest peaks after subtraction are labelled with the respective colour.

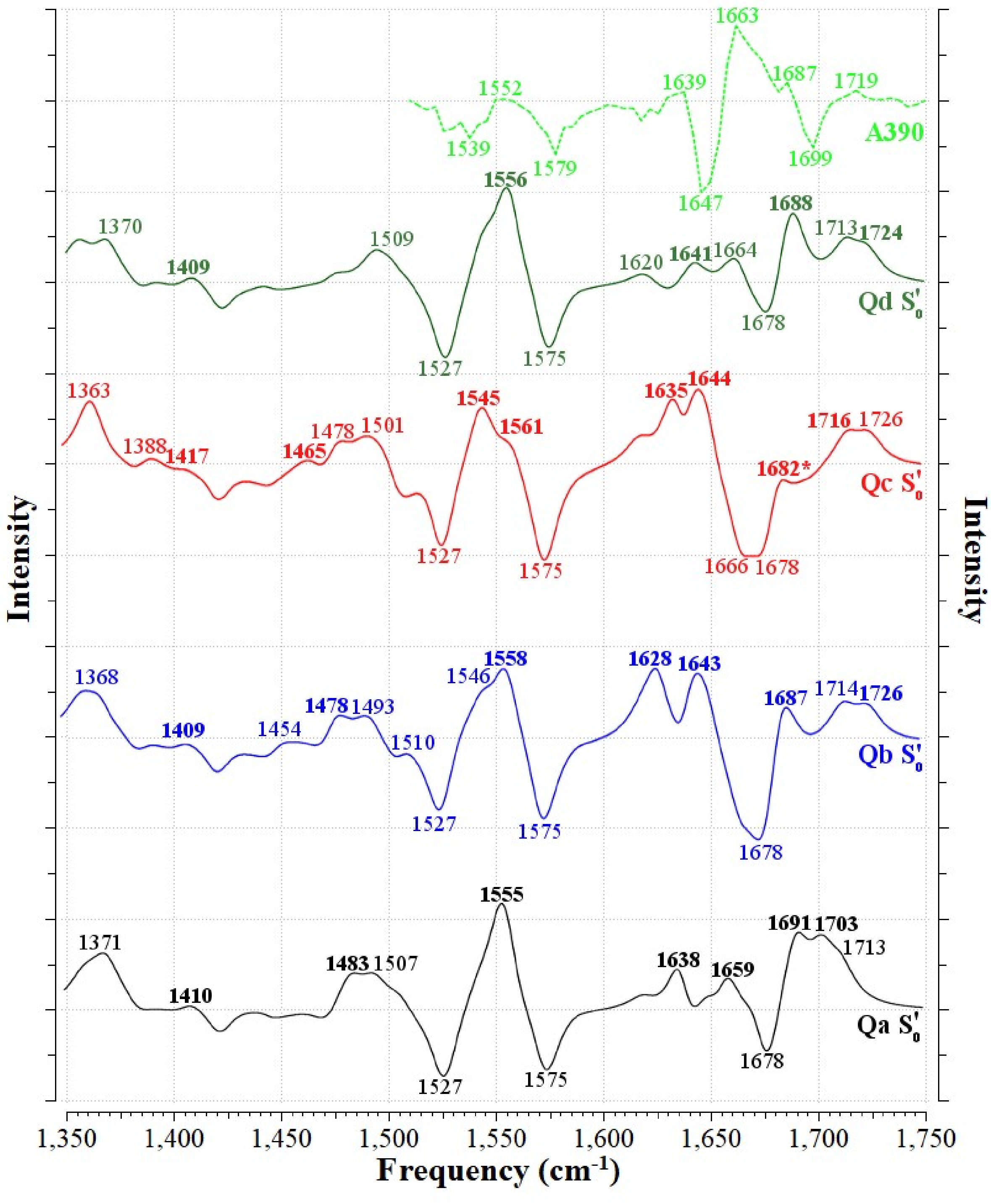

Figure S25 Calculated deuterated infrared difference spectra between the ground state S_0 of **Qa** and the adduct $S_0^{'}$ states of **Qa**-**Qd** of the EL222 cluster. The difference spectra of **Qa** (—), **Qb** (—), **Qc** (—), and **Qd** (—) are plotted with the indicated line colours. The experimental A390 EADS of EL222 is included in light green (—). The calculated spectra were normalised before subtraction, scaled by 0.97 and a half-width at half-maximum peak (HWHM) of 8 cm^{-1} was applied to resemble the experimental curve. The apparent strongest peaks after subtraction are labelled with the respective colour.

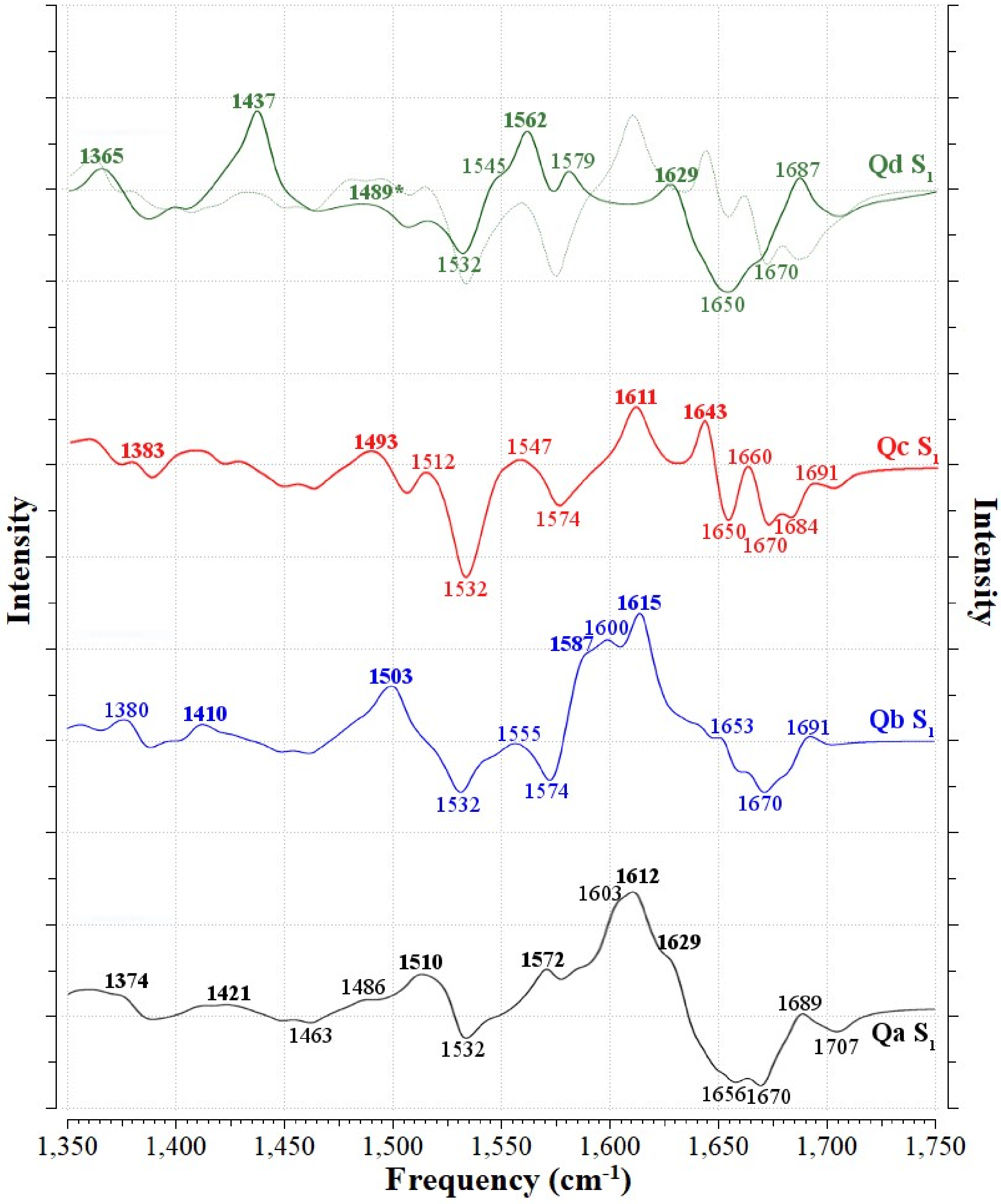

Figure S26 Calculated deuterated infrared difference spectra between the ground state S_0 of **Qa** and the S_1 states of **Qa**-**Qd** of the AsLOV2 cluster. The difference spectra of **Qa** (—), **Qb** (—), **Qc** (—), **Qd** (—) and **Qd'** (– –) are plotted with the indicated line colours. The calculated spectra were normalised before subtraction, scaled by 0.97 and a half-width at half-height maximum (HWHM) of 8 cm^{-1} was applied to resemble the experimental curve. The apparent strongest peaks after subtraction are labelled with the respective colour.

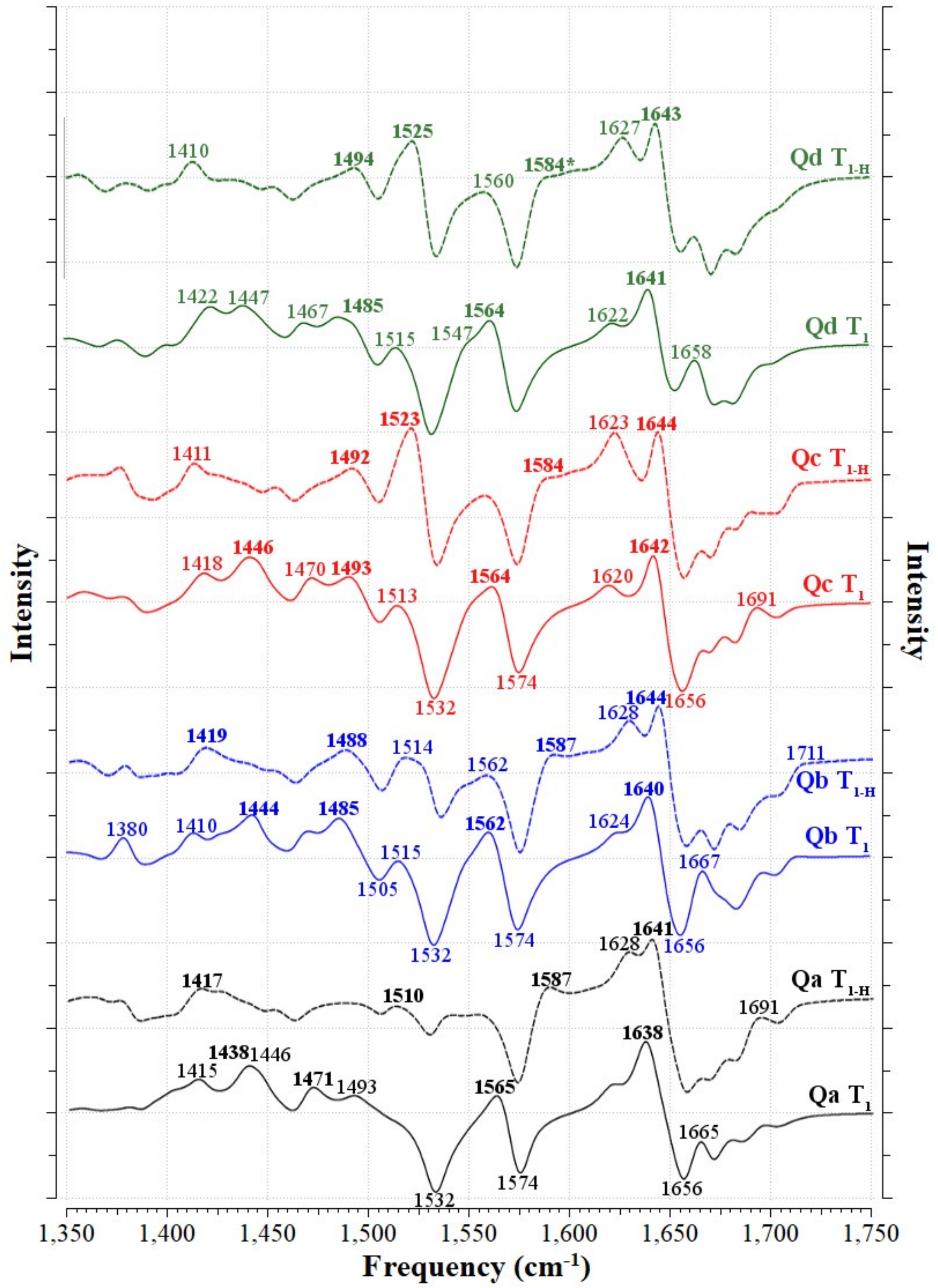

Figure S27 Calculated deuterated infrared difference spectra between the ground state S_0 of **Qa** and the T_1 and T_{1-H} states of **Qa-Qd** of the AsLOV2 cluster. The T_1 difference spectra of **Qa** (—), **Qb** (—), **Qc** (—), **Qd** (—) and the T_{1-H} difference spectra of **Qa** (––), **Qb** (––), **Qc** (––), and **Qd** (––) are included with the indicated line colours. The calculated spectra were normalised before subtraction, scaled by 0.97 and a half-width at half-height maximum (HWHM) of 8 cm^{-1} was applied to resemble the experimental curve. The apparent strongest peaks after subtraction are labelled with the respective colour.

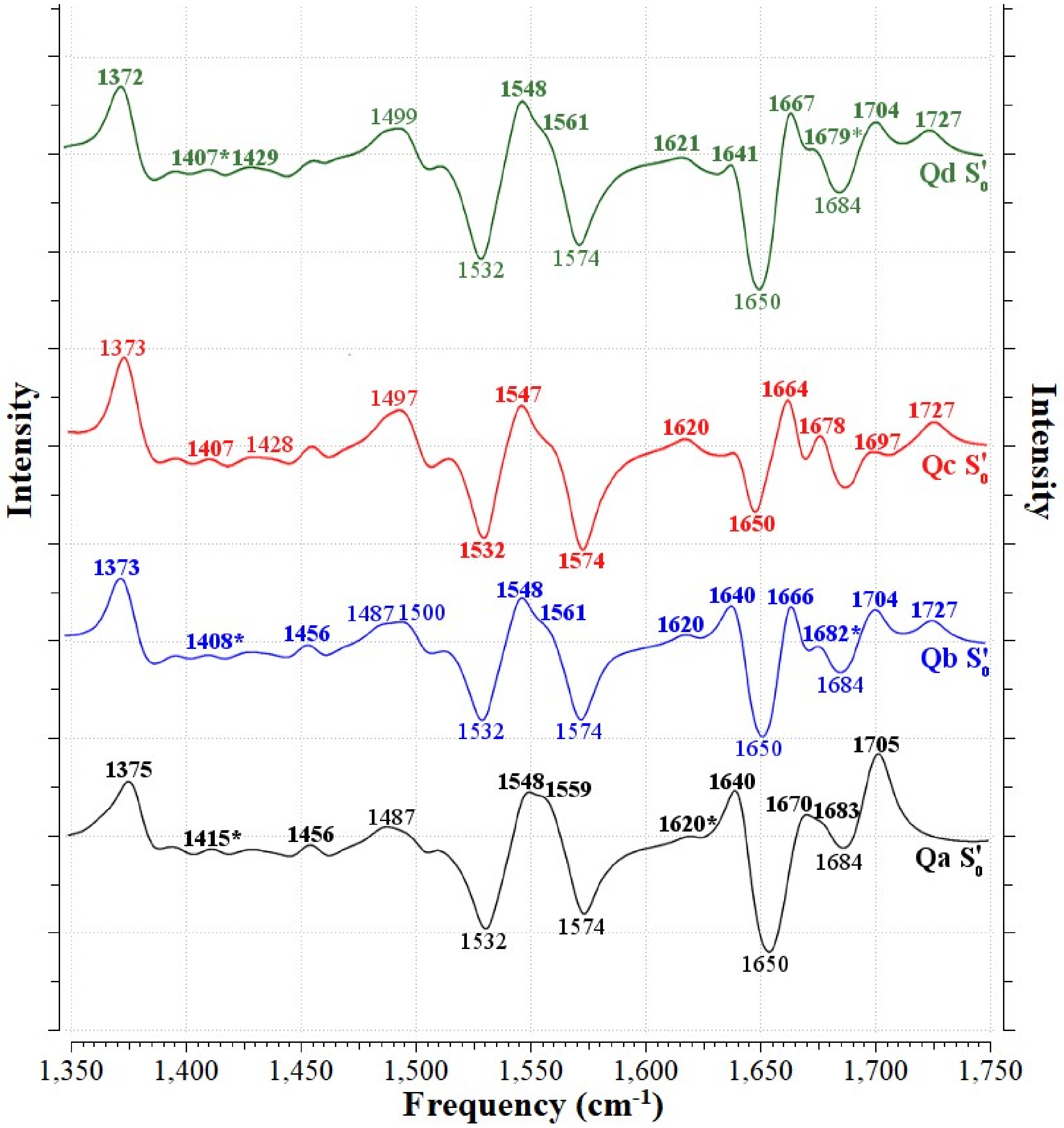

Figure S28 Calculated deuterated infrared difference spectra between the ground state S_0 of **Qa** and the adduct S_0' states of **Qa-Qd** of the AsLOV2 cluster. The difference spectra of **Qa** (—), **Qb** (—), **Qc** (—), and **Qd** (—) are included with the indicated line colours. The calculated spectra were normalised before subtraction, scaled by 0.97 and a half-width at half-height maximum (HWHM) of 8 cm^{-1} was applied to resemble the experimental curve. The apparent strongest peaks after subtraction are labelled with the respective colour.

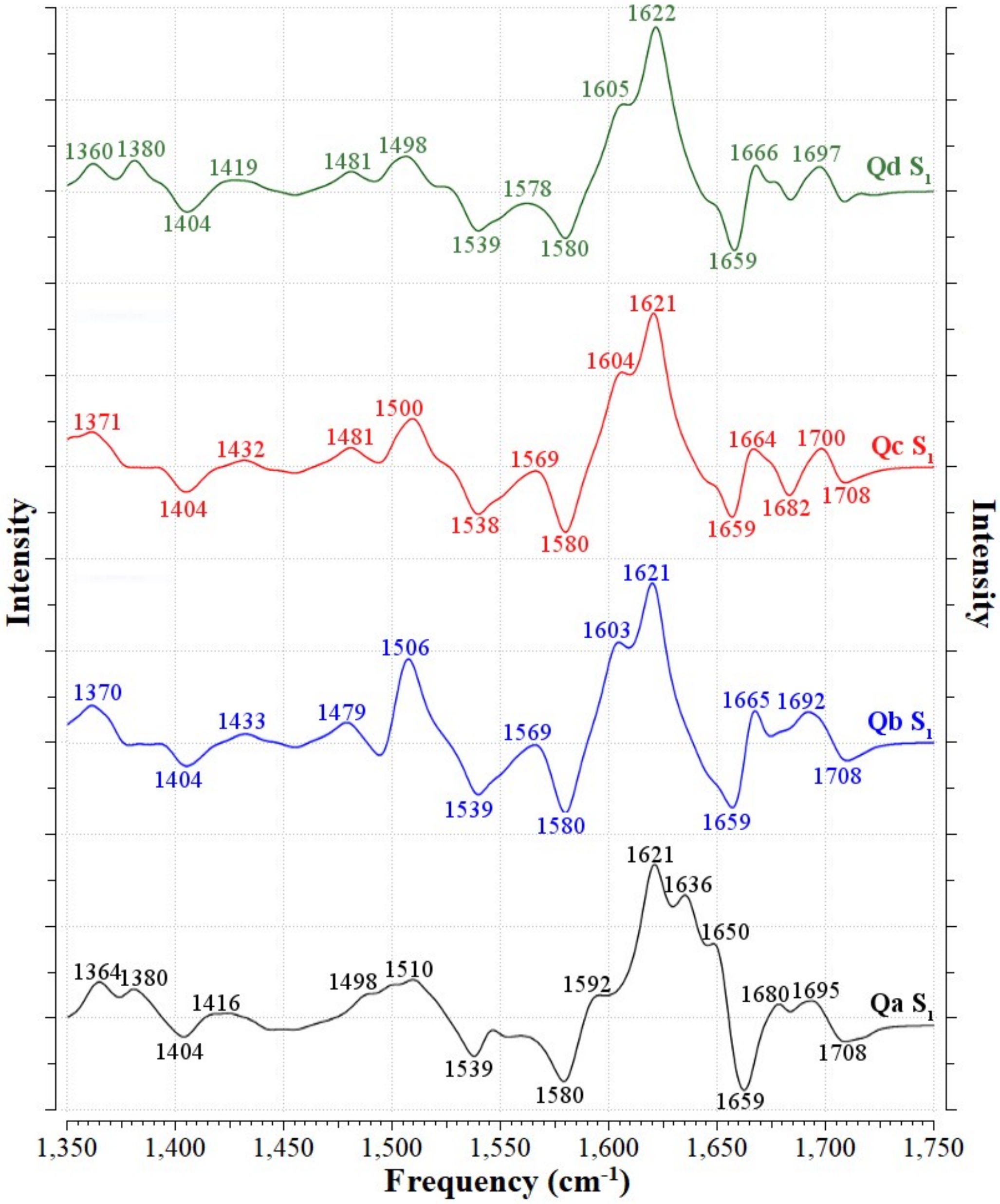

Figure S29 Calculated deuterated infrared difference spectra between the ground state S_0 of **Qa** and the S_1 states of **Qa**-**Qd** of the RsLOV cluster. The difference spectra of **Qa** (—), **Qb** (—), **Qc** (—), and **Qd** (—) are included with the indicated line colours. The spectra were normalised before subtraction, scaled by 0.97 and a half-width at half-height maximum (HWHM) of 8 cm^{-1} was applied. The apparent strongest peaks after subtraction are labelled with the respective colour. Since the same S_0 spectrum is subtracted from all plots, the negative peaks ground state peaks are labelled mostly in the first **Qa** (—) plot.

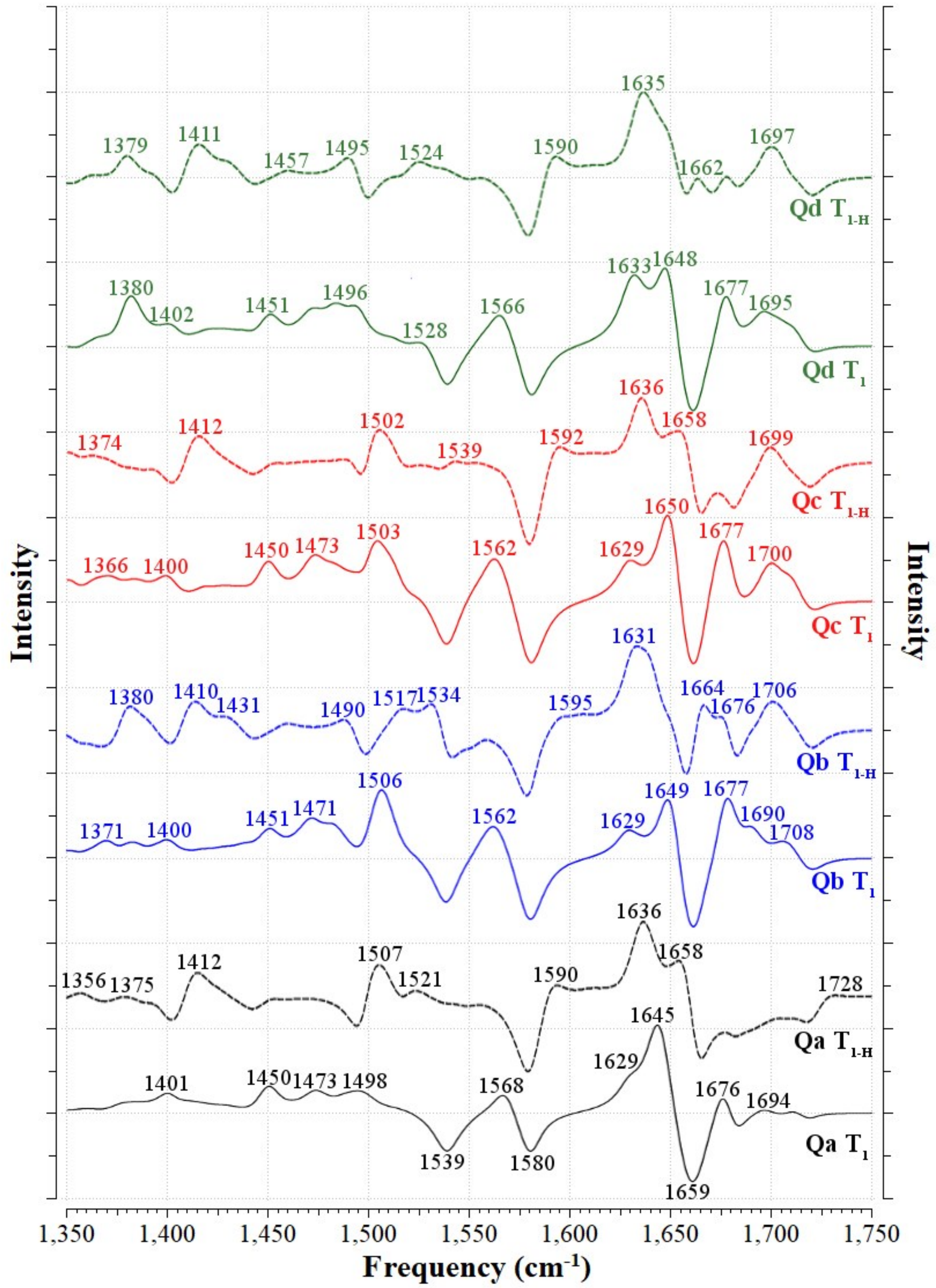

Figure S30 Calculated deuterated infrared difference spectra between the ground state S_0 of **Qa** and the T_1 and $T_{1\text{-}H}$ states of **Qa-Qd** of the RsLOV cluster. The T_1 difference spectra of **Qa** (—), **Qb** (—), **Qc** (—), **Qd** (—) and the $T_{1\text{-}H}$ difference spectra of **Qa** (––), **Qb** (––), **Qc** (––), and **Qd** (––) are included with the indicated line colours. The spectra were normalised before subtraction, scaled by 0.97 and a half-width at half-height maximum (HWHM) of 8 cm^{-1} was applied. The apparent strongest peaks after subtraction are labelled with the respective colour. Since the same S_0 spectrum is subtracted from all plots, the negative peaks belonging to the **Qa** ground state are labelled mostly in the first **Qa** (–) plot.

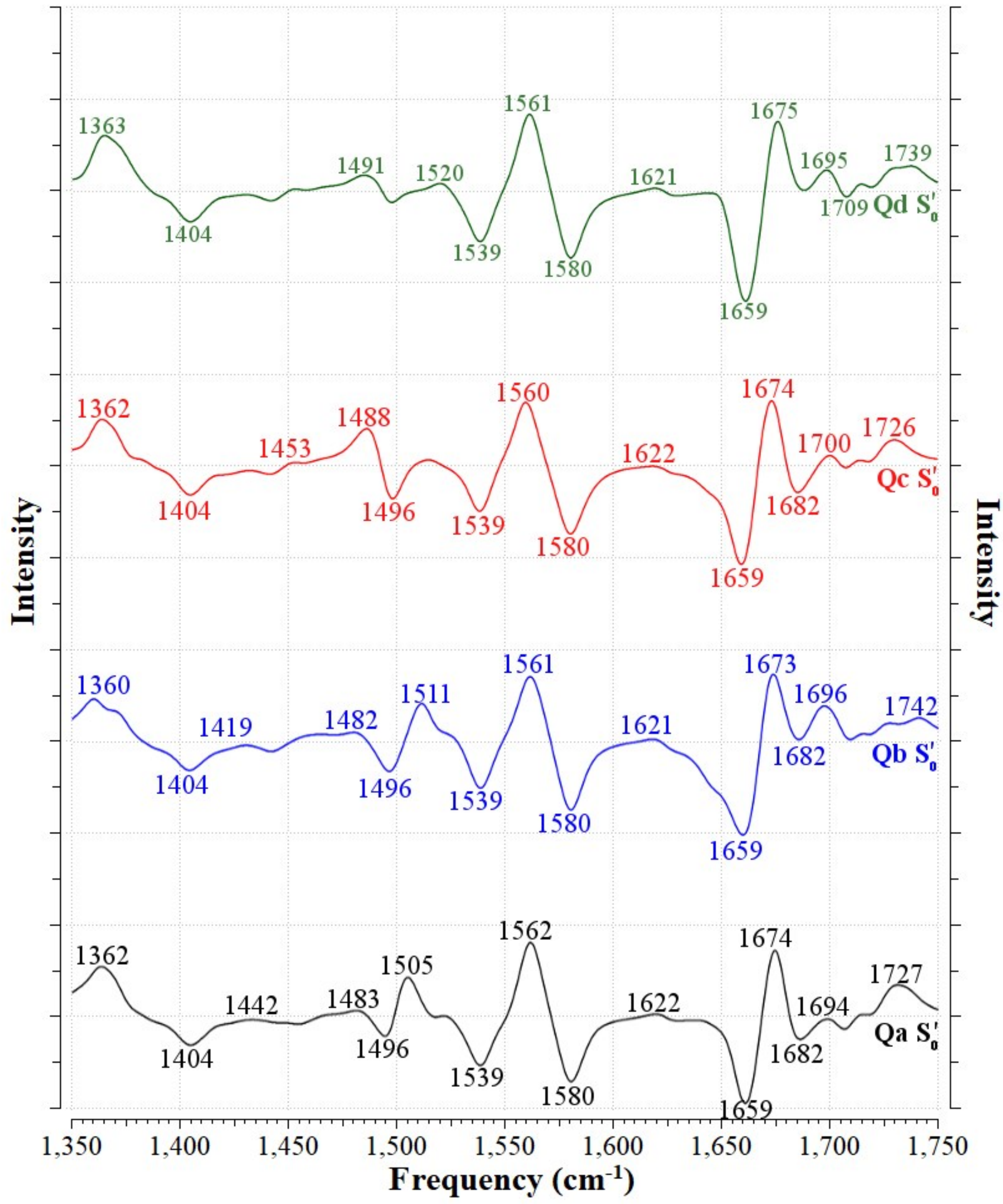

Figure S31 Calculated deuterated infrared difference spectra between the ground state of **Qa** and the adduct $S_0^{'}$ states of **Qa-Qd** of the RsLOV cluster. The difference spectra of **Qa** (—), **Qb** (—), **Qc** (—), and **Qd** (—) are included with the indicated line colours. The calculated spectra were normalised before subtraction, scaled by 0.97 and a half-width at half-height maximum (HWHM) of 8 cm^{-1} was applied. The apparent strongest peaks after subtraction are labelled with the respective colour. Since the same S_0 spectrum is subtracted from all plots, the negative peaks ground state peaks are labelled mostly in the first **Qa** (—) plot.

EL222

Exp.	Qa	Assignment	Qc	Assignment
**1FMN***		**Ground State (S$_0$)**		
1547†	1527+0	FMN	1527+0	FMN
1579†	1575+1	FMN, *sc*NH$_2$	1574+0	FMN, *sc*NH$_2$
1655†	1667-1	*s*C=O, *s*C$_2$=O$_{2'}$	1669+0	*s*C=O, *s*C$_2$=O$_{2'}$
1699†	1688-5	*s*C=O, *s*C$_4$=O$_{4'}$	1695-7	*s*C=O, *s*C$_4$=O$_{4'}$
MAD	11.8 (11.0)		10.8 (10.5)	
**1FMN***		**Excited State (S$_1$)**		
1378‡	1391+1	*r*N$_3$-H, CH$_3$	1383-1	FMN, *r*N$_3$-H
1419‡	1423+17	CH$_3$, *r*N$_3$-H, CH$_2$	1415+1	*sc*CH$_2$, CH$_3$/*s*C-N
1498‡	1494+2<br>1512+0	*r*N-H<br>FMN, *sc*CH$_2$, CH$_3$	1476+0	*r*N-H
1571†	1572-34	*sc*NH$_2$, *s*C=O (*sc*NH$_2$)	1595-4	*s*C=O/FMN, *r*C$_{6,9}$-H, *sc*H$_2$O
1623†	1609-11	*sc*NH$_2$, *s*C$_2$=O$_{2'}$, *s*C$_4$=O$_{4'}$ (*sc*NH$_2$)	1637#-6	*s*C=O, *sc*NH$_2$
1639†	1641-1	*r*N$_3$-H, *s*C$_2$=O$_{2'}$, *sc*NH$_2$	1642-4	*r*N$_3$-H, *s*C$_2$=O$_{2'}$, *sc*NH$_2$
MAD	6.3 (12.0)		12.0 (14.7)	
**3FMN***		**Excited State (T$_1$)**		
1428‡	1445+0	*sc*CH$_2$, *r*N$_3$-H, CH$_3$	1447+0	*r*N$_3$-H, *sc*CH$_2$
1488‡	1469+0	CH$_3$, *r*C$_{6,9}$-H	1470+1	FMN, CH$_3$
1567†	1563+0	CH$_3$, *r*C$_{6,9}$-H	1561+0	FMN, *sc*NH$_2$
1639†	1634+0	*r*N$_3$-H, *s*C$_2$=O$_{2'}$	1640+0	*r*N$_3$-H, *s*C$_2$=O$_{2'}$, *s*C=O, *s*C$_4$=O$_{4'}$
MAD	11.3 (11.3)		11.0 (11.3)	
**3FMN***		**Excited State (T$_{1\text{-H}}$)**		
1428‡	1414-1	*r*N$_3$-H, CH$_3$, *sc*CH$_2$	1412#-1	CH$_3$, *sc*CH$_2$
1488‡	1508#+0	*r*N-H	1499#+0	*r*N-H
1567†	1532-1	FMN, *r*N-H	1586-18	FMN, *sc*NH$_2$, (*r*N$_5$-H)
1639†	1631-1<br>1659+0	*r*N$_3$-H, *s*C=O, *s*C$_4$=O$_{4'}$<br>*r*N$_3$-H, *s*C=O, *s*C$_2$=O$_{2'}$	1626-11<br>1648-1	*s*C=O (*sc*NH$_2$)<br>*r*N$_3$-H, *s*C=O, *s*C$_4$=O$_{4'}$
MAD	19.3 (18.5)		14.8 (16.3)	
**A390**		**Adduct State (S$_0'$)**		
-	-	-	-	-
1418‡	1410+3	CH$_2$/CH$_3$, *r*N$_5$-H	1417+3	*sc*CH$_2$
1450‡	1483+0	*r*N-H	1465+1	*r*N$_3$-H
1555†	1555+0	*sc*NH$_2$, FMN	1545-1<br>1561+1	*sc*NH$_2$, FMN (*r*N$_5$-H)<br>*sc*NH$_2$, FMN
-	-	-	-	-
1639†	1638+0	*s*C=O	1635-8	*s*C=O, *s*C=O (*s*C=O)
1663†	1659-12	*s*C=O	1644+0	*s*C=O, *sc*NH$_2$
1687†	1691+0	*r*N$_3$-H, *s*C$_2$=O$_{2'}$	1682#+0	*s*C$_2$=O$_{2'}$, *r*N$_3$-H
-	-	-	-	-
1719†	1703-4<br>1713+0	*s*C$_2$=O$_{2'}$, *s*C=O, *s*C$_4$=O$_{4'}$<br>*s*C=O	1716+0<br>1726-1	*s*C=O<br>*s*C$_4$=O$_{4'}$, *r*N$_3$-H (*r*N$_5$-H)
MAD	8.0 (9.0)		7.6 (7.7)	

AsLOV2

Exp.	Qa	Assignment	Qc	Assignment
**1FMN***		**Ground State (S$_0$)**		
1550‡	1532+0	FMN, *r*N$_3$-H, *sc*NH$_2$	1531+0	FMN, *r*N$_3$-H, *sc*NH$_2$
1583‡	1574-1	FMN, (*sc*NH$_2$), *sc*NH$_2$	1569+0	FMN, *sc*NH$_2$, *sc*NH$_2$
1669‡	1670-1	*s*C=O, *s*C=O	1651+0	*s*C$_2$=O$_{2'}$, *r*N$_3$-H, *s*C=O
1690‡	1684-6	*s*C=O	1691-6	*s*C=O
	8.5 (7.0)		13.0 (14.5)	
**1FMN***		**Excited State (S$_1$)**		
1375‡	1374+11	*r*N-H, *r*C-H	1381+0<br>1383-93	*r*N-H, *r*N$_3$-H<br>*r*N-H, *sc*CH$_2$
1413‡	1421+135<br>1430-0	*sc*CH$_2$, *s*C-N, *r*N-D(H)<br>CH$_3$, *r*N$_3$-H, CH$_2$	1415+146<br>1431+0	*sc*CH$_2$, *s*C-N, *r*N-D(H)<br>*sc*CH$_2$
1498‡	1510+1	FMN, C$_{6,9}$-H, CH$_3$	1493+0<br>1512+0	*r*N-H<br>*r*N-H
1574‡	1572-2	*sc*NH$_2$	1546-1	*r*N-H
1622‡	1612-1<br>1629-3	*sc*NH$_2$, *sc*NH$_2$, *s*C$_2$=O$_{2'}$, *s*C$_4$=O$_4$, *s*C=O<br>*r*N$_3$-H, *sc*NH$_2$, *s*C$_4$=O$_{4'}$	1611+0<br>1643+0	*r*N$_3$-H, *s*C$_2$=O$_{2'}$, *sc*NH$_2$, *sc*NH$_2$<br>*s*C=O, *sc*NH$_2$
-	-	-	-	-
	6.0 (9.8)		10.8 (13.4)	
**3FMN***		**Excited State (T$_1$)**		
1438‡	1438+0	*r*N$_3$-H, *sc*CH$_2$	1446+0	*r*N$_3$-H, *sc*CH$_2$
1491‡	1471+1	FMN, C$_{6,9}$-H	1493+0	*r*N-H
1567‡	1565+0	*sc*NH$_2$, FMN, *r*C$_{6,9}$-H	1564+0	*r*C$_{6,9}$-H, *sc*NH$_2$
1622‡	1638-1	*r*N$_3$-H, *s*C=O, *s*C$_4$=O$_{4'}$	1642+0	*s*C=O, *r*N$_3$-H, *s*C$_4$=O$_{4'}$
	9.5 (10.0)		8.3 (8.3)	
**3FMN***		**Excited State (T$_{1\text{-H}}$)**		
1438‡	1417+136<br>1451+20	*s*C-N, *r*N-D(H)<br>*r*N$_3$-H, *sc*CH$_2$	1411+147<br>1423-5	*s*C-N, *r*N-D(H)<br>*r*N$_3$-H
1491‡	1510+0	*r*N-H	1492+0<br>1523-1	*r*N-H<br>FMN, *r*N-H
1567‡	1587-10	FMN (*r*N$_5$-H)	1584-8	FMN (*r*N$_5$-H)
1622‡	1641+0	*r*N$_3$-H, *s*C$_2$=O$_{2'}$, *s*C=O	1644+0	*s*C=O, *sc*NH$_2$, *r*N$_3$-H, *s*C$_4$=O$_{4'}$
	19.8 (18.8)		16.8 (14.5)	
**A390**		**Adduct State (S$_0'$)**		
1389‡	1375+7	FMN, *sc*CH$_2$	1373-7	FMN, *sc*CH$_2$ (*r*N$_3$-H)
1417‡	1415#+0	*sc*CH$_2$, CH$_3$	1407#+0	*r*N-H, *sc*CH$_2$
1435‡	1456-1	*r*N$_3$-H	1412-11	CH$_3$, CH$_2$ (*r*N$_3$-H)
1541‡	1548-2<br>1559+0	*sc*NH$_2$, *sc*NH$_2$ (*sc*NH$_2$)<br>*sc*NH$_2$	1547+0<br>1562+0	*sc*NH$_2$, FMN<br>*sc*NH$_2$/*sc*NH$_2$
1610‡	1620#-1	FMN, *r*C$_{6,9}$-H	1620-1	FMN
1634‡	1640+0	*s*C=O, *sc*NH$_2$	1639-11	*s*C=O
1665‡	1642+0	*s*C=O, *sc*NH$_2$	1664+0	*r*N$_3$-H, *s*C$_2$=O$_{2'}$, *sc*NH$_2$
1671‡	1670+0	*s*C=O, *r*N-H	1678-1	*s*C=O, *s*C=O (side ch.)
1695‡	1683-6	*s*C=O	1697+0	*s*C=O
1722‡	1705-5	*s*C=O, *s*C$_4$=O$_{4'}$	1705+0<br>1727-1	*s*C=O<br>*r*N$_3$-H, *s*C$_4$=O$_{4'}$
	11.3 (11.3)		8.5 (8.1)	

FMN: ring breathing, *r*: rocking, *s*: stretch, *sc*: scissoring
#: Relatively intense ES peak in proximity to the experimental, offset by the GS signal
(): Normal mode only in non-deuterated spectrum
†: This study

‡: Taken from ref.[32]

Table S9 EL222 and AsLOV2 IR spectra assignments for the **Qa** and **Qc** models. Normal modes are coloured according to residue; FMN black, Q138/Q513 red, C75/C450 orange, N107/N482 green, N117/N492 violet, Q454 grey, A39/N414 blue and other backbone modes, light blue. Mean absolute deviations are included for each theoretical/experimental correlation. Isotopic shifts from the non-deuterated spectra are given with red numbers. All values in cm^{-1}.

Table S10 EL222 and AsLOV2 IR spectral assignments for the **Qb** and **Qd** models. Normal modes are coloured according to residue; FMN black, Q138/Q513 red, C75/C450 orange, N107/N482 green, N117/N492 violet, Q454 grey, A39/N414 blue, and light blue for backbone modes of other residues. Isotopic shifts from the non-deuterated spectra are given with red numbers. Mean absolute deviations are included for each theoretical/experimental correlation. All values are given in cm^{-1}.

EL222

Exp.	Qb	Assignment	Qd	Assignment
**1FMN***	**Ground State (S_0)**			
1547†	1528+1	*s*FMN	1528+0	*s*FMN
1579†	1573+0	*s*FMN, *sc*NH_2	1575+0	*s*FMN, *sc*NH_2
1655†	1669+0	*s*C=O, *s*C_2=$O_{2'}$	1669+0	*s*C=O, *s*C_2=$O_{2'}$
1699†	1689+0	*s*C=O, *s*C=O	1686-1	*s*C=O, *s*C=O
MAD	12.3 (12.5)		12.5 (12.3)	
**1FMN***	**Excited State (S_1)**			
1378‡	1403-13	*sc*CH_2, CH_3	1402-2	CH_3
1419‡	1454+9	*sc*CH_2, CH_3	1439+2	*sc*CH_2, *r*N_3-H
1498‡	1535+16	*r*N-H	1483+0	*r*$C_{6,9}$-H
1571†	1593-3	*s*C_2=$O_{2'}$, *r*N_3-H, *sc*NH_2	1570-11<br>1570#-1	*r*N-H<br>*sc*NH_2, *s*FMN, *r*N-H
1623†	1631#-10	*r*N_3-H, *s*C=O, *sc*NH_2	1630-2	*s*C=O, *r*N_3-H, *s*C_2=$O_{2'}$, *sc*NH_2
1639†	1643#-1	*s*C=O	1652+0	*s*C_4=$O_{4'}$, *s*C=O
MAD	21.8 (22.2)		13.3 (16.7)	
**3FMN***	**Excited State (T_1)**			
1428‡	1448+0	*r*N_3-H, *sc*CH_2, *r*$C_{6,9}$-H	1447+0	*sc*CH_2, *r*N_3-H
1488‡	1469+0	*s*FMN, CH_3, *r*$C_{6,9}$-H	1470+0	*s*FMN, CH_3, *r*$C_{6,9}$-H
1567†	1561+0	*s*FMN, *sc*NH_2, *sc*NH_2, CH_3, *r*$C_{6,9}$-H	1562+0	*s*FMN, *sc*NH_2, *r*$C_{6,9}$-H
1639†	1640+0	*r*N_3-H, *s*C_2=$O_{2'}$, *s*C=O, *s*C_4=$O_{4'}$	1638+0	*r*N_3-H, *s*C_2=$O_{2'}$, *s*C=O, *s*C_4=$O_{4'}$
MAD	11.3 (11.3)		10.5 (10.5)	
**3FMN***	**Excited State (T_{1-H})**			
1428‡	1419+0<br>1418-131	CH_3, *sc*CH_2<br>*s*C-N	1414+0<br>1417-129	*sc*CH_2, CH_3<br>*s*C-N
1488‡	1508#+0	*r*N-H	1503#+0	*r*N-H
1567†	1588-9	*s*FMN, *sc*NH_2	1553+7	*r*N-H
1639†	1649+0	*s*C_2=$O_{2'}$, *r*N_3-H, *s*C=O	1650+0	*r*N_3-H, *s*C_4=$O_{4'}$
MAD	15.5 (17.5)		12.5 (15.0)	
**A390**	**Adduct State (S_0')**			
-	-	-	-	-
1418‡	1409+2	CH_3/*r*N_5-H, *sc*CH_2	1409+3	(*r*N_5-H), *sc*CH_2, CH_3
1450‡	1478+0	*r*N-H	1494+0	*r*N-H
1555†	1558+0	*sc*NH_2, *s*FMN	1556+0	*sc*NH_2, *s*FMN
-	-	-	-	-
1639†	1628-3	*s*C=O	1641#+0	*s*C=O
1663†	1643+0	*s*C=O	1664-19	*s*C=N
1687†	1687-1	*r*N_3-H, *s*C=O, *s*C_2=$O_{2'}$	1688-1	*r*N_3-H, *s*C_2=$O_{2'}$
-	-	-	-	-

AsLOV2

Exp.	Qb	Assignment	Qd	Assignment
**1FMN***	**Ground State (S_0)**			
1550‡	1531+0	*s*FMN, *r*N_3-H, *sc*NH_2	1533+0	*s*FMN, *r*N_3-H
1583‡	1577+1	*s*FMN, *sc*NH_2	1576+0	*s*FMN, *sc*NH_2, *sc*NH_2
1669‡	1654+0	*s*C_2=$O_{2'}$, *r*N_3-H	1652+0	*s*C_2=$O_{2'}$, *r*N_3-H, *s*C=O
1690‡	1692+0	*s*C=O	1691+0	*s*C=O
	10.5 (10.8)		10.5 (10.5)	
**1FMN***	**Excited State (S_1)**			
1375‡	1373+0<br>1380-97	*r*N_3-H<br>*sc*CH_2	1365+0	*s*FMN, *r*N-H
1413‡	1417+0<br>1410+126	*r*N_3-H, *r*N-H<br>*sc*CH_2, *s*C-N, *r*N-D(H)	1437+1	*sc*CH_2
1498‡	1503+0	*s*FMN, *r*$C_{6,9}$-H	1489#+0	*s*FMN, CH_3
1574‡	1587+0	*sc*NH_2, *r*N_3-H *s*C_4=$O_{4'}$	1562-1	*sc*NH_2, *r*N-H, *r*N-H
1622‡	1615+0	*sc*NH_2, *r*N_3-H *s*C_2=$O_{2'}$, *sc*NH_2	1629+0	*s*C_2=$O_{2'}$, *r*N_3-H, *sc*NH_2, *sc*NH_2
-	-	-	-	-
	6.6 (6.2)		12.4 (12.0)	
**3FMN***	**Excited State (T_1)**			
1438‡	1444+0	*r*N_3-H, *sc*CH_2	1447+0	*r*N_3-H, *sc*CH_2
1491‡	1485+0	*r*N_3-H, *s*FMN	1485+0	*r*N_3-H, *s*FMN
1567‡	1562+0	*sc*NH_2, $C_{6,9}$-H	1564+1	*s*FMN, *r*$C_{6,9}$-H, *sc*NH_2
1622‡	1640+0	*r*N_3-H, *s*C=O, *s*C_4=$O_{4'}$	1641+0	*s*C_4=$O_{4'}$, *r*N_3-H, *s*C=O
	8.8 (8.8)		9.3 (9.5)	
**3FMN***	**Excited State (T_{1-H})**			
1438‡	1419-3	*r*N_3-H/CH_2, *r*N_3-H	1410+146<br>1417-4	*s*C-N, *r*N-D(H)<br>*r*N_3-H
1491‡	1488+0	*r*N-H	1494-1<br>1525+0	*r*N-H<br>*s*FMN, *r*N-H
1567‡	1587-9	*r*N_5-H, *sc*H_2O, *s*FMN	1584#-8	*r*N_5-H, *s*FMN
1622‡	1644+0	*s*C=O, *sc*NH_2, *r*N_3-H, *s*C_4=$O_{4'}$	1643+0	*s*C=O, *sc*NH_2, *r*N_3-H, *s*C_4=$O_{4'}$
	16.0 (17.5)		17.3 (16.8)	
**A390**	**Adduct State (S_0')**			
1389‡	1373-8	*s*C-N/*r*HNCOH	1372+5	FMN, *sc*CH_2
1417‡	1408#+0	*r*N-H, *sc*CH_2	1407#+0	*r*N-H, *sc*CH_2
1435‡	1456-2	*r*N_3-H	1429+8	CH_3, *r*N_5-H/CH_3, CH_2
1541‡	1548+0<br>1561+0	*sc*NH_2, FMN<br>*sc*NH_2	1548-1<br>1561+0	*sc*NH_2, FMN<br>*sc*NH_2
1610‡	1620-2	*s*FMN	1621-1	*s*FMN
1634‡	1640+0	*s*C=O	1641+0	*s*C=O, *sc*NH_2
1665‡	1666-1	*r*N_3-H, *s*C_2=$O_{2'}$	1667-1	*r*N_3-H, *s*C_2=$O_{2'}$
1671‡	1682#-5	*s*C=O	1679#+0	*s*C=O, *s*C_2=$O_{2'}$
1695‡	1704+0	*s*C=O (side ch.)	1682-5	*s*C=O

1719†	1714+0	*s*C=O	1713+0	*s*C=O		1722‡	1727-1	*r*N_3-H, *s*C_4=$O_{4'}$	1704+0	*s*C=O (side ch.)
	1726-1	*s*C_4=$O_{4'}$, *r*N_3-H	1724+0	*s*C_4=$O_{4'}$, *r*N_3-H					1727-1	*r*N_3-H, *s*C_2=$O_{2'}$
MAD	11.3 (11.3)		9.4 (12.7)				9.5 (9.8)		8.6 (9.8)	

FMN: ring breathing, *r*: rocking, *s*: stretch, *sc*: scissoring
#: Relatively intense ES peak in proximity to the experimental, offset by the GS signal
(): Normal mode only in non-deuterated spectrum
†: This study
‡: Taken from ref.[32]

3. Cartesian coordinates of all optimised ground–state and excited–state species

EL222 **Qa** S_0

C	15.46499756	-9.61300565	29.59100173
C	14.06130527	-9.05222260	29.74087843
O	13.31016706	-9.35450695	30.66707599
H	16.07699962	-8.80499893	29.19000312
H	15.81200321	-9.88599745	30.58800236
H	15.48128405	-10.48194612	28.91669852
N	13.70631301	-8.11759630	28.80144012
C	12.31901008	-7.66555013	28.76126249
C	11.43801547	-8.87870850	28.45700701
O	11.68880273	-9.59541685	27.48416634
C	12.14085975	-6.59058693	27.68952124
H	14.18162282	-8.14442596	27.90052585
H	12.06805655	-7.26718825	29.75634777
H	11.07497157	-6.35491017	27.55867587
H	12.68906156	-5.67836886	27.97053330
H	12.53086492	-6.95850307	26.72928647
N	10.40424613	-9.11266813	29.28942383
C	9.57603150	-10.29300942	29.12699171
H	10.40049558	-8.63210539	30.18128858
H	9.58399017	-10.54799533	28.06700062
H	9.94598710	-11.13799960	29.70801256
H	8.54468992	-10.07159563	29.43957528
C	-2.49301852	-7.05790373	23.10698713
C	-1.19322721	-6.32685504	22.90066230
O	-1.16948544	-5.10201773	22.69946736
H	-2.34441762	-8.04129519	23.57918808
H	-3.09800662	-6.43603077	23.76702441
H	-3.02497673	-7.22205012	22.16999695
N	-0.07543858	-7.08587233	22.94639316
C	1.25910271	-6.52817603	22.72903906
C	1.51953334	-6.36353422	21.21022029
O	2.45034321	-6.93441969	20.62980492
C	2.30529660	-7.40829352	23.41183297
S	3.95942207	-6.62329067	23.58314389
H	-0.15355056	-8.10431300	23.03306557
H	1.26841329	-5.52238286	23.17804754
H	1.97899481	-7.61563903	24.44154945
H	2.41468810	-8.36383872	22.88134860
H	4.22219647	-6.59421317	22.25392843
N	0.60989330	-5.55380736	20.62475599
C	0.69799297	-5.07509283	19.26898620
H	-0.06428191	-5.10573202	21.25326023
H	1.59902440	-5.48295777	18.81201122
H	-0.16301524	-5.28496126	18.63400906
H	0.81144457	-3.97559505	19.27521155
C	8.88998299	-1.76498056	16.94499816
C	8.76371283	-2.65387269	18.18652974
C	8.08837090	-4.01319396	18.04262200
O	8.17683473	-4.83796477	18.97796851
N	7.37293988	-4.28800456	16.94550469
H	7.93503746	-1.71708790	16.39202418
H	9.74749192	-2.84713824	18.64247188
H	8.17590301	-2.13868294	18.96707428
H	7.26162083	-3.60945048	16.20399560
H	6.83338737	-5.16405150	16.91796207
H	9.10001842	-0.73100172	17.21899392
H	9.65300235	-2.12300787	16.25400703
C	13.21299376	-5.73700863	18.45901888
C	11.77435752	-5.51700345	18.92348913
C	11.38571747	-6.45493562	20.07318576
O	12.20919500	-7.18119748	20.64660080
N	10.07586201	-6.41809705	20.39026607
H	13.87501080	-5.50899319	19.29499167
H	11.62127839	-4.47903773	19.27086600
H	11.05248360	-5.66820786	18.10264917
H	9.43470990	-5.77123555	19.92933522
H	9.70226690	-7.03397587	21.10094667
H	13.32599935	-6.78000005	18.16399907
H	13.46390548	-5.08529868	17.60857791
C	10.79999583	-11.64399497	25.39899985
C	11.24722125	-10.75324450	24.24884188
O	11.17537819	-11.09888223	23.06535782
H	10.50800191	-11.02000381	26.24400447
H	11.57900158	-12.34599997	25.69500208
H	9.94487101	-12.23922550	25.04424366
N	11.69194033	-9.53690837	24.65395035
C	12.13658480	-8.50396549	23.74494449
C	13.60167313	-8.16887892	24.05272128
O	14.01641638	-8.07616278	25.21492962
C	11.26775502	-7.23503025	23.85509459
C	9.79551190	-7.49655425	23.53179888
C	8.91835377	-7.93026126	24.70591064
O	9.26277011	-7.83434238	25.88399498
N	7.69954469	-8.40741165	24.32733993
H	11.77269812	-9.35302171	25.65700133
H	12.04760946	-8.89909749	22.72392202
H	11.34624206	-6.81804059	24.86959865
H	11.67041298	-6.49971878	23.14298108
H	9.32907639	-6.57554766	23.14367129
H	9.69731528	-8.25038672	22.73318574
H	7.36481584	-8.29804135	23.36913445
H	7.00578813	-8.53975973	25.05492399
N	14.39310163	-7.97107146	22.97135704
C	15.77100060	-7.52000485	23.12301607
H	13.94901476	-7.94083375	22.05613055
H	15.82100208	-6.93799650	24.04299918
H	16.49000251	-8.33799990	23.16499284
H	16.03608263	-6.87544130	22.27184044
O	7.23726730	-7.48142021	21.41050694
C	6.43320157	-7.72532282	20.51324554
N	6.45823256	-7.08815901	19.30114721
C	5.60449248	-7.34841352	18.21459350
O	5.75856741	-6.70207002	17.17595471
N	4.62421289	-8.30533818	18.32935236
C	4.47659003	-8.92634959	19.47834148
C	5.32880431	-8.72480095	20.64198515
N	5.18695122	-9.34412756	21.77809241
C	4.15249788	-10.22975416	21.89480954
C	3.97565531	-10.89117371	23.13156609
C	2.96056954	-11.80938175	23.33733296
C	2.79794148	-12.50622696	24.66377236
C	2.06998361	-12.08700284	22.25405686
C	0.94588851	-13.07221880	22.44172941
C	2.21743443	-11.42770414	21.03370904
C	3.25866848	-10.49878756	20.81708604
N	3.46367514	-9.85643755	19.60750295
C	2.59505136	-10.10380977	18.42523742
C	1.56094945	-8.98809246	18.23480108
H	7.15014848	-6.32649065	19.16365247
H	4.68510694	-10.64377562	23.92323288
H	1.79473483	-12.34117906	25.09125296
H	2.91593605	-13.59745918	24.55798586
H	3.54158395	-12.15372622	25.39144018
H	0.38724046	-13.23303513	21.50961078
H	1.32552249	-14.04559490	22.79062137
H	0.23767957	-12.70926015	23.20486190
H	1.50934473	-11.65359308	20.23866557
H	2.16270142	-11.10484045	18.51855624
H	3.27153301	-10.11139907	17.56194544
H	2.14197889	-8.07300111	18.34611627
H	1.14185391	-9.04663232	17.21921072
H	0.74306622	-8.98692620	18.95507635
O	0.03329284	-9.94078661	23.22820523
H	0.72099654	-10.09703180	23.89499503
H	0.45440004	-10.25352475	22.41024368

EL222 **Qb** S_0

C	15.46501185	-9.61300888	29.59101321
C	14.05061880	-9.08687307	29.74069777
O	13.28015847	-9.46071621	30.62433583
H	16.07697268	-8.80499723	29.18996422
H	15.81198649	-9.88598718	30.58801014
H	15.49408717	-10.48647106	28.92254466
N	13.69893792	-8.10456674	28.85309681
C	12.31789013	-7.64076437	28.83447676
C	11.42358428	-8.83360418	28.47872809
O	11.65916246	-9.51458879	27.48003600
C	12.16816122	-6.50808910	27.81942907
H	14.19832154	-8.05195144	27.96735544
H	12.06920704	-7.28777674	29.84753416
H	11.12315438	-6.17637534	27.75400599
H	12.79850092	-5.65726193	28.12017177
H	12.48869092	-6.85445190	26.82575009
N	10.37057876	-9.08496883	29.29396316
C	9.57606135	-10.29298899	29.12696054
H	10.38711714	-8.65131284	30.21065093
H	9.58399843	-10.54799909	28.06700131
H	9.94596297	-11.13801283	29.70800959
H	8.53875329	-10.09400215	29.43696871
C	-2.49259357	-7.05784308	23.10670905
C	-1.17692053	-6.34906657	22.91892352
O	-1.11508485	-5.11545684	22.80300862
H	-2.35690007	-8.04352191	23.57837447
H	-3.09807856	-6.43632441	23.76723539
H	-3.02520825	-7.22188724	22.17010051
N	-0.08425038	-7.14770204	22.90083444
C	1.25911419	-6.61300462	22.69307972
C	1.51903303	-6.39943287	21.18285087
O	2.44833010	-6.95104734	20.58411338
C	2.29064988	-7.53529352	23.33864215
S	3.94308001	-6.76492284	23.57278810
H	-0.21622817	-8.15706832	22.76736557
H	1.28657740	-5.62516876	23.17816766
H	1.94867858	-7.80510227	24.34805574
H	2.40922987	-8.45723512	22.75258829
H	4.24742855	-6.70736143	22.25347715
N	0.60715057	-5.57394726	20.61825060
C	0.69863062	-5.07631404	19.26819429
H	-0.04987473	-5.12171176	21.25938710
H	1.59887039	-5.48332740	18.81203673
H	-0.16358858	-5.28352765	18.63431117
H	0.81243653	-3.97723333	19.28574821
C	8.88997278	-1.76463053	16.94540399
C	8.74480004	-2.67512828	18.16846358
C	8.12296168	-4.05179789	17.95073646
O	8.38231532	-4.98310120	18.74102204
N	7.26373389	-4.22933330	16.93753025
H	7.94368072	-1.70690141	16.37823213
H	9.71508949	-2.84937287	18.65711972
H	8.10573559	-2.19075695	18.92914048
H	7.00894462	-3.46659215	16.32455588
H	6.75966832	-5.12426875	16.86106636
H	9.10032132	-0.73096528	17.21862598
H	9.65281279	-2.12330135	16.25395062
C	13.21281612	-5.73702852	18.45919901
C	11.77887563	-5.52935662	18.95144816
C	11.43336702	-6.43426559	20.14292539
O	12.26575495	-7.18369413	20.67168872
N	10.15465941	-6.32784873	20.56656353
H	13.87517268	-5.50892709	19.29484487
H	11.61161064	-4.48338749	19.26566662
H	11.03548394	-5.72052597	18.15909984
H	9.48549637	-5.76394089	20.04199314
H	9.77744229	-7.00722707	21.21747308
H	13.32594913	-6.78002513	18.16406903
H	13.44742558	-5.08121954	17.60783378
C	10.79996720	-11.64384637	25.39901229
C	11.24364576	-10.74933600	24.25323834
O	11.08851650	-11.05031244	23.06799047
H	10.50804515	-11.02005054	26.24405364
H	11.57898618	-12.34604188	25.69494355
H	9.93872340	-12.22728720	25.04460847
N	11.79866183	-9.57714558	24.66237438
C	12.13027986	-8.49751773	23.75485693
C	13.57632903	-8.08099512	24.04922056
O	13.95664290	-7.88385183	25.21139990
C	11.17468677	-7.29780089	23.92196095
C	9.68659912	-7.70001058	23.89052740
C	9.14897081	-8.28184334	25.17764141
O	9.47460061	-7.49761605	26.25192308
N	8.44427552	-9.34452365	25.20732333
H	11.90674661	-9.39985168	25.66233946
H	12.03505330	-8.88305855	22.73150137
H	11.39095744	-6.77854444	24.86717888
H	11.37896633	-6.59437721	23.10122868
H	9.08404277	-6.79927121	23.68100750
H	9.47345912	-8.42342588	23.09110589
H	8.13451907	-9.55441535	26.16651948
N	14.39239565	-7.96905916	22.97586526
C	15.77096467	-7.51993616	23.12315270
H	13.96508301	-7.99969365	22.05286727
H	15.82101870	-6.93803228	24.04302061
H	16.48999129	-8.33801657	23.16485891
H	16.03735779	-6.87486650	22.27227654
O	7.38105719	-7.49247398	21.16958762
C	6.55167531	-7.74697262	20.30683386
N	6.55322072	-7.14533942	19.07016050
C	5.63445344	-7.37465028	18.03739982
O	5.74045119	-6.72078596	16.99513585
N	4.63900957	-8.31204195	18.20075027
C	4.53283178	-8.92498944	19.35988853
C	5.43080365	-8.72212892	20.48956046
N	5.31865544	-9.32069105	21.63930493
C	4.27148684	-10.18179912	21.80922607
C	4.11657214	-10.79963499	23.07069081
C	3.07593444	-11.67194770	23.33903644
C	2.92173705	-12.30629861	24.69681175
C	2.14563068	-11.95674158	22.29379214
C	0.99361668	-12.89386395	22.54721418
C	2.28057550	-11.34892409	21.04379425
C	3.33805295	-10.45480404	20.76906823
N	3.51624131	-9.84353357	19.53877114
C	2.60208213	-10.10152319	18.39628783
C	1.56130718	-8.98791158	18.23450786
H	7.27553295	-6.42150358	18.89857292
H	4.85900959	-10.54473734	23.82916617
H	1.93802259	-12.07515991	25.13818267
H	2.99135253	-13.40531794	24.63655180
H	3.69912453	-11.95442278	25.38872656
H	0.40551377	-13.07038219	21.63599138
H	1.34893065	-13.86624694	22.92364198
H	0.31733970	-12.47691615	23.31124473
H	1.55293269	-11.58979774	20.27062798
H	2.17621941	-11.10321286	18.51378301
H	3.24282654	-10.11415973	17.50638188
H	2.14170559	-8.07292274	18.34689876
H	1.13365233	-9.03448426	17.22137188
H	0.74296055	-8.98716258	18.95495597
O	-0.35658376	-9.91772205	22.22791238
H	0.53292555	-10.22422574	21.97787719
H	-0.87429540	-10.03421290	21.41565383
H	9.15158949	-7.91746374	27.06707301

EL222 **Qc** S_0

C	15.46501111	-9.61300369	29.59103311
C	14.05308699	-9.08052385	29.75070245
O	13.29385279	-9.44152026	30.64899411
H	16.07697014	-8.80499861	29.18995809
H	15.81199612	-9.88599470	30.58800522
H	15.48914699	-10.48563285	28.92149281
N	13.69448748	-8.10470920	28.85815633
C	12.31463833	-7.63536819	28.85188463
C	11.41299481	-8.81826572	28.47791745
O	11.63920951	-9.46713561	27.45567702
C	12.16353018	-6.48833570	27.85454342
H	14.18269842	-8.06412368	27.96565445
H	12.07444124	-7.29493153	29.87104477
H	11.12355152	-6.13353553	27.83674285
H	12.81825690	-5.65235609	28.14453472
H	12.44659143	-6.82889407	26.84712318
N	10.38465424	-9.09712260	29.30984340
C	9.57603971	-10.29299672	29.12699012
H	10.42277786	-8.68719920	30.23641440
H	9.58401668	-10.54799575	28.06700099
H	9.94595773	-11.13801579	29.70800811
H	8.53934904	-10.08806410	29.43446316
C	-2.49259318	-7.05785351	23.10671868
C	-1.17853059	-6.34543583	22.91946227
O	-1.12192849	-5.11206391	22.79810177
H	-2.35527435	-8.04328632	23.57831404
H	-3.09807922	-6.43632245	23.76723269
H	-3.02520522	-7.22188052	22.17009760
N	-0.08167587	-7.13903147	22.90941447
C	1.25858786	-6.59703654	22.69881139
C	1.51681227	-6.39347798	21.18705000
O	2.44414468	-6.95009969	20.58962179
C	2.29826178	-7.50485112	23.35118349
S	3.94768872	-6.71959780	23.56208671
H	-0.20945022	-8.14906039	22.77596739
H	1.27842310	-5.60528149	23.17605683
H	1.96484760	-7.76233623	24.36661504
H	2.41908147	-8.43407959	22.77734923
H	4.23657851	-6.67105828	22.23892308
N	0.60593581	-5.56961002	20.61927181
C	0.69863105	-5.07630536	19.26818540
H	-0.05194373	-5.11561867	21.25835996
H	1.59887112	-5.48332740	18.81203937
H	-0.16359001	-5.28353238	18.63431435
H	0.81260929	-3.97711755	19.28329214
C	8.88991065	-1.76458513	16.94537721
C	8.74943600	-2.66980431	18.17298143
C	8.11941202	-4.04523248	17.97307134
O	8.34612473	-4.95495713	18.79793897
N	7.28858146	-4.24393994	16.94083470
H	7.94169726	-1.70902894	16.38129231
H	9.72267181	-2.84491749	18.65592880
H	8.11910409	-2.17914448	18.93683159
H	7.06002959	-3.49738329	16.29830308
H	6.77863753	-5.13667092	16.87683976
H	9.10036566	-0.73097805	17.21863926
H	9.65281168	-2.12331716	16.25395718
C	13.21285094	-5.73699942	18.45915932
C	11.77684185	-5.53376186	18.94839676
C	11.42460274	-6.45513719	20.12544873
O	12.25316545	-7.21342807	20.64658714
N	10.14356114	-6.35334761	20.54464305
H	13.87515500	-5.50895641	19.29486741
H	11.60805642	-4.49173815	19.27479736
H	11.03817285	-5.71539761	18.14917428
H	9.47905649	-5.77153480	20.03353673
H	9.76220363	-7.03952096	21.18631830
H	13.32594797	-6.78002450	18.16406633
H	13.44727318	-5.08035588	17.60842450
C	10.79998779	-11.64383484	25.39904181
C	11.23978240	-10.75379352	24.25026992
O	11.07522849	-11.05410209	23.06524394
H	10.50803075	-11.02005080	26.24404887
H	11.57897983	-12.34605389	25.69492984
H	9.93711957	-12.22752824	25.04942864
N	11.81123514	-9.58548684	24.65130362
C	12.12979249	-8.50923769	23.73549815
C	13.56277289	-8.05779647	24.03955461
O	13.91905607	-7.81221039	25.20003778
C	11.14691581	-7.32783371	23.87417090
C	9.66917109	-7.77770877	23.84460476

C	9.12526906	-8.29229332	25.17702153
O	8.65751903	-9.41633692	25.33312675
N	9.18845198	-7.39808331	26.20876317
H	11.89645226	-9.39337031	25.65125272
H	12.05238253	-8.90767027	22.71558647
H	11.36124329	-6.78644951	24.80926668
H	11.34197166	-6.63406768	23.04311221
H	9.03542879	-6.92784667	23.53880318
H	9.51137091	-8.57741694	23.10762759
H	9.42185016	-6.42548267	26.05987889
H	8.80044245	-7.66761641	27.10534813
N	14.39448299	-7.97338005	22.97568460
C	15.77098012	-7.51994955	23.12314466
H	13.98318754	-8.04797568	22.04839927
H	15.82101515	-6.93801952	24.04301273
H	16.48999420	-8.33801360	23.16486769
H	16.03753591	-6.87407877	22.27267453
O	7.34562227	-7.49068070	21.22750967
C	6.52504600	-7.74544907	20.35581949
N	6.53554032	-7.13868401	19.12174935
C	5.63315754	-7.37446936	18.07556847
O	5.75195545	-6.72242440	17.03352570
N	4.63976576	-8.31515674	18.22667191
C	4.52154975	-8.92860219	19.38452031
C	5.40624062	-8.72564218	20.52440737
N	5.28244754	-9.32717463	21.67186441
C	4.23489517	-10.19040465	21.82859422
C	4.06548098	-10.81165874	23.08684754
C	3.02305386	-11.68600762	23.34125898
C	2.85445158	-12.32409505	24.69550262
C	2.10416819	-11.96811111	22.28525011
C	0.94807289	-12.90376015	22.52430816
C	2.25247075	-11.35652260	21.03868410
C	3.31299730	-10.46203160	20.77762265
N	3.50411841	-9.84815778	19.55080606
C	2.60057768	-10.10217763	18.39923609
C	1.56126911	-8.98791365	18.23446532
H	7.25294460	-6.40838459	18.95883293
H	4.79628358	-10.55797662	23.85679880
H	1.86594426	-12.09421855	25.12668370
H	2.92468159	-13.42290594	24.63241480
H	3.62437661	-11.97453146	25.39676910
H	0.36946726	-13.07714274	21.60642625
H	1.29747327	-13.87748534	22.90269005
H	0.26473274	-12.48659363	23.28194049
H	1.53163062	-11.59335106	20.25793805
H	2.17372929	-11.10428609	18.50931300
H	3.24879610	-10.11123121	17.51476076
H	2.14171716	-8.07293157	18.34691130
H	1.13433736	-9.03531740	17.22107041
H	0.74297405	-8.98715962	18.95497136
O	-0.36415528	-9.90635093	22.22972481
H	0.52153730	-10.22218185	21.97725980
H	-0.88484814	-10.01659199	21.41849276

EL222 **Qd** S_0

C	15.46501111	-9.61300369	29.59103115
C	14.05253045	-9.07778668	29.74028914
O	13.29016707	-9.42565457	30.64154817
H	16.07697014	-8.80499861	29.18995809
H	15.81199511	-9.88599470	30.58800522
H	15.49155734	-10.48509764	28.92072265
N	13.69819718	-8.11782861	28.83018365
C	12.31734867	-7.64979637	28.79845482
C	11.42100042	-8.84489423	28.46081063
O	11.64922160	-9.53400557	27.46416402
C	12.17167369	-6.53671912	27.76138801
H	14.19451141	-8.08996330	27.94150365
H	12.06631016	-7.27820688	29.80420830
H	11.11803062	-6.25176738	27.64060674
H	12.76249311	-5.66069582	28.07052912
H	12.54146762	-6.88978158	26.78715645
N	10.38763806	-9.09918211	29.29438571
C	9.57603971	-10.29299672	29.12698912
H	10.40448442	-8.65338763	30.20452190
H	9.58401568	-10.54799575	28.06700099
H	9.94595873	-11.13801579	29.70800811
H	8.54164768	-10.08447818	29.44055267
C	-2.49259418	-7.05784949	23.10671969
C	-1.17639297	-6.34957775	22.91938157
O	-1.11392720	-5.11608108	22.80376465
H	-2.35726673	-8.04360515	23.57832716
H	-3.09808022	-6.43632245	23.76723169
H	-3.02520422	-7.22188253	22.17009760
N	-0.08379705	-7.14873060	22.90151216
C	1.25955531	-6.61425004	22.69238226
C	1.51792028	-6.40078423	21.18187941
O	2.44557973	-6.95423976	20.58201582
C	2.29211779	-7.53707579	23.33561631
S	3.94617808	-6.76746945	23.56413351
H	-0.21675867	-8.15773540	22.76648369
H	1.28795498	-5.62662850	23.17782073
H	1.95301820	-7.80605304	24.34621330
H	2.40796402	-8.45907407	22.74912943
H	4.24051740	-6.70095124	22.24287826
N	0.60715764	-5.57379258	20.61826970
C	0.69863105	-5.07630437	19.26818540
H	-0.04895465	-5.12090900	21.25990188
H	1.59887112	-5.48332740	18.81203937
H	-0.16359001	-5.28353338	18.63431435
H	0.81236223	-3.97726155	19.28585161
C	8.88991166	-1.76458813	16.94537419
C	8.74776132	-2.67148921	18.17188217
C	8.11566745	-4.04574099	17.96887486
O	8.34658564	-4.96101834	18.78638645
N	7.27818286	-4.23813149	16.94066436
H	7.94224649	-1.70810614	16.38039572
H	9.72051102	-2.84875611	18.65492151
H	8.11786994	-2.18111712	18.93626331
H	7.04468377	-3.48764743	16.30455145
H	6.76738293	-5.12998239	16.87633471
H	9.10035667	-0.73097505	17.21863423
H	9.65282068	-2.12331615	16.25396618
C	13.21284993	-5.73699741	18.45915932
C	11.77806991	-5.52812893	18.94620549
C	11.42473154	-6.44469001	20.12577058
O	12.25400113	-7.20039216	20.65117654
N	10.14339932	-6.34356678	20.53945611
H	13.87515299	-5.50896038	19.29487038
H	11.61296438	-4.48476099	19.26998391
H	11.03880119	-5.70977045	18.14761326
H	9.48077510	-5.76049526	20.02735223
H	9.76207943	-7.02015011	21.19030232
H	13.32594696	-6.78002349	18.16406231
H	13.45038543	-5.08159419	17.60817986
C	10.79998779	-11.64383785	25.39903980
C	11.24996689	-10.75305368	24.25087017
O	11.14543702	-11.08363404	23.06753903
H	10.50802975	-11.02004980	26.24404787
H	11.57897983	-12.34605289	25.69493085
H	9.94365618	-12.23389218	25.04123459
N	11.74312913	-9.55365101	24.65692973
C	12.13551326	-8.50280861	23.74140752
C	13.59106492	-8.12986184	24.04399314
O	13.99001186	-7.99465615	25.20816589
C	11.22178238	-7.26593997	23.87005131
C	9.72989836	-7.63430618	23.79187410
C	9.16689748	-8.16377834	25.09436653
O	8.52078005	-9.35198140	24.91669765
N	9.28713396	-7.52038872	26.18949943
H	11.85203465	-9.37380853	25.65757009
H	12.04237262	-8.89987924	22.72199931
H	11.41144369	-6.75086869	24.82347895
H	11.47712887	-6.57670031	23.05221475
H	9.14610547	-6.73615496	23.53482715
H	9.54085455	-8.37673213	23.00274662
H	8.86023339	-8.02273350	26.97849419
N	14.39356095	-7.96716645	22.96695873
C	15.77098012	-7.51994955	23.12314365
H	13.95805374	-7.95952947	22.04701108
H	15.82101415	-6.93801952	24.04301273
H	16.48999420	-8.33801360	23.16486864
H	16.04072992	-6.87463695	22.27362086
O	7.37090466	-7.53716205	21.21009879
C	6.54063879	-7.77525699	20.34343960
N	6.54242545	-7.15434909	19.11613097
C	5.63297344	-7.37953861	18.07362385
O	5.74558077	-6.71986620	17.03621477
N	4.63881332	-8.32037708	18.22285401
C	4.52439951	-8.94120111	19.37644517
C	5.41612555	-8.74975986	20.51306636
N	5.29392571	-9.35665060	21.65779211
C	4.24089865	-10.21389118	21.81475418
C	4.06981853	-10.83771945	23.07124507
C	3.02343054	-11.70711391	23.32601561
C	2.85282920	-12.34667732	24.67934557
C	2.10122622	-11.98216145	22.27103308
C	0.94121386	-12.91310161	22.50941901
C	2.25045007	-11.36751261	21.02632257
C	3.31474956	-10.47707499	20.76585274
N	3.50511210	-9.85920843	19.54133156
C	2.59781334	-10.10504905	18.39142612
C	1.56128011	-8.98791465	18.23447331
H	7.25921244	-6.42302549	18.95399276
H	4.80293741	-10.58864307	23.84033350
H	1.86636335	-12.11173241	25.11234453
H	2.91639538	-13.44579069	24.61480531
H	3.62518044	-12.00253880	25.38084457
H	0.36014284	-13.08058367	21.59204326
H	1.28661934	-13.88988396	22.88362570
H	0.26109930	-12.49524558	23.26950741
H	1.52662166	-11.59766746	20.24636319
H	2.16869159	-11.10662272	18.49760230
H	3.24357916	-10.11151565	17.50512667
H	2.14171516	-8.07292956	18.34690935
H	1.13200212	-9.03163787	17.22174521
H	0.74297205	-8.98716063	18.95496935
O	-0.36734473	-9.91694204	22.22480950
H	0.51888783	-10.23074330	21.97237745
H	-0.88876247	-10.03244201	21.41478815
H	8.14622665	-9.63751306	25.76670872

EL222 **Qa** S_1

C	15.46499708	-9.61300824	29.59099676
C	14.06013663	-9.05507598	29.74176179
O	13.30946748	-9.36060540	30.66727338
H	16.07700078	-8.80499766	29.19000756
H	15.81200263	-9.88599650	30.58800273
H	15.48249179	-10.48231083	28.91714785
N	13.70318229	-8.11869154	28.80486601
C	12.31803618	-7.65988460	28.77197003
C	11.43010200	-8.86552527	28.45663807
O	11.66968081	-9.56469838	27.46867748
C	12.14405164	-6.57427118	27.71058641
H	14.17782920	-8.14021064	27.90357439
H	12.07176667	-7.26905757	29.77116113
H	11.08256839	-6.30421732	27.61196952
H	12.72345268	-5.67929862	27.98433806
H	12.50120993	-6.94543848	26.73875546
N	10.40370600	-9.11224596	29.29472666
C	9.57602870	-10.29301074	29.12699420
H	10.41152935	-8.64732723	30.19496675
H	9.58398827	-10.54799485	28.06700078
H	9.94598958	-11.13799886	29.70801171
H	8.54414571	-10.07312685	29.43914977
C	-2.49318114	-7.05766592	23.10709784
C	-1.18878654	-6.33491341	22.89934227
O	-1.15826984	-5.11028863	22.69740886
H	-2.34882229	-8.04173980	23.57973435
H	-3.09804175	-6.43608744	23.76704584
H	-3.02484570	-7.22216749	22.16994244
N	-0.07575065	-7.10148274	22.93937341
C	1.26205644	-6.55337577	22.71477027
C	1.50993224	-6.38945417	21.19435539
O	2.41732085	-6.98062898	20.59908692
C	2.30888783	-7.43871105	23.39099862
S	3.97937333	-6.67765458	23.50307673
H	-0.15600460	-8.11625135	23.06164828
H	1.28235921	-5.54727445	23.16316850
H	2.00330596	-7.62002798	24.43194832
H	2.39359062	-8.40708055	22.88051599
H	4.26603965	-6.80316584	22.18370391
N	0.61470669	-5.55315483	20.62431259
C	0.69803550	-5.07506684	19.26900986
H	-0.04916538	-5.10005221	21.26014848
H	1.59901174	-5.48297809	18.81200540
H	-0.16301418	-5.28497181	18.63401166
H	0.81085744	-3.97561444	19.27545328
C	8.89002173	-1.76499987	16.94500864
C	8.75272299	-2.65269992	18.18653186
C	8.03868751	-3.99344181	18.04989602
O	8.16146605	-4.84452725	18.95592579
N	7.24874552	-4.21995306	16.99275805
H	7.93896750	-1.71110051	16.38584969
H	9.73481389	-2.87235949	18.63317000
H	8.18524966	-2.12146224	18.97172014
H	7.10747859	-3.51875039	16.27809692
H	6.69133878	-5.08368758	16.97260539
H	9.09998201	-0.73099649	17.21900132
H	9.65299875	-2.12300131	16.25399925
C	13.21300837	-5.73701127	18.45901174
C	11.77346850	-5.51609020	18.91994385
C	11.36846745	-6.47506140	20.04727216
O	12.18588766	-7.20332968	20.62739542
N	10.05186716	-6.45210929	20.33329357
H	13.87500048	-5.50899134	19.29499918
H	11.62618635	-4.48425821	19.28769389
H	11.05639957	-5.64447241	18.09120047
H	9.42052367	-5.79836435	19.86888259
H	9.65590568	-7.08521806	21.01668292
H	13.32599020	-6.78000010	18.16399463
H	13.46603944	-5.08499607	17.60935887
C	10.79999377	-11.64401386	25.39899308
C	11.25306580	-10.76026632	24.24573149
O	11.19350278	-11.11533282	23.06457993
H	- 10.50800249	-11.01999609	26.24399945
H	- 11.57900375	-12.34599420	25.69501038
H	9.94487514	-12.23935504	25.04449851
N	11.69244173	-9.53987743	24.64507795
C	12.13377301	-8.50859781	23.73312833
C	13.59419628	-8.15753855	24.04891602
O	13.99755269	-8.04512241	25.21324980
C	11.24917217	-7.25003433	23.83083943
C	9.78937312	-7.52908199	23.46801000
C	8.88366964	-7.99369289	24.60908205
O	9.20281607	-7.91296531	25.79863117
N	7.68411727	-8.47208325	24.19629538
H	11.75556278	-9.34485465	25.64724388
H	12.05630226	-8.91015116	22.71365757
H	11.30145456	-6.83637135	24.84880865
H	11.65650498	-6.50585158	23.13073317
H	9.31616679	-6.61364450	23.07662135
H	9.72262969	-8.27763718	22.66227964
H	7.37321188	-8.35847461	23.22668406
H	6.97900795	-8.64524217	24.90405301
N	14.39314872	-7.97153862	22.97108081
C	15.77100303	-7.52000337	23.12301189
H	13.95655125	-7.96100011	22.05196264
H	15.82099949	-6.93799708	24.04299924
H	16.49000193	-8.33799974	23.16499390
H	16.03629663	-6.87505897	22.27214499
O	7.28118975	-7.55375974	21.38875014
C	6.43395644	-7.80951465	20.51927408
N	6.44907514	-7.12001501	19.30456745
C	5.57809718	-7.32989930	18.25967310
O	5.62006337	-6.64320509	17.23139873
N	4.63828544	-8.35150228	18.36225009
C	4.49789935	-8.96970381	19.53656986
C	5.36426580	-8.78378516	20.65706596
N	5.23966269	-9.47035370	21.81996038
C	4.17461920	-10.30489850	21.90443347
C	3.95227144	-11.00613958	23.10827917
C	2.88024488	-11.88403151	23.29242555
C	2.71638109	-12.62354021	24.58950889
C	1.94386075	-12.07785617	22.23067977
C	0.77716704	-13.01776345	22.39617313
C	2.13152551	-11.38084121	21.03277975
C	3.22905412	-10.51910590	20.83071336
N	3.45937563	-9.87693449	19.62137211
C	2.59942457	-10.10142577	18.43067318
C	1.56105690	-8.98825227	18.23447590
H	7.12795976	-6.34633490	19.19019483
H	4.67428271	-10.83853634	23.90925688
H	1.72657483	-12.43418757	25.03905761
H	2.77329229	-13.71411012	24.42890206
H	3.48917794	-12.34263726	25.31735965
H	0.17549095	-13.07406351	21.47816850
H	1.11633214	-14.03715833	22.64448214
H	0.11308629	-12.69212121	23.21419331
H	1.40329976	-11.52656133	20.23653388
H	2.16617363	-11.10334489	18.51388710
H	3.27843098	-10.10085214	17.57009217
H	2.14180724	-8.07293999	18.34622258
H	1.14428573	-9.05546201	17.21839050
H	0.74317250	-8.98687429	18.95519716
O	0.05797187	-9.91322711	23.47505520
H	0.60234099	-9.95361974	24.27740592
H	0.63006542	-10.31870389	22.80065108

EL222 **Qb** S_1

C	15.46501216	-9.61300856	29.59101263
C	14.05050693	-9.08666024	29.74091558
O	13.28064288	-9.45985699	30.62537942
H	16.07697327	-8.80499771	29.18996417
H	15.81198585	-9.88598660	30.58801035
H	15.49399277	-10.48643027	28.92249259
N	13.69827327	-8.10509322	28.85281222
C	12.31750139	-7.64004336	28.83473934
C	11.42217873	-8.83185573	28.47796438
O	11.65602195	-9.51044554	27.47730762
C	12.16848969	-6.50660100	27.82041641
H	14.19761276	-8.05223630	27.96708762
H	12.06942601	-7.28759798	29.84814182
H	11.12414293	-6.17240239	27.75702844
H	12.80118268	-5.65727553	28.12054214
H	12.48624586	-6.85312309	26.82589905
N	10.37084610	-9.08517521	29.29467898
C	9.57606246	-10.29298846	29.12695927
H	10.38900519	-8.65319464	30.21211353
H	9.58399806	-10.54799893	28.06700105
H	9.94596281	-11.13801272	29.70800917
H	8.53870402	-10.09398067	29.43681594
C	-2.49258835	-7.05784324	23.10670730
C	-1.17048938	-6.35865770	22.91937136
O	-1.09830211	-5.12484887	22.81203294
H	-2.36187220	-8.04451237	23.57809046
H	-3.09807932	-6.43632134	23.76723180
H	-3.02520632	-7.22189195	22.17010030
N	-0.08451803	-7.16711215	22.88977559
C	1.26313594	-6.64526094	22.67306216
C	1.50992936	-6.42426430	21.16288612
O	2.41986686	-6.98873010	20.54620084
C	2.29758328	-7.57817164	23.29867291
S	3.95828041	-6.81800122	23.50326765
H	-0.22694349	-8.17441274	22.75121306
H	1.30526580	-5.66088733	23.16395190

H 1.96964016 -7.85139835 24.31187213
H 2.40244344 -8.49824403 22.70621390
H 4.28115201 -6.84716656 22.18657978
N 0.60930364 -5.57579817 20.61626973
C 0.69862997 -5.07631600 19.26819651
H -0.03614710 -5.11925359 21.26613120
H 1.59887117 -5.48332619 18.81203731
H -0.16358716 -5.28353099 18.63431001
H 0.81179437 -3.97735806 19.28708110
C 8.88996490 -1.76463913 16.94540912
C 8.74046184 -2.66938969 18.17386039
C 8.06842939 -4.02523306 17.97356056
O 8.35085753 -4.98114611 18.72452695
N 7.13723676 -4.15256722 17.01673078
H 7.94614933 -1.70625114 16.37485921
H 9.71397171 -2.87301503 18.64369655
H 8.13152562 -2.16143221 18.94398025
H 6.86070773 -3.36634873 16.44412946
H 6.60428442 -5.02938425 16.95141422
H 9.10032179 -0.73096548 17.21862773
H 9.65281137 -2.12330116 16.25394866
C 13.21281236 -5.73702927 18.45920065
C 11.77777309 -5.52779279 18.94819663
C 11.42268119 -6.44133556 20.13075035
O 12.25248631 -7.19178864 20.66208135
N 10.13968968 -6.34006632 20.54255328
H 13.87517151 -5.50892974 19.29484619
H 11.61415244 -4.48363821 19.27053092
H 11.03716016 -5.70870399 18.15075831
H 9.47577776 -5.77451756 20.01337550
H 9.75160574 -7.02750355 21.17949160
H 13.32594977 -6.78002461 18.16406643
H 13.44929770 -5.08139030 17.60811980
C 10.79996620 -11.64384542 25.39901281
C 11.24742017 -10.75197014 24.25264513
O 11.09889049 -11.05740818 23.06777086
H 10.50804467 -11.02005128 26.24405422
H 11.57898597 -12.34604204 25.69494313
H 9.93851072 -12.22634945 25.04385286
N 11.80041712 -9.57817515 24.66018168
C 12.12962596 -8.49954327 23.75049593
C 13.57378628 -8.07800543 24.04661405
O 13.95137118 -7.87523286 25.20873020
C 11.16854175 -7.30318786 23.91182763
C 9.68262061 -7.71404288 23.88840084
C 9.15239871 -8.28366456 25.18381728
O 9.47259852 -7.48278940 26.24830473
N 8.45778372 -9.35211767 25.22896864
H 11.90310518 -9.39723611 25.65995525
H 12.03750646 -8.88848368 22.72812686
H 11.38544346 -6.77563725 24.85242664
H 11.36648934 -6.60547745 23.08470274
H 9.07388077 -6.81981815 23.66947355
H 9.47049022 -8.44700708 23.09763731
H 8.14982722 -9.55130967 26.19101685
N 14.39251334 -7.96891126 22.97478987
C 15.77096112 -7.51993648 23.12314879
H 13.96790268 -8.00586725 22.05085862
H 15.82101801 -6.93803270 24.04302093
H 16.48999098 -8.33801673 23.16485923
H 16.03814065 -6.87460176 22.27264077
O 7.40349939 -7.55499204 21.17056279
C 6.53101914 -7.81649371 20.34213814
N 6.51152383 -7.14929407 19.10564511
C 5.58576019 -7.33407599 18.11182241
O 5.56487865 -6.63290841 17.09178912
N 4.65077068 -8.36153992 18.25042562
C 4.54554638 -8.96912902 19.43750471
C 5.44548208 -8.77469236 20.52640067
N 5.34784035 -9.44829817 21.70057545
C 4.28533319 -10.27361264 21.82426651
C 4.07705786 -10.93035838 23.05707370
C 3.00038964 -11.78797351 23.28826950
C 2.83338209 -12.45557173 24.62247335
C 2.05438987 -12.02618741 22.24190616
C 0.87635727 -12.93722368 22.46299302
C 2.22960457 -11.37885049 21.01549137
C 3.31742236 -10.51277991 20.77347215
N 3.51024068 -9.87845747 19.55571327
C 2.60545428 -10.10067048 18.39796013
C 1.56132425 -8.98790380 18.23452379
H 7.22365929 -6.41197775 18.95546567
H 4.81012125 -10.72937295 23.84005722
H 1.84569488 -12.22861068 25.05881434
H 2.88160469 -13.55389059 24.52298168
H 3.60975708 -12.14021193 25.33205983
H 0.29269926 -13.06759353 21.54063089
H 1.19561277 -13.93423928 22.80806094
H 0.19850705 -12.52929258 23.23158959
H 1.50507057 -11.56974981 20.22455903
H 2.17795838 -11.10377564 18.49418668
H 3.25281395 -10.09814138 17.51352630
H 2.14169798 -8.07291618 18.34688315
H 1.13638423 -9.04288029 17.22069485
H 0.74296170 -8.98716089 18.95495824
O -0.37794553 -9.92524441 22.21396015
H 0.51158292 -10.25358497 21.99250095
H -0.87930016 -10.04950576 21.39287545
H 9.15507963 -7.89736342 27.06827382

EL222 **Qc** S_1

C 15.46501174 -9.61300268 29.59103613
C 14.05174177 -9.08603925 29.75053042
O 13.29003297 -9.45556594 30.64312157
H 16.07696914 -8.80499946 29.18995486
H 15.81199596 -9.88599475 30.58800527
H 15.49147283 -10.48637147 28.92248253
N 13.69264208 -8.10370355 28.86499028
C 12.31522958 -7.62952808 28.87003096
C 11.40757873 -8.80444869 28.48300660
O 11.62300452 -9.43372426 27.44668228
C 12.16766205 -6.46749459 27.88927316
H 14.18038555 -8.05377742 27.97273611
H 12.08053873 -7.30128298 29.89427496
H 11.14187900 -6.07158645 27.91619871
H 12.85703581 -5.65592941 28.16747667
H 12.41203441 -6.80341006 26.86989019
N 10.38089872 -9.09390732 29.31461690
C 9.57603992 -10.29299614 29.12698938
H 10.42507232 -8.69680636 30.24679755
H 9.58401795 -10.54799639 28.06700115
H 9.94595662 -11.13801653 29.70800780
H 8.53856247 -10.09082307 29.43352959
C -2.49258473 -7.05791526 23.10673821
C -1.22674962 -6.26967543 22.90253008
O -1.26273140 -5.05646037 22.64754698
H -2.31301780 -8.03526400 23.57749249
H -3.09806887 -6.43628165 23.76720380
H -3.02521599 -7.22187205 22.17010225
N -0.05660982 -6.94725435 23.01129982
C 1.22526606 -6.28695762 22.83820150
C 1.57210699 -6.00811592 21.35776808
O 2.74274456 -6.14799276 20.94993932
C 2.32522068 -7.14006215 23.47826868
S 3.98081936 -6.44009585 23.15218522
H -0.05299349 -7.97668498 23.02182748
H 1.16513338 -5.29446272 23.31747992
H 2.18470171 -7.16514277 24.56864656
H 2.29620995 -8.16400383 23.08772836
H 4.58290042 -7.67143961 22.74584160
N 0.54721523 -5.59593098 20.60889252
C 0.69862718 -5.07635305 19.26820085
H -0.27567253 -5.29124101 21.13995756
H 1.59887872 -5.48329878 18.81202884
H -0.16359239 -5.28351486 18.63431186
H 0.82004755 -3.97876465 19.30118611
C 8.88993669 -1.76459027 16.94536705
C 8.77658909 -2.64480474 18.19599768
C 8.12692554 -4.02289692 18.08500238
O 8.23932501 -4.81908914 19.04047212
N 7.40737302 -4.33957904 17.00103486
H 7.93208660 -1.72640253 16.39669941
H 9.76455333 -2.81260960 18.65333822
H 8.18202268 -2.12741242 18.97012626
H 7.28393909 -3.68174553 16.24279884
H 6.88907835 -5.23591069 16.98941968
H 9.10034545 -0.73097377 17.21863863
H 9.65281703 -2.12331225 16.25396051
C 13.21284252 -5.73699730 18.45918208
C 11.77241200 -5.53582623 18.94270874
C 11.41360109 -6.45578792 20.11965535
O 12.24358555 -7.20486006 20.65206577
N 10.12678749 -6.36763371 20.52334631
H 13.87516992 -5.50895837 19.29485609
H 11.60002002 -4.49363090 19.26689865
H 11.04159790 -5.72114421 18.13675523
H 9.46043210 -5.75479258 20.04864638
H 9.76811649 -7.02065267 21.21067067
H 13.32594215 -6.78002413 18.16406278
H 13.44736938 -5.08055512 17.60838217
C 10.79998763 -11.64381462 25.39904711
C 11.23942107 -10.75320497 24.24929338
O 11.07512768 -11.05422285 23.06519218
H 10.50803361 -11.02005673 26.24405427
H 11.57897766 -12.34606040 25.69492016
H 9.93666813 -12.22676342 25.04956342
N 11.80889189 -9.58151452 24.64782184
C 12.12537851 -8.50588493 23.72980050
C 13.55575547 -8.04657400 24.03682966
O 13.90047433 -7.78302965 25.19712360
C 11.14006492 -7.32532599 23.86409864
C 9.66305740 -7.77614844 23.85859197
C 9.13944059 -8.30010737 25.19620246
O 8.64133001 -9.41184453 25.34052582
N 9.20066116 -7.40827897 26.23436355
H 11.89792718 -9.38768372 25.64645927
H 12.04992022 -8.90647581 22.71067343
H 11.37156732 -6.76651677 24.78561071
H 11.32519150 -6.64145097 23.02289917
H 9.02019864 -6.92810300 23.56718353
H 9.49894678 -8.57624656 23.12299030
H 9.74591238 -6.55997154 26.15638939
H 9.03769494 -7.76739892 27.16928158
N 14.39438975 -7.97527466 22.97772797
C 15.77097520 -7.51994542 23.12315688
H 13.98832977 -8.06552976 22.04965881
H 15.82101806 -6.93802254 24.04301453
H 16.48999452 -8.33801397 23.16485446
H 16.03608046 -6.87391584 22.27240825
O 6.83819468 -7.13725306 21.58659464
C 6.17244604 -7.54532958 20.62316221
N 6.36051008 -7.03181825 19.35036636
C 5.62953045 -7.37737449 18.21499435
O 5.86376790 -6.78380203 17.14510707
N 4.67199848 -8.35233039 18.30904237
C 4.45622235 -8.91620500 19.49392627
C 5.13920431 -8.57611363 20.69879709
N 4.84160004 -9.10080816 21.91473121
C 3.91764018 -10.10663753 21.95572668
C 3.66193477 -10.76141198 23.18289607
C 2.72895031 -11.78891942 23.31134454
C 2.51858539 -12.47821311 24.63817309
C 1.98563931 -12.17812643 22.16965298
C 0.93281068 -13.25348974 22.26811943
C 2.21402114 -11.52588208 20.94624831
C 3.18115608 -10.51638011 20.80156464
N 3.44648447 -9.87794157 19.57924076
C 2.60257792 -10.10305812 18.39168182
C 1.56123283 -8.98795751 18.23442383
H 7.06422464 -6.28611866 19.24466791
H 4.25421464 -10.44219237 24.04326398
H 1.46785863 -12.41970563 24.97164154
H 2.76153222 -13.55223273 24.57191925
H 3.15044469 -12.03839178 25.42270138
H 0.48701291 -13.47345343 21.28749392
H 1.34819551 -14.19239517 22.66979666
H 0.11714947 -12.95139510 22.94825190
H 1.62406265 -11.84296778 20.08782862
H 2.16546357 -11.10534978 18.44222480
H 3.27292730 -10.07699085 17.52444725
H 2.14172082 -8.07293740 18.34693946
H 1.12720065 -9.01218378 17.22266789
H 0.74298682 -8.98710834 18.95498576
O 0.23313364 -9.74756844 22.95677710
H 0.77653829 -10.13177266 23.66293239
H 0.63646704 -10.13545801 22.15932228

EL222 **Qd** S_1

C 15.46501195 -9.61300290 29.59103348
C 14.05263899 -9.07646807 29.73995756
O 13.29119293 -9.42085631 30.64337118
H 16.07696956 -8.80499898 29.18995660
H 15.81199506 -9.88599538 30.58800506
H 15.49128280 -10.48486586 28.92042419
N 13.69770705 -8.11947118 28.82698811
C 12.31718982 -7.64976255 28.79476105
C 11.41958243 -8.84412719 28.45825539
O 11.64522710 -9.53155569 27.45990568
C 12.17223478 -6.53839513 27.75564247
H 14.19364223 -8.09386530 27.93804124
H 12.06683706 -7.27647722 29.80004950
H 11.11873188 -6.25304577 27.63377914
H 12.76326158 -5.66196018 28.06326786
H 12.54183207 -6.89340480 26.78204592
N 10.38870663 -9.09980579 29.29436428
C 9.57603992 -10.29299656 29.12698843
H 10.40783321 -8.65506978 30.20496207
H 9.58401663 -10.54799628 28.06700115
H 9.94595778 -11.13801579 29.70800875
H 8.54179061 -10.08384613 29.44055590
C -2.49257348 -7.05783160 23.10673810
C -1.17069055 -6.35779736 22.91974231
O -1.09915134 -5.12410138 22.81172729
H -2.36151931 -8.04442273 23.57812904
H -3.09809903 -6.43632234 23.76721433
H -3.02520741 -7.22189348 22.17010136
N -0.08420074 -7.16580620 22.89203390
C 1.26340359 -6.64386555 22.67401796
C 1.50872959 -6.42538340 21.16319612
O 2.41584356 -6.99391292 20.54577755
C 2.29723753 -7.57680917 23.30042555
S 3.96151072 -6.82076808 23.49747041
H -0.22705169 -8.17250209 22.75000908
H 1.30602437 -5.65899488 23.16380669
H 1.97121372 -7.84470526 24.31567967
H 2.39735179 -8.49955771 22.71172819
H 4.27721557 -6.85135299 22.17907477
N 0.61003221 -5.57479569 20.61681473
C 0.69863603 -5.07624427 19.26821445
H -0.03431494 -5.11698841 21.26690872
H 1.59885353 -5.48335191 18.81202651
H -0.16357618 -5.28356683 18.63430646
H 0.81179278 -3.97732341 19.28672973
C 8.88981710 -1.76457324 16.94536255
C 8.74277107 -2.66654376 18.17657872
C 8.05970415 -4.01915831 17.99142463
O 8.31216859 -4.95791422 18.77425405
N 7.15097823 -4.16209358 17.01585759
H 7.94463832 -1.70637571 16.37704217
H 9.71831928 -2.87414732 18.64082069
H 8.14362680 -2.15297244 18.95058300
H 6.89814258 -3.39008314 16.41382184
H 6.60993116 -5.03461195 16.96532994
H 9.10042123 -0.73098676 17.21862873
H 9.65283640 -2.12331444 16.25398263
C 13.21283168 -5.73699910 18.45916885
C 11.77675178 -5.52677476 18.94219353
C 11.41192725 -6.45469035 20.10982678
O 12.23755229 -7.21325355 20.63689383
N 10.12587704 -6.35901373 20.50961326
H 13.87516262 -5.50896361 19.29486360
H 11.61542395 -4.48603760 19.27641956
H 11.04121932 -5.69587071 18.13732938
H 9.47008276 -5.77046253 19.99524440
H 9.73193675 -7.04354048 21.14550821
H 13.32595622 -6.78002291 18.16406374
H 13.45253727 -5.08171189 17.60859654
C 10.79999419 -11.64382865 25.39904430
C 11.25310343 -10.75629039 24.24962914
O 11.15778511 -11.09345250 23.06743875
H 10.50802874 -11.02005207 26.24404924
H 11.57897618 -12.34605829 25.69492767
H 9.94369581 -12.23373417 25.04095069
N 11.74021003 -9.55336208 24.65254046
C 12.13505500 -8.50652126 23.73363052
C 13.58933113 -8.13153436 24.03955445
O 13.98638404 -7.99592374 25.20434332
C 11.21967027 -7.26966702 23.85195932
C 9.72851684 -7.64084766 23.77483406
C 9.16726235 -8.16299310 25.08101766
O 8.52873850 -9.35581672 24.91268231
N 9.28340067 -7.51057661 26.17172632
H 11.84523721 -9.36957734 25.65277289
H 12.04573030 -8.90887785 22.71590789
H 11.40791296 -6.74720001 24.80180724
H 11.47497956 -6.58682617 23.02882088
H 9.14292802 -6.74582547 23.51155330
H 9.53943466 -8.38749464 22.99001753
H 8.85830210 -8.01063101 26.96325193
N 14.39390730 -7.96691663 22.96430189
C 15.77097583 -7.51994389 23.12314514
H 13.96110328 -7.96240073 22.04310675
H 15.82101986 -6.93802704 24.04301718
H 16.48999595 -8.33801223 23.16486589
H 16.04208350 -6.87425853 22.27425926
O 7.39191242 -7.60564171 21.21627524
C 6.52038030 -7.85057828 20.38168371
N 6.50457128 -7.16513705 19.15412627
C 5.58631440 -7.34258966 18.15168353
O 5.56640553 -6.63136062 17.13926214
N 4.65812736 -8.37841406 18.27189011
C 4.53689586 -8.98996606 19.45546509
C 5.42937175 -8.80527256 20.55217742
N 5.31614486 -9.48459952 21.72203084
C 4.24813742 -10.30492464 21.83233625
C 4.01830580 -10.96158709 23.06177534
C 2.93600118 -11.81540066 23.27765283
C 2.74714581 -12.48294289 24.60916185
C 2.00342929 -12.04831740 22.21858008
C 0.81725873 -12.95241366 22.42310957
C 2.19693547 -11.39840206 20.99626022
C 3.29141101 -10.53723112 20.77020686
N 3.49903358 -9.89618993 19.55825634
C 2.60147871 -10.10406890 18.39292496
C 1.56125035 -8.98794788 18.23435446
H 7.20910904 -6.41855940 19.01555994
H 4.73821058 -10.76246843 23.85703212
H 1.75543190 -12.24933299 25.03252958
H 2.78930334 -13.58145311 24.50987359
H 3.51572636 -12.17299372 25.32966625
H 0.24674317 -13.08031426 21.49224615
H 1.12515973 -13.95083931 22.77413083
H 0.13081439 -12.53854344 23.18082022
H 1.48010036 -11.58258215 20.19676341
H 2.17089565 -11.10700267 18.47635922

H	3.25479443	-10.09356595	17.51280074
H	2.14170695	-8.07292972	18.34695253
H	1.13486641	-9.04004417	17.22089546
H	0.74301837	-8.98713766	18.95502190
O	-0.38464319	-9.91916660	22.20293823
H	0.50190215	-10.25382792	21.97872128
H	-0.89130205	-10.04588661	21.38554782
H	8.15017076	-9.63423804	25.76320609

EL222 **Qa** T_1

C	15.46499703	-9.61300554	29.59100263
C	14.06131892	-9.05207898	29.74122002
O	13.31029813	-9.35507042	30.66729058
H	16.07699909	-8.80499861	29.19000312
H	15.81200337	-9.88599792	30.58800204
H	15.48105735	-10.48194491	28.91670249
N	13.70626856	-8.11665807	28.80258071
C	12.31865315	-7.66536196	28.76236593
C	11.43838050	-8.87896976	28.45775680
O	11.69013139	-9.59595947	27.48543753
C	12.13936970	-6.59021423	27.69097510
H	14.18168643	-8.14269280	27.90172539
H	12.06745292	-7.26750867	29.75760837
H	11.07320359	-6.35528535	27.56102975
H	12.68705587	-5.67765007	27.97195452
H	12.52884124	-6.95749441	26.73027450
N	10.40403837	-9.11265173	29.28954078
C	9.57603166	-10.29300990	29.12699330
H	10.39927975	-8.63137423	30.18099626
H	9.58399012	-10.54799538	28.06700094
H	9.94598667	-11.13799997	29.70801198
H	8.54461763	-10.07168918	29.43938525
C	-2.49301392	-7.05790838	23.10698433
C	-1.18803165	-6.33506099	22.90023234
O	-1.15906739	-5.11004024	22.69970681
H	-2.34916565	-8.04213901	23.57952903
H	-3.09800375	-6.43603230	23.76702870
H	-3.02497820	-7.22204462	22.16999610
N	-0.07302404	-7.09947663	22.94040033
C	1.26310267	-6.54520305	22.71699477
C	1.51324419	-6.37883004	21.19749850
O	2.43059242	-6.95878772	20.60511732
C	2.31908673	-7.42133996	23.39046061
S	3.97581331	-6.63403386	23.52207948
H	-0.15099258	-8.11558464	23.05195778
H	1.27669515	-5.53936823	23.16589588
H	2.01092778	-7.62099235	24.42730194
H	2.42216722	-8.38147562	22.86666811
H	4.24348824	-6.68463802	22.19405313
N	0.61093119	-5.55372168	20.62339008
C	0.69799569	-5.07509349	19.26898832
H	-0.05665991	-5.10304136	21.25703247
H	1.59902402	-5.48295746	18.81201122
H	-0.16301568	-5.28496075	18.63401002
H	0.81102970	-3.97556821	19.27514307
C	8.88998104	-1.76497835	16.94499641
C	8.76657298	-2.65091469	18.18948229
C	8.07871913	-4.00453853	18.05433068
O	8.15962224	-4.82596339	18.99266762
N	7.36006298	-4.27512091	16.95748225
H	7.93424803	-1.71717906	16.39356012
H	9.75233008	-2.85057489	18.63832604
H	8.18883250	-2.12978893	18.97354427
H	7.25472851	-3.59775102	16.21407616
H	6.80806084	-5.14087492	16.93397095
H	9.10001985	-0.73100209	17.21899349
H	9.65300319	-2.12300907	16.25400814
C	13.21299276	-5.73700815	18.45902047
C	11.77409473	-5.51659117	18.92296503
C	11.38255214	-6.45728554	20.06978886
O	12.20384169	-7.18891463	20.63918633
N	10.07302239	-6.41640088	20.38805839
H	13.87501175	-5.50899351	19.29499082
H	11.62192103	-4.47921492	19.27251560
H	11.05320905	-5.66511921	18.10065666
H	9.43461767	-5.76527886	19.92993578
H	9.69419181	-7.03626731	21.09299546
H	13.32599787	-6.78000074	18.16399976
H	13.46426448	-5.08520204	17.60873407
C	10.79999419	-11.64399227	25.39900075
C	11.25057909	-10.75508747	24.24869588
O	11.18453359	-11.10378464	23.06584455
H	10.50800159	-11.02000477	26.24400553
H	11.57900226	-12.34599976	25.69500091
H	9.94477285	-12.23853042	25.04345084
N	11.69309611	-9.53768557	24.65295296
C	12.13468473	-8.50395560	23.74317127
C	13.59883499	-8.16420327	24.05184830
O	14.01174861	-8.06597580	25.21424814
C	11.25929882	-7.23920001	23.85140225
C	9.78826180	-7.50910576	23.52805379
C	8.91569709	-7.95363740	24.70209601
O	9.25839249	-7.84575795	25.88059089
N	7.70645950	-8.45140724	24.32549686
H	11.76801686	-9.35101311	25.65582608
H	12.04803238	-8.90095882	22.72266729
H	11.33533334	-6.82074559	24.86556770
H	11.65839938	-6.50248876	23.13860664
H	9.31528312	-6.58950646	23.14463899
H	9.69377598	-8.25900405	22.72559316
H	7.36967549	-8.36854994	23.36475244
H	7.01928829	-8.60913286	25.05396337
N	14.39272628	-7.97069008	22.97117882
C	15.77099822	-7.52000400	23.12301623
H	13.95061748	-7.94543854	22.05489904
H	15.82100256	-6.93799613	24.04299844
H	16.49000140	-8.33800027	23.16499295
H	16.03639003	-6.87536732	22.27193919
O	7.26551848	-7.53285132	21.42552923
C	6.43252015	-7.77591073	20.54619486
N	6.45444777	-7.08998647	19.33712497
C	5.60153137	-7.31743051	18.27985428
O	5.68406804	-6.66821570	17.23157759
N	4.62319425	-8.30378192	18.39977034
C	4.50353514	-8.94085434	19.55328329
C	5.36047896	-8.76711312	20.67330678
N	5.25297311	-9.46133048	21.83865039
C	4.19394316	-10.29789558	21.91515328
C	3.99352988	-11.02674289	23.11178703
C	2.93716806	-11.91001888	23.29266543
C	2.78877519	-12.67039954	24.57925688
C	1.98134033	-12.09651195	22.23420885
C	0.82066894	-13.03984220	22.41152937
C	2.14272954	-11.38370824	21.04882080
C	3.23148815	-10.50761842	20.84058474
N	3.44752729	-9.87083139	19.63628384
C	2.59770954	-10.10144424	18.44100928
C	1.56094506	-8.98809177	18.23480008
H	7.14161528	-6.32146664	19.21964550
H	4.72693210	-10.86580082	23.90337460
H	1.80424979	-12.48781169	25.04192305
H	2.84550918	-13.75746598	24.39903186
H	3.56989904	-12.39955901	25.30169045
H	0.21039600	-13.09850589	21.49964421
H	1.16557743	-14.05708894	22.65865044
H	0.16512204	-12.71261164	23.23559911
H	1.40489696	-11.52236633	20.26029913
H	2.16278425	-11.10290059	18.52500173
H	3.28084297	-10.11161772	17.58375929
H	2.14198201	-8.07300534	18.34611399
H	1.14619517	-9.05798412	17.21822465
H	0.74306428	-8.98692514	18.95507434
O	0.06762663	-9.92721311	23.40973928
H	0.64415503	-9.99065267	24.18787516
H	0.61410449	-10.30788184	22.70076443

EL222 **Qb** T_1

C	15.46501211	-9.61300872	29.59101311
C	14.05056043	-9.08700727	29.74135464
O	13.28041126	-9.46175943	30.62489771
H	16.07697316	-8.80499766	29.18996412
H	15.81198612	-9.88598671	30.58801019
H	15.49392196	-10.48648488	28.92256562
N	13.69852945	-8.10404190	28.85473700
C	12.31754108	-7.63988207	28.83689515
C	11.42304410	-8.83222166	28.47953980
O	11.65881262	-9.51201725	27.48014835
C	12.16823187	-6.50560852	27.82357650
H	14.19788946	-8.05018891	27.96907579
H	12.06909633	-7.28832438	29.85051798
H	11.12374721	-6.17199741	27.75990562
H	12.80031182	-5.65622898	28.12482573
H	12.48690114	-6.85101564	26.82896976
N	10.37010710	-9.08467334	29.29453716
C	9.57606167	-10.29298873	29.12696012
H	10.38645958	-8.65162383	30.21151016
H	9.58399869	-10.54799877	28.06700099
H	9.94596270	-11.13801283	29.70800912
H	8.53860819	-10.09432490	29.43670645
C	-2.49259318	-7.05784351	23.10670868
C	-1.17415023	-6.35241266	22.91937025
O	-1.10916671	-5.11863138	22.80618394
H	-2.35874399	-8.04398838	23.57806807
H	-3.09807822	-6.43632446	23.76723571
H	-3.02520822	-7.22188750	22.17010061
N	-0.08384761	-7.15448641	22.89812410
C	1.26127493	-6.62541621	22.68397152
C	1.51298212	-6.41160601	21.17329689
O	2.42931596	-6.97331305	20.56463442
C	2.29630387	-7.54939885	23.32164164
S	3.95254366	-6.78118282	23.53311838
H	-0.22046465	-8.16322646	22.76372573
H	1.29524744	-5.63809609	23.16957308
H	1.96297562	-7.81691171	24.33465755
H	2.40829580	-8.47358813	22.73732481
H	4.28482198	-6.81937073	22.21966769
N	0.60794304	-5.57263570	20.61772015
C	0.69863105	-5.07631436	19.26819439
H	-0.04222201	-5.11643183	21.26294624
H	1.59887012	-5.48332740	18.81203636
H	-0.16358901	-5.28352738	18.63431133
H	0.81225369	-3.97719166	19.28483559
C	8.88997262	-1.76463013	16.94540420
C	8.74689251	-2.67246508	18.17095145
C	8.11077203	-4.04349955	17.96204572
O	8.35520409	-4.97146300	18.76046227
N	7.25380996	-4.21689512	16.94560910
H	7.94303332	-1.70644675	16.37953433
H	9.71938260	-2.85378828	18.65264425
H	8.11800234	-2.18193723	18.93610069
H	7.01164088	-3.45598536	16.32539129
H	6.73884747	-5.10337943	16.87235472
H	9.10032164	-0.73096505	17.21862624
H	9.65281269	-2.12330115	16.25395019
C	13.21281596	-5.73702842	18.45919933
C	11.77832026	-5.52964084	18.95090712
C	11.43021302	-6.43836232	20.13912611
O	12.26087303	-7.19199190	20.66410196
N	10.15162083	-6.33054590	20.56341852
H	13.87517299	-5.50892741	19.29484440
H	11.61147840	-4.48438046	19.26777566
H	11.03623304	-5.71845821	18.15658599
H	9.48435668	-5.76111307	20.04268663
H	9.77035650	-7.01431699	21.20787487
H	13.32594897	-6.78002550	18.16406929
H	13.44755692	-5.08100209	17.60801820
C	10.79996678	-11.64384685	25.39901181
C	11.24659153	-10.75024910	24.25363401
O	11.09487800	-11.05222886	23.06827072
H	10.50804477	-11.02005080	26.24405390
H	11.57898586	-12.34604188	25.69494387
H	9.93847151	-12.22615439	25.04366860
N	11.80200063	-9.57816049	24.66300129
C	12.13014323	-8.49750673	23.75538574
C	13.57500619	-8.07716224	24.04950230
O	13.95385985	-7.87395797	25.21112743
C	11.17056469	-7.30083710	23.92245202
C	9.68395937	-7.70934754	23.89797678
C	9.15258794	-8.28247074	25.19147945
O	9.47207336	-7.48425988	26.25797676
N	8.45765000	-9.35082160	25.23294149
H	11.90594342	-9.39931578	25.66305845
H	12.03565106	-8.88378754	22.73222677
H	11.38849581	-6.77787482	24.86529517
H	11.36921391	-6.59899460	23.09891972
H	9.07679669	-6.81309178	23.68275140
H	9.47089864	-8.43988562	23.10510215
H	8.14853836	-9.55274617	26.19401024
N	14.39242587	-7.96921373	22.97661601
C	15.77096515	-7.51993653	23.12315265
H	13.96687798	-8.00708139	22.05318705
H	15.82101912	-6.93803249	24.04302072
H	16.48999119	-8.33801662	23.16485864
H	16.03721004	-6.87480448	22.27223082
O	7.39193575	-7.53338843	21.17961940
C	6.54126787	-7.79383328	20.33028557
N	6.54421846	-7.14724366	19.09481508
C	5.63659285	-7.35430458	18.08354243
O	5.67553554	-6.69823878	17.03521949
N	4.65057891	-8.32881719	18.24901838
C	4.56430446	-8.94993740	19.41599175
C	5.45427780	-8.76454079	20.50723149
N	5.36424083	-9.42274775	21.69499359
C	4.29787459	-10.23778559	21.82272179
C	4.09949520	-10.89573781	23.06189303
C	3.03176022	-11.74768868	23.30189475
C	2.86996544	-12.41189190	24.63910328
C	2.07343129	-11.99242606	22.25762955
C	0.90060088	-12.90525444	22.49535523
C	2.23481161	-11.35217149	21.02988647
C	3.31834457	-10.48441960	20.77127898
N	3.50658339	-9.87568923	19.54779599
C	2.60298090	-10.10358587	18.39573298
C	1.56130711	-8.98791164	18.23450829
H	7.25988978	-6.41154774	18.94598668
H	4.84120458	-10.69170436	23.83567611
H	1.88599616	-12.17985101	25.08021025
H	2.91540861	-13.51003656	24.54118660
H	3.65177961	-12.09615026	25.34238693
H	0.30813705	-13.04227610	21.57981562
H	1.22720595	-13.89863280	22.84333600
H	0.23072385	-12.49382754	23.26871063
H	1.50360112	-11.54683919	20.24624931
H	2.17120998	-11.10524924	18.49504982
H	3.24424960	-10.11192354	17.50698853
H	2.14170515	-8.07292258	18.34689834
H	1.13338673	-9.03808732	17.22142538
H	0.74296005	-8.98716264	18.95495639
O	-0.37909305	-9.91471547	22.22549690
H	0.50726913	-10.23521453	21.98009042
H	-0.89473357	-10.02268351	21.41085530
H	9.15410335	-7.90038741	27.07702017

EL222 **Qc** T_1

C	15.46501111	-9.61300369	29.59103311
C	14.05333845	-9.07950899	29.75135572
O	13.29465052	-9.43991944	30.65032372
H	16.07697014	-8.80499861	29.18995809
H	15.81199612	-9.88599470	30.58800522
H	15.48843985	-10.48552881	28.92136512
N	13.69484590	-8.10357258	28.85877155
C	12.31435559	-7.63546873	28.85082283
C	11.41419562	-8.81990146	28.47829972
O	11.64283401	-9.47145576	27.45821162
C	12.16259761	-6.49035150	27.85128125
H	14.18262011	-8.06463037	27.96593539
H	12.07302368	-7.29367484	29.86926213
H	11.12068703	-6.14193776	27.82603151
H	12.81174035	-5.65072046	28.14341456
H	12.45218685	-6.83102173	26.84577653
N	10.38511145	-9.09751525	29.30949161
C	9.57603971	-10.29299672	29.12699012
H	10.42091521	-8.68492131	30.23492001
H	9.58401668	-10.54799575	28.06700099
H	9.94595773	-11.13801579	29.70800811
H	8.53942085	-10.08767616	29.43437574
C	-2.49259318	-7.05785351	23.10671868
C	-1.17521248	-6.34947298	22.92016565
O	-1.11481663	-5.11594808	22.80157539
H	-2.35754025	-8.04376920	23.57812284
H	-3.09807922	-6.43632245	23.76723269
H	-3.02520522	-7.22188052	22.17009760
N	-0.08085108	-7.14695981	22.90685584
C	1.26125443	-6.61086532	22.68934579
C	1.51097851	-6.40592402	21.17710427
O	2.42569772	-6.97174267	20.56952481
C	2.30542047	-7.52056874	23.33245702
S	3.95687626	-6.73494511	23.52427340
H	-0.21340973	-8.15631806	22.77262606
H	1.28790042	-5.61989366	23.16778007
H	1.98045088	-7.77913226	24.35040544
H	2.42244368	-8.45002058	22.75755457
H	4.27298671	-6.77367152	22.20679144
N	0.60668568	-5.56868401	20.61865246
C	0.69863105	-5.07630536	19.26818540
H	-0.04431757	-5.11094939	21.26198134
H	1.59887112	-5.48332740	18.81203937
H	-0.16359001	-5.28353238	18.63431435
H	0.81236664	-3.97708063	19.28259241
C	8.88991065	-1.76458513	16.94537721
C	8.75209813	-2.66671537	18.17579210
C	8.10679770	-4.03591208	17.98575926
O	8.31641391	-4.94041063	18.82042360
N	7.28003440	-4.23183135	16.94915247
H	7.94081374	-1.70873480	16.38298811
H	9.72783616	-2.84949390	18.65088924
H	8.13315925	-2.16939004	18.94456446
H	7.06638771	-3.48844852	16.29795716
H	6.75820657	-5.11537300	16.88833238
H	9.10036566	-0.73097805	17.21863926
H	9.65281168	-2.12331716	16.25395718
C	13.21285094	-5.73699942	18.45915932
C	11.77602337	-5.53427722	18.94717447
C	11.42064253	-6.46000001	20.12023819
O	12.24712119	-7.22331369	20.63683806
N	10.13969799	-6.35657909	20.54008742
H	13.87515500	-5.50895641	19.29486741
H	11.60725202	-4.49314563	19.27643993
H	11.03935641	-5.71351580	18.14538717
H	9.47745092	-5.76785601	20.03432658
H	9.75413235	-7.04689510	21.17529665
H	13.32594797	-6.78002450	18.16406633
H	13.44754401	-5.08016689	17.60863601
C	10.79998779	-11.64383484	25.39904181
C	11.24262365	-10.75482373	24.25076465
O	11.08316498	-11.05702971	23.06557949
H	10.50803075	-11.02005080	26.24404887
H	11.57897983	-12.34605389	25.69492984
H	9.93720307	-12.22705278	25.04856269
N	11.81236043	-9.58568544	24.65218560
C	12.12986197	-8.50950704	23.73602537
C	13.56250110	-8.05689840	24.03973442
O	13.91900734	-7.80962239	25.19974080
C	11.14519202	-7.32936256	23.87389964
C	9.66812448	-7.78224340	23.84796366
C	9.12710467	-8.28835556	25.18461898

O 8.66934577 -9.41437251 25.35230435
N 9.18692239 -7.38450018 26.20995292
H 11.89406561 -9.39227010 25.65216280
H 12.05290997 -8.90886446 22.71640267
H 11.35942932 -6.78582640 24.80772794
H 11.33748566 -6.63703081 23.04089273
H 9.03253075 -6.93594853 23.53640627
H 9.51025562 -8.58683714 23.11644035
H 9.38197247 -6.40588375 26.04401770
H 8.77701799 -7.64441363 27.09985824
N 14.39450008 -7.97332634 22.97583801
C 15.77098012 -7.51994955 23.12314466
H 13.98383816 -8.05152355 22.04864195
H 15.82101515 -6.93801952 24.04301273
H 16.48999420 -8.33801360 23.16486769
H 16.03769805 -6.87401961 22.27273597
O 7.34901493 -7.52075438 21.24204998
C 6.50961932 -7.78496432 20.38208954
N 6.52230973 -7.13458035 19.14857139
C 5.63231927 -7.34984330 18.12326883
O 5.68519228 -6.69749841 17.07323897
N 4.64844396 -8.32777260 18.27672473
C 4.55145618 -8.95076530 19.44141051
C 5.42796716 -8.76421349 20.54352347
N 5.32891814 -9.42831437 21.72723710
C 4.26422623 -10.24889720 21.84043996
C 4.05592276 -10.91539447 23.07312037
C 2.98745764 -11.77092972 23.29871615
C 2.81532186 -12.44352067 24.63021363
C 2.03794887 -12.00962819 22.24510106
C 0.86046141 -12.91962737 22.46919482
C 2.21010516 -11.36267958 21.02259028
C 3.29555340 -10.49284225 20.77850315
N 3.49517399 -9.87981163 19.55924426
C 2.60213908 -10.10373812 18.39838824
C 1.56126911 -8.98791365 18.23446532
H 7.23232076 -6.39200851 19.00872932
H 4.78801447 -10.71475850 23.85673832
H 1.82746287 -12.21485344 25.06433853
H 2.86197189 -13.54098026 24.52514887
H 3.59112269 -12.13224104 25.34199185
H 0.27652583 -13.05233534 21.54759624
H 1.18029625 -13.91475434 22.81821310
H 0.18488193 -12.50698818 23.23696317
H 1.48482017 -11.55176625 20.23217071
H 2.17022986 -11.10605507 18.48981911
H 3.25078826 -10.10764424 17.51500583
H 2.14171716 -8.07293157 18.34691130
H 1.13410733 -9.03899052 17.22112306
H 0.74297405 -8.98715962 18.95497136
O -0.37932594 -9.90672881 22.22939191
H 0.50462235 -10.23294521 21.98245637
H -0.89701301 -10.01369940 21.41591106

EL222 **Qd** T_1

C 15.46501111 -9.61300369 29.59103115
C 14.05265428 -9.07700847 29.74048182
O 13.29088818 -9.42340387 30.64284238
H 16.07697014 -8.80499861 29.18995809
H 15.81199511 -9.88599470 30.58800522
H 15.49124168 -10.48494878 28.92053114
N 13.69794508 -8.11822497 28.82934416
C 12.31717299 -7.64955030 28.79746589
C 11.42046796 -8.84445766 28.46019742
O 11.64814885 -9.53302670 27.46306545
C 12.17172296 -6.53717643 27.75956367
H 14.19402298 -8.09117898 27.94051837
H 12.06642219 -7.27733295 29.80306200
H 11.11810195 -6.25226074 27.63807622
H 12.76240389 -5.66085970 28.06818593
H 12.54166723 -6.89097461 26.78565972
N 10.38801065 -9.09941881 29.29453753
C 9.57603971 -10.29296672 29.12698912
H 10.40498841 -8.65349564 30.20459244
H 9.58401568 -10.54799575 28.06700099
H 9.94595873 -11.13801579 29.70800811
H 8.54168080 -10.08421761 29.44044112
C -2.49259418 -7.05784949 23.10671969
C -1.17321328 -6.35323844 22.92009760
O -1.10765294 -5.11961078 22.80684346
H -2.35937329 -8.04411713 23.57807188
H -3.09808022 -6.43632245 23.76723169
H -3.02520422 -7.22188253 22.17009760
N -0.08259639 -7.15566981 22.89955986
C 1.26236497 -6.62629311 22.68334397
C 1.51191741 -6.41225906 21.17234533
O 2.42714889 -6.97490709 20.56243076
C 2.30016862 -7.54991501 23.31754549
S 3.95741149 -6.78034947 23.52154178
H -0.21994165 -8.16404198 22.76324666
H 1.29705873 -5.63921027 23.16932553
H 1.97140718 -7.81730938 24.33205913
H 2.41026966 -8.47375937 22.73215866
H 4.27200606 -6.79794736 22.20307735
N 0.60760188 -5.57248710 20.61772216
C 0.69863105 -5.07630437 19.26818540
H -0.04180611 -5.11579658 21.26338424
H 1.59887112 -5.48332740 18.81203937
H -0.16359001 -5.28353338 18.63431435
H 0.81208144 -3.97721432 19.28500355
C 8.88991166 -1.76458813 16.94537419
C 8.75140295 -2.66691577 18.17609601
C 8.10082736 -4.03412404 17.98795953
O 8.30777208 -4.93939798 18.82246924
N 7.27194836 -4.22791513 16.95263604
H 7.94097017 -1.70840197 16.38271950
H 9.72736551 -2.85263060 18.64955783
H 8.13573592 -2.16762529 18.94618062
H 7.05865400 -3.48419134 16.30175760
H 6.74690642 -5.10953022 16.89586421
H 9.10035667 -0.73097505 17.21863423
H 9.65282068 -2.12331615 16.25396618
C 13.21284993 -5.73699741 18.45915932
C 11.77690164 -5.52868091 18.94403010
C 11.41802744 -6.45103601 20.11785790
O 12.24373811 -7.21304119 20.63924830
N 10.13606846 -6.34817226 20.52965993
H 13.87515299 -5.50896038 19.29487038
H 11.61186629 -4.48652898 19.27180550
H 11.04078582 -5.70667471 18.14145834
H 9.47755035 -5.75380355 20.02547276
H 9.74900065 -7.02723759 21.17507382
H 13.32594696 -6.78002349 18.16406231
H 13.45101139 -5.08143699 17.60845271
C 10.79998779 -11.64383785 25.39903980
C 11.25274745 -10.75467117 24.25073761
O 11.15494523 -11.08875659 23.06788554
H 10.50802975 -11.02004980 26.24404787
H 11.57897983 -12.34605289 25.69493085
H 9.94366628 -12.23343598 25.04057550
N 11.74215412 -9.55329937 24.65571357
C 12.13569128 -8.50447096 23.73855901
C 13.59047198 -8.13029047 24.04242280
O 13.98860790 -7.99356673 25.20670155
C 11.22143497 -7.26741802 23.86230277
C 9.72983830 -7.63680618 23.78550360
C 9.16846538 -8.16270560 25.09022408
O 8.52993074 -9.35496326 24.91839340
N 9.28448775 -7.51278756 26.18230738
H 11.84793199 -9.37130574 25.65622116
H 12.04413467 -8.90434285 22.72005606
H 11.41101590 -6.74856740 24.81378543
H 11.47661070 -6.58126272 23.04178360
H 9.14508125 -6.73990763 23.52664459
H 9.53981646 -8.38112811 22.99855025
H 8.85948746 -8.01429577 26.97292042
N 14.39388042 -7.96727821 22.96608438
C 15.77098012 -7.51994955 23.12314365
H 13.96031645 -7.96396456 22.04528590
H 15.82101415 -6.93801952 24.04301273
H 16.48999420 -8.33801360 23.16486864
H 16.04122158 -6.87445697 22.27386254
O 7.37087894 -7.57841528 21.24011023
C 6.52176409 -7.82157397 20.38397463
N 6.52745418 -7.15300122 19.15953107
C 5.63153175 -7.35473454 18.13643497
O 5.67846633 -6.69125306 17.09350085
N 4.64716177 -8.33365070 18.28295478
C 4.55141453 -8.96687504 19.44248014
C 5.43333190 -8.79449317 20.54226350
N 5.33371566 -9.46882226 21.72128311
C 4.26231421 -10.28028821 21.83175230
C 4.04799978 -10.95062772 23.06192123
C 2.97412248 -11.79946655 23.28458293
C 2.79579932 -12.47508715 24.61403308
C 2.02361627 -12.02772002 22.23009069
C 0.83985011 -12.93051784 22.45003543
C 2.20009848 -11.37622392 21.01046572
C 3.29060111 -10.51210695 20.77018940
N 3.49253577 -9.89260079 19.55464995
C 2.59894066 -10.10674591 18.39256316
C 1.56128011 -8.98791465 18.23447331
H 7.23562119 -6.40807994 19.02326465
H 4.78093188 -10.75684524 23.84635120
H 1.81007633 -12.23894038 25.04891491
H 2.83288857 -13.57260378 24.50627968
H 3.57369903 -12.17245444 25.32746821
H 0.25508084 -13.05481903 21.52781834
H 1.15247074 -13.92954135 22.79437816
H 0.16695671 -12.51650448 23.21939263
H 1.47321331 -11.55676163 20.21946919
H 2.16413106 -11.10834636 18.47824415
H 3.24768581 -10.10756391 17.50923028
H 2.14171516 -8.07292956 18.34690935
H 1.13233139 -9.03622218 17.22162975
H 0.74297205 -8.98716063 18.95496935
O -0.38528812 -9.91457910 22.22335818
H 0.49936624 -10.23899572 21.97685477
H -0.90308052 -10.02428829 21.41030818
H 8.15031512 -9.63548901 25.76776188

EL222 **Qa** $T_{1\text{-}H}$

C 15.46499814 -9.61300623 29.59100475
C 14.06108661 -9.05250460 29.73930170
O 13.31049795 -9.34951380 30.66767174
H 16.07700025 -8.80499924 29.19000524
H 15.81200448 -9.88599861 30.58800427
H 15.48233880 -10.48191300 28.91664243
N 13.70561825 -8.12305583 28.79499125
C 12.32022978 -7.66544038 28.75575757
C 11.43424689 -8.87504707 28.45159776
O 11.67807753 -9.58716650 27.47381208
C 12.14868877 -6.58994567 27.68354863
H 14.17916828 -8.15531267 27.89321119
H 12.07167004 -7.26542344 29.75073267
H 11.08525819 -6.34197099 27.55601702
H 12.70851570 -5.68395448 27.96166922
H 12.53154100 -6.96368282 26.72266414
N 10.40483441 -9.11233846 29.28910331
C 9.57603235 -10.29301064 29.12699541
H 10.40996966 -8.63901121 30.18492599
H 9.58399081 -10.54799612 28.06700295
H 9.94598736 -11.13800076 29.70801409
H 8.54482639 -10.07158176 29.44027686
C -2.49301410 -7.05790891 23.10698597
C -1.19219165 -6.32709285 22.90302957
O -1.16960304 -5.10264068 22.69803154
H -2.34540263 -8.04199481 23.57808633
H -3.09800397 -6.43603278 23.76703039
H -3.02497842 -7.22204514 22.16999769
N -0.07161324 -7.08248914 22.95126651
C 1.26004302 -6.52304169 22.73277918
C 1.54006635 -6.32397674 21.22224589
O 2.53474374 -6.81384517 20.67439742
C 2.32160165 -7.41486638 23.37357488
S 4.00353110 -6.74325875 23.24287498
H -0.14651496 -8.10174242 23.03941204
H 1.28111748 -5.52325536 23.19632257
H 2.11420213 -7.50522631 24.45485932
H 2.29763182 -8.42919217 22.94702637
N 0.59473222 -5.57630046 20.61624925
C 0.69799574 -5.07509385 19.26898969
H -0.10360585 -5.15195337 21.23524773
H 1.59902413 -5.48295788 18.81201260
H -0.16301569 -5.28496113 18.63401134
H 0.81224536 -3.97599272 19.28856581
C 8.88998167 -1.76497848 16.94499763
C 8.76896984 -2.64733951 18.19213807
C 8.08794470 -4.00610558 18.06708476
O 8.17121725 -4.81251685 19.01941827
N 7.37618405 -4.29740942 16.97275504
H 7.93357687 -1.72057132 16.39443665
H 9.75551313 -2.84027871 18.64250940
H 8.18917710 -2.12463930 18.97363222
H 7.27054064 -3.62860638 16.22148909
H 6.82854386 -5.17259060 16.95731307
H 9.10002048 -0.73100214 17.21899471
H 9.65300388 -2.12300922 16.25400930
C 13.21299371 -5.73700857 18.45902179
C 11.77160289 -5.52636317 18.92391596
C 11.37370996 -6.50056139 20.04050710
O 12.20155263 -7.20448741 20.63525285
N 10.05002340 -6.52643377 20.29835279
H 13.87501276 -5.50899393 19.29499219
H 11.62052987 -4.50010822 19.30563575
H 11.05490411 -5.64703823 18.09390589
H 9.41262003 -5.86026300 19.85927093
H 9.67683538 -7.14258595 21.00957263
H 13.32599882 -6.78000122 18.16400108
H 13.46076609 -5.08269533 17.60987995
C 10.79999499 -11.64399311 25.39900260
C 11.24170659 -10.75199829 24.24755990
O 11.17065698 -11.10045945 23.06475413
H 10.50800233 -11.02000556 26.24400744
H 11.57900311 -12.34600066 25.69500276
H 9.94574606 -12.24206538 25.04627977
N 11.67544651 -9.53052554 24.64958903
C 12.14082726 -8.50937390 23.73871973
C 13.60866626 -8.19145352 24.04890930
O 14.02663363 -8.11892882 25.21136550
C 11.30082469 -7.22096017 23.84106885
C 9.82054267 -7.44403743 23.52504176
C 8.91588463 -7.73623795 24.72046814
O 9.30105619 -7.76315248 25.88648285
N 7.61102037 -7.95985333 24.36619498
H 11.76633281 -9.34813423 25.65211178
H 12.04755448 -8.90541740 22.71837000
H 11.39752490 -6.79671533 24.85092552
H 11.71950337 -6.50296142 23.12097847
H 9.40131115 -6.54729637 23.04038827
H 9.68960167 -8.26651647 22.80130472
H 7.29864678 -7.68713310 23.43230308
H 6.92728638 -7.93813494 25.11583171
N 14.39490691 -7.97331126 22.96839355
C 15.77099933 -7.52000453 23.12301787
H 13.94893067 -7.93400355 22.05433642
H 15.82100372 -6.93799661 24.04300019
H 16.49000257 -8.33800085 23.16499464
H 16.03639289 -6.87535610 22.27210704
O 7.16276507 -7.48021384 21.45481183
C 6.38433809 -7.72601408 20.51410153
N 6.43848694 -7.07254712 19.30829333
C 5.60078337 -7.33081097 18.21236857
O 5.76272140 -6.67637587 17.17306382
N 4.62697943 -8.29040416 18.31878347
C 4.48755010 -8.93110324 19.46658273
C 5.33803078 -8.72249072 20.58428435
N 5.17080480 -9.43356090 21.73216252
C 4.11676651 -10.30130745 21.90113363
C 3.91227988 -10.95952489 23.12365193
C 2.85891089 -11.85220431 23.30640636
C 2.67000400 -12.55959459 24.62377180
C 1.97045883 -12.08351012 22.22418438
C 0.81816361 -13.04610518 22.37545721
C 2.16002264 -11.40087390 21.01459034
C 3.23282229 -10.51452730 20.80982779
N 3.46761836 -9.87132506 19.58711333
C 2.59946271 -10.10191214 18.41040384
C 1.56094517 -8.98809240 18.23480140
H 7.11818596 -6.29849030 19.19936521
H 4.61038524 -10.76084703 23.94009924
H 1.67483715 -12.35689176 25.05487525
H 2.73728697 -13.65342714 24.50119376
H 3.42815091 -12.25337601 25.35778192
H 0.24933571 -13.14312871 21.44022305
H 1.17262447 -14.04852596 22.66569538
H 0.12033264 -12.71160388 23.16089325
H 1.44964058 -11.58113892 20.21013466
H 2.16867521 -11.10614805 18.48670792
H 3.26769722 -10.09553799 17.54040025
H 2.14198216 -8.07300592 18.34611531
H 1.13494985 -9.03470456 17.22080820
H 0.74306433 -8.98692578 18.95507572
O 0.00657494 -9.92185746 23.30190063
H 0.53029319 -10.08309481 24.10250083
H 0.55876350 -10.30816844 22.60015751
H 5.80158519 -9.22600700 22.50347487

EL222 **Qb** $T_{1\text{-}H}$

C 15.46502926 -9.61301485 29.59098416
C 14.05230984 -9.07560975 29.73292987
O 13.28587512 -9.41759377 30.63276340
H 16.07698173 -8.80500300 29.18996803
H 15.81197315 -9.88597898 30.58801961
H 15.49302543 -10.48501937 28.92058956
N 13.69855522 -8.11908324 28.81653553
C 12.31781805 -7.65125467 28.79073111
C 11.41804374 -8.84358568 28.45784507
O 11.63098573 -9.51948013 27.44950544
C 12.15500223 -6.54678619 27.74643949
H 14.19322545 -8.09461821 27.92680040
H 12.07273130 -7.27423366 29.79576274
H 11.10065990 -6.24539953 27.66553533
H 12.75954543 -5.67147114 28.02872989
H 12.49129649 -6.91097179 26.76449015
N 10.38770722 -9.09841326 29.29616878
C 9.57610098 -10.29298338 29.12693286
H 10.42386818 -8.67072036 30.21483323
H 9.58396911 -10.54800914 28.06700512
H 9.94596662 -11.13801235 29.70801108
H 8.54172065 -10.08527973 29.44097405
C -2.49258799 -7.05783679 23.10670360
C -1.17380728 -6.35056478 22.92523306
O -1.10989186 -5.11704223 22.81138502
H -2.35920990 -8.04422085 23.57768405
H -3.09807851 -6.43632832 23.76724058
H -3.02520960 -7.22189094 22.17010337
N -0.07989061 -7.14936821 22.90920264
C 1.26162509 -6.61766271 22.69330663
C 1.53153476 -6.37181315 21.19019426
O 2.50979352 -6.86097571 20.61625444
C 2.30937882 -7.55692417 23.28541792
S 3.99642278 -6.89027429 23.20603218
H -0.21431837 -8.15714606 22.76208676
H 1.30866776 -5.63855758 23.19557617
H 2.09196967 -7.71920886 24.35575728

H	2.28917430	-8.54005528	22.78912152
N	0.59189056	-5.59236374	20.61100410
C	0.69863080	-5.07631378	19.26821043
H	-0.08269780	-5.15667461	21.24527383
H	1.59886607	-5.48332418	18.81202630
H	-0.16358679	-5.28353045	18.63431054
H	0.81300925	-3.97771202	19.29719849
C	8.88998707	-1.76466087	16.94542452
C	8.74115845	-2.66895451	18.17124626
C	8.10823357	-4.04104820	17.96121242
O	8.37278166	-4.97003014	18.75273284
N	7.23746151	-4.21958732	16.95980189
H	7.94446549	-1.70720061	16.37699015
H	9.71136837	-2.84840768	18.65816172
H	8.10767259	-2.17592892	18.93105821
H	6.97648192	-3.45637540	16.34993729
H	6.72677371	-5.11589362	16.89753424
H	9.10031301	-0.73096227	17.21862392
H	9.65281570	-2.12330413	16.25395538
C	13.21281495	-5.73704085	18.45920229
C	11.77830015	-5.52230507	18.93949769
C	11.41044137	-6.44230787	20.11117502
O	12.22624880	-7.21642375	20.63143727
N	10.13082231	-6.31837805	20.52432037
H	13.87518723	-5.50890863	19.29483005
H	11.61693962	-4.47920647	19.26599630
H	11.04225455	-5.69603332	18.13621525
H	9.48199808	-5.73314463	19.99627958
H	9.72176946	-7.01364678	21.13767744
H	13.32594860	-6.78003180	18.16409072
H	13.45550850	-5.08326946	17.60819854
C	10.79995159	-11.64381923	25.39901408
C	11.24933754	-10.76640361	24.24086840
O	11.15185727	-11.11309652	23.06218058
H	-10.50802774	-11.02006049	26.24405618
H	-11.57899523	-12.34603569	25.69494011
H	9.94201853	-12.23525058	25.04687462
N	11.73543653	-9.55953964	24.63323571
C	12.12596347	-8.51675671	23.70761320
C	13.57470467	-8.12220464	24.02583717
O	13.95249357	-7.96631384	25.19472875
C	11.19505469	-7.29237740	23.81487865
C	9.70965183	-7.65360660	23.64632109
C	9.05424570	-8.33606052	24.82344339
O	9.44296226	-7.77839157	26.00819092
N	8.20426850	-9.27834099	24.66973640
H	11.83775697	-9.36647308	25.63138058
H	12.04973088	-8.92622280	22.69176593
H	11.34654354	-6.79462265	24.78410763
H	11.48049735	-6.58539761	23.02274127
H	9.13842653	-6.72665216	23.46630531
H	9.54369623	-8.30096342	22.77432319
H	7.83465100	-9.59121958	25.57857393
N	14.39330266	-7.96794593	22.95979071
C	15.77094398	-7.51991844	23.12318255
H	13.97230178	-7.98089437	22.03323532
H	15.82102325	-6.93804619	24.04303062
H	16.48999368	-8.33801662	23.16484933
H	16.04313662	-6.87339100	22.27529137
O	7.39918659	-7.60238859	21.19244697
C	6.56747845	-7.80779398	20.29726877
N	6.57675487	-7.16057962	19.08171778
C	5.64688493	-7.36035440	18.05357565
O	5.74776167	-6.68219391	17.01937656
N	4.65110432	-8.29138991	18.21138425
C	4.56290722	-8.93727974	19.36331353
C	5.47957702	-8.75558118	20.43113781
N	5.35282356	-9.44107098	21.59862924
C	4.27751824	-10.26764500	21.82979731
C	4.09618872	-10.88181775	23.07810273
C	3.01657847	-11.72460290	23.33076047
C	2.84181093	-12.36675981	24.68262520
C	2.08655774	-11.96529621	22.28872818
C	0.90602252	-12.87705442	22.51333688
C	2.25806261	-11.33299887	21.04842974
C	3.34174363	-10.47699519	20.78068584
N	3.52955085	-9.85599167	19.53765161
C	2.61222497	-10.09669747	18.39979109
C	1.56131025	-8.98791968	18.23451887
H	7.29344105	-6.43005508	18.92882006
H	4.83143345	-10.67889732	23.85988851
H	1.86350709	-12.11222084	25.12344577
H	2.87791488	-13.46690904	24.61085442
H	3.62602201	-12.04760301	25.38326529
H	0.32071840	-13.01256772	21.59277967
H	1.22986654	-13.87137773	22.86106555
H	0.23061212	-12.46735608	23.28223116
H	1.52805120	-11.53175086	20.26516285
H	2.18763685	-11.10164306	18.50381040
H	3.24708346	-10.10052702	17.50502015
H	2.14170792	-8.07292454	18.34689733
H	1.13397555	-9.03516007	17.22110793
H	0.74295803	-8.98715671	18.95495538
O	-0.37239911	-9.89858515	22.20674069
H	0.51374205	-10.23323948	21.97834975
H	-0.87484790	-9.99918333	21.38297602
H	9.02923540	-8.25496474	26.74646965
H	6.03150725	-9.23184219	22.33324183

EL222 **Qc** T_{1-H}

C	15.46502709	-9.61299670	29.59108915
C	14.02930407	-9.10740938	29.70386598
O	13.32505340	-9.31045570	30.69702356
H	16.07695617	-8.80500162	29.18993210
H	15.81201316	-9.88601671	30.58799622
H	15.52605345	-10.48225109	28.91794008
N	13.57845860	-8.35882202	28.65380543
C	12.31015763	-7.63170173	28.72515334
C	11.15725668	-8.56541038	28.32164119
O	10.84838597	-8.71453052	27.12747610
C	12.35106425	-6.39617007	27.83133926
H	14.11414748	-8.28112700	27.79288818
H	12.17258636	-7.34435737	29.77861417
H	11.39382388	-5.85625957	27.88687874
H	13.15450719	-5.72257410	28.16555862
H	12.53601720	-6.68759867	26.78786353
N	10.54213019	-9.20818204	29.32958637
C	9.57608368	-10.29299772	29.12686010
H	11.00867445	-9.14902722	30.23262782
H	9.58400970	-10.54804576	28.06701502
H	9.94594873	-11.13797478	29.70807611
H	8.56413951	-10.00892606	29.45140715
C	-2.49260318	-7.05785049	23.10669069
C	-1.16408369	-6.36649275	22.92954490
O	-1.08305662	-5.13226740	22.83824732
H	-2.36876591	-8.04578023	23.57785529
H	-3.09806823	-6.43632446	23.76724672
H	-3.02521022	-7.22188454	22.17010257
N	-0.07985890	-7.17968816	22.89080764
C	1.26715110	-6.65900701	22.67406601
C	1.53116505	-6.38935225	21.17440139
O	2.50763661	-6.86898073	20.58959459
C	2.31660700	-7.61410040	23.24000128
S	4.00434560	-6.94930259	23.14603158
H	-0.22452562	-8.18142931	22.71645681
H	1.32667775	-5.68895182	23.19218663
H	2.11234698	-7.79066012	24.31057179
H	2.28843513	-8.58909170	22.72813649
N	0.59174792	-5.60041337	20.60784158
C	0.69863605	-5.07632637	19.26819937
H	-0.07783912	-5.16720682	21.24843763
H	1.59887012	-5.48332439	18.81203535
H	-0.16359201	-5.28352638	18.63431636
H	0.81228627	-3.97787519	19.30187785
C	8.88984064	-1.76459613	16.94535324
C	8.67694840	-2.68972681	18.14547093
C	8.03758021	-4.03379416	17.80968654
O	8.51807738	-5.09587429	18.25675844
N	6.93390623	-4.04611019	17.04752250
H	7.96366806	-1.69711576	16.34640275
H	9.62182640	-2.90932063	18.66174769
H	8.01279730	-2.20068895	18.88061746
H	6.50226825	-3.18330471	16.74245784
H	6.44791291	-4.94026426	16.86605825
H	9.10040667	-0.73098705	17.21864424
H	9.65283772	-2.12329915	16.25397718
C	13.21285893	-5.73699942	18.45914832
C	11.78153057	-5.45970541	18.88683175
C	11.34672901	-6.30632053	20.08324309
O	12.10877515	-7.09193633	20.66338069
N	10.06384683	-6.09068329	20.45542789
H	13.87515400	-5.50895239	19.29486937
H	11.65042040	-4.39780782	19.16301549
H	11.06009000	-5.63780885	18.07200467
H	9.45514017	-5.60496049	19.79398427
H	9.59073406	-6.79692626	21.00665114
H	13.32594897	-6.78002746	18.16407728
H	13.49775917	-5.10198081	17.60510831
C	10.79993275	-11.64381986	25.39903282
C	11.23418778	-10.80273263	24.21593791
O	11.05113351	-11.14678817	23.04785639
H	10.50803276	-11.02007679	26.24406988
H	11.57900486	-12.34602490	25.69493984
H	9.93414072	-12.24202992	25.07979542
N	11.83626935	-9.63893676	24.57153295
C	12.09342986	-8.56161015	23.63116911
C	13.50201408	-8.03123156	23.98136533
O	13.80701539	-7.76666961	25.15199224
C	10.97291275	-7.50177295	23.71574290
C	9.60866274	-7.98195648	23.16202903
C	8.61035150	-8.82520064	23.95756553
O	7.64289055	-9.35637241	23.38502638
N	8.74088423	-8.90821881	25.29503806
H	11.82916202	-9.35971400	25.55168236
H	12.09260318	-8.98725831	22.61971839
H	10.88787376	-7.14205371	24.75463177
H	11.27941042	-6.64545689	23.09666204
H	9.00910042	-7.09593115	22.89650826
H	9.75460009	-8.52629884	22.21841561
H	9.56698374	-8.63454141	25.82320188
H	8.05295206	-9.47363258	25.78118044
N	14.38409709	-7.96179118	22.95351197
C	15.77094911	-7.51998056	23.12320266
H	14.00892593	-8.05811657	22.01326100
H	15.82103214	-6.93799349	24.04299675
H	16.49000320	-8.33800858	23.16485166
H	16.04646234	-6.86962532	22.27911271
O	7.54896699	-7.46085680	20.78654415
C	6.65878242	-7.71150113	19.96428304
N	6.58036449	-7.10518445	18.73162459
C	5.54750793	-7.28868983	17.80702382
O	5.54068053	-6.58667477	16.78149784
N	4.57722319	-8.22379694	18.05969792
C	4.59951968	-8.86483194	19.22123960
C	5.60057853	-8.66664109	20.20697833
N	5.59836376	-9.34654829	21.38334216
C	4.54951720	-10.17166733	21.70959613
C	4.48935869	-10.77218138	22.97709703
C	3.43279669	-11.60140551	23.34324715
C	3.38786131	-12.22666214	24.71392624
C	2.40253986	-11.84727153	22.40098044
C	1.24530131	-12.74923055	22.75235275
C	2.46199803	-11.24088938	21.13720891
C	3.51837836	-10.39361353	20.75535058
N	3.59618455	-9.79507802	19.48784312
C	2.61964773	-10.09083296	18.41221040
C	1.56128611	-8.98789565	18.23453231
H	7.32156095	-6.42319599	18.49696851
H	5.30796351	-10.55900386	23.66820474
H	2.46829481	-11.94523425	25.25422198
H	3.39188781	-13.32822073	24.65356215
H	4.24921691	-11.91729623	25.32234001
H	0.54890419	-12.86366068	21.90990760
H	1.59671015	-13.75268534	23.04434683
H	0.67246181	-12.35052888	23.60622501
H	1.66704441	-11.45712819	20.42404366
H	2.19954638	-11.08696828	18.59247495
H	3.20957024	-10.14694285	17.48838297
H	2.14171716	-8.07292761	18.34687733
H	1.13579748	-9.04164461	17.22107596
H	0.74295705	-8.98716766	18.95495337
O	-0.32416876	-9.91017926	22.06652724
H	0.58575622	-10.21572356	21.90525518
H	-0.73831000	-9.94974224	21.19006091
H	6.38379192	-9.19239673	22.04410901

EL222 **Qd** T_{1-H}

C	15.46501253	-9.61300364	29.59103608
C	14.05345503	-9.07339768	29.73694591
O	13.29081309	-9.41298559	30.64109794
H	16.07697014	-8.80500051	29.18995586
H	15.81199533	-9.88599401	30.58800813
H	15.49087041	-10.48457090	28.92004784
N	13.69964590	-8.11905742	28.81972981
C	12.31730512	-7.65486684	28.78482692
C	11.42390189	-8.85346569	28.45546334
O	11.65225347	-9.54669376	27.46161550
C	12.16023426	-6.55207605	27.73831948
H	14.19522172	-8.09839008	27.93041326
H	12.06590947	-7.27627433	29.78782328
H	11.10197882	-6.28319072	27.61642054
H	12.73799855	-5.66478673	28.03963237
H	12.53429574	-6.90850879	26.76713397
N	10.39173405	-9.10241496	29.29160822
C	9.57604256	-10.29299582	29.12698626
H	10.41197058	-8.65457130	30.20061773
H	9.58401726	-10.54799766	28.06700332
H	9.94595720	-11.13801727	29.70801065
H	8.54263343	-10.08165327	29.44188810
C	-2.49229845	-7.05790944	23.10640472
C	-1.17230136	-6.35367771	22.92608329
O	-1.10476561	-5.12013846	22.81653338
H	-2.36085413	-8.04486433	23.57679180
H	-3.09801126	-6.43627630	23.76725296
H	-3.02542927	-7.22190020	22.17023005
N	-0.07977412	-7.15520313	22.90666857
C	1.26171551	-6.62319906	22.69109451
C	1.53117940	-6.37621934	21.18816053
O	2.50731723	-6.86822522	20.61277896
C	2.30835551	-7.56379834	23.28246580
S	3.99568539	-6.89744782	23.20805872
H	-0.21451352	-8.16148351	22.74949059
H	1.30856749	-5.64486733	23.19465058
H	2.08928423	-7.72857117	24.35212924
H	2.28757859	-8.54528053	22.78327068
N	0.59324561	-5.59302262	20.61128610
C	0.69840823	-5.07604969	19.26867261
H	-0.07959956	-5.15694273	21.24704515
H	1.59875380	-5.48351584	18.81197677
H	-0.16335241	-5.28363179	18.63402452
H	0.81274092	-3.97756310	19.29817858
C	8.88986139	-1.76467866	16.94526391
C	8.74535837	-2.66968577	18.17235192
C	8.11501090	-4.04458145	17.96809569
O	8.36836494	-4.96608253	18.77211163
N	7.25743938	-4.23218345	16.95676659
H	7.94306370	-1.70849805	16.37882703
H	9.71715154	-2.84571107	18.65757179
H	8.11288186	-2.17776437	18.93368860
H	7.00615099	-3.47520122	16.33522150
H	6.74743358	-5.12829985	16.89600053
H	9.10033741	-0.73098555	17.21869033
H	9.65288698	-2.12326487	16.25401306
C	13.21279273	-5.73699191	18.45917467
C	11.77800413	-5.52807003	18.94452615
C	11.41604224	-6.45546131	20.11299592
O	12.23854714	-7.22304100	20.63236894
N	10.13388529	-6.34854216	20.52277824
H	13.87517839	-5.50897266	19.29485567
H	11.61490562	-4.48722673	19.27728031
H	11.04035449	-5.69941984	18.14224655
H	9.48091295	-5.75770623	20.00643899
H	9.73559474	-7.04078611	21.14668541
H	13.32597289	-6.78002561	18.16407887
H	13.45140325	-5.08142528	17.60858913
C	10.79998731	-11.64381510	25.39904060
C	11.24540041	-10.75831751	24.24518675
O	11.13584156	-11.09482757	23.06387416
H	10.50803901	-11.02006006	26.24405967
H	11.57897856	-12.34605966	25.69492418
H	9.94437231	-12.23743582	25.04429943
N	11.73673391	-9.55680724	24.64498836
C	12.13375772	-8.51073552	23.72555702
C	13.58712430	-8.13345850	24.03619497
O	13.98081376	-7.99976187	25.20221222
C	11.21334274	-7.27754478	23.83944433
C	9.72865882	-7.65337731	23.70540744
C	9.12548867	-8.22902394	24.96845690
O	8.39221204	-9.35401318	24.70233969
N	9.28363573	-7.68018982	26.10799264
H	11.84345219	-9.37222090	25.64525412
H	12.04993541	-8.91372707	22.70757340
H	11.37106826	-6.77468208	24.80500839
H	11.48937430	-6.57881020	23.03672874
H	9.14278472	-6.75520111	23.45153232
H	9.57360507	-8.37408132	22.88943406
H	8.81334522	-8.20401269	26.85694519
N	14.39353306	-7.96660351	22.96282517
C	15.77094456	-7.51991611	23.12314376
H	13.96257916	-7.96228199	22.04066010
H	15.82103733	-6.93805154	24.04303321
H	16.48999669	-8.33801387	23.16485346
H	16.04231083	-6.87400664	22.27458486
O	7.37833786	-7.62216331	21.20941387
C	6.55420779	-7.82219406	20.30536974
N	6.57364500	-7.17330766	19.09142739
C	5.65262036	-7.37522169	18.05399884
O	5.76315692	-6.69808770	17.02098479
N	4.65573486	-8.30773943	18.20220106
C	4.55733999	-8.95326111	19.35313364
C	5.46305976	-8.76791387	20.42816103
N	5.32144245	-9.44776688	21.59660207
C	4.24825324	-10.27779018	21.82181150
C	4.05564980	-10.88660860	23.07089645
C	2.97824904	-11.73448973	23.31528580
C	2.79032618	-12.37142472	24.66768599
C	2.06222559	-11.98467007	22.26310558
C	0.88405793	-12.90190067	22.47723043
C	2.24461638	-11.35624447	21.02238290
C	3.32617505	-10.49511174	20.76325898
N	3.52445764	-9.87389903	19.52195558
C	2.60576679	-10.10245068	18.38444002
C	1.56134719	-8.98782702	18.23483474
H	7.28909815	-6.44035213	18.94431294
H	4.77794904	-10.67403561	23.86217228
H	1.80724899	-12.11611183	25.09719879
H	2.82783105	-13.47174397	24.60036020
H	3.56644808	-12.04880630	25.37575838
H	0.30337938	-13.03438004	21.55354387
H	1.21070763	-13.89673458	22.82114987
H	0.20355325	-12.50058265	23.24611280
H	1.52182864	-11.56038994	20.23441241
H	2.17670168	-11.10625748	18.47969664
H	3.23838348	-10.10053860	17.48818538
H	2.14169889	-8.07288919	18.34666211

H	1.12862977	-9.02684389	17.22298873
H	0.74290711	-8.98718418	18.95489691
O	-0.36977600	-9.89520524	22.13613082
H	0.52244451	-10.24975378	21.96565838
H	-0.79039343	-9.91408760	21.26140178
H	8.01686371	-9.68676846	25.53534560
H	5.98040095	-9.21638312	22.33923826

EL222 **Qa** S_0'

C	15.46508313	-9.61337671	29.59095215
C	14.02815014	-9.09906918	29.67391174
O	13.35472071	-9.15997584	30.70780814
H	16.07692416	-8.80491966	29.19004910
H	15.81212016	-9.88575572	30.58802818
H	15.53807376	-10.47849673	28.91324411
N	13.55187649	-8.50130135	28.54393601
C	12.32013908	-7.70420055	28.54311235
C	11.10417496	-8.59587760	28.23651107
O	10.66704973	-8.74308709	27.09015736
C	12.43934843	-6.56214011	27.53929126
H	14.06435344	-8.55461638	27.66569212
H	12.20230543	-7.31451278	29.56600483
H	11.47436286	-6.05359638	27.41029700
H	13.20061535	-5.84365636	27.87902014
H	12.73969430	-6.95830373	26.55974734
N	10.55303744	-9.21583838	29.30374225
C	9.57539670	-10.29301572	29.12725407
H	11.08738348	-9.17387135	30.16978194
H	9.58429868	-10.54801078	28.06700803
H	9.94601869	-11.13798780	29.70800912
H	8.56463175	-10.00361831	29.45190395
C	-2.49296918	-7.05816149	23.10714065
C	-1.14747453	-6.36731123	22.99101635
O	-1.03153737	-5.14952502	23.09004923
H	-2.37876250	-8.05107460	23.57464414
H	-3.09811822	-6.43600446	23.76689672
H	-3.02502222	-7.22183750	22.16998658
N	-0.06636309	-7.18261895	22.76826612
C	1.26577147	-6.59635131	22.66024755
C	1.28393455	-5.47790508	21.60626358
O	2.01771753	-4.50043187	21.70101695
C	2.25625809	-7.70604822	22.22601179
S	3.95414177	-7.00182491	21.96209421
H	-0.16018790	-8.19858884	22.86675400
H	1.58965539	-6.13925123	23.60899139
H	2.30539058	-8.48883616	22.99528459
H	1.90059535	-8.15239611	21.28692358
N	0.50613871	-5.74786073	20.53276551
C	0.69830805	-5.07554236	19.26890439
H	-0.01435482	-6.61627690	20.54472544
H	1.59897011	-5.48301741	18.81195936
H	-0.16315601	-5.28459038	18.63407733
H	0.81427236	-3.99478970	19.42141370
C	8.88984064	-1.76489413	16.94497922
C	8.77999043	-2.64155284	18.20311162
C	8.03652429	-3.97118683	18.11670248
O	8.16894116	-4.81234850	19.03446040
N	7.21033260	-4.19181945	17.08826962
H	7.93294048	-1.71892611	16.39660389
H	9.77364216	-2.86873340	18.61933688
H	8.24857265	-2.09108614	19.00037371
H	7.05021822	-3.48582671	16.38228457
H	6.60165457	-5.01927790	17.12261084
H	9.10013563	-0.73102205	17.21897926
H	9.65302468	-2.12299115	16.25402417
C	13.21306795	-5.73697741	18.45890934
C	11.76831183	-5.53862235	18.92132876
C	11.36112401	-6.57585231	19.97336928
O	12.19062131	-7.24519242	20.60190082
N	10.02439672	-6.71075181	20.12645750
H	13.87502101	-5.50899742	19.29498537
H	11.62219387	-4.53722525	19.36580373
H	11.05962638	-5.60260173	18.07888710
H	9.39164721	-6.01565945	19.72661398
H	9.66290373	-7.32134240	20.85020682
H	13.32598295	-6.78002349	18.16407633
H	13.45923454	-5.07940288	17.61205302
C	10.79988878	-11.64410086	25.39862382
C	11.34386733	-10.78881680	24.25262614
O	11.59900183	-11.26743910	23.14500095
H	10.50815775	-11.01995381	26.24401987
H	11.57901385	-12.34591588	25.69516586
H	9.94237689	-12.22853474	25.03333033
N	11.51765438	-9.47648771	24.56311253
C	12.12411246	-8.51669575	23.66643258
C	13.62082268	-8.33551102	23.99143869
O	14.06503099	-8.44887682	25.14065054
C	11.44203759	-7.13555668	23.78904750
C	9.93377078	-7.16324053	23.50732877
C	9.04640244	-6.78642199	24.69437357
O	9.46041077	-6.21775060	25.69929931
N	7.72939886	-7.11983525	24.52706190
H	11.35058296	-9.16383703	25.52332020
H	12.01447677	-8.88722344	22.63774503
H	11.59949359	-6.73637484	24.80120810
H	11.94533495	-6.46302388	23.08172590
H	9.68023703	-6.43440804	22.72138064
H	9.61192369	-8.14612441	23.12729261
H	7.38388967	-7.31256996	23.58870560
H	7.07509215	-6.69386693	25.17608727
N	14.39434826	-7.97246976	22.93959831
C	15.77113914	-7.51986652	23.12286467
H	13.93720246	-7.82929853	22.04096808
H	15.82104516	-6.93803550	24.04302072
H	16.48994917	-8.33804361	23.16502967
H	16.04298802	-6.87256686	22.27665283
O	7.15886429	-7.57897202	21.57098873
C	6.25458001	-7.61025873	20.74369127
N	6.28429099	-6.93616163	19.56359452
C	5.36344018	-7.12083026	18.51323864
O	5.45627872	-6.42057228	17.50900051
N	4.42569085	-8.11755984	18.60785739
C	4.24265860	-8.74925487	19.73887401
C	4.95043360	-8.38369843	21.02720714
N	5.23476808	-9.56513559	21.76658867
C	4.17465323	-10.44489446	21.97077727
C	4.05635955	-11.22326900	23.12809776
C	2.99220718	-12.11074269	23.31403980
C	2.89880595	-12.94050352	24.56901931
C	1.99976267	-12.21088065	22.30950067
C	0.83216111	-13.15585691	22.47237020
C	2.11387638	-11.42015765	21.15784529
C	3.19501147	-10.55268765	20.96048895
N	3.34954190	-9.77485403	19.78267110
C	2.59368795	-10.08370791	18.53500244
C	1.56095111	-8.98775966	18.23530332
H	7.03206003	-6.23541898	19.39155694
H	4.82145873	-11.12397709	23.90187391
H	2.90941373	-14.01812102	24.33404572
H	3.73354021	-12.73364427	25.25300106
H	1.95740049	-12.74680808	25.10995694
H	0.16976849	-13.13230810	21.59470666
H	1.17533047	-14.19524675	22.60447560
H	0.22648127	-12.90760007	23.35976277
H	1.33293984	-11.48959174	20.40103530
H	2.16154176	-11.08367468	18.64001237
H	3.33324297	-10.12181508	17.72436851
H	2.14205615	-8.07302757	18.34593233
H	1.16848123	-9.07600970	17.21328499
H	0.74288305	-8.98718566	18.95486839
O	-0.24413060	-10.05879096	23.02302416
H	0.63763044	-10.45234231	23.13493976
H	-0.56196433	-10.47618693	22.20731485
H	5.87707576	-9.42342435	22.54212931

EL222 **Qb** S_0'

C	15.46509413	-9.61311572	29.59105015
C	14.02630993	-9.10825919	29.67767943
O	13.33563821	-9.22791876	30.69468259
H	16.07694914	-8.80496262	29.19000010
H	15.81208814	-9.88593469	30.58799019
H	15.53752040	-10.47977814	28.91524900
N	13.55577266	-8.45474899	28.57347606
C	12.31862262	-7.67069014	28.61993439
C	11.10704553	-8.56139266	28.30007468
O	10.65960897	-8.65974829	27.13957632
C	12.39870560	-6.48815372	27.65940328
H	14.08024659	-8.44195455	27.70103942
H	12.21026093	-7.32079052	29.65818211
H	11.45024354	-5.93132119	27.65908746
H	13.20823365	-5.81156340	27.97133088
H	12.60216573	-6.83862739	26.63820327
N	10.57346045	-9.23479822	29.32623155
C	9.57565171	-10.29303276	29.12709310
H	11.10535000	-9.21617468	30.19610136
H	9.58409871	-10.54803375	28.06701100
H	9.94597874	-11.13796478	29.70806812
H	8.57141009	-9.98938498	29.45612011
C	-2.49298818	-7.05814048	23.10711964
C	-1.14699614	-6.36520097	22.98987005
O	-1.03970330	-5.14573153	23.07954781
H	-2.37911744	-8.05081345	23.57555136
H	-3.09812022	-6.43602145	23.76691170
H	-3.02503222	-7.22184353	22.16999361
N	-0.05936792	-7.17504579	22.77516791
C	1.27066960	-6.57949828	22.66272950
C	1.27084517	-5.45698194	21.61328909
O	1.97842663	-4.46149375	21.71464151
C	2.27155700	-7.67687013	22.21584132
S	3.96240933	-6.95483032	21.94160018
H	-0.14090758	-8.18990642	22.89368113
H	1.59644628	-6.12419492	23.61159865
H	2.33455059	-8.46231067	22.98144438
H	1.91336277	-8.12459727	21.27826788
N	0.50585289	-5.74414030	20.53402252
C	0.69830405	-5.07548737	19.26891540
H	0.01023540	-6.62707359	20.54133797
H	1.59895811	-5.48305139	18.81196635
H	-0.16314601	-5.28461238	18.63407135
H	0.81423448	-3.99471467	19.42074212
C	8.88998162	-1.76496513	16.94496022
C	8.75751342	-2.64256076	18.19554804
C	7.97457790	-3.94453484	18.08594500
O	8.17074824	-4.85483760	18.92092860
N	7.04410204	-4.07204084	17.13381501
H	7.94182347	-1.71027928	16.38273395
H	9.74315759	-2.90480069	18.60841160
H	8.23998574	-2.08151560	18.99496034
H	6.82860112	-3.30994124	16.50530066
H	6.43185381	-4.89959616	17.15412060
H	9.10002466	-0.73100205	17.21898926
H	9.65302468	-2.12295615	16.25400618
C	13.21301197	-5.73698841	18.45900734
C	11.77039446	-5.52203841	18.90890140
C	11.35481759	-6.51599659	19.99880915
O	12.16690274	-7.26977656	20.55418856
N	10.03802833	-6.49415025	20.28294405
H	13.87503001	-5.50901240	19.29498241
H	11.62065592	-4.50202017	19.30678300
H	11.06219739	-5.62289759	18.06882961
H	9.41399584	-5.82157703	19.83490407
H	9.64560175	-7.13254402	20.96308366
H	13.32604296	-6.77999148	18.16398431
H	13.46961451	-5.08451419	17.61060524
C	10.80000377	-11.64409683	25.39884485
C	11.29526039	-10.80235242	24.23635764
O	11.37425560	-11.24035957	23.08780869
H	10.50802874	-11.01993079	26.24395891
H	11.57901883	-12.34596388	25.69504187
H	9.93402557	-12.22848971	25.05609828
N	11.65550796	-9.54205864	24.58887081
C	12.12283005	-8.55359010	23.64296436
C	13.57519866	-8.19108585	23.99799054
O	13.95804162	-8.12067574	25.17410481
C	11.21325723	-7.30986936	23.64448257
C	9.73917733	-7.64867922	23.33635536
C	8.95779415	-8.16090967	24.51753343
O	9.02420716	-7.30417663	25.58592102
N	8.28487653	-9.24692136	24.48614724
H	11.53758319	-9.23612064	25.55316802
H	12.08804638	-9.01112796	22.64520484
H	11.26817159	-6.80514788	24.62157238
H	11.59324719	-6.61792666	22.88080974
H	9.22938601	-6.72711424	23.01186831
H	9.65556907	-8.38429629	22.52429599
H	7.81151801	-9.36784434	25.39318816
N	14.39207841	-7.96125004	22.94416818
C	15.77105712	-7.51997553	23.12298368
H	13.97426625	-7.94180426	22.01608522
H	15.82106415	-6.93797248	24.04297971
H	16.48998420	-8.33801561	23.16498168
H	16.04907631	-6.87152369	22.27877854
O	7.17997153	-7.47515031	21.47369854
C	6.26114954	-7.52532917	20.67237628
N	6.25123154	-6.86166284	19.48051666
C	5.30784228	-7.05834316	18.46288759
O	5.35219220	-6.35421885	17.45478508
N	4.39069982	-8.07368475	18.58176752
C	4.24506138	-8.70123928	19.72025607
C	4.97633515	-8.31917283	20.98952067
N	5.30583056	-9.48800260	21.72629310
C	4.27314344	-10.38853501	21.95784915
C	4.20398483	-11.17083446	23.11743552
C	3.15868701	-12.07240025	23.33778715
C	3.11846451	-12.90363166	24.59489724
C	2.13431305	-12.18619585	22.36738911
C	0.98211223	-13.14228198	22.57084078
C	2.20158956	-11.39729994	21.21072417
C	3.26312687	-10.51486174	20.97686407
N	3.36794412	-9.74026870	19.79043805
C	2.59960892	-10.07605897	18.55559452
C	1.56108911	-8.98771663	18.23554331
H	7.00959794	-6.17909420	19.27984413
H	4.99533300	-11.06004210	23.86272522
H	3.14745546	-13.98111354	24.35994630
H	3.96751945	-12.67772552	25.25494621
H	2.18841091	-12.73140558	25.16210281
H	0.30391100	-13.14399111	21.70478231
H	1.33912571	-14.17495672	22.71742325
H	0.38882017	-12.88229360	23.46354565
H	1.39909774	-11.48122968	20.47786215
H	2.16904614	-11.07371530	18.68660341
H	3.33296387	-10.13218473	17.73987663
H	2.14200316	-8.07297857	18.34580633
H	1.17504953	-9.08720799	17.21241481
H	0.74281405	-8.98724762	18.95478938
O	-0.14815292	-10.05668955	23.08697349
H	0.75148050	-10.41274482	23.18343562
H	-0.46344959	-10.49249501	22.28006783
H	5.99801557	-9.34495853	22.46176036
H	9.40724338	-7.80512963	26.34317843

EL222 **Qc** S_0'

C	15.46509593	-9.61303137	29.59112074
C	14.02993130	-9.10728455	29.70733008
O	13.32648657	-9.31423090	30.70021037
H	16.07694511	-8.80498046	29.18995708
H	15.81208407	-9.88599173	30.58797643
H	15.52494334	-10.48239312	28.91805613
N	13.57918273	-8.35321560	28.66141738
C	12.31035946	-7.62790250	28.73656960
C	11.15931560	-8.56222510	28.33049078
O	10.85470398	-8.70974787	27.13476780
C	12.34998838	-6.39068472	27.84486778
H	14.11185297	-8.27558249	27.79852540
H	12.17332658	-7.34305835	29.79074212
H	11.39121160	-5.85336232	27.89801126
H	13.15079258	-5.71555700	28.18220161
H	12.53911215	-6.68035920	26.80156028
N	10.54150079	-9.20816415	29.33404913
C	9.57584687	-10.29306054	29.12699054
H	11.00301791	-9.14910252	30.23960640
H	9.58402531	-10.54803147	28.06700967
H	9.94589597	-11.13795208	29.70813903
H	8.56363298	-10.00951747	29.45097715
C	-2.49299471	-7.05813371	23.10710064
C	-1.13986533	-6.37271868	22.99527601
O	-1.03037458	-5.15431284	23.10032892
H	-2.38522254	-8.05142327	23.57645398
H	-3.09810384	-6.43602177	23.76692731
H	-3.02502415	-7.22184887	22.16998954
N	-0.05001140	-7.17886312	22.77197682
C	1.27884411	-6.57352885	22.65877723
C	1.26273684	-5.44418205	21.61711335
O	1.94881888	-4.43469829	21.72449183
C	2.29775450	-7.65374228	22.20208652
S	3.98275049	-6.90746213	21.93042163
H	-0.12463936	-8.19732670	22.85956242
H	1.60035673	-6.12113940	23.61030793
H	2.37622724	-8.44105516	22.96445202
H	1.94495009	-8.10388527	21.26359808
N	0.50462148	-5.74133730	20.53464277
C	0.69830540	-5.07548414	19.26892154
H	0.03150307	-6.63664726	20.53686748
H	1.59895446	-5.48305006	18.81195698
H	-0.16314298	-5.28461268	18.63406669
H	0.81406885	-3.99468984	19.42017913
C	8.88995458	-1.76496588	16.94497996
C	8.72686257	-2.65128764	18.18311909
C	7.93316120	-3.93578290	17.99950914
O	8.26139006	-4.97448102	18.61364045
N	6.85214723	-3.91786688	17.21066842
H	7.95301964	-1.70945083	16.36453448
H	9.69964360	-2.93622951	18.60809906
H	8.19334776	-2.09284991	18.97386559
H	6.53427543	-3.05953399	16.78016024
H	6.25847222	-4.75868267	17.15710653
H	9.10002863	-0.73100200	17.21898725
H	9.65303060	-2.12295259	16.25401004
C	13.21300953	-5.73696931	18.45893130
C	11.77518817	-5.48162446	18.89923085
C	11.35032488	-6.35078227	20.08788344
O	12.13153020	-7.11307009	20.67379844
N	10.05718777	-6.18691507	20.44066470
H	13.87502165	-5.50902912	19.29499352
H	11.63529027	-4.42565234	19.19308975
H	11.05646344	-5.65709577	18.08041166
H	9.43067270	-5.62917167	19.85718561
H	9.62924594	-6.82013132	21.10364039
H	13.32602470	-6.78000926	18.16404008
H	13.48369809	-5.09533798	17.60610671
C	10.79993127	-11.64413276	25.39881025
C	11.25234999	-10.80491660	24.22210420
O	11.09920207	-11.15425502	23.05143865
H	10.50804933	-11.01992661	26.24396320
H	11.57905095	-12.34592017	25.69506140
H	9.93469647	-12.23915390	25.07261343
N	11.83968556	-9.63718720	24.58912624
C	12.10382497	-8.55823717	23.65446508
C	13.51398915	-8.03498560	23.99755566
O	13.82885787	-7.77395437	25.16633411
C	10.99067697	-7.49421921	23.75276409
C	9.62534881	-7.97650950	23.20905881

C 8.65576062 -8.86764415 23.98974030
O 7.75661831 -9.47847757 23.39160940
N 8.72860087 -8.88716618 25.33615423
H 11.81274192 -9.35874069 25.56933243
H 12.09566050 -8.97872533 22.64084616
H 10.91253871 -7.14052375 24.79406278
H 11.29383272 -6.63372932 23.13716563
H 9.00509122 -7.09156808 22.99049585
H 9.76790101 -8.49281906 22.24890326
H 9.54085598 -8.59377814 25.87589820
H 8.06990203 -9.49829214 25.80787913
N 14.38545300 -7.96319434 22.96137973
C 15.77100446 -7.52006295 23.12312376
H 14.00186136 -8.05641944 22.02425852
H 15.82108749 -6.93790977 24.04293917
H 16.49000966 -8.33799699 23.16491437
H 16.04347979 -6.87066145 22.27739194
O 7.15597826 -7.16345448 21.34149069
C 6.25367798 -7.33656810 20.53775015
N 6.23086478 -6.76463671 19.30225865
C 5.25359520 -6.98722422 18.32513561
O 5.25909794 -6.29849082 17.30338462
N 4.34913803 -8.00287369 18.50090448
C 4.26030499 -8.61332569 19.65644153
C 5.03387972 -8.20703959 20.89177825
N 5.46806329 -9.36128122 21.58336047
C 4.49743878 -10.30741114 21.86433591
C 4.55378300 -11.11471707 23.00961190
C 3.55379337 -12.04451060 23.30074226
C 3.64061653 -12.89373901 24.54358192
C 2.44967869 -12.16924207 22.42078945
C 1.33751628 -13.14913726 22.71377769
C 2.40010351 -11.36768152 21.27206327
C 3.41580589 -10.45279269 20.96480640
N 3.41323735 -9.67289190 19.77629499
C 2.61488832 -10.05923955 18.57283321
C 1.56110625 -8.98768478 18.23558860
H 7.01170655 -6.12471701 19.04883979
H 5.40909955 -10.98354339 23.67692061
H 3.68176801 -13.96712823 24.29133863
H 4.53487936 -12.65022572 25.13409404
H 2.75590973 -12.75681249 25.18788850
H 0.60249668 -13.18103437 21.89587832
H 1.72691731 -14.17107388 22.85175960
H 0.79884886 -12.88693136 23.64032795
H 1.54602402 -11.46787801 20.60154141
H 2.19406922 -11.05332870 18.75295716
H 3.32890802 -10.14241462 17.74167160
H 2.14202313 -8.07298798 18.34577881
H 1.18189376 -9.09798463 17.21148060
H 0.74279536 -8.98726556 18.95476827
O -0.01555555 -10.07555318 22.99897909
H 0.89754563 -10.40933004 22.96076096
H -0.45266488 -10.55487379 22.27833906
H 6.25996280 -9.24539436 22.23175246

EL222 **Qd** S_0'

C 15.46509911 -9.61282768 29.59102412
C 14.05333708 -9.07454753 29.73805422
O 13.29050759 -9.41815227 30.64048082
H 16.07708318 -8.80504364 29.19004111
H 15.81204115 -9.88610873 30.58795823
H 15.49090507 -10.48450771 28.92016072
N 13.69923791 -8.11720805 28.82416188
C 12.31641325 -7.65469697 28.79044329
C 11.42423210 -8.85400794 28.45989737
O 11.65632591 -9.54595370 27.46828684
C 12.15827275 -6.54996839 27.74625153
H 14.19491575 -8.09360722 27.93498371
H 12.06474491 -7.27809327 29.79412409
H 11.09981919 -6.28184577 27.62499607
H 12.73529096 -5.66284761 28.04942850
H 12.53304487 -6.90405772 26.77449890
N 10.38911298 -9.10142434 29.29257138
C 9.57470671 -10.29274774 29.12772912
H 10.40379712 -8.64975986 30.19977840
H 9.58430768 -10.54806978 28.06702200
H 9.94650670 -11.13809480 29.70754016
H 8.54101340 -10.08301770 29.44235373
C -2.49269018 -7.05836750 23.10724267
C -1.14693747 -6.36588943 22.99193013
O -1.03554812 -5.14733655 23.08939352
H -2.37839326 -8.05126584 23.57482930
H -3.09822022 -6.43593244 23.76673469
H -3.02514822 -7.22171351 22.17003658
N -0.06231515 -7.17759907 22.77326774
C 1.26850580 -6.58660117 22.66124240
C 1.27590618 -5.46517652 21.61060347
O 1.99492991 -4.47740733 21.70979668
C 2.26428335 -7.68918731 22.21840846
S 3.95416402 -6.97167207 21.93018954
H -0.15255163 -8.19409168 22.86992361
H 1.59468986 -6.13086023 23.60975621
H 2.32880733 -8.46841896 22.99038393
H 1.90030808 -8.14305624 21.28607467
N 0.50537892 -5.74476298 20.53384403
C 0.69834105 -5.07560237 19.26891640
H -0.00055949 -6.62178531 20.54308949
H 1.59899012 -5.48296042 18.81194936
H -0.16317701 -5.28459838 18.63410934
H 0.81430605 -3.99473122 19.42043356
C 8.88975566 -1.76493413 16.94490222
C 8.77956085 -2.64130694 18.20188287
C 8.04823138 -3.97611732 18.10431073
O 8.21406950 -4.84097492 18.99272615
N 7.19362907 -4.17932617 17.09505912
H 7.93383829 -1.71852845 16.39507155
H 9.77287507 -2.86024526 18.62278103
H 8.23771075 -2.09498938 18.99513888
H 7.00391971 -3.45587787 16.41470863
H 6.59424349 -5.01476579 17.12527080
H 9.10018368 -0.73103705 17.21899624
H 9.65307569 -2.12293415 16.25405116
C 13.21306695 -5.73691539 18.45885034
C 11.77535052 -5.52284472 18.93728515
C 11.40520421 -6.43789326 20.11304767
O 12.22329716 -7.19932238 20.64640368
N 10.11838135 -6.33252613 20.50993823
H 13.87497201 -5.50904542 19.29503839
H 11.61375847 -4.47890425 19.26112041
H 11.04378123 -5.70049127 18.13020174

H 9.46950056 -5.70375180 20.03291230
H 9.74415064 -6.97273524 21.20021979
H 13.32601295 -6.78002349 18.16408633
H 13.45498906 -5.08285881 17.60809874
C 10.80004880 -11.64423082 25.39876484
C 11.24620153 -10.75552304 24.24773532
O 11.13478791 -11.08611980 23.06489415
H 10.50811076 -11.01985977 26.24393489
H 11.57899681 -12.34596086 25.69510384
H 9.94445603 -12.23707428 25.04292764
N 11.74026178 -9.55652741 24.65240320
C 12.13560402 -8.50661799 23.73693291
C 13.59004023 -8.13181609 24.04342327
O 13.98667291 -7.99653865 25.20806047
C 11.21796087 -7.27249468 23.86048087
C 9.73269903 -7.64500901 23.72592078
C 9.12977919 -8.23037900 24.98483314
O 8.40073317 -9.35618386 24.71192145
N 9.28618922 -7.68620328 26.12650257
H 11.84702694 -9.37561382 25.65351749
H 12.04728137 -8.90493437 22.71748442
H 11.37759873 -6.77608446 24.82889339
H 11.49534252 -6.56854474 23.06247158
H 9.14607118 -6.74448935 23.48261598
H 9.57713780 -8.36011638 22.90497022
H 8.81752614 -8.21380163 26.87366873
N 14.39359508 -7.96794635 22.96737022
C 15.77095012 -7.52015154 23.12337369
H 13.96053664 -7.96527692 22.04647518
H 15.82114512 -6.93789448 24.04292674
H 16.49005718 -8.33796661 23.16466666
H 16.03997737 -6.87463345 22.27381644
O 7.17299216 -7.56763848 21.55784688
C 6.27458725 -7.59703168 20.73191639
N 6.30461473 -6.93329662 19.53946753
C 5.38449487 -7.12252521 18.49521714
O 5.47635491 -6.43432845 17.48085548
N 4.43839937 -8.11437647 18.59766194
C 4.25524289 -8.73737595 19.73270221
C 4.96273790 -8.36175747 21.01725617
N 5.24702965 -9.53173371 21.77200734
C 4.19577201 -10.41791171 21.97722519
C 4.08565242 -11.19748346 23.13507819
C 3.02749244 -12.09075635 23.32634327
C 2.94422617 -12.92101757 24.58185784
C 2.03109149 -12.19660630 22.32671353
C 0.86788596 -13.14607914 22.49577275
C 2.13738998 -11.40697552 21.17319989
C 3.21289777 -10.53419365 20.96883381
N 3.35803207 -9.76112553 19.78618388
C 2.59875996 -10.07909454 18.54210839
C 1.56050311 -8.98783766 18.23458332
H 7.07096446 -6.25743180 19.35528697
H 4.85466668 -11.09313336 23.90438956
H 2.96238885 -13.99872445 24.34751185
H 3.77995571 -12.70800780 25.26292398
H 2.00331410 -12.73450688 25.12623888
H 0.20457454 -13.12952113 21.61858780
H 1.21511443 -14.18353437 22.63251676
H 0.26164010 -12.89584715 23.38222037
H 1.35374463 -11.48216055 20.41964578
H 2.16996485 -11.07986387 18.65387861
H 3.33656136 -10.11983526 17.72995006
H 2.14211316 -8.07310160 18.34624332
H 1.17063373 -9.08025608 17.21214329
H 0.74317905 -8.98705564 18.95520235
O -0.22161596 -10.05544096 23.02802865
H 0.66728807 -10.43821015 23.12166069
H -0.54903681 -10.47495675 22.21719030
H 5.90420514 -9.38298653 22.53521254
H 8.02217490 -9.69253395 25.54203090

AsLOV2 **Qa** S_0

C 19.02911037 2.11195615 13.81256201
C 19.30841996 0.92811114 12.90802145
O 19.97113890 -0.05142552 13.27262278
H 18.15298033 2.65200919 13.45306497
H 18.86502541 1.75033206 14.83652136
H 19.89300043 2.77599720 13.82123501
N 18.78844241 1.03756990 11.65844368
C 18.92582222 0.00029510 10.64343287
C 20.27827158 0.08847712 9.89253908
O 20.33388234 0.11184092 8.65975300
C 17.77357289 0.08588321 9.64488112
S 16.11350581 -0.25518191 10.35742241
H 18.43955923 1.95128294 11.35037811
H 18.90571719 -0.96910868 11.16753880
H 17.91766330 -0.65733082 8.85087309
H 17.77225714 1.07139146 9.16049925
H 16.18448446 0.66895696 11.34997477
N 21.35097281 0.13122909 10.71548607
C 22.71324862 0.02577400 10.23641973
H 21.16862547 -0.04114941 11.70622399
H 22.71790464 0.02789200 9.14599963
H 23.15216289 -0.93642132 10.55695250
H 23.37787868 0.80625006 10.60769578
C 25.51286784 -3.72105827 7.23795750
C 25.85042503 -2.22594175 7.23851637
C 24.67982770 -1.39188367 6.71376182
C 24.68494611 0.06480965 7.17595169
O 25.35875343 0.46088713 8.12810598
N 23.85737598 0.87867853 6.46981228
H 25.30198129 -4.09402485 6.22032542
H 26.75697001 -2.01920023 6.64334040
H 26.07557947 -1.89592534 8.26529618
H 23.72299549 -1.81351493 7.07535626
H 24.61034392 -1.43027662 5.61480181
H 23.69039272 1.80790009 6.83890621
H 23.18355876 0.48623116 5.81141566
H 26.30805291 -4.33195531 7.66596855
H 24.60706075 -3.84099728 7.83209258
C 23.25608467 -6.51500147 3.78403627
C 21.82410691 -5.97317467 3.91474084
C 21.63412264 -4.45986910 3.97576276
O 20.47507498 -3.99402230 3.92048230
N 22.69086029 -3.65322295 4.14283466
H 23.91602072 -5.85800442 4.34998331
H 21.36207971 -6.33332540 4.85108003
H 21.16651342 -6.33567092 3.10902811
H 22.53203007 -2.64848900 4.29541712
H 23.63265609 -4.01359631 4.21054802
H 23.34093867 -7.52801157 4.17698730

H 23.60020843 -6.50885603 2.73810132
C 19.24991640 -5.26288138 -1.04999507
C 19.39599864 -4.77275063 0.38839325
C 18.15071106 -4.03257146 0.88930396
O 17.12130123 -3.92594908 0.21127482
N 18.29315383 -3.50008242 2.12293841
H 18.37510433 -5.91113742 -1.09602308
H 20.26347054 -4.10054834 0.50634743
H 19.57782447 -5.61849398 1.07663822
H 17.51245651 -3.03388439 2.56725824
H 19.08495635 -3.75043046 2.71562214
H 20.13727087 -5.83289353 -1.36547060
H 19.12095236 -4.41299632 -1.71998512
C 6.29793847 -6.61099248 1.89513814
C 7.35737910 -6.36951133 2.95953880
O 7.07771057 -6.21763185 4.14879701
H 6.66850918 -7.25504966 1.08299466
H 6.01606141 -5.65101240 1.46293511
H 5.42297537 -7.06901153 2.35494117
N 8.64643181 -6.31613486 2.51007606
C 9.74475581 -6.01331853 3.42199282
C 9.87243067 -4.49175424 3.60043724
O 10.24382107 -3.77394807 2.66356503
C 11.08410771 -6.51883811 2.88674723
C 11.05114140 -7.98538745 2.47932306
O 10.61803199 -8.87233255 3.21275115
N 11.53964740 -8.21179081 1.23259963
H 8.83325874 -6.30316748 1.51352296
H 9.50135480 -6.50347107 4.37339431
H 11.83195158 -6.40194932 3.68820459
H 11.40367901 -5.88506136 2.05126755
H 11.61092525 -9.16954035 0.90768199
H 11.93521274 -7.45059151 0.68096200
N 9.58650413 -4.01429600 4.82648769
C 9.75203570 -2.59894219 5.11999837
H 9.09884158 -4.62181026 5.47493725
H 10.66400077 -2.29001816 4.60899033
H 9.85483483 -2.45563777 6.20385557
H 8.90496866 -2.02203314 4.74802134
C 11.66098082 -0.84004706 1.57596111
C 12.84230965 -1.60413154 1.00190328
O 13.74527659 -1.06087046 0.36126067
H 11.17401281 -1.45698711 2.33201817
H 10.95099677 -0.57896304 0.79101606
H 12.03282594 0.09518036 2.01946263
N 12.81326565 -2.93696347 1.28031374
C 13.90675789 -3.82513519 0.93701014
C 13.42768478 -4.87933856 -0.06725161
O 12.45579398 -5.61024580 0.17707981
C 14.49411886 -4.51486303 2.18144891
C 15.06830343 -3.51688246 3.18977492
C 14.07320064 -2.94090777 4.19642450
O 12.91891689 -3.35393472 4.31714527
N 14.57792604 -1.92871915 4.95191725
H 12.05121993 -3.31007397 1.84942002
H 14.69020497 -3.20699811 0.47949409
H 15.30072565 -5.17905774 1.83623204
H 13.72077219 -5.13175218 2.66229207
H 15.53864781 -2.67025931 2.66315499
H 15.86843002 -3.99256199 3.78072739
H 14.01256757 -1.60216249 5.72774612
H 15.57802791 -1.72375513 4.96968161
N 14.13121011 -4.95548846 -1.21746424
C 13.90706899 -6.01797344 -2.19610516
H 14.99249165 -4.41750019 -1.26888018
H 13.08999995 -6.62896546 -1.81294813
H 13.66696894 -5.61603606 -3.19129895
H 14.81297409 -6.62204149 -2.23098516
O 17.85736155 3.57889678 10.70484145
H 18.47219428 4.32922931 10.69928202
H 17.54819281 3.52392671 9.78490792
N 20.65672560 0.71528230 5.61982561
C 20.90040606 -0.52432793 5.09569951
O 22.05621453 -0.88544165 4.82105217
N 19.84856039 -1.41159274 4.84627074
C 18.52368471 -1.16192544 5.09200941
O 17.63194643 -1.94798651 4.78174879
C 18.27519731 0.14822695 5.76368170
N 17.05562134 0.43282031 6.12283843
C 16.83241009 1.64511961 6.71110128
C 15.52353361 1.95085510 7.14757911
C 15.21199802 3.15669516 7.74969349
C 13.81682834 3.44717434 8.23514472
C 16.25368137 4.12129049 7.90955016
C 15.95537273 5.44658366 8.56002013
C 17.55465306 3.83183337 7.49306878
C 17.87875788 2.59337288 6.89987500
N 19.16517459 2.26003508 6.50399565
C 19.42403651 1.02429288 5.95737211
C 20.27902629 3.22177501 6.69346274
C 20.85342951 3.21130023 8.11838060
H 20.09659735 -2.33893839 4.44263486
H 14.76370959 1.18297922 6.99564592
H 13.80727521 3.63840972 9.32113317
H 13.40017825 4.34527140 7.75027009
H 13.14248629 2.60427782 8.03246978
H 16.84305074 6.09325689 8.59614018
H 15.16024168 5.98198245 8.01669109
H 15.59321260 5.30111136 9.59113544
H 18.32305398 4.58925410 7.63518917
H 19.89491229 4.21132755 6.41462539
H 21.04806000 2.94540989 5.96549752
H 21.49777457 2.34280317 8.25081059
H 21.45369336 4.12338262 8.26313015
H 20.05569846 3.19804023 8.86183664

AsLOV2 **Qb** S_0

C 19.02901003 2.11201190 13.81290232
C 19.33758482 0.93063716 12.91945237
O 20.08202926 0.00621998 13.27249133
H 18.15302372 2.65200421 13.45295427
H 18.86557110 1.75480391 14.83891430
H 19.89301646 2.77597988 13.82111547
N 18.77099115 0.98225853 11.68756410
C 18.93291081 -0.06620016 10.68361504
C 20.27344199 0.05980745 9.91185358
O 20.30815384 0.08740103 8.67901325
C 17.77047308 -0.03176600 9.69568266
S 16.11895718 -0.37864483 10.45689831
H 18.20286105 1.79564016 11.42784336
H 18.95760857 -1.03182057 11.21664227
H 17.92600647 -0.78404192 8.91383566

H	17.73416993	0.94766727	9.20281745
H	16.45225122	-0.01735633	11.72344141
N	21.35333093	0.11945903	10.72197209
C	22.71316734	0.02581827	10.23616054
H	21.18120889	-0.03876603	11.71813095
H	22.71792776	0.02805125	9.14600059
H	23.15844660	-0.93487926	10.55351385
H	23.37795001	0.80609787	10.60788719
C	25.51302633	-3.72099198	7.23808324
C	25.86549891	-2.22856057	7.26617715
C	24.69982340	-1.36742054	6.77920523
C	24.74711497	0.08374613	7.25699042
O	25.43069915	0.44735724	8.21544949
N	23.94287345	0.92806696	6.56262404
H	25.32086043	-4.08038536	6.21189536
H	26.76606365	-2.01992772	6.66292943
H	26.10940327	-1.92702944	8.29754604
H	23.74562485	-1.77158339	7.16804160
H	24.59924337	-1.39558052	5.68307726
H	23.81340288	1.86369894	6.93025406
H	23.27822079	0.56749489	5.87742401
H	26.30801185	-4.33201364	7.66596289
H	24.60697518	-3.84099961	7.83195992
C	23.25601503	-6.51499555	3.78394899
C	21.82557400	-5.95419197	3.94175514
C	21.70614975	-4.42997373	3.98375749
O	20.75055719	-3.84335484	3.43578834
N	22.63247336	-3.73321353	4.66528724
H	23.91599992	-5.85802030	4.35002607
H	21.39301435	-6.30535605	4.89659556
H	21.14920832	-6.30127303	3.14794073
H	22.55879797	-2.70899627	4.71708607
H	23.35607462	-4.19321406	5.20155324
H	23.34100455	-7.52799273	4.17701805
H	23.58511994	-6.49723980	2.73425887
C	19.24992407	-5.26294371	-1.04997246
C	19.36966588	-4.78121381	0.39467402
C	18.07203060	-4.14698476	0.90956475
O	17.06484051	-4.02246403	0.19893738
N	18.12568473	-3.75731666	2.20086183
H	18.37503676	-5.91104672	-1.09600850
H	20.18288928	-4.04728019	0.52675662
H	19.62117409	-5.61840884	1.06970480
H	17.37044730	-3.22184168	2.61099994
H	18.98659854	-3.85590390	2.73761460
H	20.14328354	-5.82731489	-1.35760501
H	19.12102597	-4.41301003	-1.72001626
C	6.29800456	-6.61101053	1.89501106
C	7.35982067	-6.33631890	2.95003271
O	7.09241725	-6.15567684	4.13847731
H	6.67376179	-7.25580533	1.08638864
H	6.01600717	-5.65100055	1.46299620
H	5.42299405	-7.06899116	2.35499689
N	8.64271773	-6.26019236	2.48696608
C	9.75810060	-6.01391406	3.39366577
C	9.93049215	-4.50102949	3.61224235
O	10.38029483	-3.77181623	2.72355451
C	11.07562944	-6.55114195	2.82684219
C	10.94883916	-7.97798204	2.31112746
O	10.56745831	-8.91001103	3.01542506
N	11.26734623	-8.10567849	0.99586393
H	8.83598654	-6.35221057	1.49589823
H	9.51071426	-6.51325963	4.33987177
H	11.82819813	-6.53186582	3.62953019
H	11.42300907	-5.88095235	2.03224019
H	11.28045136	-9.03686529	0.59386557
H	11.68288209	-7.32596725	0.48455018
N	9.61267465	-4.02669188	4.84201118
C	9.75200331	-2.59914514	5.11997512
H	9.00263541	-4.59975523	5.41825490
H	10.66396267	-2.28990009	4.60898302
H	9.85064222	-2.44490896	6.20330078
H	8.90502676	-2.02194600	4.74802596
C	11.66102040	-0.83998796	1.57601325
C	12.85577176	-1.60043550	1.03138808
O	13.79378807	-1.05327724	0.45090073
H	11.17399296	-1.45700338	2.33199200
H	10.95099413	-0.57901418	0.79100187
H	12.02650296	0.09306031	2.02805337
N	12.80346005	-2.94091510	1.27819380
C	13.89003483	-3.83325037	0.93336100
C	13.39602829	-4.84514055	-0.10253952
O	12.36274023	-5.50588454	0.09269239
C	14.44714301	-4.58459529	2.15748020
C	14.79039986	-3.65196154	3.33292209
C	13.63348414	-3.28051173	4.22743560
O	12.95918432	-4.39667606	4.64576357
N	13.39445261	-2.07369498	4.56488021
H	11.97587536	-3.33740787	1.72408013
H	14.69157241	-3.21375751	0.51198397
H	15.36113605	-5.10024051	1.82836479
H	13.73038147	-5.35039215	2.48687646
H	15.24746655	-2.71459515	2.98696080
H	15.52705529	-4.15878887	3.97951037
H	12.61595922	-2.02298882	5.23454588
N	14.14741627	-4.96464366	-1.21389916
C	13.90700935	-6.01802101	-2.19596468
H	15.04542705	-4.48570966	-1.21710180
H	13.08998947	-6.62899276	-1.81301392
H	13.66571808	-5.61374081	-3.19011334
H	14.81300414	-6.62199598	-2.23101441
O	16.81561284	2.91643179	10.80993870
H	17.06742004	3.23250819	9.92695968
H	16.15737392	2.22238658	10.61627364
N	20.76708969	0.72862986	5.61049404
C	21.05438547	-0.48325809	5.04238045
O	22.22798991	-0.82104634	4.81387670
N	20.03138853	-1.36764066	4.69790040
C	18.69770285	-1.20649379	5.00039245
O	17.84425435	-2.02280551	4.68639711
C	18.40712850	0.06793926	5.73029596
N	17.18084087	0.29439997	6.10389949
C	16.90916572	1.49050445	6.70588682
C	15.58412483	1.74104121	7.12817915
C	15.21626335	2.93700130	7.71949271
C	13.80168361	3.17599926	8.17751261
C	16.21570825	3.94444860	7.88935454
C	15.84834827	5.26474335	8.51573567
C	17.53454734	3.70331900	7.50047117
C	17.91231285	2.48067695	6.90524890
N	19.21040972	2.20484046	6.50117317
C	19.51955353	0.98796484	5.93902479
C	20.28063324	3.21228403	6.69474346
C	20.85323843	3.21113956	8.11815337
H	20.30071658	-2.22815361	4.18473663
H	14.85869415	0.94370308	6.95754685
H	13.76374403	3.38249762	9.26026488
H	13.35714069	4.04944574	7.67286167
H	13.16663651	2.30308422	7.97463833
H	16.70756167	5.94785666	8.55898797
H	15.04200565	5.75793780	7.94929857
H	15.47389948	5.11688529	9.54208198
H	18.27064813	4.48779989	7.66389682
H	19.85453760	4.18720149	6.42490513
H	21.06082847	2.97473075	5.96490955
H	21.49783003	2.34288383	8.25094664
H	21.45109410	4.12482615	8.26206603
H	20.05584748	3.19803259	8.86188786
H	12.20514315	-4.14124813	5.20396535

AsLOV2 **Qc** S_0

C	19.02907417	2.11199045	13.81299636
C	19.33683297	0.94137451	12.91208151
O	20.08916876	0.01870353	13.25594555
H	18.15299028	2.65196183	13.45296935
H	18.86871029	1.74993126	14.83780584
H	19.89298381	2.77602368	13.82103260
N	18.77256492	1.00226515	11.68094281
C	18.93053094	-0.06186312	10.69660963
C	20.26933933	0.06555621	9.91551797
O	20.29728158	0.10289758	8.68303299
C	17.76925856	-0.08839478	9.71026811
S	16.10027966	-0.36732301	10.44908279
H	18.11922724	1.76186249	11.48518649
H	18.98202592	-1.01462479	11.25236766
H	17.95890558	-0.84472596	8.94050005
H	17.68954997	0.87975844	9.20141502
H	16.46391852	-1.31497107	11.35261256
N	21.35275423	0.11553586	10.72204607
C	22.71300477	0.02611725	10.23604391
H	21.18148411	-0.03945800	11.71887238
H	22.71798195	0.02790570	9.14600059
H	23.15908225	-0.93470128	10.55229008
H	23.37803500	0.80597672	10.60798672
C	25.51301088	-3.72099440	7.23809350
C	25.84306566	-2.22503993	7.22163715
C	24.66588309	-1.40313405	6.69072642
C	24.68746918	0.06658809	7.10955201
O	25.30463717	0.46285675	8.10034708
N	23.94554405	0.88708209	6.32367622
H	25.30277452	-4.10471321	6.22392700
H	26.74582088	-2.02049304	6.61986170
H	26.06853142	-1.88098623	8.24368935
H	23.71659848	-1.80726423	7.09151887
H	24.57386334	-1.48064328	5.59573809
H	23.78036597	1.82897373	6.66118796
H	23.30298862	0.49814488	5.63020023
H	26.30802185	-4.33201006	7.66594913
H	24.60697619	-3.84099487	7.83196294
C	23.25603054	-6.51500602	3.78402139
C	21.83348774	-5.94439330	3.89629333
C	21.68338370	-4.42331591	3.83621287
O	20.57487024	-3.92273002	3.55714331
N	22.72850258	-3.63734894	4.14137516
H	23.91599109	-5.85798717	4.34999650
H	21.38716927	-6.22436436	4.86794907
H	21.15803351	-6.35194487	3.12907724
H	22.59285196	-2.61959194	4.20460557
H	23.62775935	-4.01889523	4.40058795
H	23.34098725	-7.52800987	4.17697766
H	23.60784578	-6.51123791	2.74035873
C	19.25003853	-5.26304797	-1.05004878
C	19.34527594	-4.76325389	0.39225638
C	18.04194286	-4.10567385	0.86429160
O	17.06907715	-3.94813362	0.11436348
N	18.04910352	-3.72925389	2.16132122
H	18.37498648	-5.91098210	-1.09597981
H	20.15949356	-4.03038633	0.52720607
H	19.57667679	-5.59011484	1.08686114
H	17.29978827	-3.15893515	2.53397301
H	18.87645310	-3.86123332	2.74164026
H	20.14884874	-5.82577240	-1.34285761
H	19.12099025	-4.41298333	-1.71997650
C	6.29798779	-6.61098190	1.89500958
C	7.35434829	-6.34791725	2.95847289
O	7.07554226	-6.17213266	4.14504291
H	6.67441014	-7.25572463	1.08660824
H	6.01599130	-5.65100097	1.46300462
H	5.42300464	-7.06902042	2.35498971
N	8.64332739	-6.28821600	2.50992536
C	9.74736052	-6.03288851	3.42830688
C	9.92911513	-4.51692483	3.62441649
O	10.39580565	-3.81350630	2.72264731
C	11.06912750	-6.58804952	2.89237099
C	10.96167599	-8.03279380	2.42269350
O	10.51437866	-8.93093089	3.13287761
N	11.39200404	-8.22004878	1.14836913
H	8.84150586	-6.34409760	1.51724881
H	9.48065498	-6.51774224	4.37641226
H	11.80710729	-6.55051551	3.70909557
H	11.43707793	-5.93987889	2.08860567
H	11.41824869	-9.16581605	0.78323046
H	11.83339184	-7.46024201	0.62962755
N	9.59309308	-4.02484183	4.83875909
C	9.75198696	-2.59891278	5.11998991
H	8.97673287	-4.59264516	5.41277368
H	10.66403332	-2.29001783	4.60909712
H	9.85241919	-2.44809957	6.20349594
H	8.90498263	-2.02206715	4.74793679
C	11.66099547	-0.83999232	1.57599959
C	12.86043058	-1.59434846	1.03744861
O	13.82648477	-1.03632469	0.51472493
H	11.17400355	-1.45700682	2.33199671
H	10.95100058	-0.57899878	0.79100144
H	12.02332605	0.09136240	2.03269014
N	12.78037157	-2.94733294	1.20511100
C	13.90470309	-3.81825391	0.92390646
C	13.45401521	-4.89619913	-0.06239478
O	12.50340475	-5.64868132	0.20665537
C	14.47848840	-4.47889173	2.19218229
C	14.73967970	-3.46437954	3.32665432
C	13.51346193	-3.16218118	4.18229762
O	12.98027833	-2.05636556	4.23215508
N	13.04052463	-4.22485143	4.89641118
H	11.97004229	-3.34228530	1.68392682
H	14.68799385	-3.19233102	0.47866894
H	15.42418222	-4.96314679	1.90634902
H	13.79216964	-5.26984164	2.53260847
H	15.08952868	-2.50433087	2.92248508
H	15.52980897	-3.85160690	3.99100788
H	12.20527841	-4.11677673	5.45968800
H	13.52426963	-5.11263750	4.92088152
N	14.13481778	-4.95471639	-1.22330032
C	13.90699099	-6.01799608	-2.19603123
H	14.99164682	-4.40963350	-1.28245734
H	13.09000895	-6.62900747	-1.81299451
H	13.66781436	-5.61779664	-3.19207902
H	14.81299996	-6.62200270	-2.23099316
O	16.32074236	2.68720171	11.56193656
H	15.88893103	2.97735643	12.38050905
H	15.98878974	1.77355189	11.43001638
N	20.73329744	0.74116748	5.50937250
C	21.00508060	-0.45461221	4.90283485
O	22.16928431	-0.77184146	4.59539024
N	19.97747871	-1.35091718	4.60810285
C	18.65315581	-1.19719615	4.95276266
O	17.79629317	-2.02046646	4.66095403
C	18.38240973	0.06085473	5.71070825
N	17.17147703	0.26033455	6.15216871
C	16.92712197	1.41232428	6.83823220
C	15.64945160	1.59245590	7.41974418
C	15.34613281	2.70271530	8.18634749
C	14.01721120	2.83349769	8.88138561
C	16.34061265	3.71958599	8.33205152
C	16.04577943	4.94480587	9.15173364
C	17.59606054	3.56504671	7.75136210
C	17.93264288	2.40332615	7.02841824
N	19.20347771	2.17265623	6.52085439
C	19.49690866	0.98001895	5.90236292
C	20.26343429	3.19558613	6.69966994
C	20.85330071	3.21119744	8.11817708
H	20.23211658	-2.23394223	4.12723364
H	14.92308629	0.79310819	7.26343464
H	14.17119236	2.91052179	9.97012673
H	13.47795122	3.74124968	8.56451020
H	13.37523474	1.96493783	8.68016797
H	16.86387841	5.67643582	9.09699819
H	15.11649492	5.43665275	8.82274563
H	15.91108562	4.64683499	10.20360201
H	18.32755485	4.35614451	7.89755117
H	19.81507627	4.16306921	6.43472491
H	21.03628200	2.96740315	5.95935642
H	21.49782997	2.34286459	8.25094908
H	21.44800206	4.12850994	8.24904340
H	20.05581414	3.19797772	8.86185124

AsLOV2 **Qd** S_0

C	19.02908036	2.11203215	13.81284798
C	19.34352002	0.94059336	12.90990014
O	20.11414238	0.03321346	13.25402214
H	18.15296731	2.65196019	13.45302295
H	18.86704105	1.74978465	14.83723988
H	19.89299842	2.77600320	13.82110298
N	18.77146709	0.99352560	11.68365437
C	18.92605379	-0.05927671	10.68349429
C	20.26906109	0.06625910	9.91190311
O	20.30452120	0.09817648	8.67862230
C	17.75971876	-0.03126516	9.69959508
S	16.10936849	-0.40214725	10.42879048
H	18.10173793	1.74033077	11.49325332
H	18.95162453	-1.02378332	11.21870876
H	17.92649046	-0.77882974	8.91487766
H	17.70923938	0.94461455	9.20210565
H	15.88224017	0.83948536	10.92515107
N	21.35214912	0.11965781	10.72023945
C	22.71305663	0.02594300	10.23613371
H	21.17825512	-0.03733054	11.71684452
H	22.71796962	0.02799300	9.14600064
H	23.15782995	-0.93520056	10.55324789
H	23.37799970	0.80604306	10.60791275
C	25.51300183	-3.72099727	7.23805154
C	25.84662480	-2.22561482	7.22725945
C	24.67081184	-1.39794154	6.70339609
C	24.69990463	0.07084538	7.12452365
O	25.33490696	0.46713066	8.10375213
N	23.94329780	0.89205757	6.35276705
H	25.30370191	-4.10214701	6.22285679
H	26.74870331	-2.02067746	6.62455138
H	26.07576336	-1.88733485	8.25044440
H	23.72121729	-1.79938019	7.10590434
H	24.57439808	-1.47188300	5.60852810
H	23.78444138	1.83378608	6.69350402
H	23.28957307	0.50288514	5.67033923
H	26.30801492	-4.33200331	7.66597056
H	24.60698878	-3.84099828	7.83198056
C	23.25601265	-6.51500348	3.78398227
C	21.83240721	-5.94678513	3.90022646
C	21.68013090	-4.42561094	3.85993116
O	20.57019591	-3.92266694	3.59006309
N	22.72469874	-3.64210649	4.17259752
H	23.91599670	-5.85800442	4.35001031
H	21.38418815	-6.23914577	4.86729075
H	21.15842278	-6.34573857	3.12723255
H	22.58657263	-2.62574755	4.24993737
H	23.62368702	-4.02583305	4.42924900
H	23.34100270	-7.52799955	4.17700330
H	23.60624782	-6.51022994	2.73985558
C	19.24998440	-5.26299838	-1.04999107
C	19.35064317	-4.76217462	0.39087515
C	18.05782357	-4.08167063	0.85853859
O	17.07980469	-3.93148030	0.11284074
N	18.08394974	-3.67050333	2.14319952
H	18.37500834	-5.91100941	-1.09600808
H	20.17773388	-4.04386304	0.52549623
H	19.56499367	-5.59203057	1.08747805
H	17.32259724	-3.12471726	2.52674895
H	18.90018660	-3.83071978	2.73206120
H	20.14747335	-5.82708597	-1.34453118
H	19.12100438	-4.41299732	-1.71999713
C	6.29799747	-6.61099846	1.89500214
C	7.36157003	-6.34204719	2.95090117
O	7.09403129	-6.17397468	4.14130791
H	6.67210854	-7.25580993	1.08547772
H	6.01600146	-5.65100039	1.46299911
H	5.42300141	-7.06900253	2.35499917

N	8.64248865	-6.26006917	2.48582181
C	9.76290378	-6.00259914	3.38497404
C	9.92400751	-4.48949981	3.60493835
O	10.36936472	-3.75415764	2.71641513
C	11.07890452	-6.51719184	2.80514216
C	11.01541003	-7.97298693	2.37143299
O	10.57405726	-8.86864858	3.08915640
N	11.49037550	-8.18044928	1.11517015
H	8.82866622	-6.32723938	1.49157764
H	9.53040600	-6.50811196	4.33153555
H	11.85636004	-6.40947305	3.57860363
H	11.37788506	-5.87479146	1.96890790
H	11.55107779	-9.13154971	0.76928342
H	11.88235873	-7.41068122	0.57350633
N	9.59543151	-4.01883120	4.82894058
C	9.75200072	-2.59900519	5.11999637
H	9.01717694	-4.60469238	5.42306147
H	10.66400077	-2.28999917	4.60900033
H	9.84858202	-2.45218021	6.20456313
H	8.90500163	-2.02199814	4.74800234
C	11.66100082	-0.84000906	1.57600611
C	12.85742899	-1.60063569	1.03388745
O	13.78881137	-1.04986456	0.44563700
H	11.17399582	-1.45699910	2.33199717
H	10.95100381	-0.57899504	0.79100005
H	12.02521193	0.09555038	2.02459321
N	12.82035059	-2.93976986	1.29598254
C	13.90589258	-3.82343039	0.91997566
C	13.40443125	-4.83881229	-0.10714509
O	12.37987689	-5.50954972	0.09635394
C	14.50845819	-4.56835828	2.12768078
C	14.81548270	-3.61974099	3.29784419
C	13.64617508	-3.41367289	4.23501894
O	13.42530629	-2.08906161	4.48358569
N	13.00576287	-4.40034852	4.72771697
H	11.99386765	-3.34470601	1.73907395
H	14.68858849	-3.19250303	0.48037941
H	15.43651348	-5.04577130	1.78177934
H	13.82503858	-5.35909019	2.46930388
H	15.15327877	-2.63790248	2.93529485
H	15.63315855	-4.03706341	3.90682971
H	12.24271357	-4.10093275	5.34641533
N	14.14825015	-4.95709066	-1.22409947
C	13.90699999	-6.01800243	-2.19600216
H	15.04227945	-4.47088181	-1.23889844
H	13.08999995	-6.62900049	-1.81300313
H	13.66519509	-5.62066255	-3.19305425
H	14.81300107	-6.62200148	-2.23099716
O	16.67539135	3.04875732	11.34010742
H	17.06780538	3.90753814	11.11860811
H	16.11150827	3.24237895	12.10579901
N	20.72687439	0.73700495	5.54257076
C	20.99560674	-0.46716195	4.94998649
O	22.15948299	-0.78891921	4.64866337
N	19.96579760	-1.36207028	4.66071877
C	18.63846595	-1.19252439	4.98629385
O	17.77538802	-2.00506138	4.68509544
C	18.37026972	0.07558797	5.73101432
N	17.15638823	0.29389053	6.15176749
C	16.91059942	1.46629516	6.80489694
C	15.61764308	1.68164860	7.33315665
C	15.29208910	2.83541364	8.02160953
C	13.93182941	3.01360946	8.64113930
C	16.29092926	3.84554829	8.16072383
C	15.95246601	5.14116355	8.84960756
C	17.57404703	3.64561682	7.65739964
C	17.92061844	2.45302344	6.99020656
N	19.19970087	2.19410965	6.51790062
C	19.49016636	0.98911284	5.92166667
C	20.27055204	3.20325614	6.69784941
C	20.85328648	3.21117323	8.11822158
H	20.21918444	-2.24810505	4.18347124
H	14.89152135	0.88034331	7.19049390
H	14.01906010	3.07291708	9.73961331
H	13.44416045	3.94293376	8.30515545
H	13.27088200	2.17026310	8.39899811
H	16.83523087	5.78602542	8.96565915
H	15.19645000	5.70235757	8.27462330
H	15.51359513	4.96001698	9.84309419
H	18.31206122	4.43160338	7.80167066
H	19.83859333	4.17642238	6.42783667
H	21.04476471	2.96300978	5.96273791
H	21.49784855	2.34287417	8.25091860
H	21.45040802	4.12642073	8.25479561
H	20.05581043	3.19799523	8.86184764
H	12.74231441	-2.00466177	5.16972200

AsLOV2 **Qa** S_1

C	19.02910984	2.11195499	13.81255656
C	19.30726540	0.92719039	12.90530767
O	19.96967191	-0.05207966	13.27594501
H	18.15298208	2.65200932	13.45306090
H	18.86501478	1.74870762	14.83600811
H	19.89299736	2.77600121	13.82123623
N	18.79255237	1.03538025	11.65575138
C	18.92592652	-0.00099418	10.63644911
C	20.28317842	0.06661210	9.89177426
O	20.35006775	0.03627261	8.66376379
C	17.77659899	0.09962922	9.63500296
S	16.12466314	-0.28734863	10.34756643
H	18.41892344	1.94075554	11.34769593
H	18.89495362	-0.97232991	11.15752343
H	17.92782769	-0.61400381	8.81600957
H	17.75429618	1.09669674	9.17632546
H	16.18067158	0.61742920	11.35894459
N	21.35447062	0.14712451	10.71952723
C	22.71323105	0.02576796	10.23641719
H	21.17458078	-0.01253825	11.71205002
H	22.71791914	0.02788612	9.14599969
H	23.14951129	-0.93844560	10.55699129
H	23.37786931	0.80626266	10.60768599
C	25.51286027	-3.72107483	7.23792978
C	25.83874112	-2.22408782	7.21756333
C	24.66554145	-1.41539033	6.66074964
C	24.63884599	0.05076202	7.09425889
O	25.31656525	0.48001126	8.03188737
N	23.78411340	0.82185712	6.37829329
H	25.30245421	-4.10606744	6.22406300
H	26.75154012	-2.02024609	6.63074603
H	26.04717573	-1.87311266	8.24095382
H	23.70764966	-1.84401031	7.00976490
H	24.61354698	-1.47371276	5.56148979
H	23.57836785	1.74587985	6.74175138
H	23.10167938	0.38127011	5.75041581
H	26.30805694	-4.33192742	7.66600088
H	24.60705440	-3.84101268	7.83207978
C	23.25606859	-6.51493390	3.78395429
C	21.82048400	-5.98574543	3.93109440
C	21.59904195	-4.47294381	3.97057964
O	20.44098591	-4.03673269	3.80457517
N	22.61848753	-3.64734774	4.24300616
H	23.91596410	-5.85800215	4.35004673
H	21.38568096	-6.34027995	4.88326457
H	21.15464535	-6.37186559	3.14391021
H	22.42853565	-2.63827237	4.39130323
H	23.54931650	-3.99905224	4.42159755
H	23.34092401	-7.52801823	4.17697326
H	23.59263632	-6.50305577	2.73580173
C	19.24993662	-5.26289940	-1.04995596
C	19.38860095	-4.75464097	0.38336303
C	18.17015838	-3.94677985	0.84975881
O	17.11153802	-3.90456446	0.20434757
N	18.37497437	-3.29040310	2.00900025
H	18.37508306	-5.91110879	-1.09602218
H	20.28567402	-4.12467147	0.50508237
H	19.51583188	-5.59354698	1.09200038
H	17.62793431	-2.78158825	2.46702639
H	19.18363864	-3.52210245	2.58738959
H	20.13907626	-5.83377599	-1.35784515
H	19.12096480	-4.41298140	-1.71996859
C	6.29796869	-6.61097264	1.89507997
C	7.37753832	-6.45621083	2.94905932
O	7.15169092	-6.52980521	4.15185117
H	6.65032929	-7.24963428	1.06960620
H	6.01603342	-5.65102320	1.46292939
H	5.42299464	-7.06897925	2.35500993
N	8.65786982	-6.27224982	2.46439243
C	9.74836093	-6.00949850	3.39534532
C	9.90887309	-4.49489656	3.60416922
O	10.33063170	-3.79788743	2.67139391
C	11.09731650	-6.49894179	2.85769666
C	11.07099846	-7.89103647	2.25666122
O	10.54829590	-8.81449930	2.92861171
N	11.60979165	-8.07739466	1.07240304
H	8.76984605	-5.93134606	1.51349093
H	9.48293060	-6.51745125	4.32957290
H	11.83616812	-6.51027973	3.67309401
H	11.46207282	-5.82456952	2.07070878
H	11.63425033	-9.00851367	0.66821621
H	12.03389276	-7.26663558	0.58617416
N	9.58042193	-4.01473884	4.81181366
C	9.75202713	-2.59899506	5.11992649
H	9.10644194	-4.62750559	5.46620101
H	10.66402612	-2.29001398	4.60903811
H	9.85287323	-2.46752721	6.20440967
H	8.90498517	-2.02201507	4.74801168
C	11.66103802	-0.84007116	1.57597691
C	12.84152107	-1.58554985	0.99299356
O	13.74897829	-1.03871241	0.36633599
H	11.17403011	-1.45696562	2.33204685
H	10.95099831	-0.57900348	0.79100122
H	12.02882346	0.09476531	2.02198555
N	12.82087840	-2.93448959	1.22803245
C	13.95293802	-3.78455065	0.91409057
C	13.49306267	-4.88382519	-0.04323978
O	12.54947418	-5.64190526	0.26551143
C	14.58167330	-4.38850159	2.18547947
C	15.16643549	-3.32365166	3.11665276
C	14.18624836	-2.67043392	4.08990537
O	12.97239486	-2.94025152	4.08357423
N	14.74225209	-1.79092866	4.93675967
H	12.08346254	-3.31332179	1.82640799
H	14.70717462	-3.15636564	0.42413614
H	15.39659251	-5.04781445	1.85226690
H	13.83174717	-4.99528183	2.71569519
H	15.61834238	-2.51257799	2.52361096
H	15.98601759	-3.74430656	3.71821864
H	14.14400789	-1.32629679	5.61002053
H	15.76120145	-1.62647873	4.96815033
N	14.14395688	-4.95918459	-1.21295251
C	13.90705100	-6.01797058	-2.19607571
H	14.97852571	-4.38498151	-1.31009134
H	13.09000636	-6.62896360	-1.81293149
H	13.66797824	-5.60405866	-3.18511834
H	14.81297213	-6.62204371	-2.23099707
O	17.79446343	3.52907759	10.67493892
H	18.46356494	4.22278121	10.56806718
H	17.39867420	3.46349464	9.78483940
N	20.62747771	0.69813979	5.61384950
C	20.86052176	-0.52093843	5.07874088
O	22.02903027	-0.91417100	4.81264465
N	19.79761030	-1.36840838	4.79970580
C	18.46615822	-1.08286617	5.05066504
O	17.57959556	-1.89035328	4.69142857
C	18.23350269	0.17692332	5.72811062
N	16.96411008	0.48041196	6.10564223
C	16.79514077	1.68607058	6.71125437
C	15.50692734	2.04739729	7.17730564
C	15.23382854	3.26003947	7.80067417
C	13.84438318	3.57771611	8.29569853
C	16.28677506	4.19276601	7.96786433
C	16.03767207	5.52998951	8.62453599
C	17.57868416	3.85075130	7.52869600
C	17.86145161	2.61803388	6.91995484
N	19.15565088	2.25883967	6.51081753
C	19.37219228	1.01252254	5.94172630
C	20.27204348	3.19949235	6.69289779
C	20.85339533	3.21125439	8.11835287
H	20.01642039	-2.29050690	4.39811440
H	14.71489627	1.31049117	7.03249608
H	13.84000535	3.77277596	9.38170439
H	13.43834966	4.48274754	7.81244344
H	13.15093979	2.74766622	8.09919369
H	16.94906949	6.14593156	8.64126857
H	15.25642965	6.10181676	8.09644361
H	15.69472213	5.41171491	9.66662511
H	18.37527547	4.57862886	7.67716637
H	19.90989965	4.19677011	6.40286024
H	21.04553969	2.90789969	5.97521353
H	21.49778706	2.34281138	8.25080355
H	21.45466001	4.12367665	8.26303178
H	20.05570301	3.19801947	8.86184119

AsLOV2 **Qb** S_1

C	19.02898284	2.11202276	13.81256011
C	19.33166714	0.93318480	12.90988442
O	20.06741582	0.00127177	13.26577882
H	18.15301478	2.65204889	13.45304671
H	18.86571192	1.74908491	14.83648035
H	19.89304832	2.77593816	13.82130280
N	18.77486689	0.99402037	11.67486473
C	18.93092301	-0.05431545	10.66719764
C	20.27983540	0.04051076	9.90577354
O	20.33028324	0.00307057	8.67714282
C	17.77522429	-0.00484588	9.67038291
S	16.12763003	-0.39809007	10.42194716
H	18.20314125	1.80606169	11.41309959
H	18.93859084	-1.02146202	11.19918217
H	17.94369972	-0.73341038	8.86938710
H	17.72156403	0.98390788	9.19846449
H	16.45199981	-0.05866013	11.69694704
N	21.35549352	0.14060138	10.72241458
C	22.71296292	0.02623452	10.23607471
H	21.18257485	-0.00062815	11.72022898
H	22.71802222	0.02780807	9.14600117
H	23.15280171	-0.93690526	10.55592473
H	23.37806082	0.80597147	10.60795825
C	25.51293081	-3.72098930	7.23802889
C	25.86317953	-2.22807732	7.26509520
C	24.69525570	-1.37298428	6.77599365
C	24.72843189	0.07853436	7.25489356
O	25.43202167	0.45287148	8.19624728
N	23.88866363	0.90149014	6.58176893
H	25.31863968	-4.07978770	6.21176365
H	26.76413729	-2.01949500	6.66217646
H	26.10657788	-1.92470436	8.29608324
H	23.74118209	-1.78203730	7.15967351
H	24.59461408	-1.39895962	5.68014218
H	23.73118376	1.82758830	6.96267659
H	23.21378071	0.51853429	5.91426422
H	26.30804154	-4.33198213	7.66595723
H	24.60701900	-3.84101820	7.83202093
C	23.25598656	-6.51502296	3.78399241
C	21.83901298	-5.93811186	3.99965346
C	21.77895687	-4.41291501	3.94791377
O	20.98912823	-3.82366299	3.17140965
N	22.57494285	-3.70382318	4.75402413
H	23.91601559	-5.85798617	4.34997004
H	21.47416951	-6.23361137	4.99937983
H	21.11561858	-6.31788649	3.26478923
H	22.51893278	-2.66652060	4.74776002
H	23.16977020	-4.15700573	5.43714510
H	23.34100614	-7.52799342	4.17701802
H	23.55709355	-6.48874386	2.72681523
C	19.24995609	-5.26295697	-1.04991218
C	19.50855374	-4.80133059	0.38227257
C	18.44045201	-3.92134086	1.04830905
O	17.52936468	-3.48241025	0.28311594
N	18.51625638	-3.65626006	2.31426962
H	18.37502400	-5.91102470	-1.09605627
H	20.42112853	-4.19129771	0.46653672
H	19.62458109	-5.65036728	1.07085898
H	18.00547826	-2.90035696	2.82655079
H	19.40768240	-3.94868909	2.76549253
H	20.12069990	-5.83901961	-1.39072109
H	19.12100681	-4.41300859	-1.72001020
C	6.29797213	-6.61098724	1.89505700
C	7.37278753	-6.33678865	2.94175861
O	7.12668116	-6.19654403	4.13987634
H	6.66944757	-7.25648643	1.08473729
H	6.01602178	-5.65100441	1.46297935
H	5.42299744	-7.06901253	2.35498192
N	8.64198254	-6.20718198	2.45261744
C	9.78681757	-5.98529574	3.33035117
C	9.98942435	-4.48184029	3.58847364
O	10.52997097	-3.75607665	2.74403203
C	11.07275427	-6.51235924	2.70669347
C	11.06872114	-8.01539625	2.45834065
O	10.39972145	-8.79859218	3.12953380
N	11.88193401	-8.39890333	1.44123910
H	8.80991281	-6.28602531	1.45626060
H	9.57247136	-6.51197453	4.26974008
H	11.90976572	-6.28604648	3.38940575
H	11.29986497	-5.96016707	1.78654670
H	12.01713938	-9.39089069	1.28292415
H	12.47069858	-7.71935418	0.96190114
N	9.59439036	-4.01721761	4.79522228
C	9.75193531	-2.59902337	5.11996000
H	8.95961997	-4.59947728	5.33476475
H	10.66401707	-2.29003555	4.60899110
H	9.84606219	-2.47116541	6.20640859
H	8.90503645	-2.02191189	4.74805886
C	11.66102103	-0.84007152	1.57602569
C	12.85136255	-1.57667700	0.97816074
O	13.75263639	-0.98857089	0.38017183
H	11.17397958	-1.45698165	2.33200085
H	10.95100905	-0.57899465	0.79099565
H	12.03939303	0.08479907	2.03029389
N	12.85342385	-2.93988131	1.15259858
C	14.05796928	-3.71553405	0.88051371
C	13.74072990	-4.95084831	0.01867916
O	13.22151616	-5.95863235	0.51709215
C	14.75682891	-4.17613995	2.17507153
C	14.97567635	-3.04936332	3.19830815
C	13.77957294	-2.74248732	4.06670050
O	13.23314631	-3.89198055	4.57460363
N	13.40331940	-1.54785604	4.30290839
H	12.11706535	-3.36380751	1.72079291
H	14.71671325	-3.03208587	0.32682617
H	15.72656686	-4.62098079	1.89976373
H	14.16788120	-4.98657404	2.62677599
H	15.29404605	-2.10991525	2.72746941
H	15.78282295	-3.33831047	3.89127511
H	12.62300145	-1.52385220	4.97194018
N	14.07919231	-4.88623969	-1.28751812
C	13.90703052	-6.01801609	-2.19599152
H	14.51751871	-4.04077082	-1.63369757
H	13.09001149	-6.62900975	-1.81299548
H	13.66655666	-5.65554930	-3.20457819
H	14.81301505	-6.62197968	-2.23104953
O	16.79873367	2.88945082	10.79098013
H	16.98827973	3.20464454	9.88856041
H	16.13817383	2.18973721	10.63012300
N	20.80385862	0.72610716	5.55600937

C 21.09429755 -0.43327663 4.93119427
O 22.27754243 -0.83600214 4.76777609
N 20.06295306 -1.22467702 4.43366799
C 18.71076633 -1.01059336 4.65730425
O 17.85403196 -1.76678626 4.14189312
C 18.42051637 0.13460047 5.49849664
N 17.13497920 0.34834454 5.88073687
C 16.90246427 1.49208954 6.57414899
C 15.58580439 1.76095093 7.02712789
C 15.23562859 2.93211037 7.68933046
C 13.81949794 3.16060555 8.15667705
C 16.23405445 3.91320559 7.91838398
C 15.88585371 5.22490186 8.58290244
C 17.55611745 3.65045977 7.51757249
C 17.91555173 2.46070386 6.86452590
N 19.23419449 2.18458378 6.46724323
C 19.51962513 0.98697989 5.82942875
C 20.28896535 3.18766992 6.68785463
C 20.85307592 3.21093468 8.11827915
H 20.34606331 -2.04797430 3.89651084
H 14.83728163 0.99709471 6.80639102
H 13.77790206 3.30024070 9.25083277
H 13.38444417 4.06959229 7.70793966
H 13.17219107 2.31024805 7.89945373
H 16.76962072 5.87224305 8.68188790
H 15.12563450 5.77834965 8.00598536
H 15.46886267 5.07027793 9.59224100
H 18.30866750 4.40997862 7.72412148
H 19.86823684 4.16589683 6.41055580
H 21.08999921 2.95989655 5.97697437
H 21.49800280 2.34299822 8.25086293
H 21.45071097 4.12580186 8.26097889
H 20.05587293 3.19804468 8.86191443
H 12.47283942 -3.67053706 5.13642074

AsLOV2 **Qc** S_1

C 19.02908216 2.11200251 13.81297524
C 19.33212530 0.95054655 12.89659366
O 20.06945882 0.01283509 13.23126948
H 18.15298562 2.65195375 13.45297258
H 18.86986156 1.74089941 14.83433083
H 19.89298778 2.77602041 13.82104795
N 18.78062339 1.03838687 11.65987563
C 18.92685120 -0.01279785 10.65962135
C 20.27702616 0.09865453 9.89548988
O 20.31994656 0.14617497 8.66260278
C 17.77784933 0.00359142 9.65872577
S 16.09649250 -0.26730487 10.37179026
H 18.13823216 1.80925454 11.47060697
H 18.95396879 -0.97618459 11.19859976
H 17.96235201 -0.73324248 8.86933815
H 17.72110878 0.98694233 9.17564928
H 16.43876662 -1.23095062 11.26680870
N 21.35085925 0.12337915 10.71297348
C 22.71299149 0.02613373 10.23604026
H 21.16741345 -0.03840892 11.70701088
H 22.71799005 0.02790232 9.14600127
H 23.15338089 -0.93537345 10.55664224
H 23.37804437 0.80596862 10.60799085
C 25.51300654 -3.72097468 7.23813700
C 25.85986624 -2.22697909 7.26258197
C 24.68960621 -1.37193061 6.77387451
C 24.73242390 0.08190867 7.24506250
O 25.42164387 0.45367321 8.19596793
N 23.91585302 0.91835574 6.55537193
H 25.31988251 -4.08209812 6.21269563
H 26.75878371 -2.01554920 6.65800311
H 26.10319284 -1.92151427 8.29286473
H 23.73721046 -1.77821496 7.16506614
H 24.58914281 -1.40493319 5.67762700
H 23.80287649 1.86401494 6.90177686
H 23.26598997 0.55582564 5.85735068
H 26.30803180 -4.33202073 7.66591992
H 24.60697481 -3.84100325 7.83195706
C 23.25594873 -6.51500597 3.78413193
C 21.82271962 -5.96001339 3.94020541
C 21.69222377 -4.43618748 3.97728298
O 20.73747371 -3.85741725 3.42204743
N 22.61151355 -3.73431956 4.66490229
H 23.91603422 -5.85797172 4.34993006
H 21.39236130 -6.31096274 4.89616869
H 21.14903237 -6.31403429 3.14719483
H 22.53242071 -2.71176480 4.71819684
H 23.33506898 -4.19075398 5.20428029
H 23.34101810 -7.52802453 4.17693524
H 23.58523467 -6.49729483 2.73447143
C 19.25009479 -5.26310902 -1.05009573
C 19.36206716 -4.77161409 0.39238625
C 18.07063257 -4.10509173 0.88273792
O 17.08440334 -3.94973738 0.15020569
N 18.10385707 -3.72537898 2.17882878
H 18.37497188 -5.91096110 -1.09597861
H 20.18651192 -4.05055515 0.52664642
H 19.59120493 -5.60547241 1.07926985
H 17.37510131 -3.13672829 2.56546297
H 18.95672913 -3.83382890 2.72540003
H 20.14504036 -5.82780264 -1.35205808
H 19.12096755 -4.41296423 -1.71994735
C 6.29799985 -6.61098539 1.89499590
C 7.35496823 -6.34942572 2.95867399
O 7.07575981 -6.17362187 4.14519270
H 6.67383540 -7.25573744 1.08628925
H 6.01598352 -5.65100071 1.46301161
H 5.42300559 -7.06901740 2.35499449
N 8.64421122 -6.29235740 2.51071809
C 9.74820959 -6.03181158 3.42801365
C 9.92608638 -4.51539605 3.62312901
O 10.38898783 -3.81101283 2.71998672
C 11.07130395 -6.57925433 2.89085134
C 10.98645556 -8.03478319 2.45184124
O 10.51653394 -8.91871256 3.16565690
N 11.47258768 -8.25103734 1.20278869
H 8.84128567 -6.34211244 1.51750605
H 9.48521691 -6.51792454 4.37647803
H 11.81695195 -6.51196146 3.69918696
H 11.42564368 -5.94142250 2.07281519
H 11.51520899 -9.20419521 0.85943559
H 11.91101151 -7.49621082 0.67472085
N 9.59253289 -4.02437845 4.83833639
C 9.75198897 -2.59891364 5.11998567
H 8.97953957 -4.59353561 5.41445197
H 10.66403422 -2.29001623 4.60909873
H 9.85242263 -2.44825633 6.20352928
H 8.90498258 -2.02206628 4.74794030
C 11.66099579 -0.83999606 1.57599544
C 12.85935022 -1.59410335 1.03320702
O 13.82117892 -1.03469636 0.50442460
H 11.17400461 -1.45700599 2.33199781
H 10.95099947 -0.57899545 0.79100435
H 12.02496417 0.09067920 2.03256633
N 12.78328025 -2.94777693 1.20158758
C 13.91258498 -3.81449408 0.92310807
C 13.46711150 -4.90148494 -0.05615970
O 12.53226862 -5.66920430 0.22477716
C 14.49429201 -4.46382387 2.19361754
C 14.74974910 -3.44124253 3.32213955
C 13.52080982 -3.14511773 4.17578196
O 12.97812193 -2.04394971 4.22205267
N 13.04973073 -4.21159815 4.88708708
H 11.97737584 -3.34311004 1.68757230
H 14.69082093 -3.18598961 0.47262925
H 15.44345396 -4.94058368 1.90715993
H 13.81481313 -5.25848878 2.53924271
H 15.09440907 -2.48154710 2.91334025
H 15.54358842 -3.81809714 3.98799029
H 12.22880806 -4.09739826 5.46987053
H 13.55832674 -5.08408353 4.94218437
N 14.13332947 -4.95159355 -1.22618980
C 13.90698903 -6.01800984 -2.19602018
H 14.98145465 -4.39490418 -1.29738561
H 13.09000588 -6.62900393 -1.81299685
H 13.66800195 -5.61930543 -3.19269383
H 14.81300716 -6.62199376 -2.23099760
O 16.35233806 2.76131502 11.53241403
H 15.91759905 3.05475188 12.34828215
H 16.00810196 1.85313912 11.39328952
N 20.68924740 0.74233854 5.53838973
C 21.00048596 -0.49683308 4.99637056
O 22.19498015 -0.80677255 4.82809324
N 19.98828842 -1.34272386 4.63531336
C 18.63326695 -1.16651621 4.96419230
O 17.80290349 -2.01290906 4.63104784
C 18.35022448 0.05345702 5.71160362
N 17.08435297 0.27452966 6.14245640
C 16.88208121 1.44019496 6.80762940
C 15.62899557 1.67448045 7.40238875
C 15.35040846 2.81629297 8.16778855
C 14.03131976 2.96035779 8.86435081
C 16.35044350 3.82317512 8.31197464
C 16.08700514 5.05691608 9.12918550
C 17.59713694 3.61809256 7.72442057
C 17.90525145 2.44174099 7.00547719
N 19.18073960 2.18482693 6.52417492
C 19.43043850 0.96376287 5.92292950
C 20.26030310 3.18418654 6.69976466
C 20.85330526 3.21119706 8.11821047
H 20.24570246 -2.21561178 4.14123071
H 14.87477813 0.89642067 7.27467129
H 14.19997850 3.03059896 9.95301589
H 13.51485959 3.88826353 8.56555035
H 13.36758745 2.10932462 8.66231025
H 16.92181104 5.76894283 9.05997240
H 15.16855654 5.57447893 8.80734609
H 15.95858055 4.77615641 10.18730372
H 18.35336076 4.38959058 7.85315908
H 19.82986545 4.15704004 6.42318187
H 21.03295972 2.93747956 5.96574502
H 21.49782151 2.34285091 8.25092775
H 21.44530802 4.13110161 8.24230370
H 20.05581768 3.19799935 8.86185452

AsLOV2 **Qd** S_1

C 19.02909407 2.11209222 13.81275336
C 19.32800047 0.94115790 12.89765868
O 20.06225915 0.00567130 13.24551525
H 18.15294085 2.65193383 13.45305163
H 18.86459985 1.74240878 14.83356279
H 19.89301964 2.77597694 13.82116141
N 18.77592858 1.01339455 11.66036718
C 18.93484786 -0.03935559 10.65992695
C 20.28450322 0.07372448 9.90312591
O 20.33804908 0.10736306 8.67065426
C 17.79662147 -0.01799661 9.63970142
S 16.12696676 -0.33160780 10.34082905
H 18.19291855 1.81593763 11.41479094
H 18.94705101 -1.00175812 11.19858589
H 17.97160107 -0.79131597 8.88201353
H 17.77328359 0.94133187 9.11097239
H 15.91343300 0.95120826 10.74186381
N 21.35633782 0.12027537 10.72237019
C 22.71302880 0.02603958 10.23610710
H 21.17704320 -0.04162820 11.71685490
H 22.71796766 0.02794182 9.14600122
H 23.15916612 -0.93432000 10.55404509
H 23.37802838 0.80600545 10.60794418
C 25.51299976 -3.72101147 7.23798836
C 25.85988286 -2.22670032 7.26684439
C 24.68417156 -1.37193191 6.79231099
C 24.72948490 0.08232914 7.26049024
O 25.46659722 0.46985526 8.16803594
N 23.85917412 0.90312546 6.61834762
H 25.32023134 -4.07949512 6.21178217
H 26.75367710 -2.01151277 6.65613802
H 26.11255282 -1.92660714 8.29649753
H 23.73509290 -1.77886040 7.18997176
H 24.57141352 -1.40346744 5.69705612
H 23.75282590 1.84840504 6.96748152
H 23.17648943 0.52622195 5.96032931
H 26.30800502 -4.33199469 7.66600575
H 24.60700524 -3.84099290 7.83200463
C 23.25601673 -6.51497983 3.78380523
C 21.81587878 -5.97516786 3.94226351
C 21.65953797 -4.45792331 4.05586203
O 20.68087243 -3.87310792 3.54983112
N 22.57622087 -3.77103743 4.75989228
H 23.91599082 -5.85805343 4.35007579
H 21.37107652 -6.37607202 4.87165050
H 21.15801277 -6.29808579 3.12297033
H 22.46860860 -2.75441312 4.86667422
H 23.31712728 -4.23752661 5.26590896
H 23.34100762 -7.52796124 4.17710292
H 23.58454494 -6.49575673 2.73411711
C 19.24994842 -5.26297848 -1.04998388
C 19.36868838 -4.77054084 0.39190067
C 18.08861608 -4.08353577 0.88547543
O 17.09566196 -3.93068702 0.16064825
N 18.14590433 -3.67708415 2.17135861
H 18.37502183 -5.91102666 -1.09600333
H 20.20440362 -4.06153284 0.52178464
H 19.58557247 -5.60754039 1.07919381
H 17.40545169 -3.11721220 2.57780188
H 18.98901323 -3.82558146 2.72490380
H 20.14358036 -5.82892254 -1.35364531
H 19.12102618 -4.41299788 -1.72000149
C 6.29800118 -6.61099994 1.89499968
C 7.36215667 -6.34234390 2.95074083
O 7.09459265 -6.17429292 4.14115265
H 6.67182400 -7.25576776 1.08528096
H 6.01600072 -5.65100076 1.46300004
H 5.42300178 -7.06900163 2.35500070
N 8.64340608 -6.26127601 2.48599330
C 9.76354081 -6.00233778 3.38531728
C 9.92407651 -4.48919992 3.60517472
O 10.37015087 -3.75402385 2.71682025
C 11.08082073 -6.51469820 2.80649044
C 11.02205527 -7.97166652 2.37613871
O 10.58012946 -8.86657717 3.09450670
N 11.50243439 -8.18107138 1.12234625
H 8.82961064 -6.32702374 1.49164840
H 9.53145959 -6.50799543 4.33189376
H 11.85808225 -6.40263460 3.57973637
H 11.37821686 -5.87325674 1.96893378
H 11.56607097 -9.13271342 0.77852404
H 11.89290825 -7.41122892 0.57967579
N 9.59507342 -4.01862808 4.82889026
C 9.75200215 -2.59900552 5.11999623
H 9.01735892 -4.60470258 5.42327992
H 10.66400209 -2.28999843 4.60900216
H 9.84870363 -2.45215783 6.20451990
H 8.90500263 -2.02199840 4.74800174
C 11.66100659 -0.84000413 1.57600673
C 12.85478892 -1.60106875 1.02872546
O 13.77921358 -1.05149148 0.42865500
H 11.17399651 -1.45700039 2.33199631
H 10.95100280 -0.57899911 0.79100039
H 12.02724476 0.09482379 2.02427616
N 12.82314550 -2.93915044 1.29708045
C 13.90846390 -3.82167169 0.91802842
C 13.40562412 -4.83782156 -0.10743419
O 12.38222485 -5.50971970 0.09784559
C 14.51558743 -4.56602466 2.12358191
C 14.82805622 -3.61568478 3.29054963
C 13.65715466 -3.40051108 4.22358853
O 13.42942085 -2.07416188 4.45202342
N 13.01889721 -4.38310903 4.72842786
H 11.99964182 -3.34417795 1.74560483
H 14.68940977 -3.18975033 0.47661049
H 15.44212858 -5.04302335 1.77342896
H 13.83290136 -5.35604816 2.46861787
H 15.17399124 -2.63795359 2.92570349
H 15.64180933 -4.03612447 3.90217696
H 12.25307174 -4.07677438 5.34038906
N 14.14804806 -4.95637683 -1.22521758
C 13.90700306 -6.01800355 -2.19599806
H 15.04060571 -4.46787106 -1.24241534
H 13.09000080 -6.62900028 -1.81300236
H 13.66510730 -5.62139007 -3.19333614
H 14.81300208 -6.62200185 -2.23100115
O 16.80431358 2.95365476 10.80873324
H 16.83718745 3.32259545 9.90738362
H 16.27285548 3.58631088 11.31482767
N 20.63239620 0.72566684 5.61899379
C 20.91415112 -0.54044713 5.14160161
O 22.10212568 -0.89166125 5.00508918
N 19.88079654 -1.36914965 4.79484265
C 18.52265726 -1.12542369 5.06168693
O 17.66836250 -1.93779653 4.70655493
C 18.26767235 0.12496296 5.77295717
N 16.99910495 0.41928953 6.15928207
C 16.81157691 1.63161478 6.72750122
C 15.52161571 1.98090748 7.19192702
C 15.23336551 3.21141728 7.77650347
C 13.84420475 3.51756141 8.26168095
C 16.27774486 4.17407955 7.93126336
C 15.98990461 5.53975130 8.50354533
C 17.57258513 3.84198803 7.51339940
C 17.86782952 2.60154549 6.91772589
N 19.15528967 2.25202671 6.51829528
C 19.37270404 1.00277837 5.97747116
C 20.27410272 3.20280558 6.69569196
C 20.85317741 3.21094570 8.11826497
H 20.12524373 -2.25572496 4.31950441
H 14.74325070 1.22645970 7.06993246
H 13.83933372 3.66038632 9.35670352
H 13.45793816 4.45206855 7.82222083
H 13.14480239 2.70549227 8.02178633
H 16.90946301 6.13121768 8.61609387
H 15.30320183 6.10536960 7.85025262
H 15.49810241 5.48434776 9.48952981
H 18.35955954 4.58077336 7.65391760
H 19.89283263 4.19149695 6.40733946
H 21.04285152 2.91718272 5.97137351
H 21.49796296 2.34295421 8.25089293
H 21.44996018 4.12616547 8.25729100
H 20.05581038 3.19810945 8.86184865
H 12.74718131 -1.98254722 5.13783158

AsLOV2 **Qd2** S_1

C 19.02907459 2.11192868 13.81300377
C 19.33883527 0.93890214 12.92874218
O 20.07385056 0.01270951 13.26194864
H 18.15300917 2.65199726 13.45298062
H 18.86824503 1.75241893 14.83784764
H 19.89296190 2.77605386 13.82100980
N 18.78037711 0.97672736 11.66084524
C 18.87564849 -0.11068674 10.76446576
C 20.30029620 -0.00188661 9.95088495
O 20.25871303 -0.08317631 8.73550393
C 17.75443879 -0.18309995 9.72394914
S 16.17091276 -0.66095148 10.47884488
H 18.18208127 1.77293549 11.38506976
H 19.00482748 -1.04811451 11.33014507
H 18.00458712 -0.95513698 8.98592701
H 17.64917586 0.76742579 9.18853342
H 15.86806732 0.58811393 10.92785850
N 21.35279747 0.15472903 10.73780099

C 22.71306574 0.02587142 10.23615530
H 21.18933636 0.04775535 11.74319363
H 22.71798867 0.02803020 9.14600143
H 23.13106295 -0.94052494 10.56038167
H 23.37798161 0.80607329 10.60788555
C 25.51299474 -3.72098635 7.23815944
C 25.83790099 -2.22361665 7.23389697
C 24.66395926 -1.41333056 6.68040794
C 24.59550579 0.03076867 7.17659374
O 25.16213599 0.40491478 8.20942824
N 23.82210213 0.84074635 6.41747315
H 25.31631527 -4.09692887 6.21823665
H 26.75126590 -2.01269104 6.65131695
H 26.03951567 -1.88314843 8.26228294
H 23.70776047 -1.87029956 6.99998644
H 24.63249047 -1.43409987 5.58023309
H 23.60331655 1.75965510 6.78618084
H 23.20395849 0.43436563 5.70332602
H 26.30804466 -4.33201232 7.66590690
H 24.60697074 -3.84100309 7.83194997
C 23.25598418 -6.51502963 3.78410142
C 21.82313772 -5.96119441 3.94288373
C 21.67577773 -4.43592857 3.93115174
O 20.71241764 -3.89270771 3.35893836
N 22.58396977 -3.69613259 4.59426770
H 23.91601374 -5.85797310 4.34995587
H 21.40972043 -6.28595689 4.91559243
H 21.14144227 -6.34628791 3.17140515
H 22.47555432 -2.66794674 4.62675711
H 23.31721015 -4.12731392 5.14146651
H 23.34101820 -7.52802194 4.17694426
H 23.58400206 -6.49765319 2.73388735
C 19.25001906 -5.26302636 -1.05000492
C 19.35420284 -4.76495577 0.39059470
C 18.06813972 -4.07297646 0.86226302
O 17.08298473 -3.92919684 0.12332881
N 18.10920568 -3.65591429 2.14410635
H 18.37499850 -5.91099438 -1.09601271
H 20.18937604 -4.05805939 0.53103804
H 19.56100827 -5.59756370 1.08584953
H 17.38234427 -3.06659171 2.53287306
H 18.95582413 -3.78069637 2.69678005
H 20.14650533 -5.82784677 -1.34702279
H 19.12098956 -4.41299187 -1.71998682
C 6.29799647 -6.61100021 1.89500255
C 7.36217805 -6.34382502 2.95124682
O 7.09371892 -6.17591215 4.14159456
H 6.67156147 -7.25566473 1.08506036
H 6.01600214 -5.65100044 1.46299983
H 5.42300130 -7.06900280 2.35499867
N 8.64391499 -6.26534025 2.48775894
C 9.76314043 -6.00308212 3.38740741
C 9.92077217 -4.48951574 3.60586501
O 10.36285108 -3.75439253 2.71587368
C 11.08211022 -6.51159992 2.80951961
C 11.03203175 -7.97077930 2.38600920
O 10.58626807 -8.86380719 3.10473736
N 11.52644967 -8.18471175 1.13869339
H 8.83035668 -6.32683589 1.49316617
H 9.53200883 -6.50887492 4.33413840
H 11.85989457 -6.39141160 3.58123851
H 11.37636384 -5.87238397 1.96908868
H 11.59680961 -9.13772489 0.80009384
H 11.91643108 -7.41518283 0.59482659
N 9.59526562 -4.01890872 4.83073392
C 9.75200077 -2.59900576 5.11999444
H 9.01835578 -4.60455475 5.42622389
H 10.66400146 -2.28999971 4.60900031
H 9.84896922 -2.45094758 6.20436940
H 8.90500163 -2.02199783 4.74800488
C 11.66100415 -0.84000848 1.57601001
C 12.85205768 -1.60159612 1.02283635
O 13.77045035 -1.05314378 0.41343308
H 11.17399360 -1.45699919 2.33199539
H 10.95100439 -0.57899584 0.79100006
H 12.02941487 0.09400662 2.02395899
N 12.82339553 -2.93938194 1.29506635
C 13.91086055 -3.82004895 0.91733646
C 13.41026580 -4.84054681 -0.10463030
O 12.39095755 -5.51815558 0.10482776
C 14.52281789 -4.55745649 2.12477643
C 14.83003952 -3.60150389 3.28858998
C 13.65756726 -3.38965931 4.21999272
O 13.42124348 -2.06423538 4.44186127
N 13.02354243 -4.37349971 4.72929354
H 12.00353667 -3.34377164 1.74985574
H 14.68889472 -3.18702044 0.47252884
H 15.45228746 -5.02968162 1.77595995
H 13.84465402 -5.35057615 2.47204641
H 15.17024149 -2.62288392 2.92126788
H 15.64823375 -4.01253576 3.90048411
H 12.25506001 -4.06633460 5.33766496
N 14.14840515 -4.95598114 -1.22561239
C 13.90699935 -6.01800640 -2.19600515
H 15.04032980 -4.46588535 -1.24460452
H 13.08999979 -6.62899985 -1.81300389
H 13.66541375 -5.62139779 -3.19343724
H 14.81300335 -6.62200032 -2.23099490
O 16.76074721 2.69237666 10.83874764
H 16.69661887 3.02089377 9.91724415
H 16.21967321 3.30688135 11.35675745
N 20.70466387 0.71140480 5.59536359
C 20.97204719 -0.47384058 4.99403327
O 22.15473087 -0.83326839 4.74280539
N 19.93423990 -1.32178210 4.64707330
C 18.58937831 -1.13086271 4.96829529
O 17.74098721 -1.96398822 4.62876650
C 18.32949830 0.10211554 5.70948093
N 17.06553204 0.33773689 6.14386317
C 16.85868141 1.52061022 6.77840813
C 15.56438070 1.81405628 7.27727828
C 15.24803028 3.00744327 7.91990828
C 13.85450968 3.25523600 8.44287796
C 16.26332505 3.98417308 8.08761947
C 15.95441513 5.32816449 8.70765196
C 17.56447300 3.70289070 7.62708027
C 17.88583610 2.49772711 6.98595187
N 19.18345470 2.19950692 6.53711447
C 19.43587558 0.97651218 5.92212282
C 20.25960278 3.18812838 6.70014646
C 20.85343808 3.21147666 8.11814961
H 20.17586953 -2.17661433 4.12919246
H 14.80409571 1.04574044 7.12407893
H 13.85660369 3.36495586 9.54183655
H 13.42374924 4.18413144 8.03406197
H 13.17921802 2.42564141 8.19030658
H 16.86565824 5.92954935 8.83937275
H 15.25948274 5.90862732 8.07624602
H 15.46607555 5.23678506 9.69301672
H 18.32924234 4.46307455 7.77904129
H 19.85099804 4.17209149 6.42634264
H 21.03415174 2.93444073 5.96934273
H 21.49769546 2.34276732 8.25096897
H 21.45766399 4.12069314 8.26309470
H 20.05581716 3.19784586 8.86185119
H 12.73896982 -1.97258900 5.12662955

AsLOV2 **Qa** T_1

C 19.02917980 2.11192613 13.81246776
C 19.30611169 0.93423863 12.89847091
O 19.95666966 -0.05564746 13.25628623
H 18.15296144 2.65199946 13.45309635
H 18.86603577 1.74482913 14.83442238
H 19.89297053 2.77603559 13.82127306
N 18.79654443 1.06310390 11.64594599
C 18.92262853 0.03594825 10.61956512
C 20.28111998 0.11336363 9.87731910
O 20.34399079 0.14752682 8.64456657
C 17.77406375 0.15398065 9.61921888
S 16.11285572 -0.18697472 10.32628514
H 18.45918663 1.98385095 11.34525578
H 18.88631601 -0.93979640 11.13105933
H 17.90981074 -0.56659440 8.80362227
H 17.78035837 1.15322777 9.16235174
H 16.22455429 0.66372038 11.37935993
N 21.34814547 0.13511623 10.70748036
C 22.71316527 0.02574816 10.23649646
H 21.15697177 -0.04281421 11.69578835
H 22.71792792 0.02792188 9.14599985
H 23.14781353 -0.93712061 10.55967946
H 23.37790948 0.80625108 10.60763842
C 25.51286287 -3.72105587 7.23796317
C 25.85195689 -2.22590265 7.24427355
C 24.67977616 -1.38611669 6.73179132
C 24.69507578 0.06929554 7.19809453
O 25.38127712 0.46155706 8.14222448
N 23.86023787 0.88846276 6.50571632
H 25.30236081 -4.09136722 6.21942412
H 26.75516393 -2.01667905 6.64499974
H 26.08386094 -1.90225922 8.27158334
H 23.72460921 -1.80568048 7.10034136
H 24.60177977 -1.42080060 5.63321771
H 23.71435069 1.82423576 6.86667262
H 23.19035107 0.50542430 5.83940099
H 26.30805567 -4.33195540 7.66596326
H 24.60706138 -3.84099869 7.83209322
C 23.25608266 -6.51499565 3.78403616
C 21.82440171 -5.97345719 3.91369155
C 21.63706773 -4.46030918 3.97165488
O 20.48060329 -3.99102507 3.90847446
N 22.69649836 -3.65736752 4.14497738
H 23.91602278 -5.85800575 4.34998245
H 21.36232657 -6.33126039 4.85092466
H 21.16659163 -6.33685237 3.10867159
H 22.54236108 -2.65349346 4.29615296
H 23.63679257 -4.02045945 4.21820774
H 23.34093613 -7.52801109 4.17698898
H 23.60063875 -6.50903421 2.73823519
C 19.24991836 -5.26288193 -1.04999541
C 19.39460336 -4.77157189 0.38799789
C 18.15058422 -4.02579619 0.88465435
O 17.12566832 -3.91166546 0.20114408
N 18.29043698 -3.49808773 2.12019324
H 18.37510333 -5.93115363 -1.09062224
H 20.26391770 -4.10179711 0.50665180
H 19.57249915 -5.61685967 1.07780757
H 17.51482612 -3.02238038 2.56356288
H 19.08007448 -3.75271324 2.71317926
H 20.13746899 -5.83304557 -1.36470828
H 19.12095172 -4.41299700 -1.71998586
C 6.29793947 -6.61099296 1.89513750
C 7.35673388 -6.37085798 2.96049486
O 7.07549088 -6.21811012 4.14926677
H 6.66843949 -7.25506067 1.08294490
H 6.01606057 -5.65101244 1.46293550
H 5.42297516 -7.06901095 2.35494128
N 8.64660612 -6.32116416 2.51297976
C 9.74315594 -6.01493088 3.42591077
C 9.86838828 -4.49295760 3.60168161
O 10.23634962 -3.77622654 2.66271246
C 11.08419259 -6.51769562 2.89348411
C 11.06225676 -7.98849674 2.50087254
O 10.61594391 -8.86878923 3.23454312
N 11.57938438 -8.22711028 1.26823238
H 8.83402948 -6.30408545 1.51658223
H 9.49963186 -6.50431135 4.37765860
H 11.83268719 -6.38804164 3.69268308
H 11.40060238 -5.88976681 2.05232304
H 11.65909123 -9.18787481 0.95455260
H 11.97827057 -7.46946297 0.71411303
N 9.58599332 -4.01444405 4.82810494
C 9.75203565 -2.59894201 5.11999869
C 9.10258096 -4.62210321 5.47950701
H 10.66400088 -2.29001831 4.60899045
H 9.85508593 -2.45476172 6.20373238
H 8.90496871 -2.02203337 4.74802087
C 11.66098182 -0.84004202 1.57596119
C 12.83999212 -1.60444439 0.99651851
O 13.73808851 -1.06108161 0.34919087
H 11.17401302 -1.45698805 2.33201753
H 10.95099571 -0.57896580 0.79101612
H 12.03470622 0.09449256 2.01917553
N 12.81296339 -2.93750823 1.27451657
C 13.91159748 -3.82262664 0.93767678
C 13.43838401 -4.88638536 -0.06007111
O 12.47992939 -5.63136347 0.19487420
C 14.50192317 -4.49928130 2.18793912
C 15.07456752 -3.49153293 3.18789982
C 14.08014673 -2.91156862 4.19344483
O 12.92065783 -3.31590588 4.30437129
N 14.58950597 -1.91022333 4.95646210
H 12.06062844 -3.30714958 1.85827865
H 14.69173090 -3.20354426 0.47575729
H 15.31009420 -5.16441565 1.84788409
H 13.73041359 -5.11469170 2.67381822
H 15.53914662 -2.64608055 2.65417507
H 15.87947008 -3.95826768 3.77904155
H 14.01707611 -1.56645622 5.71918075
H 15.58840365 -1.69546968 4.97262880
N 14.12981996 -4.95381664 -1.21843836
C 13.90706894 -6.01797217 -2.19610229
H 14.98385796 -4.40551831 -1.27928239
H 13.09000053 -6.62896657 -1.81294865
H 13.66729809 -5.61621805 -3.19139726
H 14.81297361 -6.62204202 -2.23098706
O 17.89584808 3.61616416 10.71411031
H 18.51856190 4.35951414 10.69201108
H 17.53796984 3.58454434 9.81050814
N 20.61223841 0.70216011 5.61689174
C 20.88046116 -0.55364780 5.08706239
O 22.05836135 -0.87162519 4.84700539
N 19.84468808 -1.41225137 4.82171614
C 18.50652180 -1.15023194 5.09304335
O 17.62863754 -1.95615379 4.76667850
C 18.25403122 0.12081241 5.77094794
N 16.98137148 0.40350587 6.14991124
C 16.80522721 1.61545968 6.72779555
C 15.52266179 1.95694392 7.20558498
C 15.23862860 3.17661006 7.80945655
C 13.85947574 3.47218936 8.31856047
C 16.28256874 4.15586897 7.94862911
C 15.99736389 5.49070271 8.58438932
C 17.56296834 3.84068814 7.50292068
C 17.86819460 2.59245048 6.91112954
N 19.14811947 2.26049128 6.51673834
C 19.36806305 0.98416370 5.96137057
C 20.27273998 3.20538656 6.69396647
C 20.85341935 3.21125106 8.11840960
H 20.08181516 -2.34351925 4.42146540
H 14.74301558 1.20409835 7.08662361
H 13.88131171 3.68272421 9.40138560
H 13.44398900 4.37255517 7.83532258
H 13.17433604 2.63302983 8.14219506
H 16.89330982 6.12726203 8.60235547
H 15.20604965 6.03182613 8.04010401
H 15.64415205 5.37100355 9.62229742
H 18.34710043 4.58690869 7.61838425
H 19.90233231 4.19692537 6.40152248
H 21.03973017 2.91555907 5.96994785
H 21.49780923 2.34282705 8.25079842
H 21.45138456 4.12600416 8.25473629
H 20.05568608 3.19805514 8.86182362

AsLOV2 **Qb** T_1

C 19.02901141 2.11201205 13.81290333
C 19.33321091 0.93856719 12.90627088
O 20.06186592 -0.00097711 13.25215134
H 18.15302505 2.65200440 13.45295522
H 18.86662469 1.74736360 14.83617216
H 19.89301789 2.77598008 13.82111648
N 18.78025397 1.01676896 11.66919338
C 18.92895262 -0.01994943 10.65069022
C 20.27895771 0.08790243 9.89306572
O 20.32711389 0.12176315 8.66019555
C 17.77587074 0.05875207 9.65458263
S 16.11318386 -0.27850751 10.39468374
H 18.22339049 1.84077326 11.41839803
H 18.92852007 -0.99455761 11.16795897
H 17.92242632 -0.66803100 8.84782805
H 17.75764619 1.05515210 9.19452910
H 16.45402386 0.00124586 11.67984731
N 21.35111828 0.12548785 10.71361061
C 22.71316898 0.02581827 10.23616128
H 21.16838401 -0.03768020 11.70727097
H 22.71792940 0.02805125 9.14600127
H 23.15368464 -0.93575532 10.55656572
H 23.37795171 0.80609793 10.60788793
C 25.51302818 -3.72099225 7.23808377
C 25.86540668 -2.22823684 7.26796270
C 24.69964142 -1.36531045 6.78327915
C 24.75136717 0.08640165 7.25965986
O 25.43942291 0.45034213 8.21397858
N 23.94424693 0.93238499 6.56909429
H 25.32133055 -4.07948455 6.21156616
H 26.76537355 -2.01846111 6.66432677
H 26.11016332 -1.92830386 8.29955575
H 23.74572286 -1.76784224 7.17460139
H 24.59816793 -1.39418060 5.68709123
H 23.82918840 1.87394907 6.92601561
H 23.28740064 0.57584618 5.87576329
H 26.30801375 -4.33201395 7.66596342
H 24.60697693 -3.84099989 7.83196050
C 23.25601673 -6.51499602 3.78394926
C 21.82404536 -5.95822218 3.94161353
C 21.70146632 -4.43520921 3.99102905
O 20.74551461 -3.84681582 3.44636023
N 22.62818835 -3.74196934 4.67635002
H 23.91600162 -5.85802072 4.35002638
H 21.39011018 -6.31471090 4.89383571
H 21.15000288 -6.30301598 3.14487702
H 22.55722510 -2.71955045 4.73136159
H 23.35212336 -4.20402921 5.21030277
H 23.34100624 -7.52799326 4.17701835
H 23.58500686 -6.49704067 2.73425468
C 19.24992545 -5.26294409 -1.04997254
C 19.36808353 -4.78057303 0.39483422
C 18.07014377 -4.14483591 0.90807843
O 17.06504726 -4.01758734 0.19516898
N 18.12188233 -3.75712800 2.20010625
H 18.37503808 -5.91104714 -1.09600858
H 20.18152850 -4.04688235 0.52726528
H 19.61837231 -5.61768212 1.07046052
H 17.36898947 -3.21843499 2.61085013
H 18.98091343 -3.86041403 2.73841902
H 20.14360173 -5.82726150 -1.35671304
H 19.12102734 -4.41301035 -1.72001638
C 6.29800499 -6.61101100 1.89501120
C 7.35967790 -6.33728798 2.95049467
O 7.09187558 -6.15818143 4.13907417
H 6.67371083 -7.25579215 1.08634301
H 6.01600760 -5.65100097 1.46299631
H 5.42299442 -7.06899168 2.35499706
N 8.64269502 -6.26102920 2.48786824
C 9.75798042 -6.01436492 3.39466009
C 9.93007696 -4.50143680 3.61290010

O 10.37928595 -3.77254075 2.72360210
C 11.07556271 -6.55075036 2.82766957
C 10.95185155 -7.97920703 2.31581409
O 10.56660914 -8.90916683 3.02084944
N 11.27958224 -8.11135111 1.00336187
H 8.83577508 -6.34965089 1.49646538
H 9.51075675 -6.51388655 4.34079932
H 11.82916245 -6.52759753 3.62937700
H 11.42100486 -5.88180586 2.03114021
H 11.29483736 -9.04361871 0.60403812
H 11.69404566 -7.33197246 0.49071734
N 9.61241493 -4.02667627 4.84237100
C 9.75200400 -2.59914533 5.11997549
H 9.00391115 -4.60005001 5.41985339
H 10.66396341 -2.28990025 4.60898335
H 9.85078308 -2.44468221 6.20324104
H 8.90502740 -2.02194615 4.74802630
C 11.66102124 -0.83998802 1.57601336
C 12.85418846 -1.60131920 1.02904723
O 13.79028412 -1.05515718 0.44457768
H 11.17399376 -1.45700348 2.33199217
H 10.95099492 -0.57901422 0.79100193
H 12.02782733 0.09249145 2.02801722
N 12.80202751 -2.94162528 1.27687555
C 13.88886889 -3.83386609 0.93290888
C 13.39581450 -4.84633986 -0.10250766
O 12.36399650 -5.50932567 0.09315730
C 14.44595183 -4.58480811 2.15726859
C 14.78797311 -3.65179502 3.33254272
C 13.62983377 -3.28092693 4.22532002
O 12.96253295 -4.39876881 4.65216609
N 13.38387536 -2.07340584 4.55387757
H 11.97621303 -3.33710268 1.72680055
H 14.69022047 -3.21419907 0.51139732
H 15.36026655 -5.10018379 1.82843382
H 13.72943345 -5.35104383 2.48630200
H 15.24478791 -2.71420645 2.98708877
H 15.52423715 -4.15753114 3.98030404
H 12.60780137 -2.02190591 5.22618015
N 14.14665225 -4.96431836 -1.21443543
C 13.90701036 -6.01802143 -2.19596484
H 15.04376850 -4.48375516 -1.21831227
H 13.08999043 -6.62899324 -1.81301405
H 13.66573623 -5.61425131 -3.19035378
H 14.81300520 -6.62199646 -2.23101457
O 16.83359931 2.98101183 10.82625345
H 17.05902654 3.31109968 9.94072004
H 16.18513953 2.27534539 10.63942726
N 20.71191275 0.71843548 5.59213000
C 21.02262563 -0.51605150 5.03395800
O 22.21409858 -0.81485478 4.84105711
N 20.01197228 -1.37486966 4.69328095
C 18.66592935 -1.19409362 5.01937486
O 17.82398242 -2.02864760 4.69481593
C 18.37960012 0.04357269 5.75169615
N 17.10190302 0.26774367 6.15866833
C 16.87781736 1.47326611 6.72339179
C 15.57342180 1.77066590 7.18327328
C 15.23476010 2.98760343 7.75824357
C 13.83349647 3.24490877 8.23029000
C 16.24137853 4.00572165 7.90519583
C 15.89116416 5.33996027 8.50931839
C 17.54327188 3.73055845 7.49410475
C 17.89975436 2.49439709 6.90933933
N 19.19182316 2.21020624 6.51353301
C 19.45807610 0.95019065 5.94417993
C 20.27712024 3.19699452 6.69454687
C 20.85323991 3.21113979 8.11815395
H 20.26754690 -2.24548790 4.18966192
H 14.82634137 0.98686042 7.05152635
H 13.82021291 3.48378032 9.30733916
H 13.39659747 4.11616192 7.71398001
H 13.18439020 2.37640175 8.05896267
H 16.76167222 6.01016294 8.53352242
H 15.09087300 5.83929160 7.93851293
H 15.52158910 5.22426483 9.54207891
H 18.29778799 4.50321195 7.62964265
H 19.86907654 4.17595002 6.40934086
H 21.05615610 2.94250822 5.97002294
H 21.49783156 2.34288400 8.25094722
H 21.44932733 4.12695794 8.25520255
H 20.05584891 3.19803282 8.86188850
H 12.20713661 -4.14332728 5.20843601

AsLOV2 **Qc** T_1

C 19.02907555 2.11199060 13.81299736
C 19.33290595 0.94604536 12.90287430
O 20.07404181 0.01296621 13.24261679
H 18.15299160 2.65196202 13.45297030
H 18.86911601 1.74477536 14.83579803
H 19.89298524 2.77602388 13.82103361
N 18.77717358 1.02365450 11.66820636
C 18.92929431 -0.03184032 10.67267552
C 20.27489246 0.08686981 9.90316671
O 20.31447259 0.13300406 8.67103887
C 17.77594884 -0.03130837 9.67681193
S 16.09901001 -0.30630833 10.40045235
H 18.13516796 1.79321461 11.47352449
H 18.96501721 -0.99142046 11.21781540
H 17.96148955 -0.77399126 8.89324024
H 17.71069938 0.94722707 9.18558606
H 16.45722723 -1.23733429 11.32332922
N 21.35228580 0.11925269 10.71772671
C 22.71300641 0.02611725 10.23604465
H 21.17390793 -0.04206543 11.71235821
H 22.71798359 0.02790570 9.14600127
H 23.15673053 -0.93497939 10.55421814
H 23.37803669 0.80597678 10.60798747
C 25.51301273 -3.72099467 7.23809403
C 25.84572631 -2.22502073 7.23175105
C 24.66641396 -1.39397086 6.71981762
C 24.69083263 0.06881850 7.16306892
O 25.34349191 0.45766653 8.13299753
N 23.90505003 0.89390599 6.42526566
H 25.30324279 -4.09996243 6.22227353
H 26.74412656 -2.01641184 6.62503796
H 26.08101984 -1.89195683 8.25521964
H 23.71807848 -1.80522716 7.11538296
H 24.57082359 -1.45104564 5.62367601
H 23.76567205 1.83877018 6.76492104
H 23.26462305 0.51930424 5.72453095
H 26.30802376 -4.33201037 7.66594966
H 24.60697799 -3.84099515 7.83196352
C 23.25603223 -6.51500650 3.78402166
C 21.83138553 -5.94984626 3.89798951
C 21.67519521 -4.42960339 3.85199275
O 20.56250596 -3.93028775 3.58866579
N 22.72204032 -3.64411822 4.15328676
H 23.91599283 -5.85798760 4.34999681
H 21.38348054 -6.23967764 4.86596584
H 21.15895439 -6.35341821 3.12598701
H 22.58573436 -2.62886130 4.22677157
H 23.62535202 -4.02527298 4.39835984
H 23.34098894 -7.52801040 4.17697796
H 23.60696242 -6.51073847 2.74011741
C 19.25003991 -5.26304835 -1.05004885
C 19.34606695 -4.76299136 0.39238809
C 18.04513856 -4.09978704 0.86420566
O 17.07609341 -3.93234972 0.11183815
N 18.05116710 -3.72965600 2.16325056
H 18.37498781 -5.91098253 -1.09597989
H 20.16275430 -4.03254605 0.52645314
H 19.57482695 -5.59049897 1.08716243
H 17.30753427 -3.15244396 2.53745125
H 18.87551170 -3.87140618 2.74530226
H 20.14863559 -5.82610382 -1.34294373
H 19.12099162 -4.41298365 -1.71997663
C 6.29798826 -6.61098237 1.89500972
C 7.35471549 -6.34957945 2.95885703
O 7.07542685 -6.17402220 4.14537877
H 6.67390478 -7.25575326 1.08635819
H 6.01599172 -5.65100140 1.46300472
H 5.42300501 -7.06902095 2.35498988
N 8.64392012 -6.29254441 2.51092752
C 9.74794669 -6.03186508 3.42808520
C 9.92543819 -4.51541839 3.62310133
O 10.38746052 -3.81093697 2.71951508
C 11.07073831 -6.57926624 2.89023894
C 10.98551506 -8.03481885 2.45147553
O 10.51732035 -8.91892311 3.16617860
N 11.46946654 -8.25103433 1.20152703
H 8.84089773 -6.34178821 1.51767251
H 9.48530787 -6.51809372 4.37658744
H 11.81702249 -6.51150319 3.69787787
H 11.42401313 -5.94159215 2.07163115
H 11.51130970 -9.20414044 0.85794511
H 11.90623172 -7.49605778 0.67235954
N 9.59220184 -4.02434400 4.83826507
C 9.75198765 -2.59891297 5.11999028
H 8.97996472 -4.59370621 5.41496019
H 10.66403411 -2.29001800 4.60909745
H 9.85240581 -2.44830906 6.20354071
H 8.90498326 -2.02206730 4.74793713
C 11.66099632 -0.83999238 1.57599970
C 12.85893730 -1.59534281 1.03441046
O 13.82294002 -1.03727762 0.50808940
H 11.17400434 -1.45700692 2.33199688
H 10.95100138 -0.57899882 0.79100150
H 12.02515965 0.09065672 2.03243694
N 12.78007444 -2.94897734 1.20160801
C 13.90831478 -3.81723810 0.92455974
C 13.46346288 -4.90219049 -0.05717167
O 12.52624430 -5.66824776 0.22036979
C 14.48489747 -4.46958798 2.19593810
C 14.74036573 -3.44936494 3.32663288
C 13.51134749 -3.15166189 4.17944253
O 12.97003235 -2.04987925 4.22366614
N 13.03857657 -4.21760503 4.89043339
H 11.97279581 -3.34332738 1.68621688
H 14.68868697 -3.18964404 0.47650797
H 15.43320999 -4.95020931 1.91245385
H 13.80237016 -5.26246385 2.53937005
H 15.08606659 -2.48881079 2.92036808
H 15.53219651 -3.82878908 3.99337673
H 12.22258568 -4.09931422 5.47934794
H 13.55321023 -5.08591518 4.95575173
N 14.13321141 -4.95248348 -1.22514450
C 13.90699200 -6.01799651 -2.19603139
H 14.98277542 -4.39739171 -1.29330381
H 13.09000990 -6.62900795 -1.81299464
H 13.66809006 -5.61875831 -3.19250939
H 14.81300102 -6.62200318 -2.23099332
O 16.34175938 2.73767775 11.52610650
H 15.90898103 3.02337538 12.34578174
H 16.00751235 1.82534308 11.38752180
N 20.66787436 0.72878008 5.51481948
C 20.96263873 -0.49241620 4.92038982
O 22.14689217 -0.77635316 4.65869134
N 19.94507025 -1.35829711 4.62166587
C 18.60586625 -1.17587684 4.97344747
O 17.75894865 -2.01404521 4.66676061
C 18.33436589 0.05788428 5.71565818
N 17.06906990 0.27006373 6.15931123
C 16.88042128 1.43159906 6.83085081
C 15.62591343 1.66822233 7.43169157
C 15.35455409 2.80398269 8.18935565
C 14.03826939 2.95730075 8.89055398
C 16.36195723 3.81894182 8.32854070
C 16.09386841 5.05282533 9.14279160
C 17.60239739 3.61831525 7.73933310
C 17.91214012 2.44120224 7.02247319
N 19.17742467 2.19535402 6.52795584
C 19.42443604 0.95373832 5.90598646
C 20.25950886 3.18838575 6.69924639
C 20.85330219 3.21119767 8.11817766
H 20.18710476 -2.25375965 4.15673226
H 14.87237974 0.88985070 7.30440433
H 14.21243961 3.03152345 9.97734741
H 13.52510526 3.88475382 8.58646264
H 13.37190320 2.10709820 8.69424774
H 16.92855445 5.76494273 9.07663095
H 15.17596380 5.56820654 8.81647937
H 15.96031567 4.77148967 10.19990047
H 18.35836079 4.38987553 7.86669458
H 19.83746105 4.16527061 6.42284536
H 21.03009063 2.94050143 5.96355946
H 21.49783151 2.34286476 8.25094966
H 21.44685772 4.12979930 8.24572403
H 20.05581557 3.19797795 8.86185188

AsLOV2 **Qd** T_1

C 19.02906406 2.11199566 13.81293296
C 19.33413919 0.94071167 12.90293077
O 20.06638080 0.00418649 13.25315128
H 18.15300118 2.65197850 13.45297189
H 18.86374967 1.74664586 14.83527367
H 19.89299926 2.77600440 13.82108748
N 18.78932751 1.02262099 11.66625005
C 18.92778314 -0.02400031 10.65879065
C 20.27513964 0.08739338 9.89679769
O 20.32203268 0.13315358 8.66345512
C 17.76537895 0.02375577 9.66902594
S 16.10545819 -0.28818806 10.39711303
H 18.21306925 1.83360715 11.42965606
H 18.93523168 -0.99041650 11.19020394
H 17.90943560 -0.73878770 8.89418016
H 17.75055109 0.99134369 9.15135962
H 15.97991941 0.92151250 11.00017348
N 21.35116469 0.12064689 10.71455673
C 22.71303393 0.02595786 10.23613064
H 21.16896388 -0.04662983 11.70789915
H 22.71797925 0.02798958 9.14600133
H 23.15631111 -0.93520372 10.55457533
H 23.37800499 0.80604208 10.60790925
C 25.51300760 -3.72100054 7.23804244
C 25.84927587 -2.22565698 7.23704769
C 24.67266873 -1.38992963 6.72756668
C 24.70346589 0.07271256 7.16986626
O 25.36954801 0.46405029 8.12918481
N 23.90772285 0.89761134 6.44107658
H 25.30299196 -4.09671365 6.22123825
H 26.74858747 -2.01707441 6.63161801
H 26.08531829 -1.89712059 8.26182647
H 23.72290420 -1.79782857 7.12285457
H 24.57602577 -1.44587172 5.63144406
H 23.77346005 1.84272429 6.78188662
H 23.25455345 0.52042109 5.75434013
H 26.30800963 -4.33201429 7.66596924
H 24.60699608 -3.84099340 7.83199061
C 23.25603266 -6.51499417 3.78399667
C 21.82876134 -5.95695316 3.90128737
C 21.66345631 -4.43800244 3.88720943
O 20.53795621 -3.94235646 3.67325937
N 22.71537120 -3.65093636 4.16305497
H 23.91599902 -5.85801030 4.35001628
H 21.37551330 -6.26751448 4.86009920
H 21.16148576 -6.34889755 3.11864108
H 22.57295722 -2.63900814 4.26334126
H 23.62879130 -4.03046529 4.37011420
H 23.34099857 -7.52800659 4.17698806
H 23.60572113 -6.51011759 2.73971754
C 19.24998853 -5.26300084 -1.04997761
C 19.35441684 -4.76323162 0.39160680
C 18.06510791 -4.07916839 0.86525455
O 17.08710776 -3.92090203 0.12178569
N 18.09642837 -3.67312080 2.15192784
H 18.37500940 -5.91100862 -1.09602022
H 20.18379698 -4.04669171 0.52304494
H 19.56912443 -5.59470350 1.08637849
H 17.33575592 -3.13132518 2.54344832
H 18.90766964 -3.85086960 2.74286436
H 20.14674997 -5.82756461 -1.34590240
H 19.12099977 -4.41299790 -1.71999643
C 6.29799652 -6.61099909 1.89500084
C 7.36156415 -6.34251446 2.95114534
O 7.09363404 -6.17508124 4.14152705
H 6.67203376 -7.25579803 1.08541038
H 6.01600172 -5.65100081 1.46299928
H 5.42300252 -7.06900317 2.35500060
N 8.64276599 -6.26105513 2.48659891
C 9.76285245 -6.00311366 3.38613697
C 9.92392580 -4.48992928 3.60553957
O 10.36949040 -3.75513251 2.71656684
C 11.07937454 -6.51742003 2.80722251
C 11.01720060 -7.97353103 2.37445065
O 10.57510656 -8.86897233 3.09198914
N 11.49455245 -8.18163591 1.11918068
H 8.82912465 -6.32676847 1.49229049
H 9.53001383 -6.50837734 4.33273677
H 11.85645947 -6.40848074 3.58096660
H 11.37832481 -5.87537218 1.97070096
H 11.55612127 -9.13290123 0.77392927
H 11.88647832 -7.41187976 0.57746018
N 9.59504908 -4.01875103 4.82903580
C 9.75200236 -2.59900504 5.11999679
H 9.01834647 -4.60490372 5.42432441
H 10.66400119 -2.28999866 4.60900046
H 9.84872252 -2.45212259 6.20451191
H 8.90500226 -2.02199830 4.74800273
C 11.66100151 -0.84001236 1.57600815
C 12.85627337 -1.60116307 1.03185599
O 13.78516492 -1.05110266 0.43899706
H 11.17399466 -1.45699913 2.33199613
H 10.95100624 -0.57899257 0.79099947
H 12.02619466 0.09513991 2.02455215
N 12.82065985 -2.93991129 1.29620294
C 13.90623496 -3.82336470 0.91978599
C 13.40481083 -4.83921748 -0.10677346
O 12.38167234 -5.51170469 0.09777022
C 14.50973854 -4.56837119 2.12696351
C 14.81793618 -3.62013133 3.29697423
C 13.64756756 -3.41103169 4.23218079
O 13.42251424 -2.08594608 4.47113532
N 13.00971641 -4.39680502 4.73045788
H 11.99573967 -3.34440643 1.74259367
H 14.68842587 -3.19202926 0.47977734
H 15.43736503 -5.04609205 1.78015084
H 13.82641074 -5.35907738 2.46895234
H 15.15896345 -2.63940926 2.93469865
H 15.63308468 -4.03935548 3.90788066
H 12.24536147 -4.09530846 5.34655350
N 14.14759381 -4.95661706 -1.22462561
C 13.90700623 -6.01800370 -2.19599727
H 15.04011109 -4.46789484 -1.24131171
H 13.08999826 -6.62899932 -1.81300628
H 13.66516403 -5.62113622 -3.19324606
H 14.81300081 -6.62200381 -2.23099939
O 16.90359967 3.08364722 10.90372595
H 17.06268178 3.35254435 9.98186161
H 16.79837981 3.91973197 11.38271529
N 20.65975889 0.71838214 5.55860070
C 20.94243421 -0.51962796 4.99058180
O 22.12352434 -0.81249224 4.73018850
N 19.91720500 -1.38212070 4.71227408
C 18.57700249 -1.17185674 5.04605801

O 17.71725764 -1.99410381 4.73595396
C 18.31989030 0.07822331 5.76811525
N 17.05447705 0.32320423 6.20036961
C 16.86433742 1.52525323 6.78444255
C 15.58956012 1.82460693 7.31904286
C 15.28970214 3.03756581 7.92182162
C 13.94243423 3.27314036 8.53759466
C 16.29884871 4.06162524 7.99354086
C 15.97236371 5.41747553 8.56450237
C 17.57769624 3.78011551 7.52111305
C 17.90249513 2.53506188 6.93513572
N 19.18104456 2.23139457 6.51319047
C 19.41733369 0.96611472 5.93903686
C 20.28460472 3.19720078 6.69073547
C 20.85331246 3.21123256 8.11819470
H 20.15256367 -2.28623538 4.25778177
H 14.83954148 1.03674694 7.24891015
H 14.05424716 3.40181955 9.62867210
H 13.47425984 4.19435446 8.15487414
H 13.26276992 2.42982066 8.35931854
H 16.86232157 6.06088251 8.60734022
H 15.21308485 5.93092775 7.95023585
H 15.54967735 5.34497004 9.58036928
H 18.33958728 4.55231714 7.60863669
H 19.89905067 4.18191084 6.39365764
H 21.06195371 2.91945340 5.97317318
H 21.49781082 2.34284746 8.25093421
H 21.45081189 4.12604723 8.25685014
H 20.05581282 3.19798327 8.86184907
H 12.74432375 -1.99817247 5.16152446

AsLOV2 **Qa** T_{1-H}

C 19.02910952 2.11196333 13.81254804
C 19.30642221 0.93468564 12.89792237
O 19.97257858 -0.04676362 13.25520893
H 18.15298324 2.65200875 13.45306111
H 18.86528042 1.74426713 14.83417134
H 19.89300551 2.77599239 13.82125348
N 18.78717138 1.05080417 11.65023153
C 18.92465237 0.01773535 10.63297850
C 20.28259173 0.08852485 9.89041412
O 20.34288502 0.09569437 8.65787855
C 17.78370790 0.11709971 9.61995046
S 16.11561178 -0.14411713 10.27697963
H 18.40572052 1.95421208 11.35223706
H 18.88871657 -0.95487238 11.15110017
H 17.94016434 -0.60649568 8.80360185
H 17.79702005 1.10237473 9.12483524
N 21.35209287 0.13526538 10.71611129
C 22.71324327 0.02578256 10.23641830
H 21.16697624 -0.02854283 11.70812199
H 22.71791030 0.02788896 9.14600032
H 23.15116274 -0.93686431 10.55741500
H 23.37788445 0.80624610 10.60769764
C 25.51285392 -3.72105327 7.23795660
C 25.84449608 -2.22504139 7.22884661
C 24.67226375 -1.40404698 6.68765344
C 24.66551250 0.05855542 7.13060617
O 25.33001048 0.46969996 8.08356216
N 23.84039251 0.85722154 6.40562329
H 25.30106269 -4.09848633 6.22194380
H 26.75378923 -2.01913605 6.63751643
H 26.06098604 -1.88419702 8.25393305
H 23.71569268 -1.82640391 7.04895640
H 24.60858298 -1.45794895 5.58894420
H 23.65818794 1.78674464 6.76716920
H 23.16697509 0.44668249 5.75557952
H 26.30806106 -4.33194951 7.66596617
H 24.60706657 -3.84100651 7.83209745
C 23.25606779 -6.51499152 3.78402003
C 21.81976458 -5.98360840 3.91666840
C 21.61030358 -4.47264013 3.98107398
O 20.44252433 -4.02555645 3.94035865
N 22.65719102 -3.65216021 4.13556246
H 23.91602612 -5.85801532 4.34999149
H 21.36154937 -6.35098410 4.85205880
H 21.16575754 -6.35211331 3.11058709
H 22.48820456 -2.64659257 4.29008514
H 23.60371899 -4.00250237 4.19047969
H 23.34095174 -7.52800887 4.17699356
H 23.59886442 -6.50815725 2.73768181
C 19.24991312 -5.26288287 -1.04999237
C 19.39247903 -4.77010912 0.38921132
C 18.14731824 -4.02792940 0.88855838
O 17.11060534 -3.93997890 0.21859264
N 18.29717764 -3.47150982 2.11187097
H 18.37510343 -5.91113477 -1.09602374
H 20.26104416 -4.09973654 0.50833400
H 19.57083420 -5.61517816 1.07931596
H 17.51391424 -3.01003078 2.55803103
H 19.09139696 -3.71648215 2.70468109
H 20.13842643 -5.83237335 -1.36248244
H 19.12096019 -4.41300012 -1.71999091
C 6.29793318 -6.61099788 1.89514522
C 7.36530634 -6.36334107 2.95045915
O 7.10137215 -6.22733093 4.14529306
H 6.66657742 -7.25485498 1.08189192
H 6.01606676 -5.65101214 1.46293348
H 5.42297252 -7.06900984 2.35493740
N 8.64539055 -6.27790668 2.48166988
C 9.76010777 -5.99187822 3.37898349
C 9.88912563 -4.47375387 3.58600799
O 10.27216756 -3.73943270 2.66679853
C 11.08975393 -6.48679045 2.80477250
C 11.01510517 -7.92234387 2.30506954
O 10.68155840 -8.86375369 3.02172611
N 11.32559219 -8.05205955 0.98795180
H 8.82135802 -6.28332699 1.48311691
H 9.53500741 -6.49912833 4.32621674
H 11.84551434 -6.42713254 3.60320453
H 11.40245312 -5.80975828 2.00150146
H 11.35979932 -8.98470773 0.59109795
H 11.68814978 -7.25819414 0.45901325
N 9.58706601 -4.01245233 4.81432728
C 9.75203480 -2.59894274 5.11999810
H 9.08815204 -4.62535066 5.44925035
H 10.66400125 -2.29001847 4.60899005
H 9.85272807 -2.46214088 6.20477782
H 8.90496950 -2.02203256 4.74802233
C 11.66099870 -0.84005537 1.57596549
C 12.83809057 -1.60828286 1.00175950
O 13.70934451 -1.08101322 0.30677043
H 11.17400519 -1.45698088 2.33201806
H 10.95099561 -0.57896790 0.79101628
H 12.03218453 0.09727264 2.01661700
N 12.84975025 -2.92427093 1.35401609
C 13.89736917 -3.82592764 0.92636670
C 13.36658106 -4.80523874 -0.12637054
O 12.30629173 -5.42681027 0.04049965
C 14.48983644 -4.62355866 2.10219034
C 15.06827226 -3.72523733 3.19718728
C 14.10058731 -3.34162095 4.31447785
O 12.93502897 -3.72723872 4.37225555
N 14.65117375 -2.51708588 5.25634229
H 12.07322037 -3.30640115 1.89733251
H 14.69407833 -3.20938846 0.48975169
H 15.29303040 -5.25276255 1.69186758
H 13.71956064 -5.28168495 2.52897506
H 15.46056914 -2.78931102 2.76491092
H 15.92838119 -4.21980765 3.67759906
H 14.12441511 -2.39174887 6.11467873
H 15.66401461 -2.39539555 5.29195834
N 14.14577725 -4.96078023 -1.21682845
C 13.90706502 -6.01798298 -2.19610388
H 15.05055107 -4.49537236 -1.21033743
H 13.09000281 -6.62896355 -1.81294032
H 13.66362888 -5.61866999 -3.19201061
H 14.81298054 -6.62203302 -2.23100011
O 17.85946132 3.58689550 10.68128280
H 18.49868902 4.31633509 10.67771620
H 17.51581584 3.57365268 9.77151921
N 20.65037515 0.69904179 5.60941383
C 20.88521692 -0.53799531 5.08601306
O 22.04105275 -0.90591060 4.79289557
N 19.82674908 -1.42137828 4.86228105
C 18.51074617 -1.15049275 5.15003033
O 17.58581667 -1.94795616 4.90925887
C 18.30777886 0.14744754 5.75490329
N 17.04597661 0.51886533 6.11033138
C 16.78780839 1.73647014 6.69823344
C 15.49272750 2.07923743 7.11453141
C 15.21950281 3.30356855 7.71607105
C 13.82591209 3.64126725 8.17515475
C 16.28553784 4.22348951 7.89547373
C 16.03698620 5.56198024 8.54424832
C 17.57969886 3.87320484 7.48812150
C 17.87101501 2.63247231 6.89241201
N 19.16715689 2.26934270 6.49673660
C 19.41370040 1.01810840 5.95156434
C 20.28558592 3.21454397 6.68958223
C 20.85345264 3.21127528 8.11840848
H 20.05675089 -2.35926104 4.47915108
H 14.69204661 1.35173721 6.96662827
H 13.80319467 3.83865963 9.25987874
H 13.45125074 4.55242747 7.68004879
H 13.12327430 2.82375029 7.96329949
H 16.95531860 6.16405927 8.59403390
H 15.27969551 6.13963261 7.98966225
H 15.65466167 5.43916973 9.57131876
H 18.37846297 4.59600698 7.64133911
H 19.92195262 4.20950556 6.39842055
H 21.05976503 2.92486825 5.97195989
H 21.49776557 2.34279432 8.25080324
H 21.45525835 4.12233686 8.26644000
H 20.05569073 3.19807381 8.86182801
H 16.29043596 -0.12731864 5.89132613

AsLOV2 **Qb** T_{1-H}

C 19.02899781 2.11200839 13.81289280
C 19.32946397 0.94339758 12.89901046
O 20.04883810 -0.00580083 13.24208306
H 18.15302960 2.65200961 13.45295178
H 18.86724886 1.74277433 14.83446805
H 19.89302191 2.77597480 13.82111934
N 18.78886263 1.03801520 11.65956521
C 18.92407392 -0.00536751 10.64973542
C 20.28323145 0.06996885 9.89321595
O 20.33418360 0.06750683 8.66206312
C 17.79663449 0.08776832 9.60951027
S 16.14668607 -0.18695831 10.33722488
H 18.21628829 1.85554930 11.42218932
H 18.89958312 -0.97951704 11.16775174
H 17.98718074 -0.66193709 8.83075382
H 17.82395803 1.08362040 9.14705571
N 21.35135080 0.12916728 10.71620263
C 22.71317443 0.02581859 10.23616224
H 21.16779874 -0.01809037 11.71241325
H 22.71793094 0.02804881 9.14600127
H 23.15290448 -0.93619535 10.55666722
H 23.37795001 0.80610003 10.60788650
C 25.51303384 -3.72099080 7.23808160
C 25.86052957 -2.22774812 7.25926578
C 24.69183299 -1.37630266 6.76284111
C 24.72404136 0.07631035 7.23725693
O 25.40043297 0.44783868 8.19849047
N 23.91461036 0.90924795 6.53706547
H 25.31959940 -4.08383500 6.21315269
H 26.76209133 -2.01903083 6.65750314
H 26.09957433 -1.91874697 8.28954467
H 23.73802761 -1.78634610 7.14641799
H 24.59737532 -1.40794495 5.66628086
H 23.77011216 1.84314074 6.90349277
H 23.25628851 0.53760642 5.84990801
H 26.30801254 -4.33201104 7.66596982
H 24.60697243 -3.84100151 7.83195325
C 23.25599921 -6.51499544 3.78394913
C 21.82668236 -5.95260719 3.94391578
C 21.70839680 -4.42739344 3.96586542
O 20.75916092 -3.84874511 3.39830461
N 22.62613588 -3.72126350 4.64808123
H 23.91600051 -5.85801543 4.35002159
H 21.40144955 -6.29209651 4.90626140
H 21.14577020 -6.30985131 3.15856125
H 22.54981948 -2.69439009 4.68695389
H 23.34562099 -4.17510156 5.19527111
H 23.34100598 -7.52799141 4.17702321
H 23.58392760 -6.49711475 2.73388243
C 19.24991968 -5.26294193 -1.04996186
C 19.37357089 -4.78254864 0.39353698
C 18.08349274 -4.13380004 0.90810359
O 17.07972457 -3.98997345 0.19588796
N 18.14155021 -3.75496028 2.20227669
H 18.37503300 -5.91104026 -1.09600896
H 20.19377361 -4.05646213 0.52560484
H 19.61763347 -5.62146976 1.06904762
H 17.41132085 -3.18507434 2.61147367
H 19.00817463 -3.86063125 2.72886142
H 20.14195282 -5.82836134 -1.35976198
H 19.12103814 -4.41301148 -1.72001991
C 6.29800414 -6.61100804 1.89500984
C 7.36222298 -6.33568130 2.94726510
O 7.09891840 -6.15808227 4.13707754
H 6.67275524 -7.25572378 1.08581752
H 6.01600648 -5.65100198 1.46299472
H 5.42299485 -7.06899158 2.35499791
N 8.64319626 -6.25397389 2.47933097
C 9.76100441 -6.00762797 3.38295452
C 9.93023084 -4.49494941 3.60544580
O 10.37726814 -3.76164988 2.71860491
C 11.07878699 -6.54149754 2.81195238
C 10.94686501 -7.96218102 2.28052554
O 10.58068404 -8.90396126 2.97965433
N 11.23866176 -8.07158397 0.95719735
H 8.83350496 -6.34684694 1.48774067
H 9.51711995 -6.50964853 4.32867629
H 11.83001670 -6.53387601 3.61589601
H 11.42841324 -5.86382543 2.02484934
H 11.24606331 -8.99771851 0.54336988
H 11.65066234 -7.28668991 0.45083404
N 9.61242816 -4.02556414 4.83704759
C 9.75199892 -2.59912814 5.11997460
H 9.00425591 -4.60154978 5.41232690
H 10.66397828 -2.28994756 4.60897590
H 9.84937044 -2.44770166 6.20373799
H 8.90503205 -2.02193450 4.74803381
C 11.66102336 -0.83998455 1.57601361
C 12.85911129 -1.59653486 1.03333093
O 13.78771629 -1.04809276 0.43962902
H 11.17399402 -1.45700460 2.33199143
H 10.95099529 -0.57901480 0.79100138
H 12.02270141 0.09570114 2.02656833
N 12.82485395 -2.93346496 1.30500159
C 13.90399087 -3.82389957 0.93196942
C 13.39577825 -4.82705616 -0.10806488
O 12.34978703 -5.46862437 0.08207450
C 14.47805537 -4.58650064 2.14124529
C 14.85604086 -3.66360546 3.31283542
C 13.73159159 -3.27108645 4.24036077
O 12.95699495 -4.34879681 4.56597055
N 13.60623099 -2.08137242 4.68897127
H 11.99304975 -3.33676309 1.73776450
H 14.70057276 -3.20115827 0.50580521
H 15.38167357 -5.10699933 1.79249749
H 13.76117551 -5.34770832 2.48059123
H 15.32366157 -2.73378540 2.96082300
H 15.60043709 -4.18279195 3.94076770
H 12.82975899 -2.02106607 5.36139819
N 14.15302222 -4.96411735 -1.21359150
C 13.90701269 -6.01802021 -2.19595797
H 15.05572542 -4.49375172 -1.21562845
H 13.08998842 -6.62899229 -1.81301677
H 13.66507931 -5.61310527 -3.18963378
H 14.81300446 -6.62199783 -2.23101069
O 16.82046323 2.98908814 10.91141516
H 16.98960659 3.26690564 9.99591423
H 16.22464446 2.22052212 10.79916571
N 20.76909707 0.71998892 5.57407543
C 21.05481336 -0.47853184 4.98696383
O 22.23248913 -0.82632333 4.76327162
N 20.02667542 -1.34855140 4.61948285
C 18.69565943 -1.15390276 4.91914211
O 17.79657268 -1.94822058 4.61306887
C 18.44707291 0.08224617 5.63240865
N 17.16868715 0.36277714 6.00626460
C 16.85029221 1.53713379 6.64921522
C 15.53860304 1.79451988 7.07511204
C 15.20066394 2.98217338 7.71913150
C 13.79088744 3.22919307 8.18634706
C 16.21472758 3.95187350 7.93207427
C 15.88263001 5.26659170 8.59442358
C 17.52949269 3.67874974 7.53093506
C 17.88706284 2.47827843 6.89052486
N 19.20398847 2.19800995 6.49052581
C 19.51182464 0.98268413 5.89584885
C 20.27029227 3.20152416 6.69532624
C 20.85324065 3.21113858 8.11815676
H 20.28888105 -2.21306732 4.11345065
H 14.77502165 1.03774793 6.88140380
H 13.75872913 3.37628555 9.27904871
H 13.37092338 4.14171791 7.73218942
H 13.13064563 2.38744890 7.93601723
H 16.76788328 5.91272246 8.67554762
H 15.11245661 5.81262202 8.02526466
H 15.48099686 5.11079988 9.60918949
H 18.28931555 4.43235953 7.72679240
H 19.84801491 4.17978803 6.42585077
H 21.05296992 2.96825419 5.96607237
H 21.49783378 2.34288573 8.25094770
H 21.44945259 4.12673379 8.25711733
H 20.05584954 3.19803145 8.86188913
H 12.23826763 -4.07865440 5.16242280
H 16.45516154 -0.32001638 5.74799588

AsLOV2 **Qc** T_{1-H}

C 19.02905692 2.11198142 13.81294979
C 19.32145757 0.95797380 12.88330041
O 20.02801122 -0.00516768 13.21048710
H 18.15299626 2.65197227 13.45297437
H 18.87148804 1.73329054 14.83152826
H 19.89299471 2.77601145 13.82104493
N 18.79372090 1.08566759 11.63913828
C 18.91431833 0.04891308 10.62166982
C 20.28526211 0.10819685 9.88194829
O 20.34934140 0.12954278 8.65101385
C 17.81017941 0.16866687 9.56631253
S 16.09635634 -0.00372466 10.15482145
H 18.17022346 1.87338588 11.46396834
H 18.86684017 -0.92781114 11.13320472
H 18.00944000 -0.55076127 8.76467260
H 17.87484313 1.17683307 9.12584380
N 21.34972279 0.13098796 10.71197090
C 22.71300557 0.02616154 10.23605841
H 21.15828255 -0.02705247 11.70446828
H 22.71799185 0.02788351 9.14600127
H 23.15025748 -0.93652532 10.55774463
H 23.37803193 0.80598698 10.60797455

C 25.51300214 -3.72099401 7.23808080
C 25.83714088 -2.22411284 7.21218096
C 24.65829484 -1.41472759 6.66556933
C 24.66652112 0.05971650 7.06776622
O 25.27056361 0.47021153 8.06130200
N 23.92949929 0.86552777 6.26296763
H 25.30184645 -4.10902466 6.22553676
H 26.74250939 -2.02024260 6.61405509
H 26.05417590 -1.86962808 8.23250968
H 23.70894176 -1.82046294 7.06449812
H 24.57376783 -1.50490875 5.57091905
H 23.74644090 1.80655120 6.59400705
H 23.28884879 0.46009536 5.57512944
H 26.30802619 -4.33200333 7.66595522
H 24.60698121 -3.84100017 7.83196749
C 23.25602768 -6.51499613 3.78402454
C 21.83230138 -5.94702352 3.89863912
C 21.67445500 -4.42627998 3.83830436
O 20.55983001 -3.93386892 3.56718963
N 22.71595017 -3.63325703 4.13390485
H 23.91599432 -5.85798733 4.34999478
H 21.38870759 -6.22839606 4.87113602
H 21.15680556 -6.35765406 3.13299495
H 22.57322102 -2.61452864 4.19596066
H 23.61978629 -4.00991719 4.38455806
H 23.34098762 -7.52800807 4.17698423
H 23.60659501 -6.51081716 2.73995937
C 19.25003372 -5.26305493 -1.05004727
C 19.34710462 -4.76437965 0.39222758
C 18.04743228 -4.10176406 0.86738000
O 17.07763686 -3.92800682 0.11685334
N 18.05574639 -3.74035695 2.16888540
H 18.37498749 -5.91098189 -1.09598299
H 20.16335666 -4.03351915 0.52631440
H 19.57778880 -5.59209233 1.08601002
H 17.32408476 -3.14903664 2.54472048
H 18.88643391 -3.88203496 2.74286975
H 20.14823669 -5.82623537 -1.34381402
H 19.12099443 -4.41298323 -1.71997663
C 6.29798662 -6.61098116 1.89500538
C 7.35434253 -6.34253091 2.95662751
O 7.07751350 -6.16544815 4.14342822
H 6.67543600 -7.25568706 1.08712340
H 6.01599066 -5.65100129 1.46300559
H 5.42300543 -7.06902090 2.35499070
N 8.64213510 -6.27439029 2.50514389
C 9.74744530 -6.03283601 3.42536430
C 9.94068358 -4.51857107 3.62616793
O 10.42285798 -3.81661992 2.73174394
C 11.06571885 -6.60583231 2.89255068
C 10.91212860 -8.02560397 2.36172423
O 10.50012976 -8.95212899 3.05566664
N 11.24117424 -8.15099718 1.04935478
H 8.84266724 -6.35175035 1.51426323
H 9.47253713 -6.51541820 4.37236711
H 11.78550781 -6.63085240 3.72503113
H 11.46535108 -5.93934902 2.11941145
H 11.23333654 -9.07996874 0.64188056
H 11.69703350 -7.38468867 0.55239303
N 9.59422245 -4.02521589 4.83763978
C 9.75199035 -2.59891828 5.11998588
H 8.96802949 -4.59014466 5.40417222
H 10.66403326 -2.29001745 4.60909628
H 9.85209523 -2.44875365 6.20356759
H 8.90498231 -2.02206724 4.74793939
C 11.66099240 -0.83999141 1.57600783
C 12.86566579 -1.58907958 1.04283831
O 13.83156630 -1.02747793 0.52368564
H 11.17399990 -1.45700907 2.33199225
H 10.95100413 -0.57899406 0.79100057
H 12.01850789 0.09327407 2.03320272
N 12.79115954 -2.94086680 1.21602394
C 13.90984754 -3.81283663 0.92014870
C 13.44621419 -4.88182014 -0.06993165
O 12.47715581 -5.61270442 0.19207819
C 14.48858267 -4.48402165 2.18057451
C 14.76215598 -3.47660021 3.31779888
C 13.54730694 -3.16345554 4.18613890
O 13.05101060 -2.04179562 4.27407707
N 13.05705454 -4.22579634 4.88767160
H 11.97555997 -3.34147121 1.68085541
H 14.69352746 -3.18732379 0.47492079
H 15.43044953 -4.97078899 1.88724324
H 13.80064621 -5.27428312 2.51995950
H 15.11949551 -2.51908857 2.91416182
H 15.55222322 -3.87457762 3.97573247
H 12.19491315 -4.12425735 5.41069926
H 13.46337811 -5.14902661 4.81327454
N 14.13885392 -4.95610775 -1.22249728
C 13.90699353 -6.01799757 -2.19602782
H 15.00340847 -4.42238044 -1.27487112
H 13.09000800 -6.62900700 -1.81299720
H 13.66717098 -5.61729064 -3.19172812
H 14.81300039 -6.62200429 -2.23099011
O 16.35716071 2.86378889 11.64710447
H 15.88382696 3.00560793 12.48165281
H 16.05257200 1.97782538 11.34964166
N 20.72765593 0.74407093 5.46554536
C 20.99034714 -0.44781658 4.85240709
O 22.15696511 -0.78247055 4.55296200
N 19.95165655 -1.32656970 4.54280443
C 18.63145462 -1.12944993 4.88529350
O 17.72374016 -1.92913591 4.61608677
C 18.40790734 0.10730084 5.60276864
N 17.14737591 0.37417791 6.04170148
C 16.86775675 1.50016041 6.78051124
C 15.61088924 1.68433688 7.37588507
C 15.34189019 2.79493868 8.18005525
C 14.01238913 2.94684837 8.86837965
C 16.35284771 3.77812961 8.34144982
C 16.10016060 5.00387681 9.17766306
C 17.60231034 3.58506717 7.74909605
C 17.91155723 2.43933643 6.99149368
N 19.19821330 2.19843883 6.48668134
C 19.48369569 0.99930872 5.84513970
C 20.26655691 3.20065388 6.69462355
C 20.85330124 3.21119676 8.11820602
H 20.19587497 -2.22323134 4.08531909
H 14.84179234 0.92717447 7.21146747
H 14.16040360 3.05500823 9.95494527
H 13.48304254 3.85108006 8.52421891
H 13.36267986 2.07928920 8.68864671
H 16.93440684 5.71624498 9.10681014
H 15.17849025 5.52254764 8.86848554
H 15.98407218 4.70767881 10.23213827
H 18.36105267 4.34690927 7.90917074
H 19.84043730 4.17950923 6.42972350
H 21.04655794 2.96898490 5.96219720
H 21.49785183 2.34285641 8.25094675
H 21.44760179 4.12861007 8.25309007
H 20.05581890 3.19795723 8.86185511
H 16.43259080 -0.32101577 5.82982552

AsLOV2 **Qd** $T_{1\text{-}H}$

C 19.02908079 2.11202760 13.81285914
C 19.32202648 0.95679346 12.88473305
O 20.03298591 -0.00277480 13.21248406
H 18.15297514 2.65196765 13.45302025
H 18.87135432 1.73402417 14.83168934
H 19.89299778 2.77600613 13.82110013
N 18.79014144 1.07835038 11.64133469
C 18.91476072 0.03881462 10.62709309
C 20.28439072 0.10320071 9.88502646
O 20.34616967 0.12473862 8.65388665
C 17.80872576 0.14642805 9.57217602
S 16.09652118 -0.03624997 10.16093260
H 18.16260421 1.86251235 11.46495457
H 18.87394486 -0.93622582 11.14233509
H 18.01303872 -0.57453735 8.77316341
H 17.86474129 1.15322591 9.12695650
N 21.35003812 0.12902156 10.71306963
C 22.71305505 0.02593959 10.23613482
H 21.16039767 -0.02795448 11.70613974
H 22.71797195 0.02799537 9.14600133
H 23.15177405 -0.93609148 10.55761471
H 23.37800081 0.80604395 10.60791280
C 25.51300447 -3.72100197 7.23803831
C 25.85530685 -2.22672247 7.25078899
C 24.68324862 -1.38285173 6.74776220
C 24.70750801 0.07185205 7.21689106
O 25.37191963 0.44704295 8.18519362
N 23.90477031 0.90068525 6.50506009
H 25.31829127 -4.08839738 6.21472836
H 26.75645522 -2.01804937 6.64841018
H 26.09160233 -1.90988292 8.27926196
H 23.73099368 -1.79581763 7.13234362
H 24.59155707 -1.41978938 5.65095583
H 23.75415159 1.83572696 6.86599178
H 23.25923958 0.52822731 5.80609129
H 26.30801233 -4.33200725 7.66597421
H 24.60699661 -3.84099099 7.83199188
C 23.25602662 -6.51500687 3.78385260
C 21.82732108 -5.94914658 3.94046805
C 21.70879294 -4.42297667 3.94237887
O 20.75943763 -3.85174310 3.36825705
N 22.62779824 -3.70775055 4.61434879
H 23.91599109 -5.85803035 4.35004861
H 21.40456788 -6.27615505 4.90822594
H 21.14423405 -6.31607634 3.16150142
H 22.55132176 -2.68099601 4.63919797
H 23.34792164 -4.15375752 5.16717484
H 23.34099725 -7.52796860 4.17708617
H 23.58542565 -6.49818872 2.73414011
C 19.24994773 -5.26297851 -1.04998482
C 19.36968308 -4.77381889 0.38996099
C 18.09095885 -4.08407901 0.87721036
O 17.10200415 -3.92096938 0.14884132
N 18.14178093 -3.68867108 2.16639020
H 18.37507555 -5.91103428 -1.09602321
H 20.20702908 -4.06823563 0.52532910
H 19.58244006 -5.61123261 1.07766440
H 17.42081101 -3.09656454 2.55943686
H 18.99507601 -3.81705594 2.70834358
H 20.14249819 -5.82949225 -1.35611720
H 19.12102015 -4.41300368 -1.72000767
C 6.29799975 -6.61100121 1.89499881
C 7.36267987 -6.34225818 2.94974164
O 7.09656245 -6.17422672 4.14051669
H 6.67145876 -7.25576972 1.08511374
H 6.01599971 -5.65100034 1.46300159
H 5.42300115 -7.06900153 2.35499959
N 8.64326405 -6.26098200 2.48335556
C 9.76365987 -5.99938450 3.38121266
C 9.92148979 -4.48582002 3.60040800
O 10.36086270 -3.74909176 2.71008679
C 11.08090434 -6.51051823 2.80154623
C 11.02275744 -7.96781750 2.37216472
O 10.58658574 -8.86318186 3.09348785
N 11.49626524 -8.17697243 1.11569989
H 8.82801766 -6.32531974 1.48865036
H 9.53316973 -6.50502987 4.32819700
H 11.85826481 -6.39721344 3.57405365
H 11.37729868 -5.86882155 1.96398311
H 11.56004285 -9.12869369 0.77206025
H 11.88595714 -7.40730894 0.57234448
N 9.59626290 -4.01815656 4.82649652
C 9.75200215 -2.59900731 5.11999429
H 9.01911108 -4.60578283 5.41988583
H 10.66400157 -2.28999942 4.60900062
H 9.84797093 -2.45352369 6.20476232
H 8.90500178 -2.02199816 4.74800394
C 11.66100997 -0.84000574 1.57601394
C 12.86025839 -1.59734915 1.03395200
O 13.78572182 -1.04395408 0.43899989
H 11.17399503 -1.45700138 2.33199456
H 10.95100476 -0.57899972 0.79099840
H 12.02264854 0.09746986 2.02333025
N 12.83470865 -2.93466493 1.30789944
C 13.91817595 -3.81685160 0.91977131
C 13.41041619 -4.83395618 -0.10452183
O 12.38556915 -5.50238445 0.10341986
C 14.53390675 -4.55844547 2.12286156
C 14.88138543 -3.59910743 3.27115962
C 13.74519738 -3.35945127 4.23899669
O 13.67638814 -2.04386485 4.61207419
N 13.00428816 -4.30810765 4.65711856
H 12.00933238 -3.34341364 1.75022656
H 14.69598183 -3.18463800 0.47316640
H 15.44807262 -5.05196290 1.76377792
H 13.84436218 -5.33317759 2.48761826
H 15.22344259 -2.62738224 2.88544378
H 15.71314137 -4.01396091 3.86350226
H 12.28874141 -3.98827146 5.32035690
N 14.15071950 -4.95640649 -1.22373257
C 13.90699750 -6.01800614 -2.19599673
H 15.04398398 -4.46912762 -1.24367657
H 13.08999953 -6.62900292 -1.81300927
H 13.66506121 -5.61974787 -3.19254257
H 14.81300229 -6.62200159 -2.23099982
O 16.33533416 2.83185216 11.65682212
H 15.87149268 2.96823667 12.49760756
H 16.04088454 1.94064089 11.36540061
N 20.76811492 0.74278108 5.51527749
C 21.05438066 -0.45047849 4.91600855
O 22.23185835 -0.79768834 4.69050909
N 20.02565490 -1.31805460 4.54206258
C 18.69743884 -1.13473002 4.86168178
O 17.79837633 -1.93359007 4.56456682
C 18.45116287 0.08960301 5.59308977
N 17.18093364 0.34146391 6.01318385
C 16.88178074 1.45865461 6.75913062
C 15.61896560 1.62393728 7.34685763
C 15.32904626 2.72648669 8.15501289
C 13.99228992 2.85809659 8.83380734
C 16.32549739 3.72212386 8.32934240
C 16.05061468 4.94027516 9.16972588
C 17.58206343 3.54776196 7.74617791
C 17.91141901 2.41084068 6.98425787
N 19.20432863 2.18990598 6.48768106
C 19.51346784 0.99099520 5.85909305
C 20.25985393 3.20502413 6.69851903
C 20.85328129 3.21115595 8.11823089
H 20.29023426 -2.18545045 4.04295093
H 14.86201569 0.85620105 7.17473209
H 14.12948775 2.96964044 9.92139560
H 13.45104473 3.75336226 8.48466328
H 13.35756128 1.98005363 8.65080435
H 16.87442624 5.66524944 9.10545995
H 15.12274953 5.44664261 8.85871132
H 15.93500073 4.63812407 10.22258619
H 18.33059375 4.31746405 7.91705929
H 19.81827938 4.17995308 6.44549208
H 21.03876987 2.99214118 5.95927598
H 21.49785088 2.34287636 8.25092854
H 21.44716898 4.12882446 8.25335355
H 20.05582049 3.19802400 8.86185802
H 12.98656898 -1.94460392 5.28997657
H 16.47569081 -0.35780606 5.78159335

AsLOV2 **Qa** S_0'

C 5.51900438 -6.15600101 1.90999832
C 6.63037349 -5.97617425 2.93725172
O 6.41029703 -5.91322978 4.14650171
H 5.85396320 -6.71152192 1.02131968
H 5.18999864 -5.16600058 1.59400125
H 4.68499996 -6.67599952 2.38200056
N 7.89133514 -5.87128640 2.42283092
C 9.07268710 -5.76323153 3.27186679
C 9.48466963 -4.28517502 3.43659119
O 10.11483385 -3.69318288 2.55270851
C 10.22812488 -6.57143235 2.68934274
C 11.32238030 -6.80177998 3.73192432
O 11.06321770 -6.94256695 4.92693586
N 12.57321297 -6.89019455 3.21818432
H 8.02198017 -5.84260410 1.41800151
H 8.80111048 -6.17956760 4.24959463
H 10.63671211 -6.07604254 1.79754096
H 9.84740228 -7.56324175 2.39345398
H 13.34210603 -6.98202961 3.87329518
H 12.75837510 -6.54389624 2.27224398
N 9.10168104 -3.70965699 4.59541115
C 9.35900745 -2.30799658 4.90300893
H 8.47034861 -4.23811289 5.18883285
H 10.27627604 -2.00352368 4.38304221
H 8.52600059 -1.68400265 4.57999544
H 9.52299725 -2.18499880 5.97400082
C 18.97700054 2.19999730 13.59599664
C 19.10810335 0.94381518 12.74079361
O 19.51626602 -0.12751646 13.17898608
H 18.10700181 2.75800339 13.24900475
H 18.79699994 1.84600067 14.61100100
H 19.88482221 2.81914441 13.55373433
N 18.71862175 1.09139894 11.43790149
C 18.74186698 -0.03992900 10.52156298
C 20.16236742 -0.58975490 10.29914142
O 20.35616880 -1.75773941 9.97489166
C 18.14135552 0.44237518 9.18096089
S 17.90952869 -0.93619127 7.97628518
H 18.35805604 1.98992780 11.10986192
H 18.15677818 -0.87796607 10.93233020
H 18.81436865 1.19382893 8.75036302
H 17.16492396 0.91233150 9.36696490
N 21.14971188 0.32782096 10.41281086
C 22.50100912 0.02699568 9.97900411
H 20.89748659 1.27545430 10.66266206
H 22.49399890 -0.06799640 8.89300036
H 23.16600082 0.83900136 10.27399904
H 22.84775706 -0.91821241 10.41995386
C 25.39799314 -3.82699874 7.28699662
C 25.77002253 -2.34039968 7.33860067
C 24.60654078 -1.46691736 6.87458181
C 24.71893713 0.00491947 7.26484463
O 25.52064495 0.41670640 8.10243495
N 23.83517990 0.81975564 6.62558285
H 25.19600889 -4.16200426 6.27000034
H 26.66472050 -2.13380738 6.72630078
H 26.03271109 -2.05581490 8.36995670
H 23.66259017 -1.82526557 7.32593132
H 24.45169262 -1.54622287 5.78666932
H 23.75161424 1.77293758 6.96057888
H 23.06541491 0.41328275 6.09403002
H 26.19683869 -4.46714416 7.69295590
H 24.50300291 -3.96499680 7.89300305
C 23.10296988 -6.60201875 3.93897450
C 21.68701248 -6.06205321 4.20076441
C 21.54267425 -4.55684295 4.38060566
O 20.55214911 -3.94921299 3.91942471
N 22.47486226 -3.90791416 5.09183835
H 23.86113520 -6.02540896 4.49533231
H 21.29239675 -6.49832800 5.13666560
H 20.98713249 -6.35522868 3.40553331
H 22.35786006 -2.90142668 5.26007117
H 23.21818124 -4.40367810 5.56566828
H 23.39401568 -6.55399215 2.89000448

H	23.20701550	-7.62299222	4.30501966
C	19.07997065	-5.21997185	-0.85197806
C	19.26649796	-4.73159683	0.58058679
C	18.03136122	-4.00247498	1.11940023
O	17.03305135	-3.77642965	0.42673961
N	18.14052694	-3.63237880	2.41400909
H	18.19301053	-5.85401316	-0.88000367
H	20.12767339	-4.04669789	0.67282837
H	19.48672893	-5.57430890	1.26004686
H	17.41872525	-3.07000039	2.84482712
H	18.96651679	-3.86739559	2.96304045
H	18.93901799	-4.37400854	-1.52501394
H	19.95110478	-5.80183654	-1.19021164
C	11.32700414	-0.63299703	1.43000718
C	12.57482235	-1.36188667	0.95499377
O	13.54411298	-0.77122127	0.47197448
H	10.81299576	-1.23900554	2.17599228
H	11.64350341	0.32122998	1.87482405
H	10.64400221	-0.40799862	0.61099924
N	12.53003395	-2.71604689	1.11744711
C	13.72617679	-3.53673090	0.99601445
C	13.38951025	-4.78196142	0.16581699
O	12.68078957	-5.69584192	0.61954917
C	14.27646101	-3.96101279	2.36633876
C	14.83324422	-2.80878098	3.21915833
C	13.86789755	-2.21098123	4.23474712
O	12.66249596	-2.10240554	4.04945355
N	14.47317260	-1.74024265	5.38385912
H	11.76750605	-3.10574532	1.67483637
H	14.48497087	-2.93511204	0.47892339
H	15.09715788	-4.67044043	2.17810070
H	13.49117875	-4.49708595	2.91749320
H	15.16935247	-1.97881009	2.57354559
H	15.71928866	-3.15764357	3.76354919
H	13.84339770	-1.51940726	6.15097319
H	15.37684203	-2.13197517	5.64193617
N	13.88839290	-4.81453178	-1.08671573
C	13.70800389	-5.95800437	-1.97500691
H	14.55783932	-4.09784977	-1.34525567
H	12.91500072	-6.57699729	-1.55699580
H	13.43823446	-5.62257055	-2.98707572
H	14.62800023	-6.54200168	-2.01400133
N	20.44553207	0.53978833	6.00674133
C	20.73356293	-0.71564944	5.55218479
O	21.90298549	-1.09678085	5.42471949
N	19.70490840	-1.57774475	5.15361376
C	18.38521839	-1.35947948	5.38686130
O	17.49298041	-2.11378044	5.01507195
N	16.87085810	0.49629235	5.70870802
C	16.51494518	1.71842757	6.26518367
C	15.18131061	2.11979857	6.41690217
C	14.84696124	3.36421121	6.95540347
C	13.40262406	3.76693750	7.10748361
C	15.88427427	4.23949702	7.36079976
C	15.56701484	5.59170867	7.95144608
C	17.21633623	3.83954878	7.20012604
C	17.54878009	2.58961459	6.65857028
N	18.89190301	2.17472676	6.45747885
C	19.99614067	3.16222565	6.51351570
C	20.62397656	3.25395417	7.90800126
C	18.05276099	-0.11167417	6.21214354
C	19.19349431	0.87985396	6.20640779
H	19.99543925	-2.45584714	4.67032125
H	14.39424079	1.43057167	6.10223121
H	13.15116512	3.96947917	8.16231416
H	13.18737137	4.69413008	6.55033167
H	12.72464053	2.98422262	6.73981608
H	16.48249174	6.13208501	8.23460856
H	15.01182815	6.22194571	7.23648189
H	14.93187302	5.50373767	8.84889534
H	18.00457114	4.52598550	7.50490330
H	19.57629589	4.12440355	6.19762313
H	20.73692537	2.85791569	5.76619270
H	21.29302663	2.41602017	8.10100046
H	16.11114346	-0.14722585	5.48430993
H	21.21587132	4.17996979	7.98255673
H	19.84000137	3.28301819	8.66499985
O	17.84946490	3.54834818	10.23724795
H	17.90092754	4.47553110	10.51387858
H	17.18215910	3.54432601	9.53069318

AsLOV2 **Qb** S_0'

C	5.51900379	-6.15600175	1.90999691
C	6.63648401	-5.96465100	2.92978853
O	6.42470949	-5.88798618	4.13960809
H	5.85247934	-6.71156950	1.02080121
H	5.18999928	-5.16600039	1.59400120
H	4.68500017	-6.67599878	2.38200172
N	7.89556036	-5.85521233	2.40779947
C	9.07987465	-5.75064304	3.25282975
C	9.49883454	-4.27723697	3.43315891
O	10.15263658	-3.67892079	2.57077631
C	10.23915076	-6.54977599	2.66773550
C	11.34888049	-6.73820595	3.70267650
O	11.11207690	-6.78499459	4.90915929
N	12.58560836	-6.90882328	3.17118984
H	8.02383213	-5.85331914	1.40228163
H	8.81203212	-6.16937957	4.23046358
H	10.63363102	-6.06011981	1.76652408
H	9.87379351	-7.55202563	2.38855813
H	13.36262186	-6.95403941	3.82234007
H	12.76375726	-6.57411306	2.22069450
N	9.10582709	-3.70738872	4.59271243
C	9.35900803	-2.30799886	4.90300025
H	8.45910799	-4.23101899	5.17436347
H	10.25624901	-1.98966913	4.35581329
H	8.52600017	-1.68400112	4.57999957
H	9.52299694	-2.18500045	5.97400103
C	18.97699530	2.19998854	13.59598601
C	19.10784580	0.94292768	12.73815058
O	19.52348278	-0.12499140	13.17815734
H	18.10700282	2.75800862	13.24901068
H	18.79700158	1.84600292	14.61100205
H	19.88511886	2.81859643	13.55314075
N	18.71217087	1.08252413	11.43533900
C	18.74345954	-0.05407510	10.52193674
C	20.16717653	-0.60170707	10.31663332
O	20.36878121	-1.77502162	10.01840104
C	18.15445732	0.41565832	9.16980637
S	17.94260946	-0.97027722	7.96488707
H	18.34330866	1.97574622	11.10230321
H	18.15664531	-0.89098772	10.93233078
H	18.82701123	1.17048440	8.74391129
H	17.17333296	0.88069616	9.34430791
N	21.14955800	0.32415631	10.41459752
C	22.50101071	0.02699720	9.97900157
H	20.88908357	1.27666950	10.63610948
H	22.49399969	-0.06799613	8.89300031
H	23.16600130	0.83900024	10.27400105
H	22.84869820	-0.91792962	10.41953004
C	25.39799383	-3.82699914	7.28699763
C	25.77878687	-2.34307092	7.34385233
C	24.62228190	-1.46106566	6.88028020
C	24.73881324	0.00801351	7.27937197
O	25.54061864	0.41261647	8.12066167
N	23.85844724	0.82850840	6.64283360
H	25.19601069	-4.16200548	6.27000040
H	26.67635906	-2.14077128	6.73425988
H	26.04125005	-2.06392949	8.37679991
H	23.67452306	-1.81731506	7.32485846
H	24.47116607	-1.53356846	5.79157855
H	23.77409829	1.77863232	6.98632806
H	23.08679214	0.42365751	6.11161955
H	26.19366442	-4.47161666	7.69273667
H	24.50300074	-3.96499424	7.89300046
C	23.10292771	-6.60205388	3.93894329
C	21.69259647	-6.05446725	4.20708651
C	21.56848678	-4.54536037	4.36302197
O	20.61141188	-3.92440293	3.85338255
N	22.48597752	-3.90476682	5.10176177
H	23.86377341	-6.02699792	4.49340719
H	21.30700226	-6.47523724	5.15362986
H	20.98408930	-6.35350753	3.42194108
H	22.37998210	-2.89458358	5.25935931
H	23.19834784	-4.40955016	5.61251338
H	23.39405775	-6.55396236	2.89001751
H	23.20701333	-7.62298655	4.30503608
C	19.07994562	-5.21994884	-0.85196552
C	19.28708412	-4.73569743	0.57775666
C	18.06728568	-3.99314254	1.12750245
O	17.10809218	-3.67246893	0.41634160
N	18.13563575	-3.73775860	2.45256429
H	18.19302106	-5.85402792	-0.88000370
H	20.15365494	-4.05544924	0.65740865
H	19.51209716	-5.57920088	1.25379328
H	17.47163091	-3.10892355	2.88589359
H	18.97953366	-3.96093743	2.98011134
H	18.93902598	-4.37401296	-1.52502116
H	19.94581777	-5.80461425	-1.20015334
C	11.32700827	-0.63299522	1.43000245
C	12.56217920	-1.36494495	0.91577013
O	13.43364729	-0.79752804	0.25619061
H	10.81300015	-1.23900142	2.17599863
H	11.65451416	0.32088034	1.86871852
H	10.64399972	-0.40800538	0.61099943
N	12.62852733	-2.68238791	1.26977227
C	13.79735129	-3.50897802	1.00076558
C	13.38964503	-4.73212034	0.15574659
O	12.62446394	-5.59815982	0.60986434
C	14.45878290	-3.99461759	2.30156283
C	14.93751770	-2.87285500	3.23181680
C	13.93306641	-2.24116413	4.16903048
O	12.84570215	-3.03163410	4.37043693
N	14.17125105	-1.12202984	4.74593406
H	11.86802094	-3.09743191	1.81044874
H	14.51219148	-2.88418278	0.44944342
H	15.33699847	-4.58988488	2.01200876
H	13.76402005	-4.65287070	2.83821888
H	15.42945865	-2.06377945	2.67247427
H	15.70337942	-3.28717658	3.90589014
H	13.41940099	-0.87766595	5.40429155
N	13.90638805	-4.80795348	-1.08907317
C	13.70800436	-5.95800030	-1.97498521
H	14.60609298	-4.11953183	-1.34650873
H	12.91499940	-6.57700057	-1.55700317
H	13.43655342	-5.62224770	-2.98641083
H	14.62800018	-6.54200126	-2.01400963
N	20.46048535	0.53174014	6.01943508
C	20.76708546	-0.71728798	5.55764590
O	21.94411081	-1.08229978	5.43598599
N	19.75509890	-1.58488849	5.14458384
C	18.42415303	-1.37576957	5.34677771
O	17.54881750	-2.11659078	4.92734585
N	16.87887111	0.43942096	5.68329820
C	16.51507991	1.67172909	6.19983149
C	15.17808877	2.08740913	6.28337800
C	14.82585183	3.33464733	6.80158651
C	13.37782549	3.74645026	6.88050208
C	15.84557517	4.20544596	7.25736254
C	15.50573246	5.55576071	7.83872074
C	17.18175656	3.80145396	7.15140612
C	17.53447997	2.55002198	6.62493868
N	18.88739014	2.14877739	6.46104566
C	19.98365484	3.14859536	6.51745162
C	20.62397450	3.25394553	7.90800068
C	18.06878744	-0.14277749	6.18617364
C	19.20145070	0.85801925	6.20274816
H	20.05307845	-2.44300726	4.63457782
H	14.40767617	1.40510530	5.92008295
H	13.07680307	3.96145630	7.91977662
H	13.19339526	4.66854479	6.30381922
H	12.71402137	2.96289627	6.48900421
H	16.41073769	6.10209156	8.14282409
H	14.96375357	6.18303297	7.11112642
H	14.85027262	5.46429195	8.72147486
H	17.96012438	4.48680399	7.48471111
H	19.55243640	4.10639624	6.20357394
H	20.72363810	2.85161320	5.76608342
H	21.29303393	2.41602640	8.10100215
H	16.13143021	-0.20883624	5.40180183
H	21.21254046	4.18256011	7.97131160
H	19.83999930	3.28301802	8.66499773
O	17.81335411	3.51078877	10.18514939
H	17.82233567	4.44574804	10.43916394
H	17.18741029	3.47037496	9.44215024
H	12.28056863	-2.65167615	5.06308943

AsLOV2 **Qc** S_0'

C	5.51900242	-6.15600043	1.90999214
C	6.65431702	-5.99210212	2.91831474
O	6.46864369	-5.97668084	4.13405544
H	5.84122469	-6.70989195	1.01508349
H	5.18999537	-5.16600037	1.59400411
H	4.68500134	-6.67599947	2.38200217
N	7.90368873	-5.85336825	2.37586292
C	9.11056741	-5.71804678	3.18937485
C	9.46700533	-4.23227250	3.38923668
O	10.02884377	-3.58580942	2.49634227
C	10.26842570	-6.44316127	2.50660159
C	11.55387926	-6.39754889	3.31412697
O	11.69624572	-5.65846835	4.29356853
N	12.53204022	-7.24489354	2.89367349
H	8.00326412	-5.76845170	1.37030578
H	8.88698303	-6.17523941	4.16215349
H	10.50135206	-5.95205462	1.54867268
H	9.98637999	-7.48635791	2.29872883
H	13.45298221	-7.07842819	3.28996287
H	12.52969147	-7.49902017	1.90993498
N	9.11154575	-3.70278500	4.57626687
C	9.35901766	-2.30799617	4.90299835
H	8.53967094	-4.26584364	5.19629286
H	10.25960733	-1.98190561	4.36692492
H	8.52599763	-1.68400312	4.58000133
H	9.52299667	-2.18500116	5.97400145
C	18.97699239	2.19998616	13.59598897
C	19.11567064	0.93587046	12.74963791
O	19.55623234	-0.11806441	13.20017307
H	18.10700229	2.75800720	13.24900893
H	18.79700635	1.84600613	14.61100406
H	19.88548230	2.81806257	13.55396171
N	18.70006033	1.04805193	11.45114912
C	18.75423644	-0.10099987	10.55421962
C	20.18646700	-0.63558974	10.37954498
O	20.40897947	-1.81965626	10.14685072
C	18.18591103	0.34123936	9.18432233
S	18.07196562	-1.06042749	7.98191949
H	18.32106507	1.93294802	11.10639306
H	18.16960592	-0.93846268	10.96638551
H	18.84141480	1.11968960	8.77378488
H	17.18199538	0.76718283	9.32580094
N	21.15064149	0.31400684	10.42205469
C	22.50101362	0.02700000	9.97900474
H	20.86967689	1.27294233	10.58385524
H	22.49399561	-0.06800301	8.89300063
H	23.16599865	0.83900306	10.27399873
H	22.85195418	-0.91716895	10.41753869
C	25.39800283	-3.82699828	7.28700155
C	25.78146059	-2.34399057	7.34627162
C	24.63235013	-1.45781897	6.87191422
C	24.74530756	0.00860150	7.28304536
O	25.52678346	0.40328421	8.14819212
N	23.88572490	0.83696797	6.63024501
H	25.19600179	-4.16200130	6.27000045
H	26.68497005	-2.14436426	6.74454025
H	26.03536200	-2.06530744	8.38147133
H	23.67804929	-1.81540570	7.30146740
H	24.49701141	-1.52299004	5.78085705
H	23.80022351	1.78728126	6.97270878
H	23.13236552	0.44155020	6.06646211
H	26.19254283	-4.47307096	7.69280826
H	24.50300074	-3.96500028	7.89299956
C	23.10294067	-6.60201150	3.93895629
C	21.69884944	-6.04274978	4.20932117
C	21.59479493	-4.52806452	4.31551623
O	20.66403782	-3.90567555	3.76066090
N	22.50612632	-3.88130265	5.05701937
H	23.86761582	-6.03019600	4.49193720
H	21.32538587	-6.43187164	5.17396686
H	20.97899835	-6.36023803	3.44206953
H	22.42108414	-2.86403374	5.17898807
H	23.19611725	-4.38338641	5.60025166
H	23.39403668	-6.55399750	2.89001021
H	23.20701666	-7.62298856	4.30502831
C	19.07993837	-5.21994238	-0.85196706
C	19.27647528	-4.74448114	0.58144694
C	18.02390770	-4.07447909	1.15111700
O	17.02166139	-3.84116230	0.46371541
N	18.11492164	-3.78204133	2.46549129
H	18.19302333	-5.85403041	-0.88000006
H	20.11156439	-4.02759990	0.66903730
H	19.54312182	-5.58678436	1.24397494
H	17.41346370	-3.20744293	2.91367958
H	18.98114722	-3.95267875	2.97545687
H	18.93903239	-4.37401332	-1.52502411
H	19.94835745	-5.80108136	-1.19942927
C	11.32700282	-0.63301704	1.42999710
C	12.56597684	-1.36845903	0.94124915
O	13.46875402	-0.81115777	0.31606550
H	10.81300280	-1.23899609	2.17600416
H	11.64948222	0.32225819	1.86970732
H	10.64400375	-0.40799403	0.61099904
N	12.60057302	-2.68663513	1.29407350
C	13.74415908	-3.54326461	1.02701117
C	13.30085026	-4.71650394	0.13208891
O	12.41081852	-5.49239486	0.50134880
C	14.35949501	-4.07919763	2.33332291
C	14.90460150	-2.98317140	3.26386106
C	13.99722693	-2.24934370	4.24549046
O	14.34163141	-1.15462511	4.71526121
N	12.85369294	-2.86034052	4.63480777
H	11.79209231	-3.10078257	1.76135497
H	14.49391931	-2.92851081	0.51232826
H	15.20141199	-4.72814538	2.04888650
H	13.62249218	-4.71185881	2.84772694
H	15.41554102	-2.20060197	2.68331360
H	15.67300221	-3.42376972	3.92059329
H	12.34195372	-2.43104558	5.39744696
H	12.59200902	-3.81005258	4.37474515
N	13.92869811	-4.83280128	-1.06168911
C	13.70800897	-5.95800442	-1.97498014
H	14.73792908	-4.23987366	-1.21936424
H	12.91499495	-6.57699649	-1.55700611
H	13.43580972	-5.60767607	-2.98160435
H	14.62800007	-6.54200047	-2.01401214
N	20.49682294	0.52473920	5.98648871
C	20.82341357	-0.70649155	5.49350417
O	22.00576880	-1.04030179	5.33258132
N	19.82464539	-1.59569083	5.09217538
C	18.49644536	-1.43709555	5.34669924
O	17.63153161	-2.20503938	4.95466470
N	16.91695314	0.32650414	5.72914045
C	16.52437342	1.54330296	6.25763215
C	15.17325971	1.90061704	6.37420577

C	14.78814948	3.13829044	6.89163835
C	13.32782189	3.49374314	7.00906575
C	15.78639124	4.05294414	7.31020427
C	15.40967668	5.40042337	7.87622687
C	17.13514017	3.69781990	7.18266811
C	17.52377117	2.45522182	6.66078044
N	18.88903794	2.09446266	6.48363931
C	19.96099388	3.12593545	6.52605927
C	20.62399350	3.25399923	7.90796057
C	18.13098529	-0.22081619	6.20499447
C	19.23365351	0.81473221	6.20512575
H	20.12854876	-2.43469240	4.55462793
H	14.42684789	1.17221673	6.04631781
H	13.04705664	3.70085360	8.05577131
H	13.09133427	4.40551285	6.43495733
H	12.68618522	2.68237944	6.63830284
H	16.30014668	5.97988643	8.16140557
H	14.84033054	5.99871154	7.14494402
H	14.76666875	5.30266878	8.76727854
H	17.89358426	4.41408330	7.49662575
H	19.49845799	4.07166180	6.21987927
H	20.69547407	2.84883328	5.76187345
H	21.29300753	2.41601117	8.10102761
H	16.19058953	-0.33992873	5.44307670
H	21.20717392	4.18631081	7.95334715
H	19.84000740	3.28298023	8.66500762
O	17.80957663	3.46336954	10.18793932
H	17.75075360	4.38643851	10.47669113
H	17.16313534	3.39679607	9.46409877

AsLOV2 **Qd** $S_0^{'}$

C	5.51899781	-6.15600054	1.91000413
C	6.63784039	-5.97913288	2.92937738
O	6.42588167	-5.91704965	4.13988949
H	5.84885209	-6.71107128	1.01891241
H	5.18999886	-5.16600225	1.59399700
H	4.68500049	-6.67600090	2.38199931
N	7.89688224	-5.87645625	2.40663860
C	9.07818107	-5.74494449	3.25185763
C	9.46520581	-4.26104223	3.41001033
O	10.04205261	-3.64299449	2.50768842
C	10.25301859	-6.53359004	2.68299202
C	11.33924137	-6.73619693	3.74181660
O	11.07208576	-6.81294065	4.94033207
N	12.58939685	-6.88927986	3.23804086
H	8.02106511	-5.83720628	1.40134478
H	8.81358811	-6.15965636	4.23187497
H	10.66503515	-6.03418827	1.79484911
H	9.89597948	-7.53315618	2.38354297
H	13.34974348	-6.92864075	3.90882032
H	12.78836326	-6.54207667	2.29578083
N	9.12073041	-3.70758317	4.59230202
C	9.35903295	-2.30798012	4.90299534
H	8.53575190	-4.25720009	5.21258063
H	10.25856765	-1.98379668	4.36399766
H	8.52599318	-1.68400916	4.58000235
H	9.52299011	-2.18500779	5.97400330
C	18.97699339	2.19999075	13.59597521
C	19.11272810	0.93938113	12.74644596
O	19.54191249	-0.12116073	13.19135708
H	18.10700112	2.75800067	13.24900300
H	18.79700428	1.84599670	14.61100031
H	19.88530339	2.81835910	13.55402114
N	18.70569787	1.06477279	11.44623122
C	18.75102746	-0.07839330	10.54355188
C	20.17907439	-0.62076989	10.35727403
O	20.39047880	-1.80100892	10.09649394
C	18.18012956	0.37781067	9.18044939
S	18.04510088	-1.01888301	7.97717822
H	18.33329536	1.95438348	11.10731975
H	18.16303433	-0.91521821	10.95246222
H	18.84237319	1.14966898	8.76887962
H	17.18201554	0.81469631	9.32916952
N	21.15101980	0.31875463	10.42029814
C	22.50101806	0.02698065	9.97901527
H	20.88058218	1.27487361	10.61285447
H	22.49400297	-0.06799876	8.89300010
H	23.16599188	0.83901315	10.27398730
H	22.85109887	-0.91746561	10.41805379
C	25.39794594	-3.82694865	7.28696117
C	25.78044663	-2.34368799	7.34531688
C	24.62935546	-1.45857477	6.87359039
C	24.74351244	0.00818927	7.28248121
O	25.52764586	0.40443400	8.14417037
N	23.88192895	0.83671029	6.63135871
H	25.19602936	-4.16203634	6.27000638
H	26.68242010	-2.14309722	6.74164110
H	26.03632786	-2.06473771	8.37995476
H	23.67662993	-1.81616426	7.30664455
H	24.49030186	-1.52524697	5.78300207
H	23.79767981	1.78682942	6.97477691
H	23.12394409	0.44155142	6.07442876
H	26.19309688	-4.47242360	7.69240794
H	24.50302233	-3.96501949	7.89302639
C	23.10291760	-6.60201864	3.93897610
C	21.69307484	-6.05195947	4.20419421
C	21.56954333	-4.54021945	4.33343344
O	20.60780448	-3.92763173	3.82159689
N	22.49561786	-3.88836934	5.05177512
H	23.86452796	-6.02821889	4.49384306
H	21.31194773	-6.45648452	5.15961460
H	20.98140357	-6.36507143	3.42745296
H	22.40000130	-2.87474621	5.19038981
H	23.21170840	-4.38640732	5.56385797
H	23.39406478	-6.55398215	2.89001835
H	23.20701370	-7.62299068	4.30502402
C	19.07999399	-5.21993859	-0.85197624
C	19.27622836	-4.72354481	0.57558363
C	18.06235365	-3.94781191	1.09130366
O	17.12052784	-3.62324942	0.35979482
N	18.11362865	-3.66232342	2.41119847
H	18.19303915	-5.85405306	-0.88001860
H	20.15507615	-4.06003473	0.66086495
H	19.47217376	-5.56172867	1.26691307
H	17.43865756	-3.02748600	2.81733405
H	18.93408003	-3.90216499	2.96619101
H	18.93895465	-4.37398705	-1.52497377
H	19.94861415	-5.80460012	-1.19242354
C	11.32701033	-0.63298481	1.42999795
C	12.56672361	-1.36368055	0.92816654
O	13.47152500	-0.77990973	0.32765239
H	10.81300232	-1.23900362	2.17599878
H	11.65078415	0.32305431	1.86670989
H	10.64399152	-0.40800626	0.61100636
N	12.59978904	-2.69748996	1.21809757
C	13.79232040	-3.50586578	0.99133229
C	13.42130444	-4.75428208	0.17085303
O	12.71129706	-5.65399506	0.64747905
C	14.45694824	-3.92496844	2.31395249
C	14.81102386	-2.73959591	3.22148037
C	13.79224361	-2.39732905	4.27772698
O	14.19296034	-1.28460133	4.98743917
N	12.75129566	-3.09298886	4.50016548
H	11.84563071	-3.10674593	1.77307014
H	14.49536180	-2.87927652	0.42703344
H	15.37839696	-4.46599208	2.05539163
H	13.79123680	-4.60841063	2.85578368
H	15.03286306	-1.82934269	2.63941170
H	15.72667989	-2.95627475	3.79253866
H	12.20966497	-2.74348282	5.29947345
N	13.89827677	-4.80630747	-1.09148612
C	13.70801601	-5.95799680	-1.97504005
H	14.56202173	-4.09127885	-1.36968702
H	12.91500463	-6.57698760	-1.55697417
H	13.43699386	-5.62313635	-2.98672450
H	14.62799314	-6.54201333	-2.01398764
N	20.49268695	0.53573117	5.98976458
C	20.80626707	-0.70639061	5.51206305
O	21.98424737	-1.05530689	5.36241755
N	19.79700238	-1.58535252	5.11240624
C	18.47108872	-1.39819685	5.35272307
O	17.59205266	-2.14486468	4.95047745
N	16.90933479	0.38489237	5.74145668
C	16.53233415	1.61110556	6.26557986
C	15.19053528	2.00028742	6.37819905
C	14.82621828	3.24640136	6.89162422
C	13.37340892	3.63287401	7.00041153
C	15.84119146	4.14039102	7.31126147
C	15.49163285	5.49410473	7.87933266
C	17.18206824	3.75908694	7.17948321
C	17.54617532	2.50922941	6.65845201
N	18.90228425	2.12773263	6.47010676
C	19.98370929	3.14357422	6.51860962
C	20.62399889	3.25397406	7.90799581
C	18.12151206	-0.18027603	6.21785829
C	19.23437230	0.84251336	6.20488196
H	20.09229482	-2.43865137	4.58963532
H	14.42246610	1.30211357	6.03760528
H	13.09242919	3.85054925	8.04470304
H	13.15973244	4.54711628	6.42156010
H	12.71444603	2.83510979	6.63000561
H	16.39381740	6.05606060	8.16229046
H	14.93174437	6.10359691	7.15020573
H	14.84973063	5.40724410	8.77221730
H	17.95415187	4.46099522	7.49135668
H	19.53878082	4.09534806	6.20551830
H	20.72488633	2.85573119	5.76510408
H	21.29301949	2.41601283	8.10099400
H	16.17613510	-0.29304432	5.52677947
H	21.21237838	4.18269169	7.96995977
H	19.83999327	3.28299225	8.66499291
O	17.81798012	3.49777275	10.20536804
H	17.82448662	4.42797190	10.47670806
H	17.15669610	3.46041198	9.49382175
H	13.57743168	-1.14947761	5.72804114

RsLOV **Qa** S_0

C	50.59294706	-14.38706985	-8.87698365
C	50.86173958	-15.33316786	-7.71156442
O	51.93856548	-15.87911474	-7.50797205
H	49.93902347	-13.59599633	-8.50998367
H	50.09902979	-14.91296594	-9.69403972
H	51.54377010	-13.97385915	-9.23503995
N	49.79935021	-15.48288063	-6.85087424
C	49.86227721	-16.44320414	-5.76384733
C	49.88414954	-17.87629328	-6.29980879
O	49.20709053	-18.20016294	-7.28178418
C	48.63551761	-16.29023657	-4.85320489
H	48.87022709	-15.25461616	-7.19078029
H	50.79377433	-16.25791315	-5.20648353
H	48.61714643	-17.09571541	-4.10347195
H	48.65726140	-15.31572825	-4.34216257
H	47.70356636	-16.35595699	-5.44094099
N	50.60159540	-18.77726571	-5.60269921
C	50.45998784	-20.18685457	-5.87407702
C	51.17263153	-21.01223328	-4.81493236
O	51.84624199	-20.52534327	-3.91526525
H	51.13317533	-18.52336884	-4.77496048
H	49.39398112	-20.45851527	-5.83731550
H	50.84749000	-20.44063411	-6.87608980
N	50.97156264	-22.35557962	-4.97587009
C	51.25112349	-23.32681996	-3.92984871
C	51.53711067	-24.66831233	-4.61539232
O	50.65335455	-25.17623867	-5.32627813
C	50.01902576	-23.52806172	-2.99671197
O	48.86656159	-23.81607194	-3.80056758
C	49.69772378	-22.31357745	-2.14124205
H	50.25802893	-22.64230516	-5.63853128
H	52.10264186	-22.95563302	-3.34299054
H	50.23523938	-24.39567423	-2.34029115
H	49.16718528	-24.42364490	-4.51163907
H	48.82543980	-22.53971923	-1.51013645
H	50.55030961	-22.05857243	-1.49504088
H	49.45411328	-21.44163976	-2.76599113
N	52.72276208	-25.26302405	-4.38310754
C	53.08500365	-26.56494820	-4.93902158
H	53.39305360	-24.78147122	-3.79556246
H	52.42001745	-26.77804640	-5.77599830
H	54.12420299	-26.53722841	-5.29807891
H	52.98898920	-27.35599416	-4.19499268
C	47.64351411	-29.70872044	3.41558978
C	46.96662529	-28.35413680	3.37907537
O	46.61697491	-27.75260515	4.39814605
H	48.15863519	-29.88122058	2.47083921
H	48.35190231	-29.73682943	4.25423012
H	46.90186188	-30.49295350	3.57055641
N	46.75337395	-27.86622252	2.12523923
C	46.08698506	-26.59523365	1.86995556
C	44.53915071	-26.70524553	1.93063725
O	43.81898233	-26.18099978	1.07768945
C	46.51985353	-26.03935623	0.51546110
S	48.29454019	-25.57376958	0.40052581
H	46.82604379	-28.51027468	1.33260615
H	46.38703099	-25.90818224	2.67847792
H	45.95494692	-25.12581866	0.29356903
H	46.27804842	-26.76070076	-0.27660972
H	48.78211569	-26.76350688	0.83891754
N	44.09281219	-27.40539021	2.99851392
C	42.71046691	-27.36377444	3.47223419
H	44.79852535	-27.59630566	3.71287391
H	42.08353057	-26.86902588	2.72169777
H	42.32373729	-28.36104220	3.67629792
H	42.73884293	-26.78030149	4.39155768
C	38.93604091	-23.77993020	5.17491983
C	38.52914171	-25.09944065	4.50382896
C	39.03760235	-25.21827195	3.06017698
C	39.37283879	-26.65816621	2.64478309
O	39.46120610	-27.57837899	3.45436325
N	39.59650582	-26.82521107	1.31358922
H	38.44596425	-22.90900853	4.74003601
H	37.43469563	-25.23922743	4.53100309
H	38.95650705	-25.94170155	5.07049179
H	39.97506176	-24.64439849	2.93301916
H	38.33177023	-24.78605898	2.33030468
H	39.97351159	-27.72149736	1.02752779
H	39.73414815	-26.02565404	0.69046520
H	38.70101070	-23.78497855	6.25131859
H	40.01501596	-23.68606151	5.05604626
C	38.54901538	-19.54504935	2.58497645
C	39.81867046	-19.64074745	1.73235270
C	40.05857371	-20.91958672	0.93708050
O	40.97206360	-20.95067303	0.08815869
N	39.31261614	-22.00325410	1.19378376
H	38.38773761	-20.47590510	3.15873895
H	40.71552996	-19.54047244	2.36903396
H	39.88214538	-18.81172621	1.00970989
H	39.54479790	-22.88127875	0.70862649
H	38.59173046	-21.99308506	1.90164956
H	38.64302091	-18.77600939	3.35200556
H	37.65100656	-19.36598720	1.99400577
C	39.25293548	-18.03510864	-3.47804361
C	40.08733417	-18.92764137	-2.57286841
C	41.50871300	-19.17421865	-3.08610876
O	41.87311932	-18.87336646	-4.22674036
N	42.31912054	-19.74280172	-2.16158600
H	39.81242479	-17.11220759	-3.70622996
H	39.61484398	-19.91548872	-2.42677493
H	40.16431412	-18.49028574	-1.56203260
H	43.19629560	-20.15565269	-2.45157941
H	41.93482218	-20.05339379	-1.27034977
H	38.32704447	-17.73293483	-2.98896777
H	39.02800763	-18.49497404	-4.44001471
C	47.23011735	-20.50595231	-7.93397397
C	45.89316775	-20.01704053	-7.39070954
O	44.81687303	-20.49568468	-7.74125867
C	47.47536873	-19.99918196	-9.35973020
O	47.45439241	-18.58755042	-9.44079502
H	48.03602789	-20.16304826	-7.28500279
H	46.67095581	-20.36668175	-10.01931440
H	48.43328205	-20.42468465	-9.73124718
H	48.06414599	-18.25562791	-8.75668875
H	47.18791906	-21.59500009	-7.90301006
N	46.01094414	-19.02048451	-6.47140282
C	44.89650897	-18.46696405	-5.73251496
C	44.53944705	-17.07319108	-6.28182703
O	45.40003652	-16.20701667	-6.44173133
C	45.23622219	-18.37777239	-4.23471764
C	45.42273780	-19.74632387	-3.57596535
C	46.71548464	-20.50390386	-3.88549436
O	47.76208061	-19.94223238	-4.23757909
N	46.62204533	-21.84193859	-3.70771357
H	46.93789901	-18.67037005	-6.26020152
H	44.04710302	-19.14995106	-5.87339697
H	44.39929043	-17.86781047	-3.73368379
H	46.13867382	-17.76225617	-4.09855545
H	44.57336059	-20.39848223	-3.83236636
H	45.40445828	-19.62627853	-2.47809345
H	47.45080273	-22.42531804	-3.81640028
H	45.80414077	-22.25641853	-3.26554669
N	43.21725377	-16.89678699	-6.56259610
C	42.65097966	-15.60801189	-6.94400065
H	42.58280395	-17.61887776	-6.23013218
H	43.45276267	-14.85914872	-6.86978889
H	42.26201618	-15.58400391	-7.96200359
H	41.86001377	-15.35199451	-6.23899532
N	41.75947279	-25.73189467	-1.08403538
C	41.32779005	-24.49353948	-0.69952519
O	40.24454374	-24.35320193	-0.10887370
N	42.09153749	-23.35398797	-0.97334635
C	43.32018292	-23.34713708	-1.59441099
O	43.93386438	-22.32128100	-1.86020216
C	43.83647211	-24.71548589	-1.90842365
N	45.03738633	-24.82587104	-2.40011368
C	45.50249630	-26.08306654	-2.67938982
C	46.83328304	-26.22752870	-3.12986042
C	47.37803377	-27.47035735	-3.41306144
C	48.81312971	-27.61238408	-3.84571300
C	46.55202761	-28.62436545	-3.27136191
C	47.10601772	-29.99107185	-3.57992747
C	45.23977832	-28.49838830	-2.81074709
C	44.69493626	-27.23828704	-2.48695666
N	43.41939985	-27.08247339	-1.97074097
C	42.95146460	-25.83721889	-1.62423580
C	42.58767037	-28.26740047	-1.68391219
C	42.95012139	-28.94162251	-0.35781501
H	41.68709595	-22.44931109	-0.66211177
H	47.42571666	-25.31620785	-3.22379663
H	48.89596156	-28.13135218	-4.81464537
H	49.30137198	-26.63830238	-3.95533957
H	49.38772704	-28.20712089	-3.11497243
H	46.34835147	-30.77773431	-3.45626826
H	47.48375280	-30.04103671	-4.61367490
H	47.95734231	-30.22544879	-2.91935453
H	44.63889220	-29.39975284	-2.70564236
H	42.69188384	-28.95277354	-2.53502744
H	41.55058912	-27.91819646	-1.66104086
H	42.53987080	-28.38712026	0.48600993
H	42.48406324	-29.94198516	-0.34719678
H	44.02100453	-29.08524777	-0.21623457
O	46.73800087	-29.58555682	-0.18890374
H	46.21542828	-30.40124476	-0.19970371
H	46.47360746	-29.12501989	-1.00425907

RsLOV **Qb** S_0

C	50.59305052	-14.38692719	-8.87704355

C 50.81896591 -15.33840952 -7.70938315
O 51.86615863 -15.93485049 -7.49281382
H 49.93899029 -13.59601517 -8.50999674
H 50.09898497 -14.91303764 -9.69396590
H 51.55262371 -13.98484191 -9.22274610
N 49.74137016 -15.44313481 -6.86019152
C 49.75782286 -16.40644214 -5.77688287
C 49.83883461 -17.83137778 -6.33868676
O 49.26106566 -18.15681226 -7.37750742
C 48.50468108 -16.24975276 -4.90817317
H 48.82680582 -15.16208692 -7.19938207
H 50.66705796 -16.22373534 -5.18170142
H 48.46479381 -17.03514710 -4.13963577
H 48.51245702 -15.26504545 -4.41634824
H 47.58774486 -16.32092842 -5.51726158
N 50.54181034 -18.71876267 -5.59851971
C 50.70631249 -20.09717162 -5.98756882
C 50.88640871 -20.97211947 -4.74888928
O 50.95706646 -20.48802961 -3.61718975
H 51.01690376 -18.42302022 -4.75206269
H 49.82007342 -20.42231828 -6.55116195
H 51.57903796 -20.23273376 -6.65313774
N 50.96546271 -22.29359900 -5.01463366
C 51.18614777 -23.34772632 -4.03211843
C 51.63625705 -24.57493128 -4.83292628
O 50.97150814 -24.90489721 -5.82587093
C 49.87871648 -23.79589325 -3.29803814
O 48.83137876 -23.92942436 -4.24857229
C 49.40554925 -22.86619677 -2.19301289
H 50.78054659 -22.62182870 -5.95878618
H 51.92955968 -23.00935245 -3.29507006
H 50.10807565 -24.78238543 -2.83984460
H 49.20089884 -24.37554729 -5.03480583
H 48.49009177 -23.28357458 -1.74937239
H 50.17193586 -22.78508988 -1.40760520
H 49.18916127 -21.86139655 -2.56906959
N 52.67774095 -25.28730852 -4.36227041
C 53.08493539 -26.56506563 -4.93895057
H 53.17905485 -24.93420807 -3.55567035
H 52.42004174 -26.77795369 -5.77604254
H 54.12534284 -26.52090705 -5.29569036
H 52.98903894 -27.35600500 -4.19499931
C 47.64305452 -29.70901149 3.41498544
C 46.96320654 -28.35574185 3.36684405
O 46.62365466 -27.74163160 4.38208641
H 48.15902028 -29.88101976 2.47101184
H 48.35007067 -29.73012428 4.25474066
H 46.90198099 -30.49298234 3.57099233
N 46.73455413 -27.88365187 2.11005758
C 46.07972559 -26.60748008 1.84836356
C 44.53212170 -26.69881528 1.92757288
O 43.80840185 -26.15616608 1.09018992
C 46.50151113 -26.06592130 0.48386126
S 48.28986374 -25.66728843 0.33048714
H 46.81185973 -28.52675299 1.31724593
H 46.39677235 -25.91650562 2.64711923
H 45.96661094 -25.13126484 0.27543582
H 46.21495975 -26.77443847 -0.30443024
H 48.74249270 -26.87844683 0.74691806
N 44.08938582 -27.40683338 2.99212998
C 42.70991191 -27.36297554 3.47110095
H 44.79748911 -27.60434646 3.70183491
H 42.08004149 -26.86835595 2.72297184
H 42.32403358 -28.36103024 3.67692842
H 42.73900390 -26.78098465 4.39199008
C 38.93602593 -23.77994306 5.17495243
C 38.52517452 -25.10430289 4.51676005
C 39.03198651 -25.24310444 3.07469447
C 39.35996195 -26.69052805 2.67880496
O 39.47233787 -27.59347345 3.50549408
N 39.55003527 -26.88311533 1.34678165
H 38.44597356 -22.90901563 4.74000532
H 37.43016836 -25.23996288 4.54660183
H 38.95068896 -25.94122005 5.09273018
H 39.97374913 -24.67784397 2.94116053
H 38.33005178 -24.81458935 2.33932604
H 39.92679682 -27.78212826 1.06895772
H 39.66525303 -26.09268481 0.70618213
H 38.70367325 -23.77790138 6.25222645
H 40.01501035 -23.68604230 5.05604183
C 38.54901284 -19.54502675 2.58509138
C 39.81424871 -19.64948565 1.72844237
C 40.02259860 -20.92117425 0.91187550
O 40.87456211 -20.93325156 0.00207985
N 39.31598319 -22.01936432 1.22144115
H 38.38273810 -20.47383178 3.16087741
H 40.71377357 -19.57755279 2.36580767
H 39.89131575 -18.81169874 1.01804907
H 39.51933611 -22.90112549 0.72710289
H 38.65084549 -22.01937172 1.98212065
H 38.64298329 -18.77595187 3.35195331
H 37.65100487 -19.36603673 1.99398502
C 39.25301062 -18.03502101 -3.47802332
C 40.11886308 -18.92482128 -2.59950078
C 41.51009187 -19.18718725 -3.18966037
O 41.84009866 -18.80999389 -4.31930850
N 42.33582243 -19.85827280 -2.35469630
H 39.80350874 -17.10867189 -3.71523183
H 39.64667870 -19.90495274 -2.41229406
H 40.25730987 -18.47721490 -1.59980940
H 43.21184886 -20.23413671 -2.69625236
H 42.00176300 -20.19298207 -1.45402628
H 38.32701457 -17.73298044 -2.98899100
H 39.02798800 -18.49500515 -4.43999544
C 47.22999426 -20.50607391 -7.93403869
C 45.91367501 -20.06377979 -7.31359424
O 44.86592284 -20.68053323 -7.47481295
C 47.42880510 -19.97183106 -9.35516260
O 47.38874813 -18.55765699 -9.41716958
H 48.03598995 -20.16292919 -7.28502263
H 46.61078629 -20.33746587 -9.99873432
H 48.37848368 -20.37873778 -9.76446316
H 48.07432742 -18.22802513 -8.80917768
H 47.18801368 -21.59500110 -7.90296143
N 46.02262599 -18.95522706 -6.52326675
C 44.91131001 -18.43989552 -5.75204393
C 44.53360731 -17.06057079 -6.29876261
O 45.39266371 -16.19722652 -6.49893586
C 45.25013183 -18.37273992 -4.25317826
C 45.64227344 -19.74507496 -3.67324928
C 47.03414084 -20.24112661 -3.98764305
O 47.98685389 -19.40586636 -3.45160615
N 47.22296123 -21.33048582 -4.61685117
H 46.84581888 -18.37122324 -6.62258405
H 44.08144927 -19.14501056 -5.89276126
H 44.35137777 -18.01320673 -3.72813690
H 46.05335235 -17.64014238 -4.07975034
H 44.94816900 -20.52500534 -4.01648604
H 45.57488457 -19.69491806 -2.57307315
H 48.20891894 -21.60416296 -4.68130014
N 43.21060815 -16.89243202 -6.55454779
C 42.65106269 -15.60790378 -6.94400854
H 42.58125754 -17.60067449 -6.18201177
H 43.45148630 -14.85756203 -6.87179241
H 42.26197204 -15.58404275 -7.96198988
H 41.85998731 -15.35204251 -6.23900215
N 41.69526502 -25.76272269 -1.04543803
C 41.23180216 -24.53689539 -0.65180893
O 40.12872965 -24.42458424 -0.08590922
N 41.98160447 -23.38758252 -0.88006795
C 43.24510627 -23.34201285 -1.43732447
O 43.85880535 -22.30066737 -1.59562330
C 43.76678042 -24.69050629 -1.82773829
N 44.95027968 -24.76658652 -2.36283597
C 45.41632047 -26.00467951 -2.71746432
C 46.70793799 -26.10243755 -3.27986242
C 47.26876068 -27.32797745 -3.60911479
C 48.66160071 -27.41954236 -4.17789904
C 46.50510637 -28.51014016 -3.38745745
C 47.08969514 -29.86062881 -3.71282577
C 45.21970080 -28.42648555 -2.84684518
C 44.65026989 -27.18281159 -2.50222450
N 43.38482420 -27.06244906 -1.95080708
C 42.89347153 -25.83263208 -1.58013729
C 42.57961243 -28.26706979 -1.67971899
C 42.94996179 -28.94206797 -0.35795947
H 41.56037488 -22.48530102 -0.58549860
H 47.24988011 -25.17069358 -3.45302889
H 48.67469520 -27.95784125 -5.13975973
H 49.08460452 -26.42374376 -4.34682052
H 49.33380832 -27.96893233 -3.49640091
H 46.37643200 -30.67373939 -3.51632882
H 47.38870108 -29.92019745 -4.77170018
H 47.99789454 -30.04867957 -3.11602696
H 44.65733826 -29.34732370 -2.70297035
H 42.70183751 -28.94323792 -2.53576227
H 41.53474042 -27.94128594 -1.65670117
H 42.54003241 -28.38696363 0.48598929
H 42.48499147 -29.94316051 -0.34443142
H 44.02101051 -29.08499631 -0.21604725
O 46.75287816 -29.52569057 -0.26669089
H 46.24657497 -30.34911185 -0.32985670
H 46.50227765 -29.03540002 -1.06936271
H 48.88252782 -19.75960680 -3.60406353

RsLOV **Qc** S_0

C 50.59332664 -14.38682802 -8.87724062
C 50.84256161 -15.33061956 -7.70666276
O 51.89798007 -15.91652990 -7.50381377
H 49.93897061 -13.59603998 -8.50997562
H 50.09876658 -14.91310109 -9.69379270
H 51.54761674 -13.97856428 -9.23130031
N 49.78106359 -15.42895376 -6.83665266
C 49.81254789 -16.37797251 -5.73959027
C 49.82352794 -17.81526740 -6.27882041
O 49.19017496 -18.11970990 -7.29392541
C 48.59409520 -16.16436842 -4.83418827
H 48.85977526 -15.16777685 -7.17372237
H 50.74729986 -16.20963546 -5.18150995
H 48.57649329 -16.91517008 -4.03124436
H 48.63077772 -15.15885556 -4.38771696
H 47.65420090 -16.25510624 -5.40519200
N 50.51931919 -18.72116191 -5.56203935
C 50.58985095 -20.11288273 -5.93903331
C 50.88545397 -20.98569472 -4.72554762
O 51.13674537 -20.51647659 -3.61415635
H 51.03904592 -18.44567684 -4.73483946
H 49.62571884 -20.42351295 -6.35620348
H 51.37090339 -20.28147707 -6.70310393
N 50.89111844 -22.30914146 -5.01577985
C 51.11287927 -23.37786005 -4.05592976
C 51.60284882 -24.59630485 -4.84919298
O 50.95386314 -24.95403772 -5.84367547
C 49.79980657 -23.83781674 -3.34696536
O 48.80135901 -24.11433730 -4.32152508
C 49.22376459 -22.81159140 -2.38799900
H 50.50863711 -22.60870886 -5.90733126
H 51.83489405 -23.03093420 -3.30261272
H 50.05616141 -24.76223224 -2.78368185
H 49.24024968 -24.54215240 -5.08264707
H 48.33912813 -23.24151817 -1.89422878
H 49.95939214 -22.52609694 -1.62199683
H 48.91326857 -21.92508816 -2.95053696
N 52.65970987 -25.28656667 -4.37134531
C 53.08508197 -26.56498694 -4.93901436
H 53.14790060 -24.92165975 -3.56209775
H 52.41998279 -26.77799893 -5.77598444
H 54.12609109 -26.51389498 -5.29276369
H 52.98895057 -27.35599797 -4.19500230
C 47.64303642 -29.70899614 3.41499625
C 46.96299704 -28.35541138 3.36364462
O 46.62401943 -27.73842521 4.37738201
H 48.15899546 -29.88101013 2.47099818
H 48.34968263 -29.72872784 4.25504346
H 46.90198036 -30.49298218 3.57099826
N 46.73409263 -27.88634396 2.10555098
C 46.07707870 -26.61188490 1.83995111
C 44.52954863 -26.70346336 1.92247295
O 43.80406800 -26.16400695 1.08446360
C 46.49598330 -26.07476158 0.47273497
S 48.27961813 -25.65681469 0.31904452
H 46.80980414 -28.53251780 1.31505087
H 46.39474592 -25.91732166 2.63539092
H 45.95129211 -25.14717921 0.25840440
H 46.21758018 -26.79095367 -0.31191155
H 48.74489945 -26.85883901 0.74808690
N 44.08895449 -27.40712194 2.99089651
C 42.71004007 -27.36308995 3.47123025
H 44.79851730 -27.60192007 3.69996104
H 42.07973266 -26.86845871 2.72346426
H 42.32397405 -28.36102304 3.67684426
H 42.73899506 -26.78090491 4.39194032
C 38.93602779 -23.77994173 5.17494337
C 38.52667241 -25.10172765 4.51039138
C 39.03289961 -25.23043546 3.06705576
C 39.36338398 -26.67446006 2.66116667
O 39.46786256 -27.58503017 3.48034440
N 39.56395983 -26.85538761 1.32881739
H 38.44597176 -22.90901266 4.74001334
H 37.43190771 -25.23924537 4.53997861
H 38.95359880 -25.94149871 5.08112709
H 39.97294897 -24.66173132 2.93595501
H 38.32896813 -24.79923184 2.33503993
H 39.94279936 -27.75223330 1.04675083
H 39.68831844 -26.06008098 0.69615910
H 38.70244773 -23.78133432 6.25184914
H 40.01501088 -23.68604272 5.05604436
C 38.54897379 -19.54507443 2.58509219
C 39.81619185 -19.63680069 1.72595287
C 40.04437879 -20.90445030 0.90608436
O 40.92054995 -20.91398660 0.01910538
N 39.32540657 -22.00114146 1.19066240
H 38.38891556 -20.47697583 3.15725434
H 40.71503343 -19.55423571 2.36286140
H 39.88326840 -18.79622353 1.01748686
H 39.54245745 -22.88096505 0.69908187
H 38.64041768 -22.00245911 1.93349819
H 38.64300477 -18.77595234 3.35195224
H 37.65102170 -19.36599640 1.99397414
C 39.25297681 -18.03503529 -3.47804725
C 40.12705051 -18.92542708 -2.60720502
C 41.50418223 -19.20579180 -3.22315432
O 41.81729339 -18.83780258 -4.35971238
N 42.34134530 -19.88463096 -2.40262082
H 39.80119295 -17.10769667 -3.71725009
H 39.65038395 -19.90051840 -2.40531118
H 40.28834051 -18.47311780 -1.61303871
H 43.19307164 -20.29147529 -2.77129796
H 42.02207462 -20.21062452 -1.49315963
H 38.32702875 -17.73294525 -2.98898521
H 39.02797180 -18.49501733 -4.43998532
C 47.23003438 -20.50600650 -7.93404160
C 45.93090518 -20.09616560 -7.25507692
O 44.92291142 -20.79986402 -7.26924146
C 47.39958250 -19.95147330 -9.34932250
O 47.39288576 -18.53520617 -9.38882828
H 48.03597344 -20.16301545 -7.28495453
H 46.56104125 -20.28713371 -9.98338077
H 48.32977143 -20.37265298 -9.78803774
H 48.08943701 -18.23149632 -8.77930494
H 47.18799537 -21.59500158 -7.90301255
N 46.00094661 -18.91491804 -6.57481147
C 44.91376174 -18.43715349 -5.74625478
C 44.52764153 -17.03961849 -6.23537278
O 45.37710971 -16.15196192 -6.35035131
C 45.28633496 -18.42882223 -4.25204342
C 45.82002202 -19.80220293 -3.77317349
C 47.32167920 -20.00031043 -3.95667628
O 47.81311526 -20.72404657 -4.81794658
N 48.11959080 -19.29917920 -3.08777303
H 46.80792921 -18.31561772 -6.71274813
H 44.08265669 -19.14011397 -5.88903024
H 44.37501564 -18.17519646 -3.68823607
H 46.02696276 -17.63615360 -4.05726892
H 45.32304197 -20.61879645 -4.31561526
H 45.59107321 -19.93262734 -2.70165923
H 49.11638934 -19.50903794 -3.09714583
H 47.73004337 -18.88428704 -2.25079152
N 43.21684937 -16.89145744 -6.56010600
C 42.65096304 -15.60806714 -6.94393551
H 42.58480943 -17.62109010 -6.23769799
H 43.45014605 -14.85696655 -6.86869005
H 42.26205703 -15.58397211 -7.96202057
H 41.85998599 -15.35198509 -6.23902644
N 41.71264669 -25.75451806 -1.06417301
C 41.26875884 -24.52286045 -0.67102270
O 40.17181573 -24.39439865 -0.09773014
N 42.03324851 -23.38288200 -0.90915615
C 43.28755639 -23.35602588 -1.48849562
O 43.90853664 -22.32154575 -1.67002071
C 43.79244848 -24.71473408 -1.86858784
N 44.97435994 -24.81316100 -2.40610587
C 45.42565971 -26.06196396 -2.74390243
C 46.71834273 -26.18797365 -3.29808648
C 47.26170807 -27.42598541 -3.60971268
C 48.65751519 -27.54436940 -4.16625713
C 46.47753581 -28.59342303 -3.38271360
C 47.04126187 -29.95598481 -3.69336448
C 45.19152127 -28.48294341 -2.84915061
C 44.64247960 -27.22656153 -2.51794651
N 43.37990677 -27.08176270 -1.96791238
C 42.90694555 -25.84321293 -1.60511354
C 42.56567549 -28.27461617 -1.67867458
C 42.94995412 -28.94206109 -0.35796203
H 41.62539536 -22.47799229 -0.60670453
H 47.28084867 -25.27164064 -3.48542117
H 48.66737789 -28.08367013 -5.12780704
H 49.09876753 -26.55584418 -4.33035845
H 49.31240405 -28.10747125 -3.47924189
H 46.31284214 -30.75555189 -3.49682296
H 47.34810747 -30.02787126 -4.74918301
H 47.94158891 -30.15435912 -3.08804200
H 44.61386455 -29.39286433 -2.69799034
H 42.66823121 -28.95733325 -2.53205590
H 41.52466785 -27.93831625 -1.64214274
H 42.54003109 -28.38697204 0.48599403
H 42.48736483 -29.94443853 -0.33709511
H 44.02101019 -29.08498911 -0.21604302
O 46.73649499 -29.55077003 -0.25587887
H 46.22309765 -30.37075854 -0.30509568
H 46.48900297 -29.07158664 -1.06614062

RsLOV **Qd** S_0

C 50.59299950 -14.38701720 -8.87697656
C 50.85772555 -15.34922241 -7.72870035
O 51.96290393 -15.80307451 -7.45634144
H 49.93899760 -13.59600739 -8.50999917
H 50.09901032 -14.91299462 -9.69400856
H 51.54401177 -13.97133752 -9.23057433
N 49.75223105 -15.64721578 -6.97256903
C 49.83520107 -16.63938883 -5.91873206
C 50.14799663 -18.02278124 -6.50638901
O 49.69151738 -18.40723894 -7.58551292
C 48.52767976 -16.67783181 -5.12079987
H 48.82926607 -15.44670983 -7.34343015

H 50.67754620 -16.35934272 -5.26528571
H 48.52196321 -17.53123167 -4.42748265
H 48.40485703 -15.74184553 -4.55477134
H 47.65474780 -16.77769682 -5.78574098
N 50.92480332 -18.81769543 -5.73514084
C 51.23650962 -20.17571877 -6.10788181
C 51.43834788 -21.01914253 -4.85294614
O 51.91215377 -20.55522437 -3.82226756
H 51.32144564 -18.48313243 -4.86296208
H 50.42930651 -20.56046296 -6.74750936
H 52.17051250 -20.22992017 -6.69980292
N 51.07990427 -22.32462365 -4.99685482
C 51.23276056 -23.32865001 -3.95428960
C 51.53661980 -24.65499712 -4.66087491
O 50.69159935 -25.10960742 -5.44957246
C 49.92091513 -23.51442869 -3.13708632
O 48.82000928 -23.64958839 -4.05025254
C 49.61737557 -22.36373758 -2.19386524
H 50.60666609 -22.62398319 -5.84213376
H 52.03827248 -22.99965496 -3.28248722
H 50.01810245 -24.44853126 -2.54875282
H 49.11081287 -24.26368065 -4.75831541
H 48.63854826 -22.53206325 -1.72098980
H 50.38493696 -22.30423197 -1.40855486
H 49.59528401 -21.40066097 -2.72138897
N 52.69668349 -25.27404644 -4.37205877
C 53.08493856 -26.56491174 -4.93901261
H 53.32618888 -24.82481475 -3.71726770
H 52.42001470 -26.77802475 -5.77600291
H 54.12480731 -26.52190651 -5.29403763
H 52.98898814 -27.35597706 -4.19497610
C 47.64296096 -29.70898291 3.41493488
C 46.96639922 -28.35403425 3.38126054
O 46.61515110 -27.75789322 4.40312406
H 48.15903758 -29.88102812 2.47102331
H 48.35156597 -29.73716700 4.25349841
H 46.90198200 -30.49298911 3.57096612
N 46.75536339 -27.85887796 2.13004096
C 46.09097188 -26.58472636 1.88305328
C 44.54262465 -26.69240647 1.94091738
O 43.82397252 -26.15401725 1.09600606
C 46.52546540 -26.01733854 0.53385866
S 48.30224427 -25.55762465 0.42149703
H 46.83077316 -28.49544438 1.33156153
H 46.39023585 -25.90483557 2.69792446
H 45.96555878 -25.09888687 0.32002009
H 46.28116321 -26.72935939 -0.26539865
H 48.78745726 -26.75676754 0.83634283
N 44.09273355 -27.40670384 2.99815158
C 42.71000658 -27.36295940 3.47100919
H 44.79603853 -27.60542302 3.71258629
H 42.08293789 -26.86828541 2.72069764
H 42.32399813 -28.36101388 3.67693379
H 42.73903253 -26.78102143 4.39201198
C 38.93603170 -23.77994062 5.17491714
C 38.52592135 -25.10418854 4.51581716
C 39.03390213 -25.24212668 3.07399992
C 39.36176459 -26.68920135 2.67705912
O 39.47370008 -27.59291057 3.50276423
N 39.55241318 -26.88108467 1.34478095
H 38.44596764 -22.90899769 4.74004834
H 37.43097281 -25.24033902 4.54466487
H 38.95126269 -25.94125794 5.09167682
H 39.97587479 -24.67689770 2.94183643
H 38.33253003 -24.81284513 2.33850283
H 39.92658118 -27.78094031 1.06624722
H 39.66816293 -26.09091641 0.70429086
H 38.70345804 -23.77856772 6.25209367
H 40.01500998 -23.68605765 5.05602562
C 38.54902988 -19.54503691 2.58497660
C 39.81496775 -19.65328515 1.73261511
C 40.02915087 -20.93261115 0.93237864
O 40.90322304 -20.95674020 0.04390600
N 39.30722457 -22.02371119 1.22853285
H 38.38134737 -20.47276295 3.16228279
H 40.71374446 -19.57079361 2.36950806
H 39.89047558 -18.82361920 1.01241555
H 39.51523017 -22.90598947 0.73762785
H 38.62037292 -22.01581835 1.96960421
H 38.64300827 -18.77599182 3.35199040
H 37.65100270 -19.36599715 1.99400153
C 39.25299644 -18.03508292 -3.47805535
C 40.09053855 -18.93970661 -2.58808944
C 41.47395055 -19.25346596 -3.16600891
O 41.80473681 -18.94839628 -4.31615515
N 42.29071383 -19.88709830 -2.29229222
H 39.81491309 -17.11388132 -3.70681661
H 39.58705773 -19.90418787 -2.39801496
H 40.23352659 -18.48701347 -1.59129473
H 43.14761986 -20.31635646 -2.61646844
H 41.94314831 -20.17725677 -1.38065973
H 38.32703664 -17.73294504 -2.98897071
H 39.02798080 -18.49501315 -4.43998890
C 47.22996886 -20.50602920 -7.93398349
C 45.87945280 -19.97908074 -7.47305037
O 44.81876490 -20.37288799 -7.95162251
C 47.53730876 -20.02930458 -9.36227665
O 47.61484969 -18.62113259 -9.44925513
H 48.03600170 -20.16299857 -7.28499872
H 46.71891699 -20.34802873 -10.02923747
H 48.47102048 -20.52057665 -9.71195068
H 48.31342446 -18.34245934 -8.82683363
H 47.18803527 -21.59500290 -7.90299614
N 45.97031195 -19.03036939 -6.50411672
C 44.83307888 -18.53684789 -5.75303682
C 44.49414434 -17.10538538 -6.22049640
O 45.35727085 -16.23046441 -6.28921832
C 45.15800461 -18.58532402 -4.24973313
C 45.40553314 -20.01229106 -3.73958578
C 46.72074435 -20.69814176 -4.05326623
O 46.58977107 -22.02487220 -3.86107328
N 47.78471215 -20.07331366 -4.41186187
H 46.88030679 -18.89654565 -6.07649822
H 43.98282661 -19.20220716 -5.96181482
H 44.29147639 -18.17717632 -3.70860137
H 46.02263758 -17.93748147 -4.03666932
H 44.61327299 -20.68665158 -4.10342433
H 45.32507036 -20.03962941 -2.63871385
H 48.55361251 -20.73846067 -4.55802723
N 43.19106844 -16.91288308 -6.56976354
C 42.65102247 -15.60807243 -6.94402833
H 42.53625535 -17.64252955 -6.29950076
H 43.46494264 -14.87464959 -6.86078944
H 42.26198395 -15.58399513 -7.96199316
H 41.86005097 -15.35196435 -6.23896024
N 41.70950967 -25.76009485 -1.04707359
C 41.24895396 -24.53499411 -0.64644536
O 40.14344654 -24.42231407 -0.08652535
N 42.00541781 -23.38767841 -0.86259172
C 43.27515671 -23.34460952 -1.40385531
O 43.89492186 -22.30517628 -1.55263712
C 43.79897350 -24.69381057 -1.78917486
N 44.99810058 -24.77559820 -2.28669092
C 45.46431833 -26.01571852 -2.63351457
C 46.78222765 -26.12563340 -3.12868740
C 47.34301335 -27.35105519 -3.45459008
C 48.76491431 -27.45340918 -3.93889255
C 46.54730201 -28.52426602 -3.30644327
C 47.12379702 -29.87507551 -3.64417944
C 45.24185915 -28.43162327 -2.81911490
C 44.67794840 -27.18764366 -2.46530289
N 43.40100681 -27.06244154 -1.94302766
C 42.91322212 -25.83245936 -1.56738190
C 42.58782040 -28.26450841 -1.68136639
C 42.95002100 -28.94197002 -0.35789870
H 41.58532770 -22.48659867 -0.56086723
H 47.34708870 -25.19895773 -3.23542757
H 48.82789079 -27.95446531 -4.91864388
H 49.22550743 -26.46603508 -4.04929720
H 49.38033655 -28.04372926 -3.23841084
H 46.38606509 -30.67930920 -3.51298841
H 47.47908218 -29.90619722 -4.68665298
H 47.99428057 -30.09966050 -3.00534829
H 44.66026885 -29.34642749 -2.72017602
H 42.71259013 -28.93988754 -2.53758072
H 41.54435753 -27.93424931 -1.66473349
H 42.53997309 -28.38704216 0.48601238
H 42.48389660 -29.94239082 -0.34693501
H 44.02100712 -29.08498519 -0.21600651
O 46.74532484 -29.53984913 -0.21639355
H 46.22401916 -30.35613049 -0.23801187
H 46.48277397 -29.07159495 -1.02826662
H 47.43473929 -22.50581468 -4.00938237

RsLOV **Qa** S_1

C 50.59295590 -14.38706594 -8.87698005
C 50.85118984 -15.32663983 -7.70658687
O 51.94899419 -15.79479950 -7.43320899
H 49.93901215 -13.59600474 -8.50998515
H 50.09903270 -14.91296377 -9.69404306
H 51.54524602 -13.97369966 -9.23025815
N 49.74686090 -15.55714880 -6.92121756
C 49.80306958 -16.53549641 -5.85393537
C 49.96360979 -17.95144868 -6.41410928
O 49.50652437 -18.28136826 -7.51166803
C 48.51877005 -16.48322132 -5.01403615
H 48.82914902 -15.38311145 -7.31932232
H 50.68656074 -16.30729788 -5.23669606
H 48.50611547 -17.30676579 -4.28411013
H 48.44914874 -15.52225078 -4.48178145
H 47.62530734 -16.58786173 -5.65250997
N 50.56185102 -18.84217416 -5.59698076
C 50.46289418 -20.25355979 -5.86786390
C 51.17226618 -21.05563729 -4.79218718
O 51.83352713 -20.55359017 -3.89142160
H 50.92072262 -18.57823336 -4.68470200
H 49.40463637 -20.56106549 -5.85838876
H 50.88121489 -20.49054812 -6.86025058
N 50.97918533 -22.40090699 -4.93761300
C 51.25981448 -23.35031564 -3.87395645
C 51.52527869 -24.70067781 -4.54609743
O 50.61868873 -25.23651825 -5.20353856
C 50.03777091 -23.51712839 -2.92303916
O 48.87357826 -23.79516870 -3.70116823
C 49.74324429 -22.28516904 -2.08173661
H 50.25151761 -22.69617106 -5.58121128
H 52.12450276 -22.97807585 -3.30708714
H 50.25726411 -24.37738899 -2.25556715
H 49.13613596 -24.45185687 -4.38122977
H 48.88252723 -22.49530332 -1.42964139
H 50.61187679 -22.01608141 -1.46317926
H 49.48353019 -21.42842801 -2.72042408
N 52.73561675 -25.26539926 -4.37664747
C 53.08499095 -26.56494180 -4.93901604
H 53.42758877 -24.76660176 -3.83061363
H 52.42001904 -26.77804211 -5.77600037
H 54.12385902 -26.54196280 -5.29941360
H 52.98899290 -27.35599574 -4.19499363
C 47.64353459 -29.70874584 3.41562650
C 46.97882134 -28.36343069 3.41798094
O 46.63849565 -27.76261062 4.42791087
H 48.15863578 -29.88122243 2.47083975
H 48.35193317 -29.75188638 4.25333037
H 46.90185828 -30.49295467 3.57052911
N 46.72072329 -27.82015161 2.15806733
C 46.20926623 -26.52058732 1.99512581
C 44.56467134 -26.56826799 2.09844700
O 43.94390266 -25.80701763 1.38720415
C 46.60177895 -25.86761922 0.66622771
S 48.32344697 -25.26629331 0.70317397
H 46.79507253 -28.41965609 1.31165020
H 46.46643764 -25.90611655 2.87331482
H 45.97539412 -24.98418241 0.48971438
H 46.44315611 -26.54952618 -0.17709761
H 48.92242286 -26.47850921 0.56903884
N 44.09611552 -27.43962332 2.98924288
C 42.71048030 -27.36374745 3.47222482
H 44.78111293 -27.79852884 3.65259585
H 42.08748151 -26.87439137 2.71797578
H 42.32372962 -28.36104378 3.67627567
H 42.73883319 -26.78031080 4.39156325
C 38.93605091 -23.77992829 5.17491223
C 38.51954185 -25.08862184 4.48875487
C 38.98134843 -25.16727652 3.02428017
C 39.49462687 -26.55444557 2.60940537
O 39.66976109 -27.46909886 3.41896808
N 39.77883791 -26.67558186 1.29105516
H 38.44595689 -22.90900806 4.74004531
H 37.42731625 -25.23299864 4.54892931
H 38.96891816 -25.94031764 5.02346944
H 39.81473201 -24.46485860 2.83519626
H 38.18982188 -24.86158834 2.31933449
H 40.19074753 -27.54858435 0.98307940
H 39.83220957 -25.86313010 0.65765315
H 38.70082279 -23.79200316 6.25126636
H 40.01501570 -23.68605950 5.05604636
C 38.54902469 -19.54504977 2.58495923
C 39.81978618 -19.62568389 1.73275941
C 40.07718175 -20.88702015 0.91141727
O 41.01114350 -20.89027173 0.08913795
N 39.32888421 -21.98126064 1.11701418
H 38.39346272 -20.48199767 3.15048586
H 40.71409943 -19.52951546 2.37355358
H 39.88063696 -18.78353048 1.02490941
H 39.58289718 -22.84683402 0.60648132
H 38.58127307 -21.98881031 1.79661979
H 38.64302510 -18.77600727 3.35200261
H 37.65099603 -19.36599180 1.99402105
C 39.25293288 -18.03511700 -3.47806825
C 40.08505718 -18.93284784 -2.57811255
C 41.47773565 -19.24766962 -3.13218644
O 41.80675885 -18.99567488 -4.29599892
N 42.29616356 -19.81980426 -2.21812305
H 39.81367524 -17.11279942 -3.70691800
H 39.58342694 -19.90036128 -2.39651716
H 40.20816264 -18.47835523 -1.57945753
H 43.10696339 -20.34843794 -2.52075056
H 41.91557235 -20.08170620 -1.30954233
H 38.32705362 -17.73292112 -2.98896022
H 39.02799435 -18.49497510 -4.44001080
C 47.23008962 -20.50594760 -7.93394402
C 45.88896000 -19.95789555 -7.46417993
O 44.82296741 -20.27071858 -7.98826089
C 47.52200528 -20.03571218 -9.36643974
O 47.55708872 -18.62723734 -9.47553926
H 48.03603948 -20.16305884 -7.28501237
H 46.71117201 -20.38568979 -10.02700752
H 48.46891833 -20.50512378 -9.71213404
H 48.21138025 -18.31264025 -8.82377382
H 47.18792938 -21.59500120 -7.90302027
N 45.99051504 -19.07948259 -6.43019876
C 44.84739847 -18.51890682 -5.73963843
C 44.51645519 -17.11603984 -6.29520209
O 45.38779189 -16.26336569 -6.46478351
C 45.14421737 -18.44567017 -4.23201780
C 45.33052226 -19.82097634 -3.58454824
C 46.64311711 -20.57116086 -3.83074350
O 47.67814336 -20.01929350 -4.25169015
N 46.58259331 -21.87987510 -3.52328537
H 46.89151302 -18.98288171 -5.97460847
H 43.98888204 -19.18600322 -5.90750700
H 44.28435990 -17.95865867 -3.74826160
H 46.02956171 -17.81265665 -4.06283175
H 44.50810832 -20.48479041 -3.89080024
H 45.24316844 -19.72235399 -2.48909532
H 47.40552188 -22.46736398 -3.64719330
H 45.75316460 -22.29269098 -3.08919518
N 43.19531514 -16.90912889 -6.56210074
C 42.65098363 -15.60800649 -6.94398446
H 42.54764992 -17.62517593 -6.24160348
H 43.46262881 -14.87083475 -6.86624547
H 42.26202269 -15.58400397 -7.96200613
H 41.86000530 -15.35199917 -6.23900336
N 41.79494164 -25.70087637 -1.09479780
C 41.34358906 -24.46100751 -0.78181050
O 40.24912154 -24.29940054 -0.17914720
N 42.09426101 -23.34559749 -1.12055835
C 43.35352151 -23.37451370 -1.71778072
O 43.95510730 -22.32260161 -1.98571363
C 43.86748211 -24.71485384 -1.96687972
N 45.14164530 -24.84711052 -2.41568438
C 45.57611475 -26.11855242 -2.63376388
C 46.91763226 -26.33124035 -3.04049813
C 47.44125791 -27.59518550 -3.29883791
C 48.87800171 -27.76605747 -3.72656496
C 46.60012930 -28.72844985 -3.16053150
C 47.11077513 -30.11576204 -3.46995746
C 45.27399878 -28.54341409 -2.72900340
C 44.75242094 -27.27104671 -2.44480182
N 43.45291074 -27.08313386 -1.95187123
C 42.99875632 -25.80166638 -1.66096077
C 42.60414345 -28.24361417 -1.67808287
C 42.95009138 -28.94167209 -0.35781443
H 41.71617789 -22.43407878 -0.82879103
H 47.53332152 -25.43796052 -3.14117612
H 48.95074294 -28.29632816 -4.69094090
H 49.38480593 -26.80204337 -3.85120827
H 49.44789486 -28.36800913 -2.99657676
H 46.34007678 -30.88062150 -3.29041161
H 47.42320958 -30.20423048 -4.52420476
H 47.99462258 -30.37402166 -2.86162305
H 44.64558762 -29.42697038 -2.62139567
H 42.69199153 -28.93664702 -2.52731433
H 41.57130170 -27.88143070 -1.65027065
H 42.53989710 -28.38710613 0.48601353
H 42.48046446 -29.93985606 -0.33680088
H 44.02100834 -29.08520496 -0.21622272
O 46.79746420 -29.34896574 -0.12726588
H 46.41446963 -30.23690559 -0.18630233
H 46.63400643 -28.97062311 -1.01853936

RsLOV **Qb** S_1

C 50.59309094 -14.38691475 -8.87703186
C 50.81509255 -15.33333101 -7.70491997
O 51.86242153 -15.92721314 -7.48153361
H 49.93896817 -13.59603131 -8.51000134
H 50.09897571 -14.91304389 -9.69395654
H 51.55334752 -13.98554106 -9.22159493
N 49.73446863 -15.43422124 -6.85874067
C 49.74708300 -16.40164346 -5.77836071
C 49.85049080 -17.82127113 -6.34853522
O 49.27544478 -18.15004885 -7.38754522
C 48.48378662 -16.26471990 -4.92183328
H 48.82055163 -15.15989507 -7.20539548
H 50.64796393 -16.21252768 -5.17245872
H 48.44243565 -17.06099360 -4.16412348
H 48.47851109 -15.28674203 -4.41654148
H 47.57282910 -16.33419770 -5.53996456
N 50.57680895 -18.69690130 -5.61701070
C 50.76988192 -20.06820990 -6.01256085
C 50.85671281 -20.96961032 -4.78267534
O 50.80917555 -20.51394368 -3.63657255
H 51.02979018 -18.39560418 -4.76123646
H 49.92714836 -20.37777939 -6.64752153
H 51.68968628 -20.19291237 -6.61372145

N	51.00026601	-22.27687197	-5.07628872
C	51.13066195	-23.36824268	-4.12062435
C	51.68065857	-24.54957882	-4.92403696
O	51.15076703	-24.82174706	-6.00984083
C	49.75350113	-23.86824702	-3.56107052
O	48.81433729	-23.92169445	-4.62069607
C	49.17183002	-23.02478611	-2.43678321
H	50.90531568	-22.57808140	-6.04287964
H	51.78450579	-23.05632619	-3.29266257
H	49.93921276	-24.88955562	-3.16215089
H	49.24226288	-24.36880256	-5.37434303
H	48.17797006	-23.41571803	-2.17415815
H	49.82435875	-23.07949939	-1.55134276
H	49.07182600	-21.97344104	-2.72206687
N	52.64756081	-25.29973678	-4.35939736
C	53.08491104	-26.56506224	-4.93892413
H	53.05186975	-24.99143700	-3.48328448
H	52.42004957	-26.77794670	-5.77605043
H	54.12588154	-26.50997055	-5.29346464
H	52.98905111	-27.35601596	-4.19500942
C	47.64313040	-29.70900371	3.41503938
C	47.04414880	-28.32897104	3.44431447
O	46.91049403	-27.64732772	4.44092562
H	48.15896487	-29.88103590	2.47098468
H	48.36303239	-29.77835810	4.24137102
H	46.90196660	-30.49297160	3.57097551
N	46.62479234	-27.83449157	2.19530485
C	46.25306691	-26.48485865	2.07008501
C	44.70331996	-26.31916209	2.56101731
O	44.28314264	-25.17077480	2.60812807
C	46.37918149	-25.89977579	0.65783122
S	48.13117333	-25.76190791	0.15235157
H	46.69688634	-28.40090739	1.33248940
H	46.77019665	-25.88099809	2.82886999
H	45.96696792	-24.88224382	0.64788227
H	45.81232540	-26.48590976	-0.07392241
H	48.26065348	-27.05787316	-0.23536536
N	44.05569331	-27.41284764	2.89587056
C	42.70991471	-27.36301046	3.47111673
H	44.55694387	-28.28646011	2.77773909
H	42.03218090	-26.87812149	2.75809764
H	42.32402305	-28.36102685	3.67692327
H	42.73900358	-26.78096433	4.39197732
C	38.93602541	-23.77994067	5.17495625
C	38.51779816	-25.10268356	4.51979821
C	38.99495194	-25.22862250	3.06575052
C	39.46029401	-26.64230465	2.68630682
O	39.63543405	-27.53317334	3.52222807
N	39.70422149	-26.81115501	1.36430670
H	38.44597372	-22.90901573	4.74000528
H	37.42411457	-25.23969305	4.57300673
H	38.95692664	-25.94028854	5.08477245
H	39.86140999	-24.56760926	2.87603348
H	38.22999283	-24.90082757	2.34238721
H	40.11888573	-27.69230503	1.08296206
H	39.81463077	-25.99712117	0.73809163
H	38.70624532	-23.77550104	6.25310308
H	40.01500972	-23.68604468	5.05603821
C	38.54901067	-19.54500580	2.58507406
C	39.81808995	-19.62065015	1.72979035
C	40.06964171	-20.88316107	0.90826071
O	40.92359668	-20.85848760	0.00366658
N	39.40114554	-22.00629560	1.21421176
H	38.39186847	-20.47989906	3.15283529
H	40.71264398	-19.52641882	2.37150245
H	39.87577498	-18.77765973	1.02370699
H	39.66086499	-22.89216588	0.73826493
H	38.73826875	-22.02608063	1.97654740
H	38.64299155	-18.77596282	3.35196379
H	37.65100170	-19.36604519	1.99398705
C	39.25300184	-18.03503101	-3.47802813
C	40.12795091	-18.92319485	-2.60708925
C	41.50826494	-19.20010087	-3.22005774
O	41.81811293	-18.83021878	-4.35908962
N	42.34143970	-19.87139084	-2.39512456
H	39.80026044	-17.10728788	-3.71885053
H	39.65380619	-19.89959885	-2.40641102
H	40.28518116	-18.47236404	-1.61178962
H	43.18860554	-20.30028006	-2.75002346
H	42.00866019	-20.20406156	-1.49299077
H	38.32701970	-17.73297409	-2.98898570
H	39.02799075	-18.49500378	-4.43999676
C	47.22983853	-20.50617726	-7.93383019
C	45.90716042	-20.05157924	-7.33763725
O	44.85282010	-20.64476614	-7.53930638
C	47.44114874	-19.98486406	-9.35859771
O	47.40709703	-18.57110227	-9.43623388
H	48.03608288	-20.16280653	-7.28519488
H	46.62638120	-20.35412120	-10.00427925
H	48.39263780	-20.39898368	-9.75747003
H	48.07752507	-18.23758514	-8.81391069
H	47.18807919	-21.59500576	-7.90302086
N	46.01230878	-18.95255419	-6.53568701
C	44.90381389	-18.44552751	-5.75600778
C	44.52752696	-17.06087110	-6.28904363
O	45.38462127	-16.19155787	-6.47331009
C	45.24132214	-18.39577580	-4.25590803
C	45.64403661	-19.77209771	-3.69239215
C	47.03839225	-20.24623508	-4.02587362
O	47.98760389	-19.43808516	-3.44344503
N	47.23092355	-21.29403620	-4.72090120
H	46.86330084	-18.40385930	-6.57415031
H	44.07306514	-19.14783848	-5.90519814
H	44.33888559	-18.04995555	-3.72790775
H	46.03789635	-17.65770805	-4.07285369
H	44.95525878	-20.55329595	-4.04147125
H	45.57788411	-19.73967883	-2.59230860
H	48.21682866	-21.55661352	-4.81309455
N	43.20669684	-16.89408909	-6.55644489
C	42.65106486	-15.60790034	-6.94400822
H	42.57514205	-17.60468846	-6.19134508
H	43.45332556	-14.85977092	-6.87050312
H	42.26197331	-15.58403921	-7.96198999
H	41.85998413	-15.35204679	-6.23900400
N	41.89172170	-25.73194537	-0.84824785
C	41.40105458	-24.50884132	-0.52867268
O	40.29416315	-24.38813134	0.06837894
N	42.11565522	-23.37338247	-0.85041586
C	43.37211040	-23.34109668	-1.47082936
O	43.93370462	-22.26771858	-1.68538747
C	43.89014646	-24.66797768	-1.81750561
N	45.09782848	-24.75687956	-2.42881885
C	45.51048068	-26.00756729	-2.77248558
C	46.74814430	-26.16278635	-3.44409873
C	47.27585440	-27.40610158	-3.78365100
C	48.60579092	-27.50575830	-4.49295734
C	46.54860352	-28.57529289	-3.46017558
C	47.09176237	-29.94456045	-3.78743921
C	45.29709694	-28.44680177	-2.82435569
C	44.75894691	-27.19109416	-2.49119197
N	43.50714622	-27.04491244	-1.87394609
C	43.06282598	-25.77868421	-1.49487025
C	42.64502313	-28.20818173	-1.66576901
C	42.94995697	-28.94205876	-0.35797405
H	41.69776956	-22.46635034	-0.59817050
H	47.27272654	-25.24552130	-3.71183706
H	48.52279519	-28.06997222	-5.43708594
H	49.00103092	-26.51114024	-4.73321050
H	49.35757817	-28.03408358	-3.87969548
H	46.41744969	-30.74351030	-3.44288558
H	47.23116893	-30.07877668	-4.87383387
H	48.08338677	-30.11361538	-3.33059858
H	44.72971161	-29.35581816	-2.62249195
H	42.76290372	-28.87590478	-2.53099108
H	41.61140614	-27.84555487	-1.65812093
H	42.54003336	-28.38697389	0.48599615
H	42.47542050	-29.93753779	-0.35966293
H	44.02100971	-29.08499356	-0.21604093
O	46.88998883	-29.02657752	-0.30301674
H	47.24577359	-29.88909019	-0.56343893
H	46.45750094	-28.69891695	-1.12471925
H	48.88638266	-19.78778216	-2.59932506

RsLOV **Qc** S_1

C	50.59334675	-14.38680775	-8.87728216
C	50.83808371	-15.32386006	-7.69984569
O	51.89760468	-15.89702935	-7.48269045
H	49.93897294	-13.59603824	-8.50997515
H	50.09874404	-14.91309522	-9.69378281
H	51.54868927	-13.97954167	-9.22944671
N	49.76881028	-15.42571324	-6.83868433
C	49.79668617	-16.37914935	-5.74471911
C	49.83325962	-17.81228930	-6.29291811
O	49.24225775	-18.11391662	-7.33380563
C	48.56857934	-16.18567093	-4.84800187
H	48.84832270	-15.18224942	-7.19140425
H	50.72433759	-16.20421462	-5.17687466
H	48.55219749	-16.94327807	-4.05061517
H	48.59106217	-15.18378996	-4.39222735
H	47.63344657	-16.27935086	-5.42612192
N	50.51206930	-18.71717848	-5.55904579
C	50.61041637	-20.10076415	-5.95282187
C	50.84723504	-20.99932987	-4.74616560
O	51.03816605	-20.56165912	-3.60971297
H	50.98468401	-18.44897785	-4.70210171
H	49.67182574	-20.40701807	-6.42736893
H	51.43114393	-20.25139953	-6.67840278
N	50.87901658	-22.31109116	-5.07955935
C	51.02654521	-23.41906830	-4.15364738
C	51.62996466	-24.58486684	-4.94360928
O	51.11618689	-24.89511902	-6.02787746
C	49.64726154	-23.93982512	-3.63140940
O	48.77656081	-24.15749726	-4.73150953
C	48.95211261	-22.97834520	-2.68403036
H	50.55197747	-22.57818232	-6.00285516
H	51.64930386	-23.09365890	-3.30810843
H	49.85259184	-24.89805389	-3.10309476
H	49.29235374	-24.59304226	-5.43665720
H	47.99515641	-23.41452680	-2.35929621
H	49.57857481	-22.77218531	-1.80300925
H	48.74048532	-22.04434265	-3.21370402
N	52.62422547	-25.29934551	-4.37212231
C	53.08524919	-26.56501556	-4.93908907
H	53.01253918	-24.97533975	-3.49453817
H	52.41990405	-26.77799115	-5.77592385
H	54.12682877	-26.50069465	-5.28961529
H	52.98890453	-27.35597754	-4.19498658
C	47.64267139	-29.70879849	3.41451756
C	47.04315215	-28.32847594	3.43448958
O	46.91147200	-27.63821862	4.42543628
H	48.15917353	-29.88111961	2.47111554
H	48.36165796	-29.77380088	4.24191635
H	46.90208953	-30.49304066	3.57122221
N	46.62051119	-27.84496796	2.18180475
C	46.24554842	-26.49773200	2.04403550
C	44.69326771	-26.32754479	2.52841340
O	44.26435124	-25.18209164	2.54269668
C	46.37164188	-25.92248051	0.62714692
S	48.12231596	-25.77470659	0.12240764
H	46.69334212	-28.41854630	1.32367068
H	46.75874293	-25.88633685	2.79982022
H	45.95169068	-24.90810212	0.61135980
H	45.80996370	-26.51757726	-0.10130771
H	48.25725600	-27.06859979	-0.27063150
N	44.05447896	-27.41726251	2.89390247
C	42.71021359	-27.36321853	3.47139050
H	44.56414735	-28.28930545	2.80544381
H	42.03327619	-26.87745245	2.75827874
H	42.32385858	-28.36099383	3.67676917
H	42.73896760	-26.78084209	4.39190143
C	38.93601376	-23.77995131	5.17495213
C	38.51912274	-25.09891068	4.51098931
C	38.99433711	-25.21139794	3.05487657
C	39.46636499	-26.61961385	2.66365621
O	39.62777913	-27.52230456	3.48933501
N	39.73077544	-26.77063707	1.34329206
H	38.44598325	-22.90900822	4.74000928
H	37.42576878	-25.23812690	4.56446269
H	38.95994268	-25.94063181	5.06843053
H	39.85543505	-24.54263829	2.86718736
H	38.22473207	-24.88460554	2.33583203
H	40.14385120	-27.65023338	1.05507010
H	39.84926886	-25.95124891	0.72613370
H	38.70461286	-23.78019050	6.25262169
H	40.01500972	-23.68603875	5.05603683
C	38.54898273	-19.54509300	2.58513833
C	39.82029705	-19.60358159	1.72768284
C	40.09713596	-20.85933899	0.90203434
O	40.98399862	-20.82966898	0.02926843
N	39.41158610	-21.98098039	1.17190026
H	38.40005405	-20.48390012	3.14818563
H	40.71336779	-19.49618935	2.36920342
H	39.86549386	-18.75706479	1.02446040
H	39.68944654	-22.86304044	0.69957929
H	38.72124617	-22.00435235	1.90936583
H	38.64298731	-18.77591884	3.35192076
H	37.65102477	-19.36600768	1.99396611
C	39.25310767	-18.03488734	-3.47803182
C	40.13433014	-18.92436619	-2.61337162
C	41.50249855	-19.21837558	-3.24683059
O	41.79798599	-18.85918462	-4.39215386
N	42.34503541	-19.89491129	-2.43222821
H	39.79866110	-17.10639897	-3.72021926
H	39.65545420	-19.89611432	-2.40105444
H	40.31049298	-18.47006895	-1.62277832
H	43.16844696	-20.35429519	-2.80702379
H	42.02395568	-20.21336280	-1.52033357
H	38.32697499	-17.73302542	-2.98903749
H	39.02790148	-18.49506246	-4.43994729
C	47.23022059	-20.50590241	-7.93432624
C	45.92560944	-20.09633864	-7.26681264
O	44.91636465	-20.79736376	-7.29883114
C	47.40465742	-19.95522183	-9.35046303
O	47.40477971	-18.53866868	-9.39306053
H	48.03582787	-20.16313901	-7.28470333
H	46.56577495	-20.28841188	-9.98546457
H	48.33397834	-20.38098165	-9.78703358
H	48.10254039	-18.23758794	-8.78335854
H	47.18794446	-21.59499729	-7.90293264
N	45.98912209	-18.91653452	-6.58489917
C	44.90375478	-18.44418297	-5.75166356
C	44.52005746	-17.04513474	-6.23576009
O	45.36996211	-16.15685432	-6.34742818
C	45.27206887	-18.43884209	-4.25611590
C	45.81498203	-19.80768705	-3.77573311
C	47.31842735	-19.97903952	-3.95617721
O	47.82642677	-20.62436662	-4.86913679
N	48.10579737	-19.34122657	-3.02971020
H	46.80836366	-18.32963420	-6.69622642
H	44.07244463	-19.14623904	-5.89787317
H	44.35589917	-18.19550845	-3.69568420
H	46.00467588	-17.63994553	-4.05552897
H	45.33087110	-20.62860231	-4.32262677
H	45.57583423	-19.94832437	-2.70862214
H	49.09976428	-19.56788911	-3.04168368
H	47.70511711	-19.01776851	-2.15815063
N	43.21041616	-16.89405808	-6.56167988
C	42.65097066	-15.60805915	-6.94393514
H	42.57604610	-17.62203087	-6.23897923
H	43.45279199	-14.86004049	-6.86758487
H	42.26204766	-15.58398740	-7.96201735
H	41.85998895	-15.35198117	-6.23902458
N	41.91414220	-25.71916373	-0.87643762
C	41.44697225	-24.48728000	-0.55912978
O	40.34940577	-24.34675539	0.04921759
N	42.17724283	-23.36463671	-0.89666770
C	43.42284088	-23.35765718	-1.54115402
O	43.99586565	-22.29336880	-1.77891048
C	43.91782221	-24.69577287	-1.87932666
N	45.11929540	-24.81354121	-2.50025552
C	45.51524746	-26.07855991	-2.81770635
C	46.75169444	-26.26949531	-3.48101980
C	47.26229276	-27.52795274	-3.78990791
C	48.59715438	-27.66213627	-4.48391214
C	46.51453588	-28.67844991	-3.44769547
C	47.03721399	-30.06263466	-3.74378541
C	45.26362955	-28.51572568	-2.82062500
C	44.74696867	-27.24414196	-2.51358376
N	43.49823784	-27.06703348	-1.90046801
C	43.07747244	-25.79064298	-1.53366140
C	42.62784075	-28.21758023	-1.66603287
C	42.94996020	-28.94220264	-0.35788047
H	41.77829367	-22.45312038	-0.63348363
H	47.29483567	-25.37212617	-3.77378399
H	48.51293100	-28.23940901	-5.42012983
H	49.01123637	-26.67769825	-4.73253859
H	49.33185063	-28.19542566	-3.85446596
H	46.34683125	-30.84367350	-3.39036063
H	47.18475035	-30.21909210	-4.82600688
H	48.02213339	-30.23871204	-3.27509581
H	44.68129026	-29.41073565	-2.60096010
H	42.72219676	-28.89355744	-2.52777376
H	41.59845622	-27.84417414	-1.64213823
H	42.54003585	-28.38691812	0.48596091
H	42.47843422	-29.93935874	-0.35067929
H	44.02101622	-29.08492857	-0.21602767
O	46.89574485	-29.05168735	-0.30750660
H	47.26437845	-29.91131272	-0.55955824
H	46.45393667	-28.74163420	-1.13095347

RsLOV **Qd** S_1

C	50.59300125	-14.38701598	-8.87697703
C	50.85388209	-15.34700177	-7.72525397
O	51.96230077	-15.78116758	-7.43414034
H	49.93899675	-13.59600760	-8.50999970
H	50.09901021	-14.91299525	-9.69400834
H	51.54501832	-13.97193782	-9.22836618
N	49.74102778	-15.66356296	-6.98808239
C	49.82151771	-16.65361745	-5.93147814
C	50.17025790	-18.03116992	-6.51222753
O	49.74439510	-18.42319670	-7.60127891
C	48.50086735	-16.71558326	-5.15734999
H	48.82103266	-15.47663030	-7.37315906
H	50.64730304	-16.35878169	-5.26350838
H	48.49337161	-17.57411715	-4.46992196
H	48.35335549	-15.78527405	-4.58790089
H	47.64162055	-16.82249839	-5.83836883
N	50.94779417	-18.81017360	-5.72544647
C	51.29475796	-20.16070607	-6.09242476
C	51.43068820	-21.02181519	-4.84040092
O	51.84480210	-20.57263706	-3.77777409
H	51.30073137	-18.47502141	-4.83505127
H	50.53090473	-20.54193821	-6.78501914
H	52.26336993	-20.19809852	-6.62734337
N	51.09626548	-22.32635856	-5.03332391
C	51.17228343	-23.35281394	-4.00575909
C	51.52583067	-24.66118420	-4.72112551
O	50.73575316	-25.10207008	-5.57017386
C	49.79910075	-23.55900886	-3.29927168
O	48.77319689	-23.61395116	-4.29732992
C	49.44221774	-22.46313992	-2.31022931
H	50.64131995	-22.59913546	-5.89733452
H	51.92640144	-23.04100995	-3.26927508
H	49.83780266	-24.52949289	-2.76464039
H	49.06567601	-24.25570134	-4.97681755

H	48.42330812	-22.63811937	-1.93347909
H	50.14450321	-22.47537678	-1.46340594
H	49.48376154	-21.46822044	-2.77317104
N	52.67003703	-25.28278104	-4.37277267
C	53.08493168	-26.56490846	-4.93900913
H	53.25893522	-24.84412290	-3.67473189
H	52.42001756	-26.77802613	-5.77600433
H	54.12573919	-26.51089724	-5.29018386
H	52.98899026	-27.35597844	-4.19497753
C	47.64288407	-29.70896746	3.41489092
C	47.04265102	-28.33046655	3.46459590
O	46.88842522	-27.67511296	4.47590745
H	48.15908436	-29.88103256	2.47104962
H	48.36416859	-29.78577685	4.23977470
H	46.90198988	-30.49299716	3.57096176
N	46.64449388	-27.80557494	2.22146076
C	46.26823382	-26.45581476	2.12311513
C	44.70730302	-26.30852579	2.58589538
O	44.27928689	-25.16313010	2.63517886
C	46.42292704	-25.83306013	0.73033779
S	48.18420954	-25.67990872	0.25988899
H	46.73453831	-28.35296801	1.34740653
H	46.76382620	-25.86990177	2.91002607
H	46.00645275	-24.81732727	0.73811534
H	45.87450748	-26.40173946	-0.02840663
H	48.31046223	-26.95722571	-0.18661622
N	44.05972543	-27.40813769	2.90189358
C	42.71006674	-27.36301057	3.47105101
H	44.56646043	-28.27918239	2.78906703
H	42.03475095	-26.87773609	2.75611276
H	42.32398601	-28.36100933	3.67693437
H	42.73900189	-26.78098053	4.39198772
C	38.93603588	-23.77992723	5.17489052
C	38.51873967	-25.10128267	4.51592873
C	38.99563558	-25.22201546	3.06109640
C	39.46438148	-26.63326101	2.67719723
O	39.63446772	-27.52878794	3.50888559
N	39.71674013	-26.79564032	1.35559886
H	38.44596240	-22.90899922	4.74005122
H	37.42521695	-25.23948948	4.56883991
H	38.95847914	-25.94043904	5.07808951
H	39.85933456	-24.55720701	2.87227940
H	38.22850029	-24.89552326	2.33929313
H	40.12945314	-27.67698768	1.07209098
H	39.83115005	-25.98035620	0.73243616
H	38.70547209	-23.77787974	6.25279409
H	40.01501342	-23.68606923	5.05604841
C	38.54903941	-19.54504517	2.58498663
C	39.82014554	-19.61948755	1.73493603
C	40.09339865	-20.89195552	0.93909401
O	40.99720672	-20.88402358	0.08369542
N	39.39368832	-22.00420259	1.20885931
H	38.39317226	-20.48002225	3.15305391
H	40.71254746	-19.50110922	2.37510344
H	39.86941369	-18.78886771	1.01325301
H	39.66598261	-22.88936706	0.73927470
H	38.68351763	-22.01325851	1.92749405
H	38.64299932	-18.77598335	3.35198323
H	37.65100196	-19.36599693	1.99400242
C	39.25299882	-18.03508848	-3.47806859
C	40.09460697	-18.94141612	-2.59298920
C	41.46609412	-19.28101736	-3.18967368
O	41.77889957	-18.99092769	-4.34986220
N	42.28409642	-19.91735803	-2.32147018
H	39.81410107	-17.11352736	-3.70858588
H	39.58222750	-19.89833422	-2.38867757
H	40.25742830	-18.48385682	-1.60146546
H	43.11434234	-20.39913465	-2.64577573
H	41.93936829	-20.18434452	-1.40117066
H	38.32704103	-17.73294250	-2.98896379
H	39.02797260	-18.49501034	-4.43998881
C	47.22996685	-20.50602872	-7.93397937
C	45.87800291	-19.96966937	-7.49081136
O	44.82280569	-20.34174559	-7.99757700
C	47.55096868	-20.03637338	-9.36224845
O	47.65353313	-18.62959176	-9.45117774
H	48.03600112	-20.16299936	-7.28499760
H	46.72931379	-20.34193467	-10.03139419
H	48.47752275	-20.54376684	-9.70831634
H	48.35840146	-18.36239783	-8.83055882
H	47.18803617	-21.59500269	-7.90299948
N	45.96294581	-19.03537085	-6.50795490
C	44.81907674	-18.54437533	-5.76522600
C	44.48787634	-17.11183669	-6.22841236
O	45.35286688	-16.23872423	-6.30120587
C	45.12870719	-18.60767154	-4.25947207
C	45.36550861	-20.04318046	-3.77175720
C	46.66406592	-20.73667063	-4.13156183
O	46.48928598	-22.06697492	-4.08232981
N	47.75380814	-20.10895422	-4.40659136
H	46.86549676	-18.92634753	-6.05790732
H	43.96885607	-19.20570126	-5.98654703
H	44.25871910	-18.20388443	-3.72075360
H	45.99460436	-17.96594411	-4.03206386
H	44.55794438	-20.70086321	-4.12888092
H	45.30630881	-20.09012864	-2.67092027
H	48.50182654	-20.78226057	-4.61222512
N	43.18419543	-16.91451533	-6.57096541
C	42.65101337	-15.60807730	-6.94402812
H	42.52748943	-17.64181665	-6.29797234
H	43.46732875	-14.87751625	-6.86006145
H	42.26198998	-15.58399148	-7.96199576
H	41.86005287	-15.35196334	-6.23895865
N	41.91665605	-25.72607177	-0.85484909
C	41.44019190	-24.50002645	-0.52408923
O	40.32975773	-24.37198254	0.06314541
N	42.17480311	-23.37177182	-0.82549524
C	43.44138917	-23.35062097	-1.42474686
O	44.01741639	-22.28160974	-1.62563859
C	43.95289719	-24.68173523	-1.76643572
N	45.18324049	-24.78769011	-2.32902029
C	45.58679609	-26.04241086	-2.66753429
C	46.86145925	-26.22161080	-3.26164580
C	47.37086167	-27.46913278	-3.61345334
C	48.74078896	-27.59752256	-4.23370512
C	46.58613563	-28.62224525	-3.37588433
C	47.09966733	-29.99763164	-3.72472362
C	45.31125868	-28.47334297	-2.79486717
C	44.79443513	-27.21305153	-2.44777829
N	43.52743181	-27.05042647	-1.86870754
C	43.09848300	-25.78297589	-1.47869236
C	42.65229932	-28.20541418	-1.66649951
C	42.95004614	-28.94193218	-0.35788550
H	41.76755375	-22.46379399	-0.55862512
H	47.43506452	-25.31293737	-3.44223662
H	48.70436622	-28.14743456	-5.18896433
H	49.19255197	-26.61948886	-4.43998559
H	49.43167235	-28.15902603	-3.57901039
H	46.38683520	-30.78403755	-3.43432645
H	47.28086936	-30.10089158	-4.80826904
H	48.06565103	-30.21418690	-3.23432496
H	44.71408501	-29.37136211	-2.63703518
H	42.76530544	-28.87303886	-2.53221030
H	41.62281642	-27.83190998	-1.66160872
H	42.53995928	-28.38704956	0.48601028
H	42.47477951	-29.93686875	-0.36183513
H	44.02100426	-29.08501298	-0.21601225
O	46.96882217	-28.96610346	-0.27269184
H	47.40070806	-29.79350405	-0.53156851
H	46.51027071	-28.67982693	-1.09480143
H	47.32839731	-22.55855915	-4.22411015

RsLOV **Qa** T_1

C	50.59293314	-14.38705260	-8.87697418
C	50.86129999	-15.33268404	-7.71134762
O	51.94271561	-15.86569602	-7.49805744
H	49.93902294	-13.59599093	-8.50998716
H	50.09903900	-14.91297774	-9.69403851
H	51.54377486	-13.97362716	-9.23464931
N	49.79232474	-15.49684831	-6.86165977
C	49.85582638	-16.46100519	-5.77865636
C	49.90278938	-17.89170647	-6.31968871
O	49.26401709	-18.21822836	-7.32548527
C	48.61855309	-16.32765765	-4.87886710
H	48.86406990	-15.27836495	-7.21024507
H	50.77941679	-16.26727197	-5.21096942
H	48.60134684	-17.13840969	-4.13478922
H	48.62481103	-15.35657999	-4.36085248
H	47.69219799	-16.39826244	-5.47474156
N	50.59864905	-18.79098606	-5.59773643
C	50.45962779	-20.20098302	-5.86782590
C	51.17432161	-21.02453855	-4.80935153
O	51.84764669	-20.53743169	-3.90973185
H	51.10096029	-18.53577027	-4.75245006
H	49.39416633	-20.47624848	-5.82997179
H	50.84672179	-20.45300236	-6.87011380
N	50.97383334	-22.36837460	-4.97091676
C	51.24555787	-23.33607743	-3.91939324
C	51.53094554	-24.68032142	-4.59885911
O	50.64252388	-25.20148386	-5.29486992
C	50.00818403	-23.52989347	-2.99218865
O	48.86125526	-23.81489717	-3.80241360
C	49.68506708	-22.31230366	-2.14167364
H	50.25074729	-22.65097197	-5.62511405
H	52.09632327	-22.96550583	-3.33106995
H	50.21811854	-24.39646249	-2.33206702
H	49.16201193	-24.43353321	-4.50353978
H	48.80987554	-22.53605557	-1.51380342
H	50.53564203	-22.05470383	-1.49381399
H	49.44231051	-21.44316205	-2.77066100
N	52.72470480	-25.26358498	-4.38245635
C	53.08503381	-26.56494227	-4.93903420
H	53.40001863	-24.77541955	-3.80630488
H	52.42000655	-26.77804132	-5.77599301
H	54.12425644	-26.53806176	-5.29795619
H	52.98895322	-27.35598759	-4.19499168
C	47.64355385	-29.70864646	3.41558105
C	46.96068178	-28.35675570	3.36024093
O	46.61931800	-27.73439341	4.36954303
H	48.15859910	-29.88128456	2.47083380
H	48.34927516	-29.72708532	4.25628760
H	46.90186442	-30.49295916	3.57054549
N	46.73170027	-27.89572262	2.09835303
C	46.06975065	-26.62740704	1.81917344
C	44.52202605	-26.73004399	1.90390673
O	43.79174087	-26.22561885	1.04668341
C	46.48646684	-26.11033054	0.44367147
S	48.26231753	-25.66353138	0.29653492
H	46.80339887	-28.55243068	1.31588217
H	46.38323859	-25.92095473	2.60570803
H	45.92546064	-25.20094928	0.19758627
H	46.23120449	-26.85452950	-0.32362456
H	48.74412114	-26.82319199	0.81526283
N	44.08939852	-27.40179984	2.99330914
C	42.71034805	-27.36387604	3.47256332
H	44.80337277	-27.57968114	3.70328570
H	42.07979267	-26.86981415	2.72446255
H	42.32375719	-28.36109151	3.67609131
H	42.73889664	-26.78013972	4.39145389
C	38.93604192	-23.77992665	5.17491033
C	38.52867275	-25.10262090	4.51041325
C	39.04602998	-25.23604379	3.07108650
C	39.37565819	-26.68139037	2.67081613
O	39.45303750	-27.59551460	3.48740523
N	39.60872833	-26.86245551	1.34200280
H	38.44595848	-22.90900896	4.74003389
H	37.43368527	-25.23858728	4.53152542
H	38.94924552	-25.94173584	5.08668985
H	39.98754987	-24.66834662	2.94581415
H	38.34731175	-24.80661952	2.33276767
H	39.97228506	-27.76718964	1.06517513
H	39.74923965	-26.07044836	0.71194546
H	38.70189987	-23.78184084	6.25152460
H	40.01501443	-23.68606156	5.05605372
C	38.54900919	-19.54504702	2.58498534
C	39.81829427	-19.64220079	1.73290236
C	40.05653886	-20.92353825	0.94213967
O	40.95881687	-20.95474542	0.08229735
N	39.31976464	-22.01003233	1.21690075
H	38.38652029	-20.47471016	3.16031804
H	40.71532231	-19.54055690	2.36922305
H	39.88162477	-18.81562095	1.00764622
H	39.54922320	-22.89024777	0.73798289
H	38.60672465	-21.99833053	1.93260691
H	38.64302314	-18.77599515	3.35199226
H	37.65100170	-19.36600006	1.99400515
C	39.25293061	-18.03507837	-3.47800677
C	40.08564223	-18.92813816	-2.57244937
C	41.50259703	-19.18570069	-3.09147868
O	41.85940114	-18.89996794	-4.23820437
N	42.31741855	-19.74560551	-2.16511610
H	39.81288920	-17.11238947	-3.70597884
H	39.60809766	-19.91280722	-2.42095684
H	40.16881054	-18.48727081	-1.56363112
H	43.18202792	-20.18279144	-2.45850161
H	41.93220614	-20.05002854	-1.27247321
H	38.32702627	-17.73295287	-2.98898483
H	39.02802811	-18.49497780	-4.44001680
C	47.23015561	-20.50602253	-7.93395105
C	45.89053293	-20.00834810	-7.40655364
O	44.81458149	-20.46007397	-7.79181674
C	47.48406242	-20.00540323	-9.36098646
O	47.47189743	-18.59409095	-9.44659930
H	48.03601339	-20.16300005	-7.28501380
H	46.67909386	-20.37036435	-10.02126425
H	48.44082055	-20.43787080	-9.72738101
H	48.08690194	-18.26509812	-8.76549389
H	47.18788255	-21.59499766	-7.90302594
N	46.00391169	-19.03042294	-6.46719401
C	44.88396366	-18.48052253	-5.73344208
C	44.53311438	-17.08121040	-6.27548447
O	45.39543247	-16.21498756	-6.42459683
C	45.21410650	-18.40597426	-4.23279004
C	45.39870268	-19.78082607	-3.58593491
C	46.69440069	-20.53558237	-3.89146080
O	47.73919581	-19.96925021	-4.24778431
N	46.60789185	-21.87096841	-3.70682207
H	46.93310180	-18.72532961	-6.20399157
H	44.03340066	-19.15980344	-5.88603531
H	44.37314278	-17.90253513	-3.73207827
H	46.11425340	-17.78941069	-4.08512167
H	44.55261869	-20.43240027	-3.85388183
H	45.37092378	-19.67181090	-2.48731451
H	47.43840511	-22.45102849	-3.81876621
H	45.79228043	-22.29700420	-3.26932526
N	43.21297341	-16.89973144	-6.56381866
C	42.65097664	-15.60801734	-6.94400515
H	42.57489450	-17.62324972	-6.24176276
H	43.45498289	-14.86185546	-6.86843023
H	42.26201163	-15.58400021	-7.96200211
H	41.86001514	-15.35199303	-6.23899045
N	41.79406865	-25.70972389	-1.09929221
C	41.34352423	-24.46193135	-0.69314269
O	40.26542729	-24.37974970	-0.07826451
N	42.08122684	-23.34449807	-0.99084544
C	43.32779106	-23.35603287	-1.62176124
O	43.92269784	-22.30892619	-1.87838750
C	43.84488894	-24.69096297	-1.92739106
N	45.09434014	-24.79441786	-2.45494335
C	45.51592274	-26.05448012	-2.70949975
C	46.84352093	-26.25137009	-3.15304948
C	47.36490991	-27.50876366	-3.42958059
C	48.79314597	-27.66700872	-3.86200289
C	46.53258199	-28.67286883	-3.28313755
C	47.06914338	-30.04369789	-3.60077594
C	45.22933744	-28.51159138	-2.82191659
C	44.69778757	-27.23966740	-2.50829065
N	43.43285370	-27.08471588	-1.98282972
C	42.99636624	-25.79171218	-1.64208468
C	42.59116040	-28.25374204	-1.67988896
C	42.95018664	-28.94157287	-0.35778345
H	41.69035372	-22.43475597	-0.67885373
H	47.45970900	-25.35750436	-3.25045234
H	48.86540607	-28.22573178	-4.80983044
H	49.29162295	-26.70251735	-4.00432774
H	49.35977732	-28.24821134	-3.11315261
H	46.30764372	-30.82135831	-3.44479242
H	47.40603518	-30.10993369	-4.64848975
H	47.94165305	-30.28921121	-2.97206711
H	44.60904386	-29.39858950	-2.70449553
H	42.67472792	-28.94157223	-2.53219719
H	41.55767570	-27.89615718	-1.64754846
H	42.53981397	-28.38713254	0.48599341
H	42.48163722	-29.94073434	-0.35506328
H	44.02099871	-29.08527804	-0.21624362
O	46.72497703	-29.62250503	-0.20419432
H	46.20678174	-30.43990967	-0.24377857
H	46.52545170	-29.17530506	-1.04544317

RsLOV **Qb** T_1

C	50.59308571	-14.38695904	-8.87703704
C	50.81914716	-15.33770619	-7.70909454
O	51.87006386	-15.92538600	-7.48668510
H	49.93896780	-13.59603702	-8.50998975
H	50.09896772	-14.91300801	-9.69397458
H	51.55258788	-13.98460478	-9.22256137
N	49.73747584	-15.45185454	-6.86621578
C	49.75571875	-16.41890411	-5.78640293
C	49.85306143	-17.84094350	-6.35342038
O	49.29346401	-18.16625776	-7.40196821
C	48.49831746	-16.27614849	-4.92164538
H	48.82266041	-15.17770146	-7.21039044
H	50.66100883	-16.23148106	-5.18667140
H	48.45811618	-17.06997369	-4.16163632
H	48.49992049	-15.29680683	-4.41913009
H	47.58372322	-16.34311074	-5.53462701
N	50.55404417	-18.72488859	-5.60675456
C	50.73429062	-20.10072340	-5.99551304
C	50.84664252	-20.98870494	-4.75788143
O	50.84083829	-20.51792981	-3.61758407
H	51.00258206	-18.42838629	-4.74654689
H	49.87760441	-20.41605869	-6.60846698
H	51.64041040	-20.23428737	-6.61530342
N	50.96067773	-22.30396378	-5.03521235
C	51.12996481	-23.37610747	-4.06137124
C	51.63253715	-24.58134998	-4.86308692
O	51.01987446	-24.89666247	-5.89398281
C	49.78337556	-23.85138701	-3.41813598
O	48.79470482	-23.95148131	-4.43270106
C	49.24668521	-22.96563320	-2.30545259
H	50.83395592	-22.61811763	-5.99367759
H	51.82674541	-23.04990087	-3.27498784
H	49.98923223	-24.85395871	-2.98389514
H	49.20864028	-24.38464898	-5.20381429
H	48.28355685	-23.37203138	-1.96366907
H	49.94746877	-22.95721034	-1.45685217
H	49.09511864	-21.93442338	-2.63801886
N	52.65751268	-25.29485946	-4.35963989
C	53.08496925	-26.56501424	-4.93898467
H	53.11805183	-24.95698587	-3.52282025
H	52.42002983	-26.77799168	-5.77602338
H	54.12584503	-26.51127672	-5.29263976
H	52.98900825	-27.35599543	-4.19499302
C	47.64306219	-29.70901614	3.41499996
C	46.95729594	-28.35847775	3.34974947

O 46.62235929 -27.72740700 4.35619161
H 48.15901340 -29.88101626 2.47100744
H 48.34754343 -29.72117944 4.25667163
H 46.90197898 -30.49298202 3.57098453
N 46.71687119 -27.90916737 2.08632669
C 46.06421165 -26.63607698 1.80303974
C 44.51722075 -26.72326798 1.90316407
O 43.78348618 -26.20103110 1.06055096
C 46.47197823 -26.12700159 0.42113094
S 48.25909859 -25.73554070 0.24442173
H 46.79902612 -28.56147094 1.30134720
H 46.39171284 -25.92799596 2.58253071
H 45.93615652 -25.19870504 0.18934245
H 46.17866724 -26.85690095 -0.34574571
H 48.71325814 -26.91970916 0.73159045
N 44.08685778 -27.40403901 2.98839569
C 42.70991651 -27.36298115 3.47109760
H 44.80220890 -27.58827127 3.69495839
H 42.07733252 -26.86899726 2.72475635
H 42.32403258 -28.36102897 3.67693282
H 42.73900269 -26.78098121 4.39198794
C 38.93603557 -23.77995168 5.17491187
C 38.52520346 -25.10721178 4.52244836
C 39.04264446 -25.25981115 3.08546162
C 39.36370958 -26.71241191 2.70361479
O 39.46140888 -27.61060086 3.53623781
N 39.56636086 -26.91733315 1.37412265
H 38.44596870 -22.90900943 4.74002319
H 37.42963172 -25.23906984 4.54478655
H 38.94316750 -25.94116925 5.10810440
H 39.98936505 -24.70183576 2.95602968
H 38.34950995 -24.83240977 2.34114376
H 39.92895507 -27.82493262 1.10542727
H 39.68682161 -26.13405065 0.72796636
H 38.70430763 -23.77526698 6.25229524
H 40.01501221 -23.68603574 5.05606379
C 38.54901554 -19.54503924 2.58504021
C 39.81497802 -19.64804428 1.72922344
C 40.02899439 -20.92334494 0.92058581
O 40.87609334 -20.93712456 0.00682549
N 39.33054345 -22.02353249 1.24401059
H 38.38259845 -20.47318438 3.16170189
H 40.71401365 -19.56868949 2.36642914
H 39.88845089 -18.81398648 1.01418654
H 39.53375265 -22.90719705 0.75712123
H 38.66785600 -22.02075970 2.00677518
H 38.64297847 -18.77598261 3.35198473
H 37.65101053 -19.36598990 1.99399064
C 39.25299469 -18.03501444 -3.47799624
C 40.12007405 -18.92423977 -2.60028551
C 41.50941860 -19.18803293 -3.19449094
O 41.83395459 -18.81558200 -4.32711076
N 42.33936025 -19.85428688 -2.35973912
H 39.80299549 -17.10845953 -3.71563240
H 39.64776415 -19.90404870 -2.41138397
H 40.26108217 -18.47562367 -1.60137904
H 43.21076849 -20.23883602 -2.70414722
H 42.00747425 -20.18751530 -1.45807197
H 38.32700356 -17.73299076 -2.98900542
H 39.02800662 -18.49499986 -4.44000232
C 47.23005512 -20.50604010 -7.93404514
C 45.91186914 -20.06049286 -7.32075549
O 44.86218897 -20.67096883 -7.49263586
C 47.43270148 -19.97449309 -9.35586826
O 47.39735890 -18.56026213 -9.42009292
H 48.03598212 -20.16296475 -7.28499406
H 46.61427113 -20.33856582 -9.99982607
H 48.38168536 -20.38490550 -9.76341687
H 48.08668921 -18.23260664 -8.81516103
H 47.18797410 -21.59500009 -7.90297995
N 46.01968382 -18.95378093 -6.52831108
C 44.90929358 -18.44055805 -5.75504590
C 44.53217970 -17.06042050 -6.29861968
O 45.39052239 -16.19570905 -6.49616795
C 45.24616988 -18.37732016 -4.25552858
C 45.63470547 -19.75141583 -3.67748773
C 47.02329329 -20.25015501 -4.00166306
O 47.98032035 -19.43423794 -3.44589349
N 47.20207868 -21.32385756 -4.66013148
H 46.84938183 -18.37755802 -6.61401096
H 44.07940961 -19.14510041 -5.89863052
H 44.34727299 -18.01677016 -3.73128935
H 46.05021539 -17.64620236 -4.07933353
H 44.93618345 -20.52784935 -4.01932946
H 45.57219127 -19.70223616 -2.57721384
H 48.18517110 -21.60210171 -4.74011004
N 43.20949010 -16.89264591 -6.55555248
C 42.65103014 -15.60795908 -6.94399795
H 42.57991211 -17.60193584 -6.18552339
H 43.45186778 -14.85813518 -6.87147522
H 42.26199067 -15.58402042 -7.96199650
H 41.85999112 -15.35202134 -6.23900553
N 41.74160919 -25.73920621 -1.05476017
C 41.26221255 -24.50526356 -0.63518051
O 40.16216137 -24.44718731 -0.05259119
N 41.99627871 -23.37908706 -0.87865351
C 43.26840007 -23.35374243 -1.46502546
O 43.86141494 -22.29229727 -1.62572699
C 43.78698954 -24.67087497 -1.84361599
N 45.02422595 -24.74241598 -2.41056118
C 45.43282530 -25.97852255 -2.75743409
C 46.70356540 -26.12647998 -3.36938604
C 47.23952300 -27.36373766 -3.68935094
C 48.60355525 -27.46655463 -4.31282165
C 46.48893926 -28.56032987 -3.41591243
C 47.06232138 -29.91531478 -3.73556109
C 45.22094072 -28.44420595 -2.84886948
C 44.66194200 -27.19027045 -2.51866698
N 43.40583344 -27.06712921 -1.96155851
C 42.94813803 -25.78906476 -1.59609329
C 42.58736356 -28.25474525 -1.67761850
C 42.94997417 -28.94200362 -0.35797751
H 41.58860045 -22.47021741 -0.58448384
H 47.24938591 -25.20882164 -3.59221962
H 48.57358043 -28.03932132 -5.25455565
H 49.01931257 -26.47653909 -4.52847137
H 49.30035157 -28.00123866 -3.64378378
H 46.36024478 -30.72207894 -3.47957446
H 47.30306295 -30.00968714 -4.80759569
H 48.00143161 -30.09470365 -3.18465703
H 44.64984072 -29.35396117 -2.67014555
H 42.69381735 -28.93483634 -2.53373778
H 41.54544737 -27.92149720 -1.65015846
H 42.54003283 -28.38698717 0.48600497
H 42.48276326 -29.94209152 -0.35224712
H 44.02100458 -29.08503107 -0.21603745
O 46.75964184 -29.55416814 -0.28481412
H 46.24540252 -30.37001689 -0.37478039
H 46.56654437 -29.06569372 -1.10472642
H 48.87578499 -19.79417425 -3.59342391

RsLOV **Qc** T_1

C 50.59332563 -14.38683204 -8.87723464
C 50.84302803 -15.32871113 -7.70542755
O 51.90466448 -15.89960094 -7.49271185
H 49.93897061 -13.59603998 -8.50997462
H 50.09876658 -14.91310210 -9.69379170
H 51.54746783 -13.97810897 -9.23103879
N 49.77469827 -15.44181018 -6.84546086
C 49.80815138 -16.39619299 -5.75349546
C 49.84543890 -17.83033461 -6.30067553
O 49.26054627 -18.13378400 -7.34437431
C 48.58239573 -16.20229519 -4.85364770
H 48.85344058 -15.19216822 -7.19135699
H 50.73713045 -16.22048365 -5.18800423
H 48.56699159 -16.95946714 -4.05638188
H 48.60660871 -15.20014614 -4.39871405
H 47.64632245 -16.29540933 -5.43019653
N 50.51575465 -18.73624484 -5.55967144
C 50.60736349 -20.12386660 -5.94370140
C 50.85633488 -21.00827942 -4.72862085
O 51.07225459 -20.55342364 -3.60374142
H 50.99305877 -18.46565180 -4.70584603
H 49.66367906 -20.43315932 -6.40624719
H 51.42100859 -20.28320282 -6.67530530
N 50.86923696 -22.32728213 -5.03672130
C 51.04749233 -23.41392502 -4.08873096
C 51.59165582 -24.60959450 -4.87959460
O 50.99226405 -24.95930044 -5.90803760
C 49.69712946 -23.89967793 -3.47141314
O 48.76024797 -24.14558755 -4.51333933
C 49.06339060 -22.90480618 -2.51620146
H 50.51584435 -22.61061813 -5.94526882
H 51.72154280 -23.07690192 -3.28818180
H 49.92252679 -24.84128375 -2.92318608
H 49.24416808 -24.56880621 -5.24948958
H 48.13377736 -23.33544173 -2.11375805
H 49.74184679 -22.66850996 -1.68328227
H 48.81277670 -21.98951279 -3.06155795
N 52.63767255 -25.29411142 -4.37081719
C 53.08510102 -26.56499392 -4.93900436
H 53.08627791 -24.94065848 -3.53406853
H 52.41997977 -26.77798893 -5.77598444
H 54.12656418 -26.50360947 -5.28932018
H 52.98893999 -27.35600199 -4.19500730
C 47.64296842 -29.70897714 3.41495225
C 46.95711206 -28.35813770 3.34581945
O 46.62342130 -27.72322121 4.35027882
H 48.15901747 -29.88100817 2.47100918
H 48.34704828 -29.71941950 4.25698313
H 46.90200639 -30.49300219 3.57102126
N 46.71577945 -27.91309323 2.08079375
C 46.06091900 -26.64207848 1.79228326
C 44.51403379 -26.72836126 1.89750854
O 43.77826690 -26.20901903 1.05476514
C 46.46386293 -26.14041194 0.40612326
S 48.24582953 -25.72985840 0.22514275
H 46.79499686 -28.56986326 1.29923225
H 46.39023680 -25.92912480 2.56658774
H 45.91774444 -25.21997458 0.16742088
H 46.17788321 -26.87958577 -0.35498637
H 48.71349670 -26.90340711 0.72530223
N 44.08632469 -27.40413330 2.98687749
C 42.71010310 -27.36311196 3.47125825
H 44.80336785 -27.58624278 3.69229629
H 42.07694543 -26.86908811 2.72534433
H 42.32395002 -28.36101706 3.67682927
H 42.73897305 -26.78089094 4.39193232
C 38.93602678 -23.77994073 5.17494737
C 38.52664357 -25.10483270 4.51655436
C 39.04338637 -25.24781492 3.07821690
C 39.36640521 -26.69725193 2.68657481
O 39.45572498 -27.60303304 3.51175033
N 39.58002560 -26.89069331 1.35686952
H 38.44597277 -22.90901266 4.74001234
H 37.43131567 -25.23854585 4.53879668
H 38.94608009 -25.94149416 5.09726461
H 39.98849154 -24.68660932 2.95081664
H 38.34829459 -24.81752004 2.33722988
H 39.94458401 -27.79622994 1.08386496
H 39.71004873 -26.10248898 0.71874307
H 38.70321589 -23.77842834 6.25202250
H 40.01501088 -23.68604373 5.05604136
C 38.54897876 -19.54510740 2.58509619
C 39.81669859 -19.63567100 1.72661641
C 40.04946128 -20.90657706 0.91390629
O 40.91971977 -20.91715081 0.02175712
N 39.33911480 -22.00580748 1.21320375
H 38.38873744 -20.47630574 3.15820978
H 40.71508122 -19.54685046 2.36338205
H 39.88077211 -18.79845661 1.01404116
H 39.55608736 -22.88770042 0.72914324
H 38.65722239 -22.00461800 1.95884247
H 38.64299980 -18.77594335 3.35194324
H 37.65102371 -19.36597741 1.99397714
C 39.25296580 -18.03505228 -3.47804825
C 40.12947420 -18.92428178 -2.60863242
C 41.50420869 -19.20545890 -3.22992673
O 41.81189415 -18.83812818 -4.36813215
N 42.34513833 -19.88368977 -2.41294354
H 39.80020921 -17.10730402 -3.71806305
H 39.65345746 -19.89914518 -2.40399971
H 40.29429825 -18.47060860 -1.61562889
H 43.19205572 -20.29742086 -2.78568347
H 42.02928471 -20.20980075 -1.50274424
H 38.32703378 -17.73293626 -2.98898121
H 39.02797683 -18.49501135 -4.43998932
C 47.22997442 -20.50603349 -7.93401757
C 45.93678651 -20.10994929 -7.23564579
O 44.94768898 -20.83984278 -7.20442767
C 47.38861186 -19.93829991 -9.34468087
O 47.39172803 -18.52108962 -9.36767669
H 48.03600948 -20.16297444 -7.28500851
H 46.54191334 -20.26069194 -9.97485388
H 48.31155022 -20.35991436 -9.79742842
H 48.11233584 -18.23042870 -8.77935627
H 47.18801839 -21.59500253 -7.90299958
N 45.99275140 -18.91067335 -6.58794813
C 44.91080131 -18.43632490 -5.75091133
C 44.52511163 -17.04065981 -6.24109790
O 45.37569310 -16.15499516 -6.36538491
C 45.28806876 -18.42730539 -4.25772884
C 45.82274410 -19.80057671 -3.77908067
C 47.32545705 -19.98995496 -3.95865947
O 47.82397757 -20.67374865 -4.84824567
N 48.11792866 -19.32741954 -3.05522289
H 46.77805239 -18.29333930 -6.76392751
H 44.07895790 -19.13946531 -5.88860463
H 44.37782991 -18.17384489 -3.69190036
H 46.02871037 -17.63449050 -4.06425387
H 45.33083866 -20.61672017 -4.32679930
H 45.58822428 -19.93526936 -2.70956187
H 49.11250666 -19.55002203 -3.06301492
H 47.72209053 -18.95891220 -2.19957704
N 43.21353693 -16.89153544 -6.55916354
C 42.65096908 -15.60806613 -6.94392948
H 42.58134369 -17.61823323 -6.23029009
H 43.45089732 -14.85782419 -6.86878181
H 42.26205804 -15.58397311 -7.96202057
H 41.85998202 -15.35198811 -6.23902945
N 41.75923545 -25.73068937 -1.07209418
C 41.29918479 -24.49033801 -0.65349383
O 40.20476316 -24.41703584 -0.06272967
N 42.04620261 -23.37348910 -0.90739363
C 43.31047617 -23.36598293 -1.51314098
O 43.91079368 -22.31074746 -1.69293466
C 43.81193559 -24.69276169 -1.88516869
N 45.04364829 -24.78702726 -2.45940680
C 45.44010625 -26.03621567 -2.78503885
C 46.71010519 -26.21317969 -3.38516607
C 47.23083121 -27.46399553 -3.68352001
C 48.59957595 -27.59353383 -4.29012568
C 46.46004131 -28.64554551 -3.40305938
C 47.01359882 -30.01287861 -3.70446352
C 45.19188444 -28.50113722 -2.84587637
C 44.65301055 -27.23291642 -2.53222970
N 43.39962957 -27.08553537 -1.97841253
C 42.96093740 -25.79807374 -1.62083141
C 42.57256236 -28.26116052 -1.67595246
C 42.94996412 -28.94204511 -0.35793003
H 41.65191407 -22.46245586 -0.60534420
H 47.27565628 -25.31251202 -3.62494499
H 48.56998229 -28.17348707 -5.22768162
H 49.03260772 -26.61133540 -4.50631154
H 49.27831239 -28.13630480 -3.60905952
H 46.29651655 -30.80551808 -3.44598055
H 47.26118974 -30.12146313 -4.77350913
H 47.94547694 -30.20125211 -3.14451215
H 44.60470714 -29.39872888 -2.65919080
H 42.65796723 -28.94701435 -2.52981443
H 41.53496188 -27.91659263 -1.63355962
H 42.54001304 -28.38698204 0.48599204
H 42.48500919 -29.94343240 -0.34531243
H 44.02101215 -29.08499408 -0.21606302
O 46.73850137 -29.58710995 -0.26978279
H 46.22255593 -30.40374977 -0.34100261
H 46.54277140 -29.11477099 -1.09841324

RsLOV **Qd** T_1

C 50.59299966 -14.38701905 -8.87697666
C 50.85929292 -15.34983906 -7.72928540
O 51.96717021 -15.79356832 -7.45124996
H 49.93899760 -13.59600797 -8.50999960
H 50.09901159 -14.91299308 -9.69401067
H 51.54373660 -13.97072997 -9.23062000
N 49.75206346 -15.65997985 -6.98087367
C 49.83683495 -16.65250189 -5.92755308
C 50.15580548 -18.03503429 -6.51432376
O 49.71296059 -18.41740756 -7.59984971
C 48.52775241 -16.69591501 -5.13229560
H 48.82951510 -15.46561140 -7.35600398
H 50.67705174 -16.36971142 -5.27260530
H 48.52267342 -17.55030883 -4.44016710
H 48.40065732 -15.76094824 -4.56553180
H 47.65688197 -16.79755483 -5.79962114
N 50.92196921 -18.83249017 -5.73496103
C 51.23231711 -20.19151508 -6.10554814
C 51.43308839 -21.03400474 -4.84964891
O 51.90504734 -20.56921900 -3.81855063
H 51.30727761 -18.50141841 -4.85633974
H 50.42517268 -20.57683290 -6.74501662
H 52.16628252 -20.24766989 -6.69734214
N 51.07937055 -22.34066016 -4.99606501
C 51.21282032 -23.34194056 -3.94782751
C 51.52018355 -24.67170536 -4.64681294
O 50.67072088 -25.14234391 -5.42141584
C 49.88843619 -23.51730942 -3.14827703
O 48.79963961 -23.63225858 -4.07715884
C 49.58517953 -22.36916201 -2.20183900
H 50.58899136 -22.63293735 -5.83391686
H 52.01199327 -23.01474154 -3.26771746
H 49.96624689 -24.45580961 -2.56406845
H 49.08190741 -24.26806139 -4.76852312
H 48.59769911 -22.53007122 -1.74449233
H 50.34243826 -22.32377967 -1.40554946
H 49.58095383 -21.40254362 -2.72330880
N 52.69208134 -25.27514189 -4.37212017
C 53.08493803 -26.56490592 -4.93901236
H 53.32440714 -24.81697362 -3.72634484
H 52.42001576 -26.77802893 -5.77600243
H 54.12495654 -26.51898365 -5.29275110
H 52.98898497 -27.35597696 -4.19497630
C 47.64281343 -29.70888115 3.41485425
C 46.96059341 -28.35658176 3.36558587
O 46.61356447 -27.74458928 4.37962993
H 48.15905346 -29.88105315 2.47103618
H 48.34911858 -29.72888066 4.25523819
H 46.90205937 -30.49305420 3.57100526
N 46.73859942 -27.88289647 2.10769319
C 46.07596939 -26.61180034 1.84034120
C 44.52804746 -26.71803304 1.91596998
O 43.79975504 -26.20302996 1.06382538
C 46.50091761 -26.07386949 0.47580789
S 48.27544107 -25.61348819 0.35009781
H 46.81689829 -28.52891548 1.31684112

H 46.38298088 -25.91593813 2.63877612
H 45.93852084 -25.16383046 0.23601184
H 46.25772378 -26.80801656 -0.30460123
H 48.76336176 -26.78844918 0.82699126
N 44.09034680 -27.40368763 2.99506799
C 42.71014009 -27.36299395 3.47104725
H 44.80077260 -27.58786290 3.70691411
H 42.08058823 -26.86885565 2.72237231
H 42.32393203 -28.36099706 3.67689627
H 42.73901406 -26.78101593 4.39201032
C 38.93603080 -23.77994073 5.17492237
C 38.52616747 -25.10654581 4.52026415
C 39.04400355 -25.25596753 3.08294371
C 39.36711849 -26.70727561 2.69818573
O 39.46242506 -27.60792952 3.52819370
N 39.57446987 -26.90859691 1.36863314
H 38.44597176 -22.90899964 4.74003934
H 37.43073770 -25.23940560 4.54206358
H 38.94461834 -25.94126169 5.10447110
H 39.98961990 -24.69592517 2.95442213
H 38.34975776 -24.82869050 2.33950705
H 39.93452318 -27.81663459 1.09807306
H 39.69556494 -26.12466982 0.72374338
H 38.70382952 -23.77661390 6.25213601
H 40.01501088 -23.68605468 5.05603337
C 38.54903179 -19.54514338 2.58498819
C 39.81613761 -19.65215932 1.73368145
C 40.03734883 -20.93496334 0.94125643
O 40.90797675 -20.96044873 0.05005048
N 39.32302326 -22.02840118 1.24979258
H 38.38114374 -20.47222853 3.16312918
H 40.71404604 -19.56191502 2.37078039
H 39.88820139 -18.82621592 1.00894886
H 39.53254718 -22.91185715 0.76571420
H 38.63666454 -22.01826347 1.99123759
H 38.64301578 -18.77595837 3.35195624
H 37.65100873 -19.36595439 1.99400514
C 39.25299983 -18.03513028 -3.47803325
C 40.09043568 -18.93829175 -2.58663249
C 41.47461943 -19.25235564 -3.16263036
O 41.80232218 -18.95817807 -4.31623625
N 42.29509685 -19.87283835 -2.28237130
H 39.81451600 -17.11366912 -3.70693271
H 39.58742413 -19.90319789 -2.39696243
H 40.23134156 -18.48455708 -1.58999536
H 43.14477643 -20.31820477 -2.60516564
H 41.94413861 -20.15901376 -1.37092933
H 38.32703775 -17.73292927 -2.98897821
H 39.02798181 -18.49499235 -4.43999932
C 47.22996442 -20.50602946 -7.93397857
C 45.88031748 -19.97230531 -7.47842676
O 44.82005297 -20.35081723 -7.96998808
C 47.54144386 -20.03282250 -9.36283260
O 47.62776257 -18.62537601 -9.45190700
H 48.03600244 -20.16299846 -7.28499951
H 46.72136343 -20.34756374 -10.02963579
H 48.47238391 -20.53032050 -9.71118771
H 48.33009481 -18.35058861 -8.83183425
H 47.18803940 -21.59500253 -7.90300159
N 45.97267351 -19.03318694 -6.50033638
C 44.83378400 -18.53536720 -5.75472067
C 44.49603149 -17.10566458 -6.22749397
O 45.35941751 -16.23167390 -6.30299687
C 45.15601125 -18.57639177 -4.25076863
C 45.39640843 -20.00096936 -3.73227011
C 46.70279958 -20.70143885 -4.05131733
O 46.55477711 -22.02440907 -3.86101228
N 47.77320979 -20.08749741 -4.41251775
H 46.87901317 -18.92028030 -6.05828372
H 43.98262373 -19.20039732 -5.96145058
H 44.29034358 -18.16187331 -3.71303445
H 46.02267118 -17.93026783 -4.04044182
H 44.59615114 -20.67144752 -4.08493926
H 45.32391856 -20.01965779 -2.63082181
H 48.53272350 -20.76248717 -4.56255450
N 43.19121026 -16.91244672 -6.56999463
C 42.65101607 -15.60808312 -6.94402251
H 42.53724476 -17.64226502 -6.29830635
H 43.46484617 -14.87449787 -6.86113261
H 42.26199004 -15.58399010 -7.96199560
H 41.86004901 -15.35195810 -6.23896447
N 41.75655110 -25.73453289 -1.06879953
C 41.28346351 -24.49988685 -0.64321974
O 40.18382710 -24.43913238 -0.06125082
N 42.02384636 -23.37712095 -0.88355545
C 43.30480530 -23.35892165 -1.45053153
O 43.90577237 -22.30072738 -1.60427688
C 43.82547756 -24.67972605 -1.81362802
N 45.08411326 -24.76490792 -2.33010079
C 45.49568145 -26.00459627 -2.65551467
C 46.81260920 -26.17514724 -3.15620146
C 47.34178968 -27.41576330 -3.47318107
C 48.75492884 -27.54411064 -3.96485181
C 46.53410120 -28.59548513 -3.31364847
C 47.08790292 -29.95462329 -3.65010389
C 45.23556950 -28.46157270 -2.82525125
C 44.69026801 -27.20505508 -2.48379188
N 43.42142633 -27.07100956 -1.95888704
C 42.97197847 -25.79176653 -1.58899690
C 42.59293183 -28.25343665 -1.67881101
C 42.95012107 -28.94183010 -0.35786003
H 41.62034008 -22.46951746 -0.57946545
H 47.40316455 -25.26693737 -3.27386960
H 48.80092747 -28.09164398 -4.92061231
H 49.22745925 -26.56812633 -4.11921391
H 49.36518303 -28.11805665 -3.24541918
H 46.34271240 -30.74623070 -3.48523919
H 47.40829921 -30.01054901 -4.70370469
H 47.97478076 -30.18917718 -3.03709883
H 44.63176519 -29.36079158 -2.71311681
H 42.69621220 -28.93429832 -2.53447414
H 41.55362014 -27.91237519 -1.65462293
H 42.53985604 -28.38714402 0.48602204
H 42.48203188 -29.94138131 -0.35360646
H 44.02100717 -29.08498509 -0.21600902
O 46.73768289 -29.57395503 -0.22752365
H 46.21943675 -30.39177783 -0.25947804
H 46.50990823 -29.11939383 -1.05819769
H 47.39202998 -22.51854367 -4.00823673

RsLOV **Qa** $T_{1\text{-}H}$

C 50.59288594 -14.38706842 -8.87697645
C 50.85214988 -15.33140957 -7.70928986
O 51.93251640 -15.85846714 -7.47960952
H 49.93906036 -13.59595833 -8.50999991
H 50.09904117 -14.91298705 -9.69403300
H 51.54544076 -13.97537265 -9.23152838
N 49.77142468 -15.50304387 -6.87517052
C 49.82695490 -16.46566946 -5.79305109
C 49.92904123 -17.89475861 -6.33289920
O 49.43415864 -18.22779356 -7.41606809
C 48.57646614 -16.35263561 -4.90941016
H 48.84802123 -15.26989790 -7.22619469
H 50.73677601 -16.25996678 -5.20702305
H 48.55533212 -17.16824386 -4.17078368
H 48.56683808 -15.38497892 -4.38523046
H 47.65782788 -16.42541902 -5.51534760
N 50.53474936 -18.79158291 -5.53160313
C 50.43741425 -20.19657714 -5.82841775
C 51.06806705 -21.03161122 -4.72706075
O 51.59559406 -20.55775634 -3.72971989
H 50.87334083 -18.54817890 -4.60485706
H 49.37797795 -20.49172977 -5.88484445
H 50.90522456 -20.42389697 -6.80094658
N 50.96162120 -22.37077918 -4.97750408
C 51.14311460 -23.39347375 -3.96182463
C 51.52299047 -24.68291746 -4.69339316
O 50.72553560 -25.15157424 -5.52481416
C 49.81615471 -23.67973419 -3.19503633
O 48.76925864 -23.89247979 -4.15484277
C 49.38872851 -22.56015478 -2.26291397
H 50.37789440 -22.65493212 -5.75754393
H 51.91560056 -23.04971333 -3.26022842
H 49.96504513 -24.60848233 -2.60669871
H 49.15416810 -24.47401416 -4.84837105
H 48.45854719 -22.85363749 -1.75277257
H 50.16296795 -22.38077465 -1.50225502
H 49.20794711 -21.62379173 -2.80884818
N 52.68365578 -25.28130289 -4.37303773
C 53.08503487 -26.56500493 -4.93900527
H 53.28224494 -24.83714516 -3.68683443
H 52.41999009 -26.77800253 -5.77598777
H 54.12571697 -26.51868901 -5.29177858
H 52.98899820 -27.35600791 -4.19500618
C 47.64355316 -29.70865244 3.41558746
C 46.96212215 -28.35525035 3.36394852
O 46.60525295 -27.74940992 4.37975557
H 48.15860720 -29.88128334 2.47083590
H 48.35023429 -29.72838636 4.25558790
H 46.90186701 -30.49296160 3.57054070
N 46.74813504 -27.87591218 2.10901855
C 46.07785405 -26.60838397 1.84912565
C 44.53100572 -26.70575417 1.93246846
O 43.80678040 -26.16244112 1.09608678
C 46.48469753 -26.06150691 0.48090195
S 48.22658314 -25.60136723 0.29162258
H 46.86223100 -28.50557087 1.31051652
H 46.39117673 -25.91101921 2.64408749
H 45.88592760 -25.16415437 0.24992859
H 46.22461671 -26.77832850 -0.31453709
N 44.09046052 -27.41269502 2.99736676
C 42.71035186 -27.36345631 3.47356226
H 44.79929837 -27.60977624 3.70722022
H 42.08381611 -26.86897514 2.72194241
H 42.32376105 -28.36109423 3.67609056
H 42.73889886 -26.78014416 4.39145585
C 38.93603662 -23.77993348 5.17494025
C 38.52706760 -25.09864493 4.50385444
C 39.02669045 -25.21398901 3.05704512
C 39.36990302 -26.65187144 2.63926045
O 39.46931309 -27.57074021 3.45040400
N 39.58647775 -26.81517278 1.30796343
H 38.44597018 -22.90901192 4.74002156
H 37.43278900 -25.23914048 4.53797257
H 38.95881299 -25.94155909 5.06627678
H 39.95909934 -24.63376928 2.92257287
H 38.31347499 -24.78684803 2.33149631
H 39.97770029 -27.70475742 1.01983498
H 39.71471211 -26.01054931 0.68573848
H 38.70145881 -23.78420404 6.25158805
H 40.01501543 -23.68605812 5.05604118
C 38.54901136 -19.54504638 2.58499786
C 39.81730412 -19.64003788 1.73113272
C 40.04174677 -20.90856364 0.91419260
O 40.91369848 -20.91472364 0.02212347
N 39.33051233 -22.00798870 1.19899523
H 38.38645515 -20.47619763 3.15784670
H 40.71427909 -19.55966353 2.37097619
H 39.88902262 -18.80024473 1.02203448
H 39.55311598 -22.88512856 0.69955798
H 38.64928293 -22.01452859 1.94528304
H 38.64302562 -18.77598833 3.35198438
H 37.65100106 -19.36601043 1.99400765
C 39.25295850 -18.03503021 -3.47800991
C 40.10700692 -18.92093410 -2.58596652
C 41.51493136 -19.17901819 -3.13403386
O 41.86437123 -18.85455120 -4.27505105
N 42.33036376 -19.78749704 -2.24216701
H 39.80434177 -17.10819648 -3.71222609
H 39.63691919 -19.90553664 -2.41559195
H 40.21362243 -18.47608395 -1.58133320
H 43.19119009 -20.22187557 -2.55170430
H 41.95492594 -20.10063662 -1.34815167
H 38.32701664 -17.73298076 -2.98899208
H 39.02802096 -18.49499335 -4.44000798
C 47.23017201 -20.50592590 -7.93405763
C 45.97127828 -20.18114257 -7.13566061
O 45.11418524 -21.03354536 -6.87380762
C 47.35634332 -19.85500566 -9.31018555
O 47.39476858 -18.43995617 -9.22648591
H 48.03599715 -20.16305736 -7.28496040
H 46.48427372 -20.11299857 -9.93455089
H 48.25523555 -20.26141548 -9.81688849
H 48.19183736 -18.20562489 -8.70994345
H 47.18789525 -21.59499856 -7.90299154
N 45.95105879 -18.93280318 -6.61496766
C 44.87160600 -18.45201167 -5.77331887
C 44.51024795 -17.06947880 -6.30317695
O 45.37946497 -16.22187777 -6.52403956
C 45.26128593 -18.41339047 -4.28585289
C 45.47114360 -19.81648928 -3.70844001
C 46.79065983 -20.49932066 -4.05088133
O 47.88201398 -19.94132018 -3.91686300
N 46.66977638 -21.81895637 -4.39328468
H 46.58474288 -18.23893163 -7.00344033
H 44.02464193 -19.14210966 -5.88604595
H 44.43531936 -17.93310237 -3.73845212
H 46.16793388 -17.80646676 -4.14780577
H 44.63124681 -20.46929086 -3.98626802
H 45.47746463 -19.75772336 -2.60642352
H 47.52289519 -22.33947681 -4.59720315
H 45.84173876 -22.07703606 -4.92437789
N 43.18994605 -16.89454069 -6.54746771
C 42.65098553 -15.60803147 -6.94401928
H 42.55574966 -17.58691863 -6.15327570
H 43.45590271 -14.86338050 -6.87019006
H 42.26200332 -15.58400555 -7.96199845
H 41.86002594 -15.35198689 -6.23898484
N 41.71504979 -25.72358855 -1.07396676
C 41.26113038 -24.49181563 -0.70751812
O 40.16828009 -24.35844359 -0.11728564
N 42.00947401 -23.34947070 -0.99104232
C 43.25259598 -23.35504214 -1.59247526
O 43.91071817 -22.33622851 -1.83405014
C 43.72528324 -24.68699022 -1.91478554
N 44.96012724 -24.82564212 -2.47707521
C 45.48071134 -26.07053218 -2.77227139
C 46.77712681 -26.21262416 -3.28581431
C 47.31592042 -27.46737390 -3.56523158
C 48.72146886 -27.60324302 -4.08962264
C 46.52132814 -28.61797193 -3.33729171
C 47.06481016 -29.99521234 -3.62644020
C 45.22806921 -28.47092717 -2.81792229
C 44.67896378 -27.21228359 -2.51407451
N 43.39610324 -27.06570408 -1.97132998
C 42.91082178 -25.81282926 -1.62859086
C 42.58412229 -28.25551473 -1.67870439
C 42.95015425 -28.94165288 -0.35779220
H 41.61161765 -22.44242719 -0.68654415
H 47.36770793 -25.31148055 -3.46184921
H 48.74425634 -28.15227389 -5.04552579
H 49.19270199 -26.62900950 -4.26045441
H 49.35334375 -28.17110434 -3.38509912
H 46.31745741 -30.77692123 -3.42748855
H 47.37770869 -30.08959137 -4.67901781
H 47.95372824 -30.21038481 -3.00989900
H 44.63588690 -29.36878777 -2.65261952
H 42.68143677 -28.94003265 -2.53307942
H 41.54341072 -27.91667851 -1.64474543
H 42.53984714 -28.38710328 0.48598752
H 42.48371377 -29.94224467 -0.34896654
H 44.02100506 -29.08524671 -0.21623400
O 46.77067005 -29.55218605 -0.24149202
H 46.20042352 -30.33518581 -0.25651249
H 46.54884281 -29.08748921 -1.06785162
H 45.47780462 -23.96676713 -2.67589339

RsLOV **Qb** $T_{1\text{-}H}$

C 50.59302564 -14.38699106 -8.87701762
C 50.85379752 -15.35931266 -7.73728138
O 51.96834382 -15.73569702 -7.39417888
H 49.93899760 -13.59600998 -8.50999362
H 50.09898762 -14.91301007 -9.69398469
H 51.54505007 -13.97007739 -9.22533717
N 49.72720975 -15.77678419 -7.07538616
C 49.82617145 -16.75467792 -6.01014664
C 50.33337334 -18.09926963 -6.55311695
O 49.90219104 -18.59246881 -7.59904176
C 48.45897884 -16.94329987 -5.34850209
H 48.81112185 -15.60496812 -7.47538881
H 50.56268664 -16.38361249 -5.27852598
H 48.48811349 -17.77119535 -4.62324168
H 48.15186231 -16.02622162 -4.82541426
H 47.68311213 -17.15512429 -6.09874716
N 51.23487595 -18.73706162 -5.77015687
C 51.76045458 -20.04420214 -6.09411273
C 51.73162040 -20.93647933 -4.85400964
O 52.03821887 -20.50992738 -3.74681025
H 51.59167249 -18.29387843 -4.93076714
H 51.16890575 -20.44403429 -6.92861213
H 52.81158618 -19.97499745 -6.42908113
N 51.37206954 -22.22664945 -5.09465460
C 51.31052199 -23.25784583 -4.06793753
C 51.60987968 -24.58625218 -4.77136129
O 50.83293117 -24.97088069 -5.66021575
C 49.89316190 -23.36803191 -3.43325230
O 48.91306457 -23.37408051 -4.48292010
C 49.55906294 -22.23799130 -2.47521435
H 51.01653228 -22.48386028 -6.00870347
H 52.04253791 -23.00516486 -3.28749437
H 49.84663505 -24.32937282 -2.88343982
H 49.23890917 -24.00667460 -5.15915694
H 48.53050128 -22.36638923 -2.10595917
H 50.24609884 -22.25141717 -1.61660258
H 49.64843738 -21.25602661 -2.95965208
N 52.69422260 -25.27677319 -4.37225917
C 53.08489676 -26.56500392 -4.93895735
H 53.27591281 -24.87194180 -3.64792963
H 52.42005375 -26.77798094 -5.77604439
H 54.12504544 -26.52537770 -5.29589621
H 52.98902783 -27.35599897 -4.19499530
C 47.64307045 -29.70904313 3.41497424
C 46.96016107 -28.35535714 3.35519767
O 46.60629717 -27.74366761 4.36867744
H 48.15901244 -29.88103214 2.47101018
H 48.34869883 -29.72361143 4.25584798
H 46.90195935 -30.49296017 3.57100126
N 46.74099252 -27.88265633 2.09835197
C 46.07389454 -26.61311809 1.83403915
C 44.52714696 -26.69914004 1.93091063
O 43.80173459 -26.13934025 1.10773931
C 46.47129501 -26.07217634 0.45912923
S 48.21718834 -25.63146477 0.25591016
H 46.86444963 -28.50953489 1.29920608
H 46.39708827 -25.91215964 2.62205540
H 45.88366082 -25.16439092 0.23743227
H 46.18818555 -26.77998266 -0.33625139
N 44.08817898 -27.41459683 2.99151997
C 42.70994106 -27.36295797 3.47113325
H 44.79817572 -27.61781651 3.69808035
H 42.08151821 -26.86821090 2.72189076
H 42.32407205 -28.36105204 3.67689827
H 42.73894606 -26.78097391 4.39198531
C 38.93603382 -23.77994872 5.17485937
C 38.52089638 -25.10935452 4.53044211

C	39.02933952	-25.26880298	3.09173275
C	39.36162049	-26.72176898	2.71751329
O	39.47226405	-27.61211171	3.55890491
N	39.55749879	-26.92879233	1.38989604
H	38.44595875	-22.90900065	4.74005134
H	37.42519795	-25.23936295	4.56128733
H	38.94209359	-25.94077549	5.11751318
H	39.97072833	-24.70518603	2.95023955
H	38.32862147	-24.85302260	2.34836232
H	39.94554007	-27.82679405	1.12442972
H	39.67410442	-26.13920124	0.74495944
H	38.70622024	-23.76911942	6.25291062
H	40.01501390	-23.68604468	5.05606937
C	38.54905978	-19.54500543	2.58497219
C	39.81455097	-19.69097796	1.74675956
C	39.97122406	-20.97706887	0.94689398
O	40.72013380	-20.98259792	-0.04956085
N	39.34451383	-22.09166348	1.34864719
H	38.35851189	-20.46397527	3.16997953
H	40.70793468	-19.64571685	2.39576658
H	39.92474817	-18.86538314	1.02766283
H	39.52344173	-22.97675820	0.84305747
H	38.77039711	-22.10228226	2.18036168
H	38.64298080	-18.77603637	3.35203824
H	37.65101873	-19.36597238	1.99398415
C	39.25297183	-18.03511832	-3.47793625
C	40.05566799	-18.93370135	-2.55555872
C	41.46510683	-19.23978038	-3.05286406
O	41.81858676	-19.05559310	-4.22206530
N	42.28157520	-19.71647431	-2.07830808
H	39.82064720	-17.11530428	-3.69896530
H	39.55415301	-19.90464302	-2.39415297
H	40.13619284	-18.48323080	-1.55150619
H	43.07332666	-20.28834722	-2.34850510
H	41.84276529	-20.01811428	-1.20821746
H	38.32699478	-17.73300526	-2.98901221
H	39.02803282	-18.49493536	-4.44004032
C	47.23002638	-20.50600846	-7.93403657
C	45.85540386	-20.00741442	-7.50891695
O	44.82509788	-20.36399893	-8.07163308
C	47.56581142	-20.00621166	-9.34807126
O	47.72793254	-18.60008594	-9.37209575
H	48.03600345	-20.16299148	-7.28500655
H	46.73031118	-20.24975977	-10.02440926
H	48.47013893	-20.53443358	-9.71657786
H	48.49064761	-18.40294641	-8.79423710
H	47.18798341	-21.59500057	-7.90298254
N	45.88471214	-19.14609907	-6.46026931
C	44.72518085	-18.65354295	-5.74388751
C	44.43754730	-17.19025784	-6.18134347
O	45.33426942	-16.34784301	-6.19333420
C	45.02129653	-18.75815315	-4.23711141
C	45.10863115	-20.20007664	-3.69836838
C	46.24310822	-21.13390922	-4.06761374
O	47.41257174	-20.51037501	-4.41213943
N	46.11562900	-22.40586416	-3.98466829
H	46.78337888	-18.97076937	-6.03498469
H	43.87211262	-19.29691956	-6.00070003
H	44.19801776	-18.27335048	-3.69391199
H	45.94090081	-18.19293099	-4.01161840
H	44.18642930	-20.74402201	-3.94193326
H	45.14125207	-20.15039986	-2.59652006
H	47.00288986	-22.86358010	-4.23042501
N	43.15539448	-16.93927516	-6.57300396
C	42.65099807	-15.60800115	-6.94398150
H	42.47045481	-17.65852536	-6.35570155
H	43.48195050	-14.89626410	-6.85111147
H	42.26202507	-15.58399613	-7.96200957
H	41.85998800	-15.35202308	-6.23900945
N	41.66148488	-25.76597062	-1.00651134
C	41.14553304	-24.55930229	-0.64058333
O	40.05801109	-24.47540801	-0.02616905
N	41.82468246	-23.38521583	-0.94335231
C	43.05629419	-23.32283033	-1.56111015
O	43.65870904	-22.26473566	-1.76952933
C	43.57900965	-24.61979265	-1.93756455
N	44.77564304	-24.67542934	-2.58842532
C	45.36135166	-25.89269391	-2.86409730
C	46.64345711	-25.96886781	-3.43392264
C	47.25861513	-27.19018504	-3.70042760
C	48.64565533	-27.23920029	-4.28532138
C	46.56015831	-28.38515686	-3.40142415
C	47.18547284	-29.72958791	-3.68076956
C	45.28314672	-28.31083359	-2.82719717
C	44.65768085	-27.08551875	-2.53635796
N	43.38462655	-27.01659670	-1.95275449
C	42.84189289	-25.78744722	-1.60423299
C	42.61266220	-28.24289427	-1.68372031
C	42.95003407	-28.94185407	-0.35793903
H	41.39017507	-22.48947422	-0.65217781
H	47.17040764	-25.04044406	-3.65693314
H	48.67127089	-27.81941235	-5.22243496
H	49.03197276	-26.23827989	-4.51009778
H	49.35190782	-27.72824580	-3.59217407
H	46.50481796	-30.55503314	-3.42635727
H	47.45434981	-29.83317234	-4.74475624
H	48.11478587	-29.86777773	-3.10284124
H	44.75870248	-29.24115604	-2.61558818
H	42.76348433	-28.91662962	-2.53926355
H	41.55918021	-27.94270091	-1.68078136
H	42.53997806	-28.38703104	0.48600704
H	42.47904378	-29.93965905	-0.36547657
H	44.02100119	-29.08506812	-0.21604802
O	46.81364882	-29.49431676	-0.29898669
H	46.24169638	-30.27283185	-0.37072092
H	46.60288620	-28.97758608	-1.09665935
H	48.07122453	-21.20290743	-4.61201955
H	45.18130211	-23.78127357	-2.95158548

RsLOV **Qc** T_{1-H}

C	50.59332664	-14.38682802	-8.87724062
C	50.84437060	-15.33368572	-7.70950312
O	51.90285776	-15.91313761	-7.50443577
H	49.93897061	-13.59603998	-8.50997562
H	50.09876658	-14.91310109	-9.69379270
H	51.54730505	-13.97815247	-9.23155003
N	49.77985675	-15.44438420	-6.84496836
C	49.81484727	-16.39111220	-5.74699722
C	49.82229740	-17.83110451	-6.27960248
O	49.20336994	-18.13698246	-7.30270081
C	48.59833423	-16.17407613	-4.83908021
H	48.85840538	-15.18294344	-7.18111223
H	50.75117206	-16.22210129	-5.19173588
H	48.58221867	-16.92222877	-4.03412552
H	48.63642483	-15.16723714	-4.39599397
H	47.65700998	-16.26741892	-5.40732967
N	50.49263792	-18.74264915	-5.54448267
C	50.53913836	-20.13922946	-5.91062639
C	50.86974460	-21.00155623	-4.69930275
O	51.13879154	-20.52215715	-3.59664640
H	51.01554664	-18.46958554	-4.71823936
H	49.55684912	-20.44950354	-6.28487155
H	51.28709029	-20.32234643	-6.70357897
N	50.87587178	-22.32867752	-4.97675834
C	51.11907350	-23.38329264	-4.00540096
C	51.56947779	-24.62156571	-4.79324267
O	50.86103138	-25.01120813	-5.73432464
C	49.83075248	-23.81485500	-3.23689603
O	48.78694205	-24.08900972	-4.16485458
C	49.30921835	-22.76999812	-2.26621680
H	50.46992510	-22.63878587	-5.85434114
H	51.87250204	-23.03135421	-3.28580751
H	50.09731753	-24.73592393	-2.67381997
H	49.19009156	-24.53732995	-4.93526635
H	48.47178763	-23.20164985	-1.69562921
H	50.09121030	-22.45353970	-1.56099163
H	48.94869868	-21.90063869	-2.82666971
N	52.66641698	-25.28341225	-4.37098550
C	53.08508197	-26.56498694	-4.93901436
H	53.19630815	-24.89386392	-3.60025517
H	52.41998279	-26.77799893	-5.77598444
H	54.12592070	-26.51354890	-5.29200109
H	52.98895057	-27.35599797	-4.19500230
C	47.64303642	-29.70899614	3.41499625
C	46.96028818	-28.35667770	3.35971439
O	46.60754344	-27.74547544	4.37382054
H	48.15899546	-29.88101013	2.47099818
H	48.34910799	-29.72609370	4.25553732
H	46.90198036	-30.49298218	3.57099826
N	46.74014917	-27.88410765	2.10348393
C	46.07457807	-26.61485983	1.84100480
C	44.52818663	-26.70426178	1.92928789
O	43.80396285	-26.15483493	1.09703751
C	46.48122719	-26.07630334	0.46993793
S	48.23042253	-25.64865938	0.27353776
H	46.85276449	-28.51515755	1.30582855
H	46.39380658	-25.91602317	2.63237496
H	45.89674647	-25.16912367	0.24115568
H	46.20502381	-26.78988690	-0.32293587
N	44.08854988	-27.41255939	2.99364108
C	42.71004007	-27.36308995	3.47123025
H	44.79836861	-27.61226814	3.70160639
H	42.08154382	-26.86830938	2.72226493
H	42.32397405	-28.36102304	3.67684426
H	42.73899506	-26.78090491	4.39194032
C	38.93602779	-23.77994173	5.17494337
C	38.52744702	-25.09785598	4.50208062
C	39.02628495	-25.21018851	3.05477155
C	39.36842529	-26.64735655	2.63374353
O	39.46778197	-27.56795219	3.44304335
N	39.58411074	-26.80804371	1.30207543
H	38.44597176	-22.90901266	4.74001334
H	37.43324483	-25.23896003	4.53650825
H	38.95989230	-25.94158650	5.06275232
H	39.95882644	-24.63001201	2.92091151
H	38.31294295	-24.78086320	2.33063698
H	39.97483696	-27.69715214	1.01179497
H	39.71315167	-26.00231891	0.68138970
H	38.70119067	-23.78525779	6.25153904
H	40.01501088	-23.68604272	5.05604436
C	38.54897379	-19.54507443	2.58509219
C	39.81802105	-19.62876154	1.72652521
C	40.05683949	-20.89137332	0.90094753
O	40.94274480	-20.89152858	0.02279020
N	39.33848227	-21.99111668	1.16910152
H	38.39208088	-20.47918774	3.15435523
H	40.71524082	-19.54314881	2.36530510
H	39.88152963	-18.78451660	1.02193071
H	39.56621317	-22.86827025	0.67212220
H	38.64658376	-21.99902375	1.90552366
H	38.64300477	-18.77595234	3.35195224
H	37.65102170	-19.36599640	1.99397414
C	39.25297681	-18.03503529	-3.47804725
C	40.12402500	-18.92542057	-2.60461510
C	41.50102897	-19.21642715	-3.21567380
O	41.81522219	-18.86852624	-4.35845392
N	42.33566622	-19.88224252	-2.38124412
H	39.80155009	-17.10761904	-3.71664561
H	39.64320185	-19.89821388	-2.40104579
H	40.28446730	-18.47129553	-1.61118423
H	43.17246288	-20.32494967	-2.74391015
H	42.00461129	-20.19052949	-1.46911712
H	38.32702875	-17.73294525	-2.98898521
H	39.02797180	-18.49501733	-4.43998532
C	47.23003438	-20.50600650	-7.93404160
C	45.93692468	-20.11334946	-7.23222397
O	44.94626206	-20.84102322	-7.21064534
C	47.38580262	-19.93583749	-9.34390198
O	47.37465354	-18.51887375	-9.36515585
H	48.03597344	-20.16301545	-7.28495453
H	46.54253983	-20.26588385	-9.97447551
H	48.31302149	-20.34851261	-9.79576103
H	48.08877808	-18.22135379	-8.77289893
H	47.18799537	-21.59500158	-7.90301255
N	45.99855679	-18.92483017	-6.56322725
C	44.90154790	-18.44618395	-5.74690075
C	44.52313590	-17.04962232	-6.24333251
O	45.37934559	-16.16997506	-6.37124396
C	45.25819178	-18.43735971	-4.24899432
C	45.77457082	-19.81530440	-3.76583214
C	47.27301976	-20.04165330	-3.94471194
O	47.75112019	-20.82804149	-4.75843571
N	48.08113094	-19.30436567	-3.11845343
H	46.77068757	-18.29434633	-6.75408513
H	44.07074534	-19.14851879	-5.89482775
H	44.34314663	-18.17855314	-3.69412328
H	46.00368870	-17.65060289	-4.04820768
H	45.26358219	-20.62514049	-4.30527651
H	45.54470141	-19.93622019	-2.69320730
H	49.07927290	-19.50441107	-3.12702523
H	47.69934919	-18.80355666	-2.32646015
N	43.21156596	-16.89342021	-6.55796500
C	42.65096304	-15.60806714	-6.94393551
H	42.57694776	-17.61903461	-6.23107777
H	43.45190361	-14.85899631	-6.86844959
H	42.26205703	-15.58397211	-7.96202057
H	41.85998599	-15.35198509	-6.23902644
N	41.71367679	-25.73207592	-1.07951095
C	41.27404273	-24.49749275	-0.70599613
O	40.17999512	-24.35379355	-0.12040268
N	42.03904253	-23.36175830	-0.97566483
C	43.28414182	-23.37856640	-1.57344442
O	43.95794486	-22.36586863	-1.80115898
C	43.73623234	-24.71361387	-1.90995008
N	44.96736930	-24.86662023	-2.47406130
C	45.47296974	-26.11259362	-2.78578229
C	46.76143861	-26.25855277	-3.31464049
C	47.28918030	-27.51504473	-3.60381871
C	48.68767951	-27.64799665	-4.15036422
C	46.49210453	-28.66218945	-3.36702450
C	47.02789835	-30.04161542	-3.65959347
C	45.20264843	-28.50904545	-2.83798362
C	44.66295919	-27.24702254	-2.52966816
N	43.38244010	-27.08670803	-1.98462426
C	42.90892610	-25.83141561	-1.63437840
C	42.56960209	-28.27022681	-1.67984694
C	42.94995412	-28.94206109	-0.35796203
H	41.64958638	-22.45243668	-0.66924476
H	47.35778697	-25.36282334	-3.50217634
H	48.69690053	-28.19556699	-5.10735030
H	49.14548271	-26.66670586	-4.32216585
H	49.33316156	-28.21371507	-3.45651019
H	46.28128624	-30.82126845	-3.44988607
H	47.32773351	-30.13906225	-4.71578028
H	47.92415850	-30.25732288	-3.05378944
H	44.60743537	-29.40429233	-2.66931285
H	42.65787426	-28.96149359	-2.52961156
H	41.52968365	-27.92985286	-1.63832856
H	42.54003109	-28.38697204	0.48599403
H	42.48737372	-29.94476053	-0.33717600
H	44.02101019	-29.08498911	-0.21604302
O	46.75614017	-29.55265294	-0.25441541
H	46.19483006	-30.34194558	-0.27356316
H	46.54137983	-29.09603383	-1.08731942
H	45.50045426	-24.01686751	-2.65669084

RsLOV **Qd** T_{1-H}

C	50.59299765	-14.38701805	-8.87697862
C	50.85095632	-15.35323882	-7.73028856
O	51.95710410	-15.79237095	-7.43863406
H	49.93899860	-13.59600696	-8.50999859
H	50.09901159	-14.91299409	-9.69400972
H	51.54571943	-13.97281429	-9.22714050
N	49.73576967	-15.67753901	-6.99925750
C	49.81789819	-16.66957622	-5.94552140
C	50.18188403	-18.04351287	-6.52507711
O	49.74749258	-18.44832447	-7.60569119
C	48.49054104	-16.75015446	-5.18481268
H	48.81602352	-15.47979208	-7.37882137
H	50.63323841	-16.36823650	-5.26754449
H	48.49576915	-17.59895181	-4.48515843
H	48.31620486	-15.81856459	-4.62581266
H	47.63979807	-16.88219107	-5.87103039
N	50.97140489	-18.81075834	-5.73774307
C	51.32911691	-20.16146782	-6.09594495
C	51.47245468	-21.00628977	-4.83440281
O	51.90523800	-20.54544486	-3.78466174
H	51.35466141	-18.45255609	-4.86916413
H	50.56682666	-20.55296288	-6.78440826
H	52.29791504	-20.19645479	-6.63047208
N	51.11093438	-22.30990660	-4.99062874
C	51.25211787	-23.32002072	-3.95221995
C	51.55132908	-24.64318593	-4.66540140
O	50.70830982	-25.08134586	-5.46659360
C	49.93716516	-23.50733814	-3.14246654
O	48.84232733	-23.64308427	-4.06640376
C	49.62890200	-22.35917644	-2.19818331
H	50.68766481	-22.61333805	-5.86072852
H	52.05468634	-23.00044814	-3.27216655
H	50.02864789	-24.44354165	-2.55761851
H	49.14896925	-24.24113606	-4.78378836
H	48.65261983	-22.53196297	-1.72145318
H	50.39756963	-22.29752501	-1.41425749
H	49.60415307	-21.39530781	-2.72382617
N	52.70103243	-25.27449974	-4.36928990
C	53.08488882	-26.56489692	-4.93899636
H	53.32799814	-24.83507767	-3.70530378
H	52.42003476	-26.77803095	-5.77601740
H	54.12453108	-26.52352796	-5.29498671
H	52.98900666	-27.35597997	-4.19497730
C	47.64293244	-29.70897614	3.41490825
C	46.96278801	-28.35476880	3.36733583
O	46.60308512	-27.75443372	4.38538972
H	48.15904949	-29.88102918	2.47103018
H	48.34988424	-29.72973513	4.25479430
H	46.90199338	-30.49299621	3.57098026
N	46.75398938	-27.86716297	2.11448429
C	46.08282932	-26.59871177	1.86203274
C	44.53530391	-26.69869823	1.93831011
O	43.81384116	-26.15060506	1.10274652
C	46.49494505	-26.03668754	0.50120296
S	48.23390092	-25.55860627	0.32751301
H	46.86750277	-28.49305970	1.31286117
H	46.39067988	-25.90827855	2.66513365
H	45.88773183	-25.14508325	0.27047477
H	46.25080198	-26.74995698	-0.30267311
N	44.09104347	-27.41225199	2.99722948
C	42.71002409	-27.36296199	3.47101625
H	44.79764363	-27.61315742	3.70821299
H	42.08333181	-26.86820233	2.72057525
H	42.32399305	-28.36101304	3.67693127
H	42.73902507	-26.78101894	4.39201132
C	38.93602980	-23.77994073	5.17492237
C	38.52448230	-25.10311245	4.51489639
C	39.02581181	-25.23626562	3.07051481
C	39.36431607	-26.68036245	2.67040152
O	39.47669453	-27.58613832	3.49463173
N	39.56278966	-26.86414772	1.33911400
H	38.44596975	-22.90899663	4.74004734
H	37.42971215	-25.23997574	4.54899953
H	38.95316943	-25.94127238	5.08673162
H	39.96149937	-24.66242094	2.93192390
H	38.31676033	-24.81351465	2.33868293
H	39.95066192	-27.75773235	1.05892795
H	39.67956389	-26.06719149	0.70475807

H	38.70365277	-23.77857661	6.25224147
H	40.01500988	-23.68605770	5.05602236
C	38.54903777	-19.54506840	2.58497919
C	39.81507502	-19.65445203	1.73515357
C	40.02359398	-20.93170028	0.93109196
O	40.88932531	-20.94818039	0.03376591
N	39.30962031	-22.02596215	1.22984005
H	38.37999082	-20.47256001	3.16228499
H	40.71284994	-19.57771837	2.37427582
H	39.89463169	-18.82279543	1.01776252
H	39.51823071	-22.90897329	0.73453273
H	38.63170234	-22.02231448	1.97919981
H	38.64300176	-18.77598235	3.35198124
H	37.65100471	-19.36598138	1.99400314
C	39.25300882	-18.03510128	-3.47805525
C	40.07882511	-18.94363739	-2.58386250
C	41.44891782	-19.29618872	-3.16589412
O	41.76083584	-19.06329310	-4.33751815
N	42.27812798	-19.87902491	-2.26614351
H	39.81805344	-17.11495799	-3.70396988
H	39.55801013	-19.89687237	-2.38255052
H	40.23411969	-18.48442399	-1.59215059
H	43.08175682	-20.40354699	-2.58853044
H	41.91516541	-20.14332212	-1.35178724
H	38.32703875	-17.73294028	-2.98897021
H	39.02797180	-18.49500632	-4.43999032
C	47.22996939	-20.50602946	-7.93397958
C	45.87002133	-19.98753535	-7.48778409
O	44.82134696	-20.37825279	-7.99419746
C	47.54403567	-20.02738880	-9.36058715
O	47.63903177	-18.61928741	-9.43762604
H	48.03600244	-20.16299645	-7.28500051
H	46.72199929	-20.33220357	-10.02934352
H	48.47208609	-20.52724159	-9.71231380
H	48.35499446	-18.35664917	-8.82780583
H	47.18803538	-21.59500253	-7.90299757
N	45.93588474	-19.05287678	-6.50226460
C	44.78138773	-18.59610008	-5.74988287
C	44.45734768	-17.13758815	-6.15696660
O	45.32027163	-16.26067122	-6.13074719
C	45.08202327	-18.72831448	-4.24539059
C	45.30423803	-20.18294003	-3.79335584
C	46.63476871	-20.85369638	-4.05574101
O	46.55710909	-22.18647188	-3.77604956
N	47.68532319	-20.24018976	-4.45633234
H	46.83267370	-18.94992989	-6.03908242
H	43.93474223	-19.24776741	-6.00969705
H	44.21334850	-18.33911292	-3.69523032
H	45.95036716	-18.10172380	-3.98684195
H	44.53094428	-20.83739010	-4.22910384
H	45.15901862	-20.26463044	-2.70266812
H	48.47197946	-20.89241421	-4.55741058
N	43.17740577	-16.92670720	-6.57894794
C	42.65102506	-15.60808412	-6.94402452
H	42.50982666	-17.66832805	-6.38444905
H	43.47297121	-14.88533008	-6.85313716
H	42.26198406	-15.58399010	-7.96199359
H	41.86004901	-15.35196011	-6.23896346
N	41.68837015	-25.74299374	-1.04270268
C	41.21395952	-24.52172044	-0.66471717
O	40.11397888	-24.40748610	-0.08260939
N	41.95040597	-23.36857463	-0.92398791
C	43.19994115	-23.34966845	-1.50621886
O	43.85221582	-22.31845244	-1.70600755
C	43.68671342	-24.66558505	-1.86398939
N	44.92082297	-24.77093774	-2.43360591
C	45.46174610	-26.00346420	-2.73942337
C	46.76383615	-26.12293115	-3.24702731
C	47.32505990	-27.36537250	-3.53702049
C	48.73527631	-27.47349791	-4.05356676
C	46.54787521	-28.53045713	-3.32596535
C	47.11415371	-29.89559272	-3.62802217
C	45.25094269	-28.40912970	-2.80925052
C	44.67956958	-27.16332781	-2.49414664
N	43.39212536	-27.04692654	-1.95446997
C	42.88690482	-25.80620428	-1.59562692
C	42.59386260	-28.25153023	-1.67993737
C	42.95003910	-28.94193207	-0.35789503
H	41.54271718	-22.46517069	-0.61602704
H	47.34592022	-25.21338315	-3.39944366
H	48.77279482	-28.00909747	-5.01648910
H	49.19228230	-26.49036220	-4.21096922
H	49.37177832	-28.04016843	-3.35228675
H	46.37634876	-30.69032974	-3.44585944
H	47.43752467	-29.97188140	-4.67886103
H	48.00005834	-30.10613536	-3.00557755
H	44.67168765	-29.31786298	-2.65725010
H	42.71265029	-28.92947096	-2.53671225
H	41.54809326	-27.92736228	-1.65850907
H	42.53995605	-28.38706105	0.48601603
H	42.48229155	-29.94169178	-0.35363703
H	44.02100617	-29.08499509	-0.21600902
O	46.77434445	-29.53403077	-0.24298211
H	46.21269688	-30.32335934	-0.25438891
H	46.52975586	-29.06234694	-1.05898172
H	47.41943596	-22.62772929	-3.96326178
H	45.40853712	-23.89496619	-2.64923630

RsLOV **Qa** S_0'

C	51.38383352	-15.19412495	-7.59191469
C	51.62953761	-16.16994362	-6.44571730
O	52.66899947	-16.79442406	-6.27993073
H	50.77608324	-14.39092570	-7.17603160
H	52.34052016	-14.81826083	-7.97312277
H	50.83305515	-15.68294630	-8.39506928
N	50.58575417	-16.24760647	-5.55068590
C	50.61771785	-17.21153590	-4.46585348
C	50.54745586	-18.63855480	-5.00825484
O	49.80665645	-18.92788215	-5.95783974
C	49.42884660	-16.98916107	-3.52206834
H	49.66340002	-15.95840782	-5.86205094
H	51.57322024	-17.08370457	-3.93354938
H	49.39630865	-17.78280698	-2.76009260
H	49.51398275	-16.01086167	-3.02530006
H	48.48068331	-17.01961178	-4.08462572
N	51.27549189	-19.56955819	-4.36898438
C	51.09679312	-20.96146269	-4.68622006
C	51.74409205	-21.85339798	-3.64211217
O	52.30195875	-21.43428208	-2.63760351
H	51.81332669	-19.35581731	-3.53352747
H	50.02265761	-21.19899130	-4.67090766
H	51.48671860	-21.19545222	-5.69210026
N	51.60431025	-23.17886411	-3.95056613
C	51.69278175	-24.23731990	-2.96086631
C	52.04420159	-25.53012267	-3.69749923
O	51.28287889	-25.92904252	-4.59583393
C	50.31231087	-24.48920790	-2.27598224
O	49.31064766	-24.59817465	-3.29868006
C	49.89479685	-23.39985605	-1.30449238
H	51.00377011	-23.40333925	-4.73760198
H	52.44085206	-23.94741125	-2.21047172
H	50.38625911	-25.45578316	-1.73535817
H	49.69462248	-25.18064920	-3.99144077
H	48.92304088	-23.66098904	-0.85738975
H	50.63740996	-23.30504590	-0.49819918
H	49.79135328	-22.42806547	-1.80621154
N	53.12485070	-26.21736014	-3.28668142
C	53.45843610	-27.54075846	-3.79979163
H	53.69823912	-25.81750629	-2.55326424
H	52.79018911	-27.74808846	-4.63612734
H	54.50337119	-27.57257571	-4.14258626
H	53.30726233	-28.29112100	-3.02406590
C	48.03901954	-30.39194240	4.83695317
C	47.46641595	-28.97543339	4.75095219
O	47.34313019	-28.24616739	5.72567471
H	48.62602512	-30.56102951	3.93401943
H	47.25145298	-31.15232042	4.94103542
H	48.70097626	-30.39703277	5.70302021
N	47.12330938	-28.55782633	3.48728632
C	46.65103424	-27.19449979	3.26715474
C	45.35380993	-26.88802314	4.03619940
O	45.09919545	-25.77352187	4.46851594
C	46.40179234	-27.00342727	1.75232957
S	45.94474655	-25.25137511	1.37138821
H	47.22224065	-29.18295146	2.68667287
H	47.39356528	-26.46988474	3.63706662
H	45.58571027	-27.67072074	1.44644006
H	47.30634554	-27.26841791	1.18480543
N	44.48648577	-27.93055666	4.08289519
C	43.17401967	-27.77693783	4.66402537
H	44.82006350	-28.84427178	3.80611190
H	42.52589660	-27.18976164	3.99220347
H	42.70599684	-28.75200332	4.80097229
H	43.21398837	-27.24404650	5.61402588
C	39.62402843	-24.39405384	6.51676861
C	39.20272973	-25.69846549	5.82816274
C	39.73184554	-25.81358250	4.39554953
C	39.84815336	-27.26102510	3.90224563
O	39.73586687	-28.22856855	4.64895762
N	40.10924862	-27.38361991	2.56918945
H	39.15999079	-23.50696200	6.08609368
H	38.10513082	-25.81514082	5.83312193
H	39.59669315	-26.55591292	6.39644439
H	40.74807536	-25.38218006	4.32479702
H	39.12084262	-25.23626446	3.67994588
H	40.36575856	-28.31066838	2.24826561
H	40.45556449	-26.58064958	2.04063829
H	39.36001434	-24.40594011	7.58683443
H	40.70800940	-24.31397942	6.43610570
C	39.31107380	-20.01214315	3.83697798
C	40.57019365	-20.08985468	2.96362813
C	40.86245009	-21.40414528	2.24421295
O	41.75921249	-21.45046628	1.37650287
N	40.17506869	-22.50004009	2.59149432
H	39.16219290	-20.95064043	4.40105742
H	41.47162767	-19.90911467	3.57547954
H	40.57911388	-19.30269770	2.19330246
H	40.47029901	-23.40335327	2.19830324
H	39.48714822	-22.47782883	3.33079389
H	39.40495217	-19.26496805	4.62497434
H	38.39799285	-19.83391461	3.26804240
C	40.10715895	-18.46907632	-2.21803520
C	40.94372405	-19.33582716	-1.28822194
C	42.32865748	-19.69343261	-1.83746200
O	42.69051555	-19.41635217	-2.98490635
N	43.10952318	-20.33348492	-0.93323372
H	40.66203855	-17.55289989	-2.47978685
H	40.43103575	-20.28492910	-1.05051132
H	41.08831310	-18.83353794	-0.31512804
H	43.95565860	-20.79995520	-1.23570757
H	42.72356198	-20.60461820	-0.02948486
H	39.18000903	-18.16299168	-1.73299556
H	39.88188857	-18.96897916	-3.15998404
C	47.89429586	-21.35966492	-6.71946355
C	46.61509353	-20.89755300	-6.03031761
O	45.62137163	-21.62274168	-5.91681131
C	48.14021686	-20.79328693	-8.12033988
O	48.18764887	-19.38145013	-8.15224149
H	48.68982104	-21.07137079	-6.03271684
H	47.31528113	-21.09515196	-8.78812516
H	49.06844620	-21.25106695	-8.52447900
H	48.87800483	-19.09787986	-7.52467468
H	47.77591039	-22.44398916	-6.70580279
N	46.71251247	-19.67789305	-5.44693393
C	45.65178916	-19.09799348	-4.64530378
C	45.39984089	-17.69382630	-5.18551825
O	46.31963265	-16.88033101	-5.30205806
C	46.01463679	-19.06683652	-3.15220209
C	46.14454277	-20.47730922	-2.57172673
C	47.41089324	-21.22789179	-2.96228198
O	48.53379048	-20.73489899	-2.82878997
N	47.21398861	-22.51920324	-3.36254108
H	47.53251871	-19.10462396	-5.62952622
H	44.76435897	-19.73144330	-4.77516462
H	45.20823157	-18.54014900	-2.61965958
H	46.95246186	-18.51113606	-3.00542882
H	45.25472133	-21.07142874	-2.82560670
H	46.19023521	-20.41521397	-1.47127868
H	48.03101902	-23.07470261	-3.61603356
H	46.34067999	-22.73981893	-3.83248176
N	44.11446125	-17.45225895	-5.54891982
C	43.66995390	-16.12811761	-5.96399466
H	43.42015138	-18.12734137	-5.24068984
H	44.54599736	-15.48498940	-5.87902800
H	43.27423669	-16.12188920	-6.99135868
H	42.90906639	-15.80293613	-5.25496181
C	45.34240880	-27.84770730	-1.33702070
N	44.11424567	-27.62888663	-0.66428745
C	43.82559239	-26.42644558	-0.10998336
N	42.68651291	-26.27882259	0.52037013
C	42.28833190	-25.03024290	0.91089494
O	41.25322733	-24.88035185	1.57226100
N	42.99939324	-23.90031336	0.49931991
C	44.19559307	-23.94002865	-0.16781314
O	44.75092395	-22.96401270	-0.64152198
C	44.86061689	-25.32971213	-0.23368009
N	45.58367219	-25.46430397	-1.45549507
C	46.10069890	-26.72810307	-1.73084437
C	47.31421790	-26.92703482	-2.40078258
C	47.79269748	-28.20884590	-2.68963205
C	49.10803802	-28.39441645	-3.40296689
C	47.03126619	-29.33520973	-2.29839568
C	47.51413535	-30.73559180	-2.58753239
C	45.81553041	-29.13394160	-1.63336451
C	43.13342045	-28.72968839	-0.52193678
C	43.32611114	-29.48978573	0.79391062
H	42.56976963	-22.98115836	0.72437817
H	46.16353102	-24.65625476	-1.68148169
H	47.89219387	-26.04945288	-2.69585841
H	48.97809294	-28.96589068	-4.33750822
H	49.58116561	-27.44006529	-3.66325729
H	49.82089264	-28.96816879	-2.78550477
H	46.80485136	-31.49310004	-2.22197736
H	47.64686041	-30.90126190	-3.66968164
H	48.49456506	-30.93264746	-2.12093372
H	45.22927658	-30.00551405	-1.34543572
H	43.25271314	-29.38114871	-1.39642025
H	42.13622318	-28.27806371	-0.57022829
H	43.00397502	-28.86008158	1.62304940
H	44.37897643	-29.74508994	0.91603865
H	42.72575928	-30.41264651	0.79346313
O	46.94268769	-30.12008002	1.06202573
H	47.00169677	-31.05993660	0.83728668
H	46.98368236	-29.66567060	0.20216638

RsLOV **Qb** S_0'

C	51.38384167	-15.19416009	-7.59188257
C	51.59835626	-16.17752769	-6.44808595
O	52.63702479	-16.79383487	-6.24316254
H	50.77607266	-14.39093904	-7.17602054
H	52.34730823	-14.82654267	-7.96246402
H	50.83305965	-15.68293413	-8.39507960
N	50.51806578	-16.28729138	-5.60309122
C	50.53195129	-17.25122552	-4.51729748
C	50.57687859	-18.67218973	-5.08455648
O	49.80150311	-19.03433668	-5.97189776
C	49.28575892	-17.08634976	-3.64281683
H	49.60465182	-15.98476784	-5.92751281
H	51.44786138	-17.07529038	-3.93041944
H	49.24551473	-17.87372794	-2.87597760
H	49.30212653	-16.10175046	-3.15112167
H	48.37165147	-17.15813054	-4.25478140
N	51.48961928	-19.50406695	-4.53259377
C	51.63261467	-20.88033421	-4.92236957
C	51.41692414	-21.82779887	-3.73742450
O	50.99036299	-21.42913303	-2.65224627
H	52.10084753	-19.14396670	-3.81061458
H	50.88321454	-21.08592814	-5.70178341
H	52.62752404	-21.06577114	-5.36285772
N	51.76614662	-23.10560905	-3.98844405
C	51.73122394	-24.22066536	-3.05098939
C	52.21942398	-25.42381313	-3.85524294
O	51.70880979	-25.64318398	-4.96102556
C	50.30158535	-24.65719744	-2.56469275
O	49.37169916	-24.46713475	-3.62210611
C	49.79942964	-23.97465102	-1.30131748
H	51.95367624	-23.37009432	-4.95197766
H	52.36470710	-23.99796186	-2.17772865
H	50.38490330	-25.74113956	-2.33938443
H	49.77537170	-24.83188673	-4.43180543
H	48.79861279	-24.35409989	-1.04714229
H	50.47765786	-24.21132607	-0.46599384
H	49.76105630	-22.88726385	-1.41306755
N	53.10831497	-26.23739987	-3.25874974
C	53.45869804	-27.54086900	-3.79991027
H	53.49772645	-25.95817314	-2.36626182
H	52.79009783	-27.74805698	-4.63606134
H	54.50339447	-27.57089531	-4.14688065
H	53.30715861	-28.29105702	-3.02402322
C	48.03901944	-30.39198818	4.83699235
C	47.46494372	-28.97511392	4.74593172
O	47.35182865	-28.23856222	5.71638844
H	48.62600449	-30.56100818	3.93400128
H	47.25208540	-31.15320997	4.94011405
H	48.70099250	-30.39701319	5.70300740
N	47.10793784	-28.56573150	3.48285830
C	46.64929198	-27.19757219	3.25213552
C	45.33382814	-26.88103694	3.98535385
O	45.05505582	-25.75359417	4.36732752
C	46.45382083	-27.00196267	1.72978384
S	46.09680394	-25.24041776	1.29770512
H	47.20931677	-29.18937229	2.68166169
H	47.38733205	-26.48196665	3.64719673
H	45.62502168	-27.64094309	1.39924639
H	47.36546337	-27.31187083	1.19728200
N	44.48226018	-27.93382142	4.07336708
C	43.17402210	-27.77696900	4.66403434
H	44.83325599	-28.85376987	3.84314397
H	42.52454471	-27.18944144	3.99425273
H	42.70599007	-28.75200105	4.80096634
H	43.21399811	-27.24402295	5.61401238
C	39.62405886	-24.39401076	6.51680449
C	39.19403641	-25.70607109	5.84963220
C	39.72725604	-25.85331423	4.42258280
C	39.82539366	-27.31029171	3.95396023
O	39.68662968	-28.26383101	4.71409000
N	40.09933814	-27.45669278	2.62542436
H	39.15995581	-23.50698772	6.08607945
H	38.09541396	-25.81255055	5.85393235
H	39.57794620	-26.55581868	6.43617655
H	40.74950975	-25.43789184	4.34913941
H	39.12890231	-25.27975293	3.69370347
H	40.35268217	-28.39155863	2.32478214
H	40.48163774	-26.66534879	2.10340474
H	39.36452012	-24.39323742	7.58844292
H	40.70800892	-24.31400276	6.43608744
C	39.31106084	-20.01205144	3.83696528
C	40.57315212	-20.10753100	2.97501280
C	40.88717225	-21.44768620	2.31928136
O	41.74042924	-21.50091878	1.40941530
N	40.27845299	-22.55345095	2.76605444
H	39.14646237	-20.94398052	4.40732007
H	41.46829915	-19.88654066	3.58329807

H	40.57521014	-19.35765662	2.16899408
H	40.57757541	-23.46564670	2.39290126
H	39.62834578	-22.52246128	3.53847884
H	39.40496985	-19.26499038	4.62499333
H	38.39798878	-19.83396944	3.26803124
C	40.10708090	-18.46898932	-2.21797716
C	40.91628319	-19.34979215	-1.27883091
C	42.28792136	-19.74517191	-1.82778105
O	42.64411734	-19.48511426	-2.98107902
N	43.05878767	-20.38989088	-0.92114596
H	40.67372718	-17.55848742	-2.47208812
H	40.37647128	-20.28278017	-1.03678899
H	41.07212092	-18.84420943	-0.30907282
H	43.89911675	-20.87003732	-1.21704525
H	42.66912732	-20.65375178	-0.01750867
H	39.17998083	-18.16303232	-1.73302413
H	39.88196588	-18.96899435	-3.15999522
C	47.89402847	-21.36001052	-6.71904746
C	46.57334076	-20.79915138	-6.20624015
O	45.48551960	-21.23898684	-6.56450505
C	48.20332474	-20.86031146	-8.13560923
O	48.18927355	-19.44975643	-8.23675966
H	48.69000948	-21.07099254	-6.03301144
H	47.42552039	-21.23135314	-8.82385034
H	49.17311688	-21.29318681	-8.46669247
H	48.76159002	-19.10530924	-7.52891805
H	47.77599945	-22.44400064	-6.70596249
N	46.72197352	-19.79559059	-5.29513478
C	45.60474250	-19.15157479	-4.63798074
C	45.38795059	-17.75520864	-5.23867958
O	46.32631230	-16.97083556	-5.39699949
C	45.84528457	-19.04243404	-3.12332252
C	46.02632716	-20.40857279	-2.44771769
C	47.29242993	-21.16321305	-2.76994429
O	48.38083767	-20.33485041	-2.75944365
N	47.27569290	-22.42308047	-2.97293289
H	47.64173676	-19.38596803	-5.17339802
H	44.72516667	-19.78494884	-4.81687938
H	44.96453026	-18.55397704	-2.68208355
H	46.72144376	-18.40389777	-2.93505953
H	45.19002830	-21.07388962	-2.69784780
H	46.02103841	-20.25860931	-1.35403074
H	48.20277223	-22.80145860	-3.19322126
N	44.09934700	-17.46502541	-5.56044483
C	43.67000613	-16.12803914	-5.96406043
H	43.39336925	-18.12943813	-5.25601288
H	44.54598323	-15.48497913	-5.87896143
H	43.27399512	-16.10882249	-6.99166714
H	42.90903109	-15.80299312	-5.25497338
C	45.34858711	-27.72799666	-1.40221227
N	44.16187283	-27.58722599	-0.63385277
C	43.86272867	-26.40171327	-0.04637556
N	42.74755990	-26.29974187	0.63428192
C	42.32789028	-25.06837022	1.05830217
O	41.29881299	-24.95759437	1.73983674
N	43.00443905	-23.91644983	0.66737230
C	44.18612926	-23.90294163	-0.02433677
O	44.71700125	-22.89086119	-0.44475643
C	44.85722957	-25.27292067	-0.21062496
N	45.46777523	-25.33273569	-1.49363796
C	46.00371145	-26.56158020	-1.85401825
C	47.14754136	-26.68560416	-2.65496395
C	47.66879698	-27.93609186	-2.99788500
C	48.91111386	-28.03494248	-3.84864429
C	47.02557960	-29.10792894	-2.53240775
C	47.56203965	-30.47451390	-2.88138337
C	45.87131543	-28.98261051	-1.74922628
C	43.18263711	-28.70221796	-0.51875257
C	43.32601911	-29.48993311	0.79397306
H	42.55481603	-23.00706836	0.90297497
H	45.95279761	-24.47920338	-1.78918128
H	47.63773719	-25.77630074	-3.00939258
H	48.71920207	-28.59902717	-4.77684250
H	49.28953465	-27.04460494	-4.12997520
H	49.71930135	-28.57033238	-3.32063860
H	46.94926526	-31.27585198	-2.44193798
H	47.58095160	-30.63355267	-3.97278309
H	48.59948763	-30.60825556	-2.52999075
H	45.36417016	-29.88739306	-1.41414557
H	43.33314422	-29.34443143	-1.39519460
H	42.18508820	-28.25285789	-0.59612874
H	43.00399608	-28.86004110	1.62302712
H	44.37899818	-29.74501512	0.91601007
H	42.71643174	-30.40503287	0.76130961
O	46.92742548	-30.05046311	0.98238267
H	47.00039457	-30.97743157	0.71219850
H	46.98452571	-29.55376961	0.14736146
H	49.22639566	-20.83081904	-2.77124042

RsLOV **Qc** $S_0^{'}$

C	51.38379071	-15.19415607	-7.59187553
C	51.61698775	-16.17248309	-6.44851834
O	52.62923794	-16.84375618	-6.29654276
H	50.77609764	-14.39091702	-7.17602551
H	52.34219246	-14.82150114	-7.97189544
H	50.83308066	-15.68295011	-8.39508362
N	50.58574940	-16.20257126	-5.53647368
C	50.59938233	-17.15462876	-4.44138106
C	50.47101526	-18.58013088	-4.98942021
O	49.61096475	-18.85169861	-5.83539411
C	49.43948242	-16.85741594	-3.48664171
H	49.67109468	-15.87378460	-5.83033708
H	51.56783988	-17.05252444	-3.92686947
H	49.44408230	-17.57226011	-2.65195670
H	49.54109652	-15.83956774	-3.08097494
H	48.47132640	-16.93893046	-4.00884434
N	51.30783245	-19.50249921	-4.47728852
C	51.28427941	-20.88912998	-4.87308131
C	51.50156657	-21.80546039	-3.67438247
O	51.63258678	-21.36315579	-2.52806250
H	51.99574187	-19.22671902	-3.78557655
H	50.29853951	-21.11648559	-5.29623293
H	52.04228412	-21.10049978	-5.64831725
N	51.53777293	-23.11117451	-3.99628487
C	51.69647604	-24.20791913	-3.06167545
C	52.22111798	-25.39231989	-3.89263287
O	51.85636891	-25.55175094	-5.05448732
C	50.32974144	-24.60943072	-2.39378031
O	49.28753595	-24.54019735	-3.34689432
C	50.01544746	-23.80378397	-1.13407256
H	51.32989830	-23.41716623	-4.94528367
H	52.41245440	-23.90942680	-2.28072085
H	50.41118568	-25.67671403	-2.11914306
H	49.01748246	-23.60196328	-3.45982914
H	49.06581269	-24.15551909	-0.70039985
H	50.80904945	-23.92557560	-0.37930390
H	49.93245305	-22.73316669	-1.36079576
N	53.04520846	-26.25294863	-3.23984486
C	53.45869804	-27.54087599	-3.79990527
H	53.33116632	-26.02360628	-2.29571172
H	52.79010777	-27.74805798	-4.63606933
H	54.50616948	-27.53645402	-4.13932254
H	53.30715173	-28.29105406	-3.02402222
C	48.03901843	-30.39199019	4.83699135
C	47.46560006	-28.97605443	4.75089383
O	47.34415235	-28.24579829	5.72522464
H	48.62600549	-30.56100818	3.93400228
H	47.25199470	-31.15295294	4.94108904
H	48.70099351	-30.39701118	5.70300740
N	47.11887471	-28.55907376	3.48801499
C	46.65289832	-27.19331777	3.26796652
C	45.34772677	-26.88465651	4.02269882
O	45.08464762	-25.76632295	4.44063571
C	46.42761137	-26.99598831	1.75059397
S	46.00803499	-25.23960420	1.35486795
H	47.22047759	-29.18017609	2.68459977
H	47.39351279	-26.47374461	3.65124242
H	45.60788465	-27.65185784	1.43065553
H	47.33654738	-27.27459812	1.19703087
N	44.48528263	-27.93051242	4.07965019
C	43.17402009	-27.77697498	4.66404033
H	44.82384251	-28.84585307	3.81459017
H	42.52495245	-27.18988886	3.99314274
H	42.70598906	-28.75200009	4.80096534
H	43.21399711	-27.24401898	5.61401042
C	39.62405187	-24.39401076	6.51682846
C	39.19921668	-25.70189016	5.83754072
C	39.72803833	-25.83146953	4.40631951
C	39.84346183	-27.28414718	3.92806399
O	39.73733745	-28.24356606	4.68621214
N	40.09629812	-27.42113111	2.59471717
H	39.15996083	-23.50698967	6.08607045
H	38.10119686	-25.81479574	5.84371092
H	39.59039721	-26.55579566	6.41305066
H	40.74480743	-25.40232822	4.33127499
H	39.11850922	-25.26059068	3.68455085
H	40.35667227	-28.35074345	2.28417533
H	40.44426555	-26.62297042	2.06002146
H	39.36214703	-24.40095716	7.58764672
H	40.70800791	-24.31400376	6.43607347
C	39.31105782	-20.01200445	3.83696928
C	40.56908179	-20.08822646	2.96072477
C	40.86318967	-21.40203947	2.23994143
O	41.75346356	-21.44422252	1.36490773
N	40.18467855	-22.50060774	2.59761398
H	39.16275838	-20.95072944	4.40065806
H	41.47134715	-19.90667326	3.57125193
H	40.57576112	-19.30084209	2.19067561
H	40.47690256	-23.40820302	2.21095901
H	39.50423419	-22.47731081	3.34392024
H	39.40497186	-19.26500340	4.62500533
H	38.39799079	-19.83399743	3.26802024
C	40.10713990	-18.46898133	-2.21798516
C	40.95800834	-19.34122064	-1.30603517
C	42.31054295	-19.72772604	-1.91402253
O	42.63556774	-19.43640555	-3.06771134
N	43.11354360	-20.40883733	-1.05775553
H	40.65949093	-17.55231150	-2.48337889
H	40.43800200	-20.27849304	-1.03997745
H	41.15215405	-18.83489359	-0.34370794
H	43.92334550	-20.90264046	-1.41399416
H	42.76305993	-20.68708926	-0.14262345
H	39.17997284	-18.16305232	-1.73302613
H	39.88193286	-18.96899038	-3.15999023
C	47.89402143	-21.36002555	-6.71899348
C	46.59673060	-20.85512997	-6.09893962
O	45.54902278	-21.49667721	-6.14213810
C	48.16527568	-20.83320700	-8.13105185
O	48.21255878	-19.42070502	-8.20616147
H	48.69000048	-21.07098550	-6.03300345
H	47.35268565	-21.15483930	-8.80461458
H	49.10174478	-21.29978243	-8.50732842
H	48.83547697	-19.11446311	-7.52395114
H	47.77600141	-22.44400064	-6.70601250
N	46.71556000	-19.66891106	-5.43308647
C	45.64565224	-19.13311968	-4.61296689
C	45.37674600	-17.69268624	-5.07188055
O	46.27414045	-16.84607112	-5.04534926
C	46.01099949	-19.20460073	-3.11859594
C	46.44535834	-20.63252098	-2.70018567
C	47.92088448	-20.97644782	-2.88560884
O	48.30982611	-21.92348698	-3.58385710
N	48.80366495	-20.20072880	-2.20640205
H	47.58100150	-19.13927644	-5.50751731
H	44.76697204	-19.76885296	-4.78337196
H	45.12057781	-18.91322907	-2.54216819
H	46.80421222	-18.47038550	-2.90241191
H	45.87077175	-21.38641525	-3.25589768
H	46.22885219	-20.78102409	-1.62759759
H	49.79819305	-20.43220574	-2.23110827
H	48.47994125	-19.50138032	-1.55127918
N	44.12443751	-17.45892283	-5.54950552
C	43.67000713	-16.12803818	-5.96411542
H	43.42608150	-18.16925803	-5.34963575
H	44.54597519	-15.48497310	-5.87892640
H	43.27543490	-16.13175989	-6.99105139
H	42.90903908	-15.80299513	-5.25496438
C	45.35537857	-27.77701281	-1.36835803
N	44.14529361	-27.60786914	-0.64098380
C	43.83799376	-26.41607963	-0.07140541
N	42.70159186	-26.29113422	0.57108367
C	42.28379854	-25.05052324	0.96707149
O	41.24035658	-24.91592008	1.61963270
N	42.98244851	-23.90488186	0.57535413
C	44.18031170	-23.91889193	-0.08235786
O	44.73583520	-22.92509097	-0.52312416
C	44.84821017	-25.29866948	-0.20234165
N	45.51073643	-25.38688561	-1.45758161
C	46.04941115	-26.62581920	-1.78634072
C	47.22323387	-26.75416976	-2.53560177
C	47.73784324	-28.00632352	-2.87651148
C	49.00983633	-28.09380573	-3.68484370
C	47.05487046	-29.17157637	-2.44997749
C	47.58294316	-30.54256503	-2.79468988
C	45.87172507	-29.03733294	-1.70899094
C	43.16774220	-28.71947919	-0.52303980
C	43.32602314	-29.48989210	0.79395605
H	42.54019661	-22.98995651	0.80080561
H	46.06662115	-24.56512462	-1.68385881
H	47.75232449	-25.85056950	-2.85014084
H	48.86215439	-28.69265969	-4.59918520
H	49.36365847	-27.09718007	-3.98481765
H	49.81416902	-28.59143136	-3.11447800
H	46.95225633	-31.34025650	-2.37421948
H	47.62436059	-30.69383389	-3.88659792
H	48.61129987	-30.68749922	-2.42214751
H	45.34054335	-29.93874866	-1.40371990
H	43.31749237	-29.36785732	-1.39504342
H	42.16893152	-28.27376377	-0.59962006
H	43.00399708	-28.86006105	1.62304212
H	44.37899521	-29.74502914	0.91601107
H	42.72050921	-30.40859550	0.77636349
O	46.92722651	-30.06503760	1.01575317
H	47.01344160	-30.99721174	0.76808106
H	46.98969604	-29.58725426	0.16979537

RsLOV **Qd** $S_0^{'}$

C	51.38378071	-15.19413511	-7.59191856
C	51.64681996	-16.18914630	-6.47026483
O	52.73124495	-16.72141141	-6.26419522
H	50.77610663	-14.39091306	-7.17601953
H	52.33644920	-14.81057072	-7.97512856
H	50.83307267	-15.68294614	-8.39508061
N	50.56679756	-16.42374534	-5.65520449
C	50.64986992	-17.42089911	-4.60350745
C	50.86360561	-18.81436024	-5.20714528
O	50.22486502	-19.21313467	-6.18444477
C	49.38179835	-17.40388988	-3.74449309
H	49.63835956	-16.15834279	-5.96723634
H	51.53341092	-17.18066217	-3.99000056
H	49.35997846	-18.27495320	-3.07343694
H	49.34220800	-16.47913173	-3.14884983
H	48.47445193	-17.43592902	-4.36859558
N	51.77317230	-19.58710074	-4.57188959
C	52.05444905	-20.94343664	-4.97029080
C	52.16370273	-21.83955015	-3.73955359
O	52.60843165	-21.43435659	-2.67257269
H	52.27831262	-19.23232782	-3.76736752
H	51.26205232	-21.26690173	-5.65934134
H	53.01475907	-21.00876187	-5.51600389
N	51.75957239	-23.12292251	-3.94955086
C	51.82074978	-24.16575619	-2.93929252
C	52.08238427	-25.48776653	-3.66588866
O	51.27424441	-25.84967826	-4.53740839
C	50.46396799	-24.32153073	-2.19119086
O	49.40856556	-24.35988968	-3.16611095
C	50.17440580	-23.20525122	-1.20310547
H	51.32747255	-23.37567937	-4.83137011
H	52.60775292	-23.89882622	-2.21976335
H	50.48792115	-25.28927299	-1.65172424
H	49.71126241	-24.94880193	-3.89143311
H	49.16521166	-23.33679653	-0.78460992
H	50.90446328	-23.23240860	-0.38092157
H	50.23538602	-22.21647252	-1.67616991
N	53.14857847	-26.21156856	-3.28136529
C	53.45893776	-27.54097399	-3.80000927
H	53.75459226	-25.82118587	-2.56897121
H	52.79002379	-27.74801898	-4.63601233
H	54.50227315	-27.58090533	-4.14649028
H	53.30704695	-28.29100104	-3.02399122
C	48.03901748	-30.39198119	4.83698435
C	47.46695047	-28.97543704	4.75518838
O	47.34190440	-28.25082362	5.73324766
H	48.62600650	-30.56101321	3.93400428
H	47.25145780	-31.15229354	4.94145244
H	48.70099250	-30.39701017	5.70300841
N	47.12753676	-28.55155484	3.49303921
C	46.65365123	-27.18771987	3.28011446
C	45.35815426	-26.88694176	4.05399859
O	45.10722206	-25.77813508	4.50248840
C	46.39945221	-26.98961305	1.76740266
S	45.94160689	-25.23633494	1.39655151
H	47.22570412	-29.17325810	2.68972674
H	47.39628287	-26.46406971	3.65170796
H	45.58306174	-27.65615598	1.46070915
H	47.30228744	-27.25227737	1.19584688
N	44.48759545	-27.92756745	4.08534590
C	43.17401808	-27.77697201	4.66403034
H	44.81669915	-28.83772929	3.79206835
H	42.52520455	-27.19030569	3.99216325
H	42.70599107	-28.75200105	4.80097234
H	43.21399711	-27.24402295	5.61401238
C	39.62405987	-24.39400674	6.51680644
C	39.19953991	-25.70171374	5.83703032
C	39.72499730	-25.82880444	4.40430506
C	39.84541254	-27.28080537	3.92521352
O	39.75243207	-28.24082572	4.68442799
N	40.08762892	-27.41616807	2.58998317
H	39.15995380	-23.50698867	6.08608045
H	38.10168661	-25.81629151	5.84561019
H	39.59355089	-26.55569993	6.41048172
H	40.73996710	-25.39575864	4.32697612
H	39.11142322	-25.25979882	3.68454609
H	40.34893676	-28.34427850	2.27599913
H	40.42116850	-26.61522480	2.05008978
H	39.36213941	-24.40139903	7.58761296
H	40.70800892	-24.31400477	6.43608649
C	39.31105882	-20.01204446	3.83697128
C	40.56840233	-20.09484181	2.96436275
C	40.85062790	-21.41146237	2.24747201
O	41.74891046	-21.46289946	1.38174511
N	40.15454461	-22.50231740	2.59363490
H	39.16058844	-20.94928410	4.40285393
H	41.47144511	-19.91859964	3.57507213
H	40.58101702	-19.30925918	2.19251366
H	40.43867003	-23.41008076	2.20131877
H	39.46344907	-22.47315974	3.32975930
H	39.40496985	-19.26499038	4.62499333
H	38.39798878	-19.83397542	3.26803024
C	40.10708090	-18.46898535	-2.21798516
C	40.93376758	-19.34747356	-1.29058598
C	42.29148749	-19.75475330	-1.86868789
O	42.62032828	-19.52160309	-3.03424234

N	43.08960357	-20.38488634	-0.97096834
H	40.66778748	-17.55576697	-2.47675872
H	40.39681793	-20.27816151	-1.03342520
H	41.11250074	-18.83958887	-0.32615496
H	43.90032275	-20.89752426	-1.29487686
H	42.72150592	-20.63652912	-0.05479608
H	39.17998183	-18.16303332	-1.73302013
H	39.88196186	-18.96899536	-3.15999423
C	47.89441646	-21.35978255	-6.71937047
C	46.56345668	-20.80467964	-6.23415023
O	45.48490798	-21.31381028	-6.53161949
C	48.23891011	-20.86215983	-8.13127347
O	48.31482376	-19.45362826	-8.20711172
H	48.68980151	-21.07129253	-6.03268642
H	47.44405181	-21.17570094	-8.82915915
H	49.18201885	-21.34993171	-8.46076500
H	48.97608323	-19.17080620	-7.54823418
H	47.77579244	-22.44397662	-6.70589550
N	46.68883311	-19.70530660	-5.44339299
C	45.60416644	-19.17148836	-4.64225298
C	45.36002824	-17.71808971	-5.08300631
O	46.25916235	-16.87654945	-5.03760860
C	45.93768151	-19.27566225	-3.14317145
C	46.09116482	-20.72730017	-2.66621906
C	47.37071927	-21.46371233	-2.99419505
O	47.19033058	-22.80771115	-2.86310794
N	48.46644289	-20.88280382	-3.31285218
H	47.62703045	-19.42602728	-5.18230268
H	44.71926947	-19.78667930	-4.85639456
H	45.10273421	-18.82443921	-2.58689904
H	46.84624979	-18.69572066	-2.92304601
H	45.26018805	-21.34195357	-3.04744552
H	46.00877748	-20.77088236	-1.56619854
H	49.20395754	-21.57757640	-3.48065632
N	44.11244937	-17.46577472	-5.56751644
C	43.66996517	-16.12801516	-5.96408642
H	43.40797137	-18.17696143	-5.39768626
H	44.54599620	-15.48499612	-5.87895841
H	43.27301492	-16.11768181	-6.99066499
H	42.90904210	-15.80299413	-5.25496238
C	45.34413344	-27.79255030	-1.32619726
N	44.11660786	-27.60547144	-0.64218423
C	43.81009834	-26.41178467	-0.07709801
N	42.66609952	-26.28481790	0.54916344
C	42.24736945	-25.04323055	0.94201768
O	41.19972725	-24.90860695	1.58815641
N	42.95184635	-23.90366904	0.55307121
C	44.15886896	-23.92045163	-0.09229051
O	44.71753805	-22.92735146	-0.52383679
C	44.83127586	-25.30143761	-0.19455962
N	45.53617520	-25.40579184	-1.42779380
C	46.07527484	-26.65288439	-1.72104741
C	47.28876543	-26.82640134	-2.40056789
C	47.78928397	-28.09663058	-2.70303158
C	49.10231465	-28.25377611	-3.42631065
C	47.05280656	-29.23992491	-2.31343013
C	47.55870647	-30.62874276	-2.61843703
C	45.84042328	-29.06653569	-1.63491497
C	43.14518073	-28.71909903	-0.51899420
C	43.32606409	-29.48986310	0.79397006
H	42.51578466	-22.98656496	0.78289677
H	46.06455752	-24.57015185	-1.68562515
H	47.85448914	-25.93776817	-2.68336867
H	48.97776919	-28.82781188	-4.35989029
H	49.55380186	-27.29039062	-3.69084639
H	49.83244924	-28.81178834	-2.81435723
H	46.86804623	-31.40183490	-2.24980357
H	47.68069352	-30.78460494	-3.70334463
H	48.54821977	-30.81113318	-2.16527995
H	45.27319798	-29.95082252	-1.34736439
H	43.28189711	-29.36258565	-1.39680463
H	42.14441807	-28.27618037	-0.57700458
H	43.00397311	-28.86006206	1.62303412
H	44.37899019	-29.74505317	0.91600407
H	42.72301354	-30.41056103	0.78447195
O	46.94546677	-30.09583148	1.05495449
H	47.01694189	-31.03075031	0.81369568
H	46.99176782	-29.62492956	0.20437986
H	48.02717370	-23.28739186	-3.06344025
